# Characterisation of Staphylococci Isolated from Milk Samples of a Water Buffalo Herd

**DOI:** 10.3390/antibiotics11111609

**Published:** 2022-11-12

**Authors:** Christiaan Labuschagne, Joanne Karzis, Hans Britz, Inge-Marié Petzer

**Affiliations:** 1 Inqaba Biotechnical Industries (Pty) Ltd., P.O. Box 14356, Hatfield 0028, South Africa; 2 Department of Production Animal Studies, Faculty of Veterinary Science, University of Pretoria, Private Bag X04, Onderstepoort 0110, South Africa

**Keywords:** *Staphylococcus simulans*, water buffalo, multi-locus sequence typing (MLST), mastitis, antibiotic resistance

## Abstract

Water buffalo produce a tenth of milk for global human consumption. Non-aureus staphylococci (NAS) are among the most commonly isolated bacteria from mastitis in water buffalo and dairy cows. These results described the initial characterisation of 17 NAS—15 *Staphylococcus simulans* and two *Staphylococcus chromogenes* from a water buffalo herd (*n* = 44) in South Africa. The isolates were identified by classical microbiology, MALDI-TOF, and 16S rRNA, and the disc diffusion method determined the antibiotic susceptibility. A multi-locus sequence typing scheme (MLST) was developed to determine *S. simulans* sequence types (ST), by defining and comparing seven housekeeping gene fragment sequences. Sequence typing confirmed all 15 *S. simulans* isolates from water buffalo which belonged to a single ST, genetically distant from the six bovine STs isolated from adjacent farms, which also varied, indicating no current bacterial transfer between species. The antibiotic resistance patterns of *S. simulans* varied between beta-lactams. The mean milk somatic cell count (SCC) for the water buffalo milk samples was 166,500 cells/mL milk. This information offers insights into the epidemiology and comparison among isolates from various origins, which leads to effective proactive mastitis strategies resulting in safe, high-quality dairy products from water buffalo and dairy cows for human consumption.

## 1. Introduction

Water buffalo (*Bubalus bubalis*) contribute 13% of the total global milk production [1]. Indian buffalo produce 66.7% and Pakistani buffalo 25.2% of the world’s water buffalo milk [2]. Water buffalo were introduced due to intensifying production in Europe, which is attributable to the popularity of buffalo mozzarella cheese and the absence of restrictions by quota in the European community [3]. Water buffalo milk is also double the price of bovine milk [3]. Water buffalo were introduced into South Africa at the century’s turn and kept in the same province as commercial dairy herds. Traditionally, the buffalo is less susceptible to mastitis than cattle [3].

Staphylococci are the most commonly isolated udder pathogens causing subclinical mastitis in dairy animals; to date, 52 NAS have been characterised [4]. Non-aureus staphylococci, originally known as minor pathogens, are emerging udder pathogens [5]. According to a comprehensive NAS study [6], milk quality and bulk somatic cell count (SCC) are unaffected by NAS intramammary infection (IMI). A recent study on NAS [7] describes lower milk yields for *Staphylococcus chromogenes*, which is shown as bovine-adapted and the most commonly isolated NAS in bovine mastitis [8]. *Staphylococcus simulans* has been isolated from bovine and water buffalo mastitis samples, however, this is generally with a lower prevalence to that of other NAS [9,10,11].

Multi-locus sequence typing (MLST) was designed for bacterial characterisation exploring global population dynamics [12]. Multi-locus sequence typing exploits high-throughput nucleotide sequencing technologies to identify this variation at the nucleotide level rather than the protein level. Most MLST schemes are contingent on the sequencing of internal fragments of seven or more housekeeping genes [12]. The loci are chosen based on their presence in organisms; the sequence variation within them is inclined to be selectively neutral [12]. Fragments of 400 to 500 base pairs are used in MLST, since such fragments can be rapidly, economically, and accurately sequenced on both strands with a single primer set. An MLST scheme for *Streptococcus uberis* relying on the six loci sequences was previously described [13]; although schemes specifically for *S. simulans* are now available, these were not available when this work commenced in 2019. This initial study aimed to:

- Identify and characterise mastitis-causing pathogens in a South African water buffalo herd.
- Compare these with the isolates of similar species established in dairy cattle near this water buffalo herd.
- Investigate antimicrobial resistance (AMR) patterns.

Benefits include insights into the IMI epidemiology as part of a proactive herd udder health approach for water buffalo farming, including improved antibiotic use management on the farm, while ensuring a better-quality product—mostly mozzarella cheese—for the consumer.

## 2. Results

The results of this study describe the initial characterisation of 15 *Staphylococcus simulans* and two *Staphylococcus chromogenes* isolated from one of the two water buffalo herds in South Africa. All the lactating animals in this water buffalo herd were sampled (*n* = 44) and only the 15 NAS were isolated. The antibiotic resistance patterns of *S. simulans* were also determined. The milk of water buffalos is used for specialty dairy products, which is a growing industry. It is important to know which organisms are present and how to handle them in practice, in order to produce high-quality products which are safe for human consumption.

### 2.1. Identifying Organisms

Only NAS—no other pathogens—were isolated from the milk samples of a South African water buffalo herd. Out of the 44 buffalo in this herd, only 15 *S. simulans* and two *S. chromogenes* were isolated.

### 2.2. Somatic Cell Counts

The milk sample SCC in the water buffalo herd of uninfected udders was 84,000 cells/mL milk compared to 143,000 cells/mL (ranging from 30,000 to 335,000 cells/mL) in quarters infected by *S. simulans*, whereas the average SCC for the milk from two *S. chromogenes* isolates was 350,000 cells/mL (Table 1).

### 2.3. Multi-locus Sequence Typing

**Figure 1:**
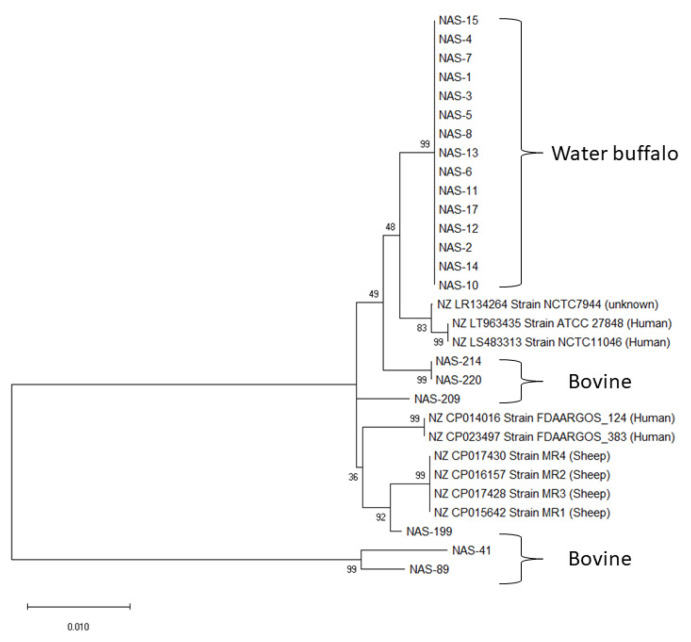
Phylogenetic demonstration of the relationship of 21 *S. simulans* isolates compared to 9 random whole *S. simulans* genomes published in NCBI. The dendrogram was constructed by using the combined nucleotide sequences of the seven housekeeping genes (*PyrR*, *glPk*, *Gmk*, *AdhP, Xpt, PTA*, *InfB)* with the maximum likelihood method.

The 21 *S. simulans* isolates’ diversity and phylogenetic relationships were evaluated by MLST. MEGA X software constructed the phylogenetic tree using ML and Tamura-Nei models [16] with seven concatenated housekeeping gene sequences (Figure S1, Data S1, and Image S1). The final dataset comprised 4112 positions. The 21 *S. simulans* isolates belonged to six STs (Figure 1 and Table 1). Sequence type 1 comprised water buffalo isolates only, whereas ST2-6 originated from bovine isolates. Sequence types 3–6 represented a single isolate each.

### 2.4. Antibiotic Resistance

Two of the 14 *S. simulans* isolated from buffalo milk indicated no resistance against the 10 antibiotics tested, and no multiple resistance (three antibiotic groups) was present; two out of a total of 56 (37.5%) resistance cases against βeta-lactam were established; no resistance was recognised against tetracyclines, with four out of 70 (5.7%) showing resistance against the cephalosporins in the water buffalo strains. Similarly, the bovine *S. simulans* sequence types (STs) were resistant to βeta-lactams in 12 out of 24 (50%), and cephalosporins in two out of 30 (6.7%) cases (Table 1). In this study, nine out of 14 and eight out of 14 buffalo, and four out of six and all six bovine *S. simulans* isolates were resistant to penicillin and oxacillin, respectively; four out of 14 buffalo and two out of six bovine isolates were resistant to a first-generation cephalosporin, cephalexin (Table 1).

## 3. Discussion

### 3.1. Bacterial Isolation and Identification

The importance of NAS as a cause of IMI increased; it is the most commonly isolated bacteria from subclinical mastitis in bovines [3]. The present data confirmed *S. simulans* as the predominant NAS isolated from the water buffalo; their prevalence of 38.5% is higher than that reported in cattle [9]. *Staphylococcus Simulans,* which was the predominant NAS in this water buffalo study, contradicted the results of a study in Egypt, which established *S. intermedius* as the predominant isolated NAS [10]. A study conducted in Italy reported a high prevalence of *Staphylococcus rostri* and a low incidence of *S. epidermidis* [11]. This difference in the prevalence and distribution of NAS species may be linked to differences in regional and environmental conditions [6].

### 3.2. Somatic Cell Count

A study in South Africa (*n* = 263,567) on dairy cattle reported an SCC exceeding 200,000 cells/mL in over 60% of the samples from which NAS were isolated; 26.7% of these had an SCC higher than 750,000 cells/mL [17]. The current study revealed comparable results for the water buffalo; 21.4% samples where *S. simulans* were isolated had an SCC higher than 200,000 cells/mL (Table 1). Another study [18] determined that the mean SCC of 168,000 cells/mL was observed in bovine milk, from which *S. chromogenes* were isolated, and 39,000 cells/mL in uninfected samples.

These results correlate with the current water buffalo study with an average SCC of 143,000 cells/mL per milk from milk samples out of which *S. simulans* was isolated, and 84,000 cells/mL in uninfected samples. According to [6], like *S. aureus,* host-adapted NAS species (*S. chromogenes*, *S. simulans*, and *S. xylosus*) hold the ability of adhesion in mammary cells, which stimulates the immune system, leading to an increase in SCC. The variation of results highlights the challenge of interpreting SCC to determine specific pathogens [17].

### 3.3. Multi-Locus Sequence Typing

The MLST analysis indicated that all the *S. simulans* isolated from water buffalo were the same ST and genetically dissimilar from *S. simulans* isolated from dairy cattle (Figure 1). These results agree with those of a similar study [8], where *S. simulans* isolated from dairy cows genetically differed from the water buffalo ST and formed the other bovine STs. Two isolates from the same farm were identified as the same ST among the cattle *S. simulans*, whereas the remaining four isolates were unique STs (Figure 1). The presence of a single ST amongst the water buffalo herd may indicate that the infection is spread between animals and that this particular ST may be adapted towards water buffalo. A longitudinal study may determine whether this ST persists within this herd over time and poses a higher risk for other water buffalo herds or dairy cattle herds in close proximity.

Establishing this MLST scheme for *S. simulans* specifically may be a valuable device for the population biology of this organism, providing insights into the epidemiology of this disease on a global scale. The MLST scheme can be used for strain tracing and initial surveillance of this mastitogenic pathogen (*S. simulans*) from water buffalo and dairy cows. This, combined with the antibiotic resistance profiles, can monitor how resistance is transferred among bacterial strains, species, herds, and animals (i.e., dairy cows and buffalo).

### 3.4. Antibiotic Resistance

Non-aureus staphylococci can produce a protective biofilm, facilitating persistent and recurrent infections while reducing their susceptibility to commonly used antibiotics [19]. *Staphylococcus simulans* holds several resistance genes to access, potentially forming a resistance reservoir to transfer to other closely related higher infection organisms through lateral gene transfer [20].

This study indicated the resistance of *S. simulans* isolates from water buffalo and bovine to all four beta-lactams evaluated and against one first-generation cephalosporin/aminoglycoside (cephalexin-kanamycin) combination (Table 1). These beta-lactams are penicillin, oxacillin, and amoxicillin with clavulanic acid. The *S. simulans* antibiotic resistance profiles agreed with water buffalo studies from Brazil and Slovakia, establishing penicillin and oxacillin resistance [21,22]; however, [11] reported no AMR from Italy. Beta-lactamase genes are usually mobile and carried in cassettes with various plasmid profiles. Plasmids often carry resistance and virulence genes that can disseminate through *S. aureus* populations by horizontal gene-transfer mechanisms [23]; genetically, the same bacterial strain may acquire or lose different plasmids.

This study indicated no cefoxitin resistance in a screening test for methicillin resistance; however, intermediate resistance in six isolates was established (two of bovine and four of buffalo origin) (Table 1). These findings disagreed with [24], who established a 60.7% resistance of NAS to methicillin in Ethiopia. A study in South Africa [25] reported 9.0% resistance in dairy cows. Conversely, [26] established erythromycin and tetracycline resistance in Poland.

This study observed no multi-drug resistance from water buffalo or dairy cattle, unlike [24,25], reporting a prevalence of 51.0% and 87.5% multi-drug resistance, respectively, in NAS isolated from dairy cattle milk.

The differences in antibiotic resistance patterns established between isolates of the same *S. simulans* ST from water buffalo and *S. simulans* STs may be linked to mobile genetic elements; they should be investigated further through screening for antibiotic resistance genes linked to integrons and plasmids and their transfer among *S. simulans* strains. The low sample numbers are a limitation of this preliminary study; however, the results provide insight into the characterisation and antibiotic resistance profiles of *S. simulans* isolated from water buffalo in South Africa.

The variation in resistance warrants further investigation of the influx of plasmids into these bacterial populations and monitoring of antibiotic resistance among STs and plasmids. Resistance is adaptable; therefore, the same base strain may become dangerous [23].

## 4. Materials and Methods

### 4.1. Milk Sample Collection

During a routine udder health investigation of a South African water buffalo herd from the Western Cape in 2019, forty-four composite milk samples were collected by the local veterinarian from all lactating animals and investigated. Another six *S. simulans* isolates from bovine milk were collected: four originating from the same province as the buffalo herd and two from another province, Gauteng, in South Africa. These bovine *S. simulans* isolates were the total of *S. simulans* isolated from a larger study on a total of 2140 NAS isolated from 17 herds of dairy cattle throughout South Africa [27]. They were submitted within 48 h after collection on ice to the South African National Accreditation System (SANAS) milk laboratory, Faculty of Veterinary Science, University of Pretoria [17].

### 4.2. Somatic Cell Count

Fluoro-opto-electronic means determined the milk samples’ SCC, using a Fossomatic Foss Electric (FC) (Rhine Ruhr, P.O. Box 76167, Wendywood 2144, South Africa). The Fossomatic FC counts somatic cells, recognising DNA from the cells.

### 4.3. Bacterial Identification

Classical microbiological phenotypical identification was conducted, as recommended by the National Mastitis Council [28], for isolates of water buffalo and bovine origin. This included catalase, coagulase (Staphylase test, Oxoid, supplied by Quantum Biotechnologies [Pty] Ltd., Ferndale, South Africa) and maltose agar tests (Merck NT Laboratory Supplies, Halfway House, South Africa). NAS used in the study was further identified with a MALDI-TOF MS (Bruker Daltonics, Bremen, Germany (Department of Plant and Soil Sciences, University of Pretoria, South Africa) and confirmed by 16S rRNA sequencing. All MALDI-TOF MS identifications (with scores of >2) were carried out by using the direct transfer method. Biological material (single colony) was smeared as a thin film directly onto a spot on a MALDI target plate. The material was overlaid with 1μL of α-cyano-4-hydroxy-cinnamic acid (HCCA) solution within 1 h and allowed to dry at room temperature. The screening was automated without any user interference. Flex Control software (Bruker Daltonics) recorded the spectra set for bacterial identification. MALDI Biolayer 3.0 software (Bruker Daltonics) with an integrated pattern-matching algorithm was used to compare generated peak lists against the reference library [29]. The MALDI-TOF identification of isolates was performed in duplicate (Table S1).

The isolates were submitted to Inqaba Biotec™ (Inqaba Biotec, Pretoria, South Africa). for identification using the genetic 16S rRNA Sanger sequencing analysis. Genomic DNA was extracted from the cultures using the Quick-DNA™ Fungal/Bacterial Miniprep Kit (Zymo Research, Catalogue No. D6005) (Distributor: Inqaba Biotec, Pretoria, South Africa), following the manufacturer’s instructions. The 16S target region was amplified using OneTaq^®^ Quick-Load^®^ 2X Master Mix (New England Biolabs, Catalogue No. M0486) (Frankenwald, Gauteng, South Africa) published primers [30]. The PCR products were run on a gel and gel-purified with the Zymoclean™ Gel DNA Recovery Kit (Zymo Research, Catalogue No. D4001) (Distributor: Inqaba Biotec, Pretoria, South Africa). The purified fragments were sequenced in the forward and reverse directions (Nimagen, BrilliantDye™ Terminator Cycle Sequencing Kit V3.1) and purified (Zymo Research, ZR-96 DNA Sequencing Clean-up Kit™, Catalogue No. D4050). The purified fragments were analysed on an ABI 3500xl Genetic Analyzer using a 50 cm array and POP-7 (Applied Biosystems, ThermoFisher Scientific, Johannesburg, South Africa). CLC Bio Main Workbench v7.6 was used to analyse the ab1 files, and the identification results were obtained by a BLAST search (BLASTN 2.2.31+) [31].

### 4.4. Multi-Locus Sequence Typing

The 15 water buffalo NAS isolates, identified as *S. simulans* and six bovine origin *S. simulans*, were further characterised using MLST (Inqaba Biotec, Inqaba Biotec, Pretoria, South Africa). Seven housekeeping genes (*PyrR*, *glPk*, *Gmk*, *AdhP*, *Xpt*, *PTA*, *InfB*) were selected and extracted from nine random whole *S. simulans* genomes published in NCBI (NZ_CP015642, NZ_CP017428, NZ_LT963435, NZ_CP014016, NZ_LR134264, NZ_CP016157, NZ_CP017430, NZ_CP023497, NZ_LS483313). Primers were designed (Table 2) requiring only one primer set for polymerase chain reaction (PCR) amplification and sequencing, with the same annealing temperature for all seven targets. Inqaba Biotechnical Industries (Pty) Ltd. synthesised Oligo.

Genomic DNA was extracted from the cultures using the Quick-DNA™ Fungal/Bacterial Miniprep Kit (Zymo Research, Catalogue No. D6005), following the manufacturer’s instructions. OneTaq^®^ Quick-Load^®^ 2X Master Mix (NEB), Catalogue No. M0486, was employed to amplify the seven targets. The amplification conditions comprised initial denaturation at 94 °C for 5 min, followed by 35 cycles of 94 °C for the 30 s, 53 °C for 30 s, and 68 °C for 1 min, with a final extension at 68 °C for 5 min. Amplicons were visualized on a 1% agarose gel stained with ethidium bromide (Figure S1).

Amplicons were sequenced in the forward direction (NimaGen, BrilliantDye™ Terminator Cycle Sequencing Kit V3.1, BRD3-100/1000) and purified (Zymo Research, ZR-96 DNA Sequencing Clean-up Kit™, Catalogue No. D4050). The purified fragments were analysed on the ABI 3500xl Genetic Analyzer (Applied Biosystems, ThermoFisher Scientific). CLC Main Workbench Version 7.7.3 (CLC bio, Aarhus, Denmark) was used to analyse, concatenate (combine), and align the sequences. MEGA X was employed to construct the maximum likelihood (ML) phylogenetic tree. Bootstrapping was performed with 1000 replicates.

### 4.5. Antibiotic Susceptibility Testing

Fourteen of 15 *S. simulans* isolates were identified from water buffalo, as one could not be recovered, and six *S. simulans* isolated from dairy cattle were assessed for antimicrobial susceptibility with the Kirby–Bauer disc diffusion method. A *Staphylococcus aureus* American Type Culture Collection (ATCC) No. 25923 isolate was used as a positive control; ten antibiotics were used in the test: ampicillin (AMP) 10 μg oxacillin (OX) 5 μg; penicillin (PEN) 10 IU (beta-lactams); amoxicillin; clavulanic acid (AMC) 30 μg; Ubrolexin ^®^ (cephalexin and kanamycin) 30 μg (CFX/K); cephalothin (KF) 30 μg; cefuroxime (CXM) 30 μg (cephalosporins); tetracycline (TE) 30 μg (tetracyclines); cefoxitin (FOX) 30 μg; ceftiofur (EFT) 30 μg.

These antibiotics belong to three antibiotic groups: βeta-lactams, cephalosporins, and tetracyclines. They were selected because of their presence in intramammary treatments in South Africa. Antibiotic susceptibility was interpreted by measuring the zone of inhibition diameter to the nearest millimetre, categorised as susceptible, intermediate, or resistant categories, as clinical breakpoints established by the Clinical Laboratory and Standards Institute (CLSI), [14,15].

## 5. Conclusions

This initial study on one of only two water buffalo herds in South Africa identified 17 NAS isolates from 44 lactating water buffalo; 15 were *S. simulans* and two *S. chromogenes*. The MLST scheme for *S. simulans* was specifically developed in this study to differentiate among *S. simulans* STs for characterising this organism. According to this scheme, all *S. simulans* isolates from the water buffalo herd were from a single ST, differing genetically from those of dairy cows of adjacent herds. The *S. simulans* STs of bovine origin differed among themselves and within herds. The antibiotic resistance patterns differed among the same STs of the *S. simulans* of water buffalo origin and STs of the *S. simulans* of bovine origin.

The MLST scheme characterisation and antibiotic resistance patterns of *S. simulans* can be used for the tracing, initial surveillance, and chronicity of STs. This could be used to monitor resistance transfer among bacterial STs, strains, and species, and among herds and various animal species (i.e., dairy cows and water buffalo). Such information forms the foundation for effective proactive udder health management strategies and prudent treatment, resulting in safe and good-quality dairy products from water buffalo and dairy cows for human consumption.

## Figures and Tables

**Table 1:** Antibiotic patterns (disc method) per product and somatic cell count (SCC) × 10^3^ cells/mL of milk for each isolate of *S. simulans* indicating the species of animal and sequence type of isolate.

Isolate	Origin	ST	Beta-Lactams				Tetracyclines	Cephalosporins					SCC × 10^3^ Cells/mL
			P	AMP	AMC	OX	TE	CFX-K	KF	FOX	CXM	EFT	
NAS 1	Buffalo	1	S	R	R	R	I	I	S	S	S	S	254
NAS 2	Buffalo	1	R	S	S	R	S	I	S	I	I	S	58
NAS 4	Buffalo	1	R	S	S	R	S	I	S	I	I	S	80
NAS 5	Buffalo	1	R	S	R	S	S	I	S	S	S	S	191
NAS 6	Buffalo	1	S	S	S	S	S	I	S	S	S	S	52
NAS 7	Buffalo	1	R	S	S	S	S	I	S	I	I	S	154
NAS 8	Buffalo	1	S	S	S	S	S	I	S	S	S	S	177
NAS 10	Buffalo	1	R	S	R	R	S	R	S	S	S	S	335
NAS 11	Buffalo	1	R	S	S	R	S	R	S	S	S	S	314
NAS 12	Buffalo	1	S	S	S	R	S	R	S	S	S	S	189
NAS 13	Buffalo	1	R	S	S	S	I	I	S	S	S	S	30
NAS 14	Buffalo	1	R	S	S	R	S	R	S	S	S	I	58
NAS 15	Buffalo	1	R	S	S	S	S	S	S	I	I	S	51
NAS 17	Buffalo	1	S	S	S	R	I	I	S	S	S	S	58
NAS 214	Bovine	2	R	S	R	R	S	R	S	I	I	S	110
NAS 220	Bovine	2	R	S	S	R	S	I	S	S	S	S	48
NAS 199	Bovine	3	R	S	S	R	I	R	I	I	I	S	1136
NAS 209	Bovine	4	S	S	S	R	I	I	S	S	S	S	6255
NAS 41	Bovine	5	R	S	R	R	S	I	S	S	S	S	1063
NAS 89	Bovine	6	S	S	S	R	S	I	S	S	S	I	1820

Refs. [14,15] ST: sequencing type, R: resistant, I: intermediate, S: susceptible, P: penicillin, AMP: ampicillin, AMC: amoxicillin, OX: oxacillin, TE: tetracycline, CFX-K: cephalexin and kanamycin, KF: cephalothin, FOX: cefoxitin, CXM: cefuroxime, EFT: ceftiofur.

**Table 2:** Summary of primers used for MLST of *Staphylococcus simulans*.

Primer Name	Primer Sequence 5′-3′	Amplicon Size	Product
SSIM-PYRR F	GGTAAGCTATTCAATTATAACTG	537 bp	bifunctional pyr operon transcriptional regulator/uracil
SSIM-PYRR R	GGAATGACAGAACGTATTG		phosphoribosyltransferase PyrR
SSIM-GLPK F	GGTAACAAATAGATTCTAACG	985 bp	glycerol kinase GlpK
SSIM-GLPK R	GTCATTGCGACAGTGTTGAATG		
SSIM-GMK F	CCTCCAATATCATTTTTCTATAC	633 bp	guanylate kinase
SSIM-GMK R	GCTTAGAGAGGTCGTAAGGCATG		
SSIM-ADHP F	GATATCTTGAACATCTTCCATTGG	576 bp	alcohol dehydrogenase AdhP
SSIM-ADHP R	GACTATTCTGTTAAAGTTCCAG		
SSIM-XPT F	CATGCGTTCTCTTCACCTTCACC	591 bp	xanthine phosphoribosyltransferase
SSIM-XPT R	CGTGGAAGCGTTGAAAAAGAAGG		
SSIM-PTA F	GCTGCAGTGATGATTGATAAGTTG	996 bp	phosphate acetyltransferase
SSIM-PTA-R	CGAGATTTTCAAGGAGGATATTATG		
SSIM-INFB-R	CCACATGCAAGCGTTAGAACC	896 bp	translation initiation factor IF-2
SSIM-INFB-F	CAGGGTTTGCTGTTGGTTTATC		

Bp; base pairs.

## Data Availability

Data supporting reported results are available on request from the authors of this manuscript.

## Supplementary Materials

The following supporting information can be downloaded at: https://www.mdpi.com/article/10.3390/antibiotics11111609/s1, Figure S1: Gel images showing 1% agarose gel of MLST PCR products: (M) Marker, NEB Fast ladder (N3238S), (Ne) Template Negative control, of each of the seven housekeeping genes (*PyrR, glPk, Gmk, AdhP, Xpt, PTA, InfB)* for the first 5 *S. simulans* isolates from water buffalo. Table S1: Raw data of MALDI-TOF identification of *Staphylococcus simulans* isolated from milk samples of water buffalo and dairy cows, which was performed in duplicate. Data S1: The FASTA format, which is the concatenated (combined) MLST sequencing data. Image S1: The alignment image of concatenated (combined) MLST sequencing data.
