# Supplementary material for: Characterisation of Staphylococci Isolated from Milk Samples of a Water Buffalo Herd
Source: Antibiotics (Basel). 2022 Nov 12;11(11):1609. doi: 10.3390/antibiotics11111609 (PMC9686803; doi:10.3390/antibiotics11111609)
Supplement: Supplementary file 1 [file antibiotics-11-01609-s001.zip › antibiotics-1985840-supplementary.pdf]

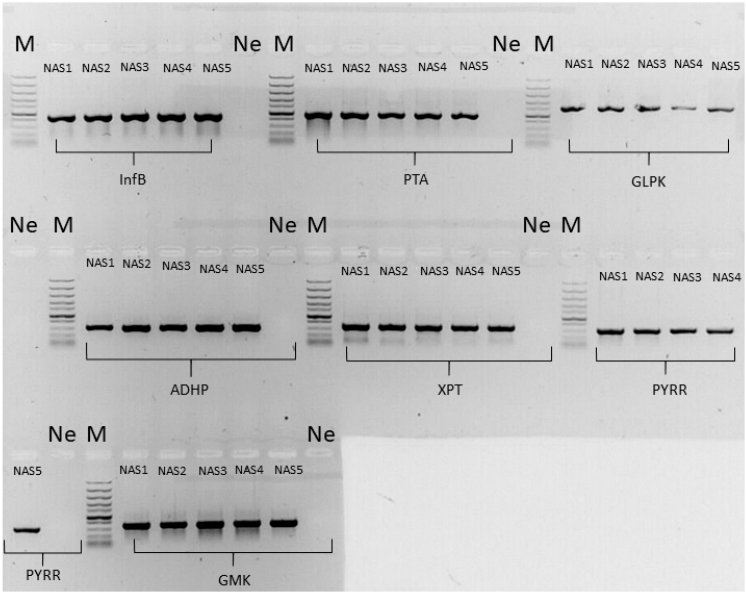

Figure S1: Gel images showing, 1% agarose gel of MLST PCR products: (M) Marker, NEB Fast ladder (N3238S), (Ne) Template Negative control, of each of the seven housekeeping genes (*PyrR, glPk, Gmk, AdhP, Xpt, PTA, InfB)* for the first 5 *S. simulans* isolates from water buffalo.

Table S1: Raw data of MALDI-TOF identification of *Staphylococcus simulans* isolated from milk samples of water buffalo and dairy cows, which were done in duplicate.

Isolate	Origin	MALDI-TOF Raw data			
		Organism (best match)	Score	Organism (second best match)	Score
NAS 1	Buffalo	*S. simulans*	2.192	*S. simulans*	2.166
NAS 2	Buffalo	*S. simulans*	2.208	*S. simulans*	2.107
NAS 3	Buffalo	*S. simulans*	2.205	*S. simulans*	2.117
NAS 4	Buffalo	*S. simulans*	2.115	*S. simulans*	2.06
NAS 5	Buffalo	*S. simulans*	2.223	*S. simulans*	2.18
NAS 6	Buffalo	*S. simulans*	2.325	*S. simulans*	2.152
NAS 7	Buffalo	*S. simulans*	2.18	*S. simulans*	2.149
NAS 8	Buffalo	*S. simulans*	2.25	*S. simulans*	2.136
NAS 10	Buffalo	*S. simulans*	2.375	*S. simulans*	1.663
NAS 11	Buffalo	*S. simulans*	2.265	*S. simulans*	2.199
NAS 12	Buffalo	*S. simulans*	2.169	*S. simulans*	2.079
NAS 13	Buffalo	*S. simulans*	2.092	*S. simulans*	2.04
NAS 14	Buffalo	*S. simulans*	2.267	*S. simulans*	2.129
NAS 15	Buffalo	*S. simulans*	2.215	*S. simulans*	2.168
NAS 17	Buffalo	*S. simulans*	2.154	*S. simulans*	2.103
NAS 214	Bovine	*S. simulans*	2.22	*S. simulans*	2.18
NAS 220	Bovine	*S. simulans*	1.93	*S. simulans*	1.918
NAS 199	Bovine	*S. simulans*	2.12	*S. simulans*	2.06
NAS 209	Bovine	*S. simulans*	2.21	*S. simulans*	2.107
NAS 41	Bovine	*S. simulans*	1.95	*S. simulans*	1.939
NAS 89	Bovine	*S. simulans*	2.04	*S. simulans*	1.97

Meaning of Score Values

Range Description

2.300 ... 3.000 highly probable species identification

2.000 ... 2.299 secure genus identification, probable species identification

1.700 ... 1.999 probable genus identification

0.000 ... 1.699 not reliable identification

Data S1: The FASTA format, which is the concatenated (combined) MLST sequencing data.

>NAS-4
GATTGCTTGAATACGTTCTTTAGCAAGTTCTACTTCATCGTTTACCACAACGTAGTCATA
AAGATTCATCATTTCCACTTCTTTACGTGCTTCTTTCACACGACTTTGAATCTTTTCGTC
TGATTCTGTACCGCGGCCGACTAAACGCTCTCTTAAGTGGTCTAAACTTGGCGGTGCTAG
GAAGATAAATAATGCATCAGGGAATTTCTTGCGTACTTGTTTCGCACCTTCAACTTCGAT
TTCTAAGAAGACATCATAGCCTTCATCCATAGTGTCTTTCACATACTGTACAGGTGTACC
GTAGTAGTTACCTACATATTCTGCATACTCAATAAATTGATCTTGTGCGATAAGTTGCTC
AAACTCTCGTTTTGTCTTGAAAAAATAATCAACGCCATCTTGTTCACCTTCACGCATTTC
ACGTGTCGTCATTGAAATTGAATATTTGTATGATGTTTTCGGATCATCAAATATTTCTTT
GCGGACAGTACCTTTTCCGACTCCAGATGGACCTGATAAGACAATTAGTAAACCTTTTTC
ATTATCCATGCCTTACGTTTAAACCTTGTAAAACAGGTCCGATTGCTTCATATCCGCCTA
AACGTTGCGCAATTTTGTAACCGATGTTACCTGCTTCTAAGCTTGGGAATACGAATACAT
TTGCGTCACCTTTAATTTGTGCATCTGGTGCTTTTTTAGCCGCCACTTCTGGAACGATTG
CTGCGTCGAATTGGAATTCACCATCGATAACAACATCTTTTAAGCCTTCTGATTCAGCTT
TTTCTTGTGCAGCTTTTGTTGCTTCCGCTACTTTTTCAACATCGTCTGATTTTGCTGAAC
CTTTTGTTGAGAAGCTTAATAATGCTACTTTAGGATCCATGCCGAAGCTTTTCGCTGTTT
TTGCACTTTCTACCGCAATTTCAGCTAAGTCTGTTGCTTCTAATGTAGGGTTGATTGCAC
AATCACCGAAAACATATTGTTCTTCGCCTTTAATCATGAAGAACACACCTGATGTTTTCG
CAACACCTGGTTTAGTTTTGATGATTTGTAGTGCTGGACGAACTGTATCGCCTGTTGAGT
GTGCAGCACCGCTGACTAGTCCTTCAGCTTTACCTGCATAAACAAGCATTGTACCGAAGT
AGTTTACATTGTTAAGCATATCTTCAGCTTGTTCTTTTGTCGCTTTGCCTTTACGACGTT
CTACAAATGCGTCTACTAATTCTGGTTTCAAGTCGCGACTAAAACTTCTTCGTCTTTCGC
AGTTGGAATATTTTTACCTACAAAGTCAGCGCGTATTGGTAATTCGCGGTGTCCGCGATC
CACTAATGTTGCTAACCCGATTTTTTGTGGTCGAGAATGTAGTAAGATTGCATCGAGTGA
CGCACGTACAGTTCGACCCGTATAAAGCACATCATCAATGATGATAACGACTTTGTCTTT
AAGGTCTACATCAATGACATAAGACTTCTCAGACATTTTAGGTGTCGGTAACTCTAAATC
ATCTCTGAATTGCGTGATGTCTAATGTTCCTGTAGGAACACTTACGTCTTCAATTTGTTG
AATCTTCATTTGAATACGTTTCGCTAAAAATTCTCCGCGTGTTTTCACACCAAGCAACAC
AAGATTTTCAGTACCTTTGTTATACTCTAGAATTTCATGTGCGATACGTGTTAAAGTCCG
CTGCATGCCTGCATCATCTAATACAATACGCGACTTGGCAAAGCGAAGAAACTTTTAAGC
CAGCTTCTTCTAATCGTTTACGGCCTTGTTGGAAACTTTTTTCCACGACAATACCGATAC
CGACTGTTTCTGCACCTGCTTGTTCAACTAAACGATTCAAACCAAGTGCCGCATCTCCAT
TCGCTAAAAAGTCATCGATAATCAATACACGGTCATTTTCCCCTAAAAATTCTTCAGAGA
TAATGACGCGATTCGTCTTGTTTTTCGTAAACGAATGAATATCTGTCTGATAAAATCCAT
CTGCCAAAGTACTTGGCTTAGCCTTCTTCGCAAAGAGACATGGTACTTCAAAACGATATG
CTGCCATAATAGCTGGCGCAATGCCTGATGCTTCAACAGTTAAGATTTTAGTGATGCCTT
GATCTTTGAACTGTTCATAAAATGTTTCGCCAATGTCATACATTAACACTGCATCAATTT
GATGGTTTAAAAACCCATCTACCTTCAAGATTTTTTCGCCAATGACGACGCCGTCTTCTT
TAACCTTCTACCTACTAGTGAACCTACAACACGAATACCGTCTAATACTAAACGTGGAAT
TGATAAGTCCATTGATTCTGTTGGTAAGCCGACTGCGACTACAGTACCGCCAGCACGTAC
AGATTCAACAGCTGCGTTGAAGGCTTGTTTAGCAACCGCTGTAACAACTGCCGCATGTGC
ACCGCCTACATGTTCTTGAATGTATTTTGCGACATCCACTTCTTTAGGGTTTAAAGTGAC
ATCTGCATCGTATTGTTTCGCAAATTCAAGTTGACTGTCATTGATGTCTACTGCGATAAC
TTTAGCGTTAAATACTTTTTTCGCATATTGTAAAGCTAGGTTTCCAAGTCCACCTAAACC
GAATAAAACAAGCCATTCACCAGCACGTACGCCTGATTCTTTAATTGCTTTGTATGTTGT
GACACCTGCACATGTGATACTGCTGGCAGCTTCTGATGATAAACCATCTGGTTTTTCCAA
CCTTTATATAATTTTTCTCTTTCTTTTTCATCCATTTCAGGCTCGAAAGCTTGATCTAAT
TTCCAACGGTTCGCAATTTCTGCTTTACTATCCCAGAAACCTACCGCAAGTCCTGCAAGA
TAAGCAGCACCTAACGCAGTCGTTTCATTGATTTCAGGACGTTCTACTGTAATATTTTGA
ATATCTGCTTGGAACTGCATTAAGAAGTTGTTCTTAACAGCACCACCATCTACACGGATA
TTCTTCACTTCAATACCTGAGTCTTGAATCATCGCTTCAACCACATCACGTGTTTGGTAA
CAAATAGATTCTAACGTTGCTCTGATAAAGTGTTCTTTCTCAGTCGCACGCGTAATACCA
AATACTGCACCACGCGCATTCGCATCCCAATATGGTGTACCGAGTCCAACAAACGCCGGT
ACCACATAAACACCATCACTTGATTCAATACGGTTCGCATAGTTTTCAGATTCGGGTGCT

GAGTTAATCATACGTAAACCATCTCGCAACCATTGAATAGCTGAACCAGAGACGAAAATA
GAACCTTCTAATGCATAATTCACTTCGCCATCTAAGCCGAATGCGATTGTTGTTAATAAA
CCGTTATCCGATTGAATCGCTTCTTTACCAGTGTTCATTAACATGAATCCACCCGTACCA
TATGTGTTTTTCACTTCACCACGTTCGAAGCATGCTTGTCCGAATAAGGCTGCTTGCTGA
TCCCCTGCGATTCCCGCAATCGGTACTTCTTGTCCAAAGAAATGATAATCTTTTGTTTTC
GCATAAACCTCACTTTTCTTTTGCATGGTTAATCGCTTCAATTGTTTGAGGCATAACACC
GTCGTCTGCTGCAACCACTAAGATTGTGATATCAGTGACTTGCGCACCACGTGCACGCAT
AGTAGTGAAGGCTGCGTGTCCAGGTGTATCTAAGAAAGTGATTTTTTTGCCGTCATTTGT
GATTTGGTAAGCACCGATATGTTGTGTGATACCGCCCGCTTCACCTTGTGTCACATGTGT
ATTTCTGATTGAGTCAAGTAGCGTTGTTTTACCATGGTCAACGTGTCCCATGATTGTTAC
AACTGCTGGACGCTCAATTGCATCCGGATCTTCTTCTTCATCATCGAAGTAGATATCTAA
GTCTTCTTCGTCAATGACTACTTCTTTTTCTACTTCTACACCATAATCATCAGCGATTAA
CTCAATTGTTTCATCGTCTAATGATTGGTTAATGTTTGCCATAATACCAAGTAAGAATAA
TTTCTTAACAATACTTGATGACTCAATATTCATTTTGTCAGCTAATTCGCCAACCGTAAT
ACCTTCTAAATAAGTAATTTTTTCTGGCATTTCTTTTTTCGGTTGTTCTGGTGCTGGTTT
TTGATTATTGTTTTTCTTATGTTTGTTGTTGT
>NAS-89
GATTGCTTGAATGCGTTCTTTCGCAAGTTCTACTTCATCGTTTACCACAACGTAGTCATA
AAGATTCATCATTTCCACTTCTTTACGTGCCTCTTTCACACGACTTTGAATCTTTTCATC
TGTTTCTGTACCGCGGCCGACTAAACGCTCTCTTAAGTGGTCTAAACTTGGCGGTGCTAG
GAAGATAAATAATGCATCAGGGAATTTCTTGCGTACTTGTTTCGCACCTTCAACTTCGAT
TTCTAAGAAGACATCATAACCTTCATCCATAGTGTCTTTCACATACTGTACAGGTGTACC
GTAGTAGTTACCTACATATTCTGCATACTCAATAAATTGATCTTGTGCGATAAGTTGCTC
AAACTCTCGTTTTGTCTTGAAAAAATAATCAACGCCATCTTGTTCACCTTCACGCATTTC
ACGTGTTGTCATTGAAATTGAATATTTGTATGATGTTTTCGGATCATCAAATATTTCTTT
GCGGACAGTACCTTTTCCGACTCCAGATGGACCTGATAAGACAATTAGTAAACCTTTTTC
ATTATCCATGCCTTACGTTTAAACCTTGTAAAACAGGTCCGATTGCTTCATATCCGCCTA
AACGTTGCGCAATTTTGTAACCAATGTTACCTGCTTCTAAACTTGGGAATACGAATACAT
TTGCGTCACCTTTAATTTGTGCATCTGGTGCTTTTTTAGATGCAACTTCTGGAACGATTG
CTGCGTCAAATTGGAATTCACCATCGATGACAACGTCTTTTAAGCCTTCTGATTCAGCTT
TTTCTTGTGCAGCTTTTGTTGCTTCCGCTACTTTTTCAACATCGTCTGATTTCGCTGAAC
CTTTTGTTGAGAAGCTTAATAACGCTACTTTAGGGTCCATGCCGAAGCTTTTCGCTGTTT
TTGCACTTTCTACTGCAATTTCAGCTAAGTCTGTTGCTTCTAATGTAGGGTTGATTGCAC
AGTCGCCGAAAACATATTGTTCTTCGCCTTTAATCATGAAGAACACACCTGATGTTTTCG
CAACACCTGGTTTAGTTTTGATGATTTGTAATGCTGGACGAACTGTATCGCCTGTTGAGT
GTGCAGCACCGCTGACTAGTCCTTCAGCTTTACCTGCATAAACAAGCATCGTACCGAAGT
AGTTTACATTGTTAAGCATATCTTCTGCTTGCTCTTTAGTCGCTTTACCTTTACGACGTT
CTACAAATGCGTCTACTAATTCTGGTTTCAAGTCGCGACTGAAACTTCTTCGTCTTTCGC
AGTTGGAATATTTTTACCTACGAAGTCAGCGCGTATTGGTAATTCGCGGTGTCCGCGATC
CACTAATGTTGCTAACCCGATTTTTTGTGGTCGAGAATGTAGTAAGATTGCATCGAGTGA
CGCACGTACAGTTCGACCCGTATAAAGCACATCATCAATGATGATAACGACTTTGTCTTT
AAGGTCTACATCAATGACATAAGACTTCTCAGACATTTTAGGTGTCGGTAACTCTAAATC
ATCTCTAAATTGCGTGATGTCTAATGTTCCTGTAGGAACACTTACGTCTTCAATTTGTTG
AATCTTCGTTTGAATACGTTTCGCTAAAAATTCTCCGCGTGTTTTCACACCAAGCAACAC
AAGATTTTCAGTACCCTTGTTATACTCTAGAATTTCATGTGCGATACGTGTTAAAGTCCG
CTGCATACCTGCATCATCTAATACAATACGCGACTTGGCAAAGTGAAGAAACCTTCAAAC
CAGCTTCTTCTAATCGTTTACGGCCTTGTTGGAAGCTTTTCTCCACCACGATTCCGATAC
CGACTGTTTCTGCACCTGCTTGTTCAACTAAGCGGTTCAAACCAAGTGCCGCATCTCCAT
TCGCTAAAAAGTCATCGATAATCAACACACGGTCATTTTCACCTAAAAATTCTTCGGAGA
TAATGACACGATTCGTCTTATTTTTCGTAAATGAATGAATATCTGTTTGATAAAATCCAT
CTGCCAAAGTGCTTGGCTTAGCCTTCTTCGCAAAGAGACACGGTACTTCAAATCGATATG
CTGCCATAATGGCTGGTGCTATACCTGATGCTTCAACAGTTAAGATTTTAGTGATGCCTT
GATCTTTAAACTGTTCATAAAATGTTTCACCGATGTCGTACATTAATACTGCATCAATTT
GATGGTTTAAAAACCCATCTACCTTCAAGATTTTTTCGCCGATGACGACGCCGTCTTCTT
TAACCTTCTACCTACTAGTGAACCTACTACGCGGATACCGTCTAATACTAAACGTGGAAT
TGATAAGTCCATAGATTCTGTTGGTAAGCCGACTGCGACTACAGTACCGCCAGCACGTAC
AGATTCAACAGCTGCGTTGAAGGCTTGTTTAGCAACCGCTGTAACAACTGCCGCATGTGC

ACCGTCTACATGTTCTTGAATGTATTTTGCGACATCTACTTCTTTAGGGTTTAAAGTAAC
ATCTGCATCGTATTGTTTCGCAAATTCAAGTTGACTGTCATTAATGTCTACTGCGATAAC
TTTAGCGTTAAATACTTTTTTCGCATATTGTAAAGCTAGGTTTCCGAGTCCGCCTAAACC
GAATAAGACAAGCCATTCACCAGCGCGTACACCTGATTCTTTAATTGCTTTGTAAGTTGT
GACACCTGCGCATGTGATACTGCTGGCAGCTTCTGATGATAAACCATCTGGTTTTTCCAA
CCTTTATATAGTTTTTCTCTTTCTTTTTCATCCATTTCTGGTTCGAACGCTTGATCTAAT
TTCCAACGATTTGTGATTTCTTCTTTACTATCCCAGAAACCTACCGCAAGTCCTGCAAGA
TAAGCAGCACCTAACGCAGTTGTTTCATTAATTTCAGGTCGTTCTACTGTAATATTTTGA
ATATCTGCTTGGAATTGCATTAAGAAGTTATTCTTAACAGCACCGCCATCTACACGAATA
TTCTTCACTTCAATACCTGAATCTTGAATCATCGCTTCAACCACGTCACGTGTTTGGTAA
CAAATAGATTCTAAAGTAGCTCTGATAAAGTGTTCTTTCTCAGTCGCACGTGTAATGCCG
AATACTGCACCGCGCGCATTCGCATCCCAATATGGTGTACCTAGTCCGACAAATGCAGGC
ACAACATAAACACCATCACTTGATTCAATACGGTTCGCGTAGTTTTCAGATTCAGGTGCT
GAGTTAATCATACGTAAACCATCACGTAACCATTGAATCGCTGAACCTGAGACAAAGATA
GAACCTTCTAATGCATAATGTACTTCTCCATCTAAACCGAAAGCAATCGTTGTTAATAAA
CCGTTATCAGATTGAATCGCTTCTTTACCAGTGTTCATTAACATGAAACCACCCGTACCA
TACGTGTTTTTCACTTCCCCGCGTTCAAAGCACGCTTGTCCGAATAATGCTGCTTGCTGG
TCTCCGGCGATACCTGCAATCGGTACTTCTTGTCCAAAGAAATGATAATCTTTTGTTTTC
GCATAAATCTCACTTTTCTTTCGCATGGTTGATTGCTTCAATTGTTTGAGGCATAACACC
ATCATCTGCTGCAACTACTAAGATAGTGATATCAGTAACTTGCGCACCACGCGCACGCAT
TGTAGTGAAGGCTGCGTGTCCAGGTGTATCTAAGAAAGTGATTTTTTTGCCGTCGTTTGT
GATTTGGTAAGCACCGATATGTTGTGTGATACCGCCCGCTTCACCTTGTGTCACATGTGT
ATTTCTGATTGAGTCAAGTAGTGTTGTTTTACCATGGTCAACGTGTCCCATGATTGTAAC
AACTGCTGGGCGCTCAATTGCATCTGGATCTTCTTCTTCATCGTCGAAGTAGATATCTAA
GTCTTCTTCATCAATGACAACTTCTTTTTCTACTTCTACGCCGTAATCATCAGCAATTAA
CTCAATTGTTTCATCGTCTAATGATTGGTTGATGTTAGCCATAATACCAAGTAAGAATAA
TTTCTTAACGATACTTGATGACTCAATATTCATTTTGTCAGCTAATTCGCCAACCGTAAT
ACCTTCTAAGTAAGTAATTTTTTCTGGTATTTCTTTTTTCGGTTGTTCTGGTGCTAGTTT
TTGATTATTGTTTTTCTTATGTTTGTTGTTGT
>NAS-11
GATTGCTTGAATACGTTCTTTAGCAAGTTCTACTTCATCGTTTACCACAACGTAGTCATA
AAGATTCATCATTTCCACTTCTTTACGTGCTTCTTTCACACGACTTTGAATCTTTTCGTC
TGATTCTGTACCGCGGCCGACTAAACGCTCTCTTAAGTGGTCTAAACTTGGCGGTGCTAG
GAAGATAAATAATGCATCAGGGAATTTCTTGCGTACTTGTTTCGCACCTTCAACTTCGAT
TTCTAAGAAGACATCATAGCCTTCATCCATAGTGTCTTTCACATACTGTACAGGTGTACC
GTAGTAGTTACCTACATATTCTGCATACTCAATAAATTGATCTTGTGCGATAAGTTGCTC
AAACTCTCGTTTTGTCTTGAAAAAATAATCAACGCCATCTTGTTCACCTTCACGCATTTC
ACGTGTCGTCATTGAAATTGAATATTTGTATGATGTTTTCGGATCATCAAATATTTCTTT
GCGGACAGTACCTTTTCCGACTCCAGATGGACCTGATAAGACAATTAGTAAACCTTTTTC
ATTATCCATGCCTTACGTTTAAACCTTGTAAAACAGGTCCGATTGCTTCATATCCGCCTA
AACGTTGCGCAATTTTGTAACCGATGTTACCTGCTTCTAAGCTTGGGAATACGAATACAT
TTGCGTCACCTTTAATTTGTGCATCTGGTGCTTTTTTAGCCGCCACTTCTGGAACGATTG
CTGCGTCGAATTGGAATTCACCATCGATAACAACATCTTTTAAGCCTTCTGATTCAGCTT
TTTCTTGTGCAGCTTTTGTTGCTTCCGCTACTTTTTCAACATCGTCTGATTTTGCTGAAC
CTTTTGTTGAGAAGCTTAATAATGCTACTTTAGGATCCATGCCGAAGCTTTTCGCTGTTT
TTGCACTTTCTACCGCAATTTCAGCTAAGTCTGTTGCTTCTAATGTAGGGTTGATTGCAC
AATCACCGAAAACATATTGTTCTTCGCCTTTAATCATGAAGAACACACCTGATGTTTTCG
CAACACCTGGTTTAGTTTTGATGATTTGTAGTGCTGGACGAACTGTATCGCCTGTTGAGT
GTGCAGCACCGCTGACTAGTCCTTCAGCTTTACCTGCATAAACAAGCATTGTACCGAAGT
AGTTTACATTGTTAAGCATATCTTCAGCTTGTTCTTTTGTCGCTTTGCCTTTACGACGTT
CTACAAATGCGTCTACTAATTCTGGTTTCAAGTCGCGACTAAAACTTCTTCGTCTTTCGC
AGTTGGAATATTTTTACCTACAAAGTCAGCGCGTATTGGTAATTCGCGGTGTCCGCGATC
CACTAATGTTGCTAACCCGATTTTTTGTGGTCGAGAATGTAGTAAGATTGCATCGAGTGA
CGCACGTACAGTTCGACCCGTATAAAGCACATCATCAATGATGATAACGACTTTGTCTTT
AAGGTCTACATCAATGACATAAGACTTCTCAGACATTTTAGGTGTCGGTAACTCTAAATC
ATCTCTGAATTGCGTGATGTCTAATGTTCCTGTAGGAACACTTACGTCTTCAATTTGTTG
AATCTTCATTTGAATACGTTTCGCTAAAAATTCTCCGCGTGTTTTCACACCAAGCAACAC

AAGATTTTCAGTACCTTTGTTATACTCTAGAATTTCATGTGCGATACGTGTTAAAGTCCG
CTGCATGCCTGCATCATCTAATACAATACGCGACTTGGCAAAGCGAAGAAACTTTTAAGC
CAGCTTCTTCTAATCGTTTACGGCCTTGTTGGAAACTTTTTTCCACGACAATACCGATAC
CGACTGTTTCTGCACCTGCTTGTTCAACTAAACGATTCAAACCAAGTGCCGCATCTCCAT
TCGCTAAAAAGTCATCGATAATCAATACACGGTCATTTTCCCCTAAAAATTCTTCAGAGA
TAATGACGCGATTCGTCTTGTTTTTCGTAAACGAATGAATATCTGTCTGATAAAATCCAT
CTGCCAAAGTACTTGGCTTAGCCTTCTTCGCAAAGAGACATGGTACTTCAAAACGATATG
CTGCCATAATAGCTGGCGCAATGCCTGATGCTTCAACAGTTAAGATTTTAGTGATGCCTT
GATCTTTGAACTGTTCATAAAATGTTTCGCCAATGTCATACATTAACACTGCATCAATTT
GATGGTTTAAAAACCCATCTACCTTCAAGATTTTTTCGCCAATGACGACGCCGTCTTCTT
TAACCTTCTACCTACTAGTGAACCTACAACACGAATACCGTCTAATACTAAACGTGGAAT
TGATAAGTCCATTGATTCTGTTGGTAAGCCGACTGCGACTACAGTACCGCCAGCACGTAC
AGATTCAACAGCTGCGTTGAAGGCTTGTTTAGCAACCGCTGTAACAACTGCCGCATGTGC
ACCGCCTACATGTTCTTGAATGTATTTTGCGACATCCACTTCTTTAGGGTTTAAAGTGAC
ATCTGCATCGTATTGTTTCGCAAATTCAAGTTGACTGTCATTGATGTCTACTGCGATAAC
TTTAGCGTTAAATACTTTTTTCGCATATTGTAAAGCTAGGTTTCCAAGTCCACCTAAACC
GAATAAAACAAGCCATTCACCAGCACGTACGCCTGATTCTTTAATTGCTTTGTATGTTGT
GACACCTGCACATGTGATACTGCTGGCAGCTTCTGATGATAAACCATCTGGTTTTTCCAA
CCTTTATATAATTTTTCTCTTTCTTTTTCATCCATTTCAGGCTCGAAAGCTTGATCTAAT
TTCCAACGGTTCGCAATTTCTGCTTTACTATCCCAGAAACCTACCGCAAGTCCTGCAAGA
TAAGCAGCACCTAACGCAGTCGTTTCATTGATTTCAGGACGTTCTACTGTAATATTTTGA
ATATCTGCTTGGAACTGCATTAAGAAGTTGTTCTTAACAGCACCACCATCTACACGGATA
TTCTTCACTTCAATACCTGAGTCTTGAATCATCGCTTCAACCACATCACGTGTTTGGTAA
CAAATAGATTCTAACGTTGCTCTGATAAAGTGTTCTTTCTCAGTCGCACGCGTAATACCA
AATACTGCACCACGCGCATTCGCATCCCAATATGGTGTACCGAGTCCAACAAACGCCGGT
ACCACATAAACACCATCACTTGATTCAATACGGTTCGCATAGTTTTCAGATTCGGGTGCT
GAGTTAATCATACGTAAACCATCTCGCAACCATTGAATAGCTGAACCAGAGACGAAAATA
GAACCTTCTAATGCATAATTCACTTCGCCATCTAAGCCGAATGCGATTGTTGTTAATAAA
CCGTTATCCGATTGAATCGCTTCTTTACCAGTGTTCATTAACATGAATCCACCCGTACCA
TATGTGTTTTTCACTTCACCACGTTCGAAGCATGCTTGTCCGAATAAGGCTGCTTGCTGA
TCCCCTGCGATTCCCGCAATCGGTACTTCTTGTCCAAAGAAATGATAATCTTTTGTTTTC
GCATAAACCTCACTTTTCTTTTGCATGGTTAATCGCTTCAATTGTTTGAGGCATAACACC
GTCGTCTGCTGCAACCACTAAGATTGTGATATCAGTGACTTGCGCACCACGTGCACGCAT
AGTAGTGAAGGCTGCGTGTCCAGGTGTATCTAAGAAAGTGATTTTTTTGCCGTCATTTGT
GATTTGGTAAGCACCGATATGTTGTGTGATACCGCCCGCTTCACCTTGTGTCACATGTGT
ATTTCTGATTGAGTCAAGTAGCGTTGTTTTACCATGGTCAACGTGTCCCATGATTGTTAC
AACTGCTGGACGCTCAATTGCATCCGGATCTTCTTCTTCATCATCGAAGTAGATATCTAA
GTCTTCTTCGTCAATGACTACTTCTTTTTCTACTTCTACACCATAATCATCAGCGATTAA
CTCAATTGTTTCATCGTCTAATGATTGGTTAATGTTTGCCATAATACCAAGTAAGAATAA
TTTCTTAACAATACTTGATGACTCAATATTCATTTTGTCAGCTAATTCGCCAACCGTAAT
ACCTTCTAAATAAGTAATTTTTTCTGGCATTTCTTTTTTCGGTTGTTCTGGTGCTGGTTT
TTGATTATTGTTTTTCTTATGTTTGTTGTTGT
>NAS-220
GATTGCTTGAATACGTTCTTTAGCAAGTTCTACTTCATCGTTTACCACAACGTAGTCATA
AAGATTCATCATTTCCACTTCTTTACGTGCTTCTTTCACACGACTTTGAATCTTTTCGTC
TGATTCTGTACCGCGGCCGACTAAACGCTCTCTTAAGTGGTCTAAACTTGGCGGTGCTAG
GAAGATAAATAATGCATCAGGGAATTTCTTGCGTACTTGTTTCGCACCTTCAACTTCGAT
TTCTAAGAAGACATCATAGCCTTCATCCATAGTGTCTTTCACATACTGTACAGGTGTACC
GTAGTAGTTACCTACATATTCTGCATACTCAATAAATTGATCTTGTGCGATAAGTTGCTC
AAACTCTCGTTTTGTCTTGAAAAAATAATCAACGCCATCTTGTTCACCTTCACGCATTTC
ACGTGTCGTCATTGAAATTGAATATTTGTATGATGTTTTCGGATCATCAAATATTTCTTT
GCGGACAGTACCTTTTCCGACTCCAGATGGACCTGATAAGACAATTAGTAAACCTTTTTC
ATTATCCATGCCTTACGTTTAAACCTTGTAAAACAGGTCCGATTGCTTCATATCCGCCTA
AACGTTGCGCAATTTTGTAACCGATGTTACCTGCTTCTAAGCTTGGGAATACGAATACAT
TTGCGTCACCTTTAATTTGTGCATCTGGTGCTTTTTTAGCCGCCACTTCTGGAACGATTG
CTGCGTCGAATTGGAATTCACCATCGATAACAACATCTTTTAAGCCTTCTGATTCAGCTT
TTTCTTGTGCAGCTTTTGTTGCTTCCGCTACTTTTTCAACATCGTCTGATTTTGCTGAAC

CTTTTGTTGAGAAGCTTAATAATGCTACTTTAGGATCCATGCCGAAGCTTTTCGCTGTTT
TTGCACTTTCTACCGCAATTTCAGCTAAGTCTGTTGCTTCTAATGTAGGGTTGATTGCAC
AATCACCGAAAACATATTGTTCTTCGCCTTTAATCATGAAGAACACACCTGATGTTTTCG
CAACACCTGGTTTAGTTTTGATGATTTGTAGTGCTGGACGAACTGTATCGCCTGTTGAGT
GTGCAGCACCGCTGACTAGTCCTTCAGCTTTACCTGCATAAACAAGCATTGTACCGAAGT
AGTTTACATTGTTAAGCATATCTTCAGCTTGTTCTTTTGTCGCTTTACCTTTACGACGTT
CTACAAATGCGTCTACTAATTCTGGTTTCAAGTCGCGACTAAAACTTCTTCGTCTTTCGC
AGTTGGAATATTTTTACCTACGAAGTCAGCGCGTATTGGTAATTCGCGGTGTCCGCGATC
CACTAATGTTGCTAACCCGATTTTTTGTGGTCGAGAATGTAGTAAGATTGCATCGAGTGA
CGCACGTACAGTTCGACCTGTATAAAGCACATCATCAATGATGATAACGACTTTGTCTTT
AAGGTCTACATCAATGACATAAGACTTCTCAGACATTTTAGGTGTCGGTAATTCTAAGTC
ATCTCTGAATTGCGTGATGTCTAATGTTCCTGTAGGAACACTTACGTCTTCAATTTGTTG
AATCTTCGTTTGAATACGTTTCGCTAAAAATTCTCCGCGTGTTTTCACACCAAGCAACAC
AAGATTTTCAGTACCTTTGTTATACTCTAAAATTTCATGTGCGATACGTGTTAAAGTCCG
CTGCATGCCTGCATCATCTAATACAATACGCGACTTGGCAAAGTGAAGAAACTTTTAAGC
CAGCTTCTTCTAATCGTTTACGGCCTTGTTGGAAACTTTTCTCTACTACAATACCGATAC
CGACTGTTTCTGCACCTGCTTGTTCAACTAAACGGTTCAAACCAAGTGCCGCATCTCCAT
TCGCTAAAAAGTCATCGATAATCAATACACGGTCATTTTCCCCTAAAAATTCTTCAGAGA
TAATGACGCGATTCGTCTTGTTTTTCGTAAACGAATGAATATCTGTCTGATAAAATCCAT
CTGCCAAAGTACTTGGCTTAGCCTTCTTCGCAAAAAGACATGGTACTTCAAAACGATATG
CTGCCATAATAGCTGGCGCAATACCTGATGCTTCAACAGTTAAGATTTTAGTGATGCCTT
GATCTTTGAACTGTTCATAAAATGTTTCGCCAATGTCATACATTAACACTGCATCAATTT
GATGGTTTAAAAACCCATCTACCTTCAAGATTTTTTCGCCAATGACGACGCCGTCTTCTT
TAACCTTCTACCTACTAGTGACCTACAACACGAATACCGTCTAATACCAAACGTGGAATT
GATAAGTCCATAGATTCTGTTGGCAAGCCGACTGCGACTACAGTACCGCCAGCACGTACA
GATTCAACAGCTGCGTTGAAGGCTTGTTTAGCAACCGCTGTAACAACTGCCGCATGTGCA
CCGTCTACATGTTCTTGAATGTATTTTGCGACATCCACTTCTTTAGGGTTTAAAGTGACA
TCTGCATCGTATTGTTTCGCAAATTCAAGTTGACTGTCATTGATGTCTACTGCGATAACT
TTAGCGTTAAATACTTTTTTCGCATATTGTAAAGCTAAGTTTCCAAGTCCACCTAAACCG
AATAAGACAAGCCATTCACCAGCACGTACGCCTGATTCTTTAATCGCTTTGTATGTTGTG
ACACCTGCACATGTGATACTGCTGGCAGCTTCTGATGATAAACCATCTGGTTTTTCCAAC
CTTTATATAATTTTTCTCTTTCTTTTTCATCCATTTCAGGCTCGAAAGCTTGATCTAATT
TCCAACGGTTCGCAATTTCTGCTTTACTATCCCAGAAACCTACCGCAAGTCCTGCAAGAT
AAGCAGCGCCTAACGCAGTCGTTTCATTGATTTCAGGACGTTCTACTGTAATATTTTGAA
TGTCTGCTTGGAACTGCATTAAGAAGTTGTTCTTAACAGCACCACCATCTACACGGATAT
TCTTCACTTCAATACCTGAGTCTTGAATCATCGCTTCAACCACATCACGTGTTTGGTAAC
AAATAGATTCTAACGTTGCTCTGATAAAGTGTTCTTTCTCAGTCGCACGCGTAATACCAA
ATACTGCACCACGCGCATTTGCATCCCAATATGGTGTACCGAGTCCAACAAACGCCGGTA
CCACATAAACACCATCACTTGATTCAATACGGTTCGCATAGTTTTCAGATTCAGGTGCTG
AGTTAATCATACGTAAACCATCTCGCAACCATTGAATAGCTGAACCAGAGACGAAAATAG
AACCTTCTAATGCATAATTCACTTCGCCATCTAAGCCGAATGCGATTGTTGTTAATAAAC
CGTTATCAGATTGAATCGCTTCTTTACCAGTGTTCATTAACATGAATCCACCTGTACCGT
ACGTGTTTTTCACTTCACCACGTTCGAAGCATGCTTGTCCGAATAAGGCCGCTTGCTGAT
CCCCTGCGATTCCCGCAATCGGTACTTCTTGTCCAAAGAAATGATAATCTTTTGTTTTCG
CATAAATCTCACTTTTCTTTCGCATGGTTAATCGCTTCAATTGTTTGAGGCATAACACCG
TCGTCTGCTGCAACCACTAAGATTGTGATATCAGTGACTTGCGCACCACGTGCACGCATA
GTAGTGAAGGCTGCGTGTCCAGGTGTATCTAAGAAAGTGATTTTTTTGCCGTCATTTGTG
ATTTGGTAAGCACCGATATGTTGTGTGATACCGCCCGCTTCACCTTGTGTCACATGTGTA
TTTCTGATTGAGTCAAGTAGCGTTGTTTTACCATGGTCAACGTGTCCCATGATTGTTACA
ACTGCTGGACGCTCAATTGCATCCGGATCTTCTTCTTCATCATCGAAGTAGATATCTAAG
TCTTCTTCGTCAATGACTACTTCTTTTTCTACTTCTACACCATAATCATCAGCGATTAAC
TCAATTGTTTCATCGTCTAATGATTGGTTAATGTTTGCCATAATACCAAGTAAGAATAAT
TTCTTAACGATACTTGATGACTCAATATTCATTTTGTCAGCTAATTCGCCAACCGTAATA
CCTTCTAAATAAGTAATTTTTTCTGGCATTTCTTTTTTCGGTTGTTCTGGTGCTGGTTTT
TGATTATTGTTTTTCTTATGTTTGTTGTTGT
>NAS-6
GATTGCTTGAATACGTTCTTTAGCAAGTTCTACTTCATCGTTTACCACAACGTAGTCATA

AAGATTCATCATTTCCACTTCTTTACGTGCTTCTTTCACACGACTTTGAATCTTTTCGTC
TGATTCTGTACCGCGGCCGACTAAACGCTCTCTTAAGTGGTCTAAACTTGGCGGTGCTAG
GAAGATAAATAATGCATCAGGGAATTTCTTGCGTACTTGTTTCGCACCTTCAACTTCGAT
TTCTAAGAAGACATCATAGCCTTCATCCATAGTGTCTTTCACATACTGTACAGGTGTACC
GTAGTAGTTACCTACATATTCTGCATACTCAATAAATTGATCTTGTGCGATAAGTTGCTC
AAACTCTCGTTTTGTCTTGAAAAAATAATCAACGCCATCTTGTTCACCTTCACGCATTTC
ACGTGTCGTCATTGAAATTGAATATTTGTATGATGTTTTCGGATCATCAAATATTTCTTT
GCGGACAGTACCTTTTCCGACTCCAGATGGACCTGATAAGACAATTAGTAAACCTTTTTC
ATTATCCATGCCTTACGTTTAAACCTTGTAAAACAGGTCCGATTGCTTCATATCCGCCTA
AACGTTGCGCAATTTTGTAACCGATGTTACCTGCTTCTAAGCTTGGGAATACGAATACAT
TTGCGTCACCTTTAATTTGTGCATCTGGTGCTTTTTTAGCCGCCACTTCTGGAACGATTG
CTGCGTCGAATTGGAATTCACCATCGATAACAACATCTTTTAAGCCTTCTGATTCAGCTT
TTTCTTGTGCAGCTTTTGTTGCTTCCGCTACTTTTTCAACATCGTCTGATTTTGCTGAAC
CTTTTGTTGAGAAGCTTAATAATGCTACTTTAGGATCCATGCCGAAGCTTTTCGCTGTTT
TTGCACTTTCTACCGCAATTTCAGCTAAGTCTGTTGCTTCTAATGTAGGGTTGATTGCAC
AATCACCGAAAACATATTGTTCTTCGCCTTTAATCATGAAGAACACACCTGATGTTTTCG
CAACACCTGGTTTAGTTTTGATGATTTGTAGTGCTGGACGAACTGTATCGCCTGTTGAGT
GTGCAGCACCGCTGACTAGTCCTTCAGCTTTACCTGCATAAACAAGCATTGTACCGAAGT
AGTTTACATTGTTAAGCATATCTTCAGCTTGTTCTTTTGTCGCTTTGCCTTTACGACGTT
CTACAAATGCGTCTACTAATTCTGGTTTCAAGTCGCGACTAAAACTTCTTCGTCTTTCGC
AGTTGGAATATTTTTACCTACAAAGTCAGCGCGTATTGGTAATTCGCGGTGTCCGCGATC
CACTAATGTTGCTAACCCGATTTTTTGTGGTCGAGAATGTAGTAAGATTGCATCGAGTGA
CGCACGTACAGTTCGACCCGTATAAAGCACATCATCAATGATGATAACGACTTTGTCTTT
AAGGTCTACATCAATGACATAAGACTTCTCAGACATTTTAGGTGTCGGTAACTCTAAATC
ATCTCTGAATTGCGTGATGTCTAATGTTCCTGTAGGAACACTTACGTCTTCAATTTGTTG
AATCTTCATTTGAATACGTTTCGCTAAAAATTCTCCGCGTGTTTTCACACCAAGCAACAC
AAGATTTTCAGTACCTTTGTTATACTCTAGAATTTCATGTGCGATACGTGTTAAAGTCCG
CTGCATGCCTGCATCATCTAATACAATACGCGACTTGGCAAAGCGAAGAAACTTTTAAGC
CAGCTTCTTCTAATCGTTTACGGCCTTGTTGGAAACTTTTTTCCACGACAATACCGATAC
CGACTGTTTCTGCACCTGCTTGTTCAACTAAACGATTCAAACCAAGTGCCGCATCTCCAT
TCGCTAAAAAGTCATCGATAATCAATACACGGTCATTTTCCCCTAAAAATTCTTCAGAGA
TAATGACGCGATTCGTCTTGTTTTTCGTAAACGAATGAATATCTGTCTGATAAAATCCAT
CTGCCAAAGTACTTGGCTTAGCCTTCTTCGCAAAGAGACATGGTACTTCAAAACGATATG
CTGCCATAATAGCTGGCGCAATGCCTGATGCTTCAACAGTTAAGATTTTAGTGATGCCTT
GATCTTTGAACTGTTCATAAAATGTTTCGCCAATGTCATACATTAACACTGCATCAATTT
GATGGTTTAAAAACCCATCTACCTTCAAGATTTTTTCGCCAATGACGACGCCGTCTTCTT
TAACCTTCTACCTACTAGTGAACCTACAACACGAATACCGTCTAATACTAAACGTGGAAT
TGATAAGTCCATTGATTCTGTTGGTAAGCCGACTGCGACTACAGTACCGCCAGCACGTAC
AGATTCAACAGCTGCGTTGAAGGCTTGTTTAGCAACCGCTGTAACAACTGCCGCATGTGC
ACCGCCTACATGTTCTTGAATGTATTTTGCGACATCCACTTCTTTAGGGTTTAAAGTGAC
ATCTGCATCGTATTGTTTCGCAAATTCAAGTTGACTGTCATTGATGTCTACTGCGATAAC
TTTAGCGTTAAATACTTTTTTCGCATATTGTAAAGCTAGGTTTCCAAGTCCACCTAAACC
GAATAAAACAAGCCATTCACCAGCACGTACGCCTGATTCTTTAATTGCTTTGTATGTTGT
GACACCTGCACATGTGATACTGCTGGCAGCTTCTGATGATAAACCATCTGGTTTTTCCAA
CCTTTATATAATTTTTCTCTTTCTTTTTCATCCATTTCAGGCTCGAAAGCTTGATCTAAT
TTCCAACGGTTCGCAATTTCTGCTTTACTATCCCAGAAACCTACCGCAAGTCCTGCAAGA
TAAGCAGCACCTAACGCAGTCGTTTCATTGATTTCAGGACGTTCTACTGTAATATTTTGA
ATATCTGCTTGGAACTGCATTAAGAAGTTGTTCTTAACAGCACCACCATCTACACGGATA
TTCTTCACTTCAATACCTGAGTCTTGAATCATCGCTTCAACCACATCACGTGTTTGGTAA
CAAATAGATTCTAACGTTGCTCTGATAAAGTGTTCTTTCTCAGTCGCACGCGTAATACCA
AATACTGCACCACGCGCATTCGCATCCCAATATGGTGTACCGAGTCCAACAAACGCCGGT
ACCACATAAACACCATCACTTGATTCAATACGGTTCGCATAGTTTTCAGATTCGGGTGCT
GAGTTAATCATACGTAAACCATCTCGCAACCATTGAATAGCTGAACCAGAGACGAAAATA
GAACCTTCTAATGCATAATTCACTTCGCCATCTAAGCCGAATGCGATTGTTGTTAATAAA
CCGTTATCCGATTGAATCGCTTCTTTACCAGTGTTCATTAACATGAATCCACCCGTACCA
TATGTGTTTTTCACTTCACCACGTTCGAAGCATGCTTGTCCGAATAAGGCTGCTTGCTGA
TCCCCTGCGATTCCCGCAATCGGTACTTCTTGTCCAAAGAAATGATAATCTTTTGTTTTC

GCATAAACCTCACTTTTCTTTTGCATGGTTAATCGCTTCAATTGTTTGAGGCATAACACC
GTCGTCTGCTGCAACCACTAAGATTGTGATATCAGTGACTTGCGCACCACGTGCACGCAT
AGTAGTGAAGGCTGCGTGTCCAGGTGTATCTAAGAAAGTGATTTTTTTGCCGTCATTTGT
GATTTGGTAAGCACCGATATGTTGTGTGATACCGCCCGCTTCACCTTGTGTCACATGTGT
ATTTCTGATTGAGTCAAGTAGCGTTGTTTTACCATGGTCAACGTGTCCCATGATTGTTAC
AACTGCTGGACGCTCAATTGCATCCGGATCTTCTTCTTCATCATCGAAGTAGATATCTAA
GTCTTCTTCGTCAATGACTACTTCTTTTTCTACTTCTACACCATAATCATCAGCGATTAA
CTCAATTGTTTCATCGTCTAATGATTGGTTAATGTTTGCCATAATACCAAGTAAGAATAA
TTTCTTAACAATACTTGATGACTCAATATTCATTTTGTCAGCTAATTCGCCAACCGTAAT
ACCTTCTAAATAAGTAATTTTTTCTGGCATTTCTTTTTTCGGTTGTTCTGGTGCTGGTTT
TTGATTATTGTTTTTCTTATGTTTGTTGTTGT
>NAS-14
GATTGCTTGAATACGTTCTTTAGCAAGTTCTACTTCATCGTTTACCACAACGTAGTCATA
AAGATTCATCATTTCCACTTCTTTACGTGCTTCTTTCACACGACTTTGAATCTTTTCGTC
TGATTCTGTACCGCGGCCGACTAAACGCTCTCTTAAGTGGTCTAAACTTGGCGGTGCTAG
GAAGATAAATAATGCATCAGGGAATTTCTTGCGTACTTGTTTCGCACCTTCAACTTCGAT
TTCTAAGAAGACATCATAGCCTTCATCCATAGTGTCTTTCACATACTGTACAGGTGTACC
GTAGTAGTTACCTACATATTCTGCATACTCAATAAATTGATCTTGTGCGATAAGTTGCTC
AAACTCTCGTTTTGTCTTGAAAAAATAATCAACGCCATCTTGTTCACCTTCACGCATTTC
ACGTGTCGTCATTGAAATTGAATATTTGTATGATGTTTTCGGATCATCAAATATTTCTTT
GCGGACAGTACCTTTTCCGACTCCAGATGGACCTGATAAGACAATTAGTAAACCTTTTTC
ATTATCCATGCCTTACGTTTAAACCTTGTAAAACAGGTCCGATTGCTTCATATCCGCCTA
AACGTTGCGCAATTTTGTAACCGATGTTACCTGCTTCTAAGCTTGGGAATACGAATACAT
TTGCGTCACCTTTAATTTGTGCATCTGGTGCTTTTTTAGCCGCCACTTCTGGAACGATTG
CTGCGTCGAATTGGAATTCACCATCGATAACAACATCTTTTAAGCCTTCTGATTCAGCTT
TTTCTTGTGCAGCTTTTGTTGCTTCCGCTACTTTTTCAACATCGTCTGATTTTGCTGAAC
CTTTTGTTGAGAAGCTTAATAATGCTACTTTAGGATCCATGCCGAAGCTTTTCGCTGTTT
TTGCACTTTCTACCGCAATTTCAGCTAAGTCTGTTGCTTCTAATGTAGGGTTGATTGCAC
AATCACCGAAAACATATTGTTCTTCGCCTTTAATCATGAAGAACACACCTGATGTTTTCG
CAACACCTGGTTTAGTTTTGATGATTTGTAGTGCTGGACGAACTGTATCGCCTGTTGAGT
GTGCAGCACCGCTGACTAGTCCTTCAGCTTTACCTGCATAAACAAGCATTGTACCGAAGT
AGTTTACATTGTTAAGCATATCTTCAGCTTGTTCTTTTGTCGCTTTGCCTTTACGACGTT
CTACAAATGCGTCTACTAATTCTGGTTTCAAGTCGCGACTAAAACTTCTTCGTCTTTCGC
AGTTGGAATATTTTTACCTACAAAGTCAGCGCGTATTGGTAATTCGCGGTGTCCGCGATC
CACTAATGTTGCTAACCCGATTTTTTGTGGTCGAGAATGTAGTAAGATTGCATCGAGTGA
CGCACGTACAGTTCGACCCGTATAAAGCACATCATCAATGATGATAACGACTTTGTCTTT
AAGGTCTACATCAATGACATAAGACTTCTCAGACATTTTAGGTGTCGGTAACTCTAAATC
ATCTCTGAATTGCGTGATGTCTAATGTTCCTGTAGGAACACTTACGTCTTCAATTTGTTG
AATCTTCATTTGAATACGTTTCGCTAAAAATTCTCCGCGTGTTTTCACACCAAGCAACAC
AAGATTTTCAGTACCTTTGTTATACTCTAGAATTTCATGTGCGATACGTGTTAAAGTCCG
CTGCATGCCTGCATCATCTAATACAATACGCGACTTGGCAAAGCGAAGAAACTTTTAAGC
CAGCTTCTTCTAATCGTTTACGGCCTTGTTGGAAACTTTTTTCCACGACAATACCGATAC
CGACTGTTTCTGCACCTGCTTGTTCAACTAAACGATTCAAACCAAGTGCCGCATCTCCAT
TCGCTAAAAAGTCATCGATAATCAATACACGGTCATTTTCCCCTAAAAATTCTTCAGAGA
TAATGACGCGATTCGTCTTGTTTTTCGTAAACGAATGAATATCTGTCTGATAAAATCCAT
CTGCCAAAGTACTTGGCTTAGCCTTCTTCGCAAAGAGACATGGTACTTCAAAACGATATG
CTGCCATAATAGCTGGCGCAATGCCTGATGCTTCAACAGTTAAGATTTTAGTGATGCCTT
GATCTTTGAACTGTTCATAAAATGTTTCGCCAATGTCATACATTAACACTGCATCAATTT
GATGGTTTAAAAACCCATCTACCTTCAAGATTTTTTCGCCAATGACGACGCCGTCTTCTT
TAACCTTCTACCTACTAGTGAACCTACAACACGAATACCGTCTAATACTAAACGTGGAAT
TGATAAGTCCATTGATTCTGTTGGTAAGCCGACTGCGACTACAGTACCGCCAGCACGTAC
AGATTCAACAGCTGCGTTGAAGGCTTGTTTAGCAACCGCTGTAACAACTGCCGCATGTGC
ACCGCCTACATGTTCTTGAATGTATTTTGCGACATCCACTTCTTTAGGGTTTAAAGTGAC
ATCTGCATCGTATTGTTTCGCAAATTCAAGTTGACTGTCATTGATGTCTACTGCGATAAC
TTTAGCGTTAAATACTTTTTTCGCATATTGTAAAGCTAGGTTTCCAAGTCCACCTAAACC
GAATAAAACAAGCCATTCACCAGCACGTACGCCTGATTCTTTAATTGCTTTGTATGTTGT
GACACCTGCACATGTGATACTGCTGGCAGCTTCTGATGATAAACCATCTGGTTTTTCCAA

CCTTTATATAATTTTTCTCTTTCTTTTTCATCCATTTCAGGCTCGAAAGCTTGATCTAAT
TTCCAACGGTTCGCAATTTCTGCTTTACTATCCCAGAAACCTACCGCAAGTCCTGCAAGA
TAAGCAGCACCTAACGCAGTCGTTTCATTGATTTCAGGACGTTCTACTGTAATATTTTGA
ATATCTGCTTGGAACTGCATTAAGAAGTTGTTCTTAACAGCACCACCATCTACACGGATA
TTCTTCACTTCAATACCTGAGTCTTGAATCATCGCTTCAACCACATCACGTGTTTGGTAA
CAAATAGATTCTAACGTTGCTCTGATAAAGTGTTCTTTCTCAGTCGCACGCGTAATACCA
AATACTGCACCACGCGCATTCGCATCCCAATATGGTGTACCGAGTCCAACAAACGCCGGT
ACCACATAAACACCATCACTTGATTCAATACGGTTCGCATAGTTTTCAGATTCGGGTGCT
GAGTTAATCATACGTAAACCATCTCGCAACCATTGAATAGCTGAACCAGAGACGAAAATA
GAACCTTCTAATGCATAATTCACTTCGCCATCTAAGCCGAATGCGATTGTTGTTAATAAA
CCGTTATCCGATTGAATCGCTTCTTTACCAGTGTTCATTAACATGAATCCACCCGTACCA
TATGTGTTTTTCACTTCACCACGTTCGAAGCATGCTTGTCCGAATAAGGCTGCTTGCTGA
TCCCCTGCGATTCCCGCAATCGGTACTTCTTGTCCAAAGAAATGATAATCTTTTGTTTTC
GCATAAACCTCACTTTTCTTTTGCATGGTTAATCGCTTCAATTGTTTGAGGCATAACACC
GTCGTCTGCTGCAACCACTAAGATTGTGATATCAGTGACTTGCGCACCACGTGCACGCAT
AGTAGTGAAGGCTGCGTGTCCAGGTGTATCTAAGAAAGTGATTTTTTTGCCGTCATTTGT
GATTTGGTAAGCACCGATATGTTGTGTGATACCGCCCGCTTCACCTTGTGTCACATGTGT
ATTTCTGATTGAGTCAAGTAGCGTTGTTTTACCATGGTCAACGTGTCCCATGATTGTTAC
AACTGCTGGACGCTCAATTGCATCCGGATCTTCTTCTTCATCATCGAAGTAGATATCTAA
GTCTTCTTCGTCAATGACTACTTCTTTTTCTACTTCTACACCATAATCATCAGCGATTAA
CTCAATTGTTTCATCGTCTAATGATTGGTTAATGTTTGCCATAATACCAAGTAAGAATAA
TTTCTTAACAATACTTGATGACTCAATATTCATTTTGTCAGCTAATTCGCCAACCGTAAT
ACCTTCTAAATAAGTAATTTTTTCTGGCATTTCTTTTTTCGGTTGTTCTGGTGCTGGTTT
TTGATTATTGTTTTTCTTATGTTTGTTGTTGT
>NAS-8
GATTGCTTGAATACGTTCTTTAGCAAGTTCTACTTCATCGTTTACCACAACGTAGTCATA
AAGATTCATCATTTCCACTTCTTTACGTGCTTCTTTCACACGACTTTGAATCTTTTCGTC
TGATTCTGTACCGCGGCCGACTAAACGCTCTCTTAAGTGGTCTAAACTTGGCGGTGCTAG
GAAGATAAATAATGCATCAGGGAATTTCTTGCGTACTTGTTTCGCACCTTCAACTTCGAT
TTCTAAGAAGACATCATAGCCTTCATCCATAGTGTCTTTCACATACTGTACAGGTGTACC
GTAGTAGTTACCTACATATTCTGCATACTCAATAAATTGATCTTGTGCGATAAGTTGCTC
AAACTCTCGTTTTGTCTTGAAAAAATAATCAACGCCATCTTGTTCACCTTCACGCATTTC
ACGTGTCGTCATTGAAATTGAATATTTGTATGATGTTTTCGGATCATCAAATATTTCTTT
GCGGACAGTACCTTTTCCGACTCCAGATGGACCTGATAAGACAATTAGTAAACCTTTTTC
ATTATCCATGCCTTACGTTTAAACCTTGTAAAACAGGTCCGATTGCTTCATATCCGCCTA
AACGTTGCGCAATTTTGTAACCGATGTTACCTGCTTCTAAGCTTGGGAATACGAATACAT
TTGCGTCACCTTTAATTTGTGCATCTGGTGCTTTTTTAGCCGCCACTTCTGGAACGATTG
CTGCGTCGAATTGGAATTCACCATCGATAACAACATCTTTTAAGCCTTCTGATTCAGCTT
TTTCTTGTGCAGCTTTTGTTGCTTCCGCTACTTTTTCAACATCGTCTGATTTTGCTGAAC
CTTTTGTTGAGAAGCTTAATAATGCTACTTTAGGATCCATGCCGAAGCTTTTCGCTGTTT
TTGCACTTTCTACCGCAATTTCAGCTAAGTCTGTTGCTTCTAATGTAGGGTTGATTGCAC
AATCACCGAAAACATATTGTTCTTCGCCTTTAATCATGAAGAACACACCTGATGTTTTCG
CAACACCTGGTTTAGTTTTGATGATTTGTAGTGCTGGACGAACTGTATCGCCTGTTGAGT
GTGCAGCACCGCTGACTAGTCCTTCAGCTTTACCTGCATAAACAAGCATTGTACCGAAGT
AGTTTACATTGTTAAGCATATCTTCAGCTTGTTCTTTTGTCGCTTTGCCTTTACGACGTT
CTACAAATGCGTCTACTAATTCTGGTTTCAAGTCGCGACTAAAACTTCTTCGTCTTTCGC
AGTTGGAATATTTTTACCTACAAAGTCAGCGCGTATTGGTAATTCGCGGTGTCCGCGATC
CACTAATGTTGCTAACCCGATTTTTTGTGGTCGAGAATGTAGTAAGATTGCATCGAGTGA
CGCACGTACAGTTCGACCCGTATAAAGCACATCATCAATGATGATAACGACTTTGTCTTT
AAGGTCTACATCAATGACATAAGACTTCTCAGACATTTTAGGTGTCGGTAACTCTAAATC
ATCTCTGAATTGCGTGATGTCTAATGTTCCTGTAGGAACACTTACGTCTTCAATTTGTTG
AATCTTCATTTGAATACGTTTCGCTAAAAATTCTCCGCGTGTTTTCACACCAAGCAACAC
AAGATTTTCAGTACCTTTGTTATACTCTAGAATTTCATGTGCGATACGTGTTAAAGTCCG
CTGCATGCCTGCATCATCTAATACAATACGCGACTTGGCAAAGCGAAGAAACTTTTAAGC
CAGCTTCTTCTAATCGTTTACGGCCTTGTTGGAAACTTTTTTCCACGACAATACCGATAC
CGACTGTTTCTGCACCTGCTTGTTCAACTAAACGATTCAAACCAAGTGCCGCATCTCCAT
TCGCTAAAAAGTCATCGATAATCAATACACGGTCATTTTCCCCTAAAAATTCTTCAGAGA

TAATGACGCGATTCGTCTTGTTTTTCGTAAACGAATGAATATCTGTCTGATAAAATCCAT
CTGCCAAAGTACTTGGCTTAGCCTTCTTCGCAAAGAGACATGGTACTTCAAAACGATATG
CTGCCATAATAGCTGGCGCAATGCCTGATGCTTCAACAGTTAAGATTTTAGTGATGCCTT
GATCTTTGAACTGTTCATAAAATGTTTCGCCAATGTCATACATTAACACTGCATCAATTT
GATGGTTTAAAAACCCATCTACCTTCAAGATTTTTTCGCCAATGACGACGCCGTCTTCTT
TAACCTTCTACCTACTAGTGAACCTACAACACGAATACCGTCTAATACTAAACGTGGAAT
TGATAAGTCCATTGATTCTGTTGGTAAGCCGACTGCGACTACAGTACCGCCAGCACGTAC
AGATTCAACAGCTGCGTTGAAGGCTTGTTTAGCAACCGCTGTAACAACTGCCGCATGTGC
ACCGCCTACATGTTCTTGAATGTATTTTGCGACATCCACTTCTTTAGGGTTTAAAGTGAC
ATCTGCATCGTATTGTTTCGCAAATTCAAGTTGACTGTCATTGATGTCTACTGCGATAAC
TTTAGCGTTAAATACTTTTTTCGCATATTGTAAAGCTAGGTTTCCAAGTCCACCTAAACC
GAATAAAACAAGCCATTCACCAGCACGTACGCCTGATTCTTTAATTGCTTTGTATGTTGT
GACACCTGCACATGTGATACTGCTGGCAGCTTCTGATGATAAACCATCTGGTTTTTCCAA
CCTTTATATAATTTTTCTCTTTCTTTTTCATCCATTTCAGGCTCGAAAGCTTGATCTAAT
TTCCAACGGTTCGCAATTTCTGCTTTACTATCCCAGAAACCTACCGCAAGTCCTGCAAGA
TAAGCAGCACCTAACGCAGTCGTTTCATTGATTTCAGGACGTTCTACTGTAATATTTTGA
ATATCTGCTTGGAACTGCATTAAGAAGTTGTTCTTAACAGCACCACCATCTACACGGATA
TTCTTCACTTCAATACCTGAGTCTTGAATCATCGCTTCAACCACATCACGTGTTTGGTAA
CAAATAGATTCTAACGTTGCTCTGATAAAGTGTTCTTTCTCAGTCGCACGCGTAATACCA
AATACTGCACCACGCGCATTCGCATCCCAATATGGTGTACCGAGTCCAACAAACGCCGGT
ACCACATAAACACCATCACTTGATTCAATACGGTTCGCATAGTTTTCAGATTCGGGTGCT
GAGTTAATCATACGTAAACCATCTCGCAACCATTGAATAGCTGAACCAGAGACGAAAATA
GAACCTTCTAATGCATAATTCACTTCGCCATCTAAGCCGAATGCGATTGTTGTTAATAAA
CCGTTATCCGATTGAATCGCTTCTTTACCAGTGTTCATTAACATGAATCCACCCGTACCA
TATGTGTTTTTCACTTCACCACGTTCGAAGCATGCTTGTCCGAATAAGGCTGCTTGCTGA
TCCCCTGCGATTCCCGCAATCGGTACTTCTTGTCCAAAGAAATGATAATCTTTTGTTTTC
GCATAAACCTCACTTTTCTTTTGCATGGTTAATCGCTTCAATTGTTTGAGGCATAACACC
GTCGTCTGCTGCAACCACTAAGATTGTGATATCAGTGACTTGCGCACCACGTGCACGCAT
AGTAGTGAAGGCTGCGTGTCCAGGTGTATCTAAGAAAGTGATTTTTTTGCCGTCATTTGT
GATTTGGTAAGCACCGATATGTTGTGTGATACCGCCCGCTTCACCTTGTGTCACATGTGT
ATTTCTGATTGAGTCAAGTAGCGTTGTTTTACCATGGTCAACGTGTCCCATGATTGTTAC
AACTGCTGGACGCTCAATTGCATCCGGATCTTCTTCTTCATCATCGAAGTAGATATCTAA
GTCTTCTTCGTCAATGACTACTTCTTTTTCTACTTCTACACCATAATCATCAGCGATTAA
CTCAATTGTTTCATCGTCTAATGATTGGTTAATGTTTGCCATAATACCAAGTAAGAATAA
TTTCTTAACAATACTTGATGACTCAATATTCATTTTGTCAGCTAATTCGCCAACCGTAAT
ACCTTCTAAATAAGTAATTTTTTCTGGCATTTCTTTTTTCGGTTGTTCTGGTGCTGGTTT
TTGATTATTGTTTTTCTTATGTTTGTTGTTGT
>NAS-10
GATTGCTTGAATACGTTCTTTAGCAAGTTCTACTTCATCGTTTACCACAACGTAGTCATA
AAGATTCATCATTTCCACTTCTTTACGTGCTTCTTTCACACGACTTTGAATCTTTTCGTC
TGATTCTGTACCGCGGCCGACTAAACGCTCTCTTAAGTGGTCTAAACTTGGCGGTGCTAG
GAAGATAAATAATGCATCAGGGAATTTCTTGCGTACTTGTTTCGCACCTTCAACTTCGAT
TTCTAAGAAGACATCATAGCCTTCATCCATAGTGTCTTTCACATACTGTACAGGTGTACC
GTAGTAGTTACCTACATATTCTGCATACTCAATAAATTGATCTTGTGCGATAAGTTGCTC
AAACTCTCGTTTTGTCTTGAAAAAATAATCAACGCCATCTTGTTCACCTTCACGCATTTC
ACGTGTCGTCATTGAAATTGAATATTTGTATGATGTTTTCGGATCATCAAATATTTCTTT
GCGGACAGTACCTTTTCCGACTCCAGATGGACCTGATAAGACAATTAGTAAACCTTTTTC
ATTATCCATGCCTTACGTTTAAACCTTGTAAAACAGGTCCGATTGCTTCATATCCGCCTA
AACGTTGCGCAATTTTGTAACCGATGTTACCTGCTTCTAAGCTTGGGAATACGAATACAT
TTGCGTCACCTTTAATTTGTGCATCTGGTGCTTTTTTAGCCGCCACTTCTGGAACGATTG
CTGCGTCGAATTGGAATTCACCATCGATAACAACATCTTTTAAGCCTTCTGATTCAGCTT
TTTCTTGTGCAGCTTTTGTTGCTTCCGCTACTTTTTCAACATCGTCTGATTTTGCTGAAC
CTTTTGTTGAGAAGCTTAATAATGCTACTTTAGGATCCATGCCGAAGCTTTTCGCTGTTT
TTGCACTTTCTACCGCAATTTCAGCTAAGTCTGTTGCTTCTAATGTAGGGTTGATTGCAC
AATCACCGAAAACATATTGTTCTTCGCCTTTAATCATGAAGAACACACCTGATGTTTTCG
CAACACCTGGTTTAGTTTTGATGATTTGTAGTGCTGGACGAACTGTATCGCCTGTTGAGT
GTGCAGCACCGCTGACTAGTCCTTCAGCTTTACCTGCATAAACAAGCATTGTACCGAAGT

AGTTTACATTGTTAAGCATATCTTCAGCTTGTTCTTTTGTCGCTTTGCCTTTACGACGTT
CTACAAATGCGTCTACTAATTCTGGTTTCAAGTCGCGACTAAAACTTCTTCGTCTTTCGC
AGTTGGAATATTTTTACCTACAAAGTCAGCGCGTATTGGTAATTCGCGGTGTCCGCGATC
CACTAATGTTGCTAACCCGATTTTTTGTGGTCGAGAATGTAGTAAGATTGCATCGAGTGA
CGCACGTACAGTTCGACCCGTATAAAGCACATCATCAATGATGATAACGACTTTGTCTTT
AAGGTCTACATCAATGACATAAGACTTCTCAGACATTTTAGGTGTCGGTAACTCTAAATC
ATCTCTGAATTGCGTGATGTCTAATGTTCCTGTAGGAACACTTACGTCTTCAATTTGTTG
AATCTTCATTTGAATACGTTTCGCTAAAAATTCTCCGCGTGTTTTCACACCAAGCAACAC
AAGATTTTCAGTACCTTTGTTATACTCTAGAATTTCATGTGCGATACGTGTTAAAGTCCG
CTGCATGCCTGCATCATCTAATACAATACGCGACTTGGCAAAGCGAAGAAACTTTTAAGC
CAGCTTCTTCTAATCGTTTACGGCCTTGTTGGAAACTTTTTTCCACGACAATACCGATAC
CGACTGTTTCTGCACCTGCTTGTTCAACTAAACGATTCAAACCAAGTGCCGCATCTCCAT
TCGCTAAAAAGTCATCGATAATCAATACACGGTCATTTTCCCCTAAAAATTCTTCAGAGA
TAATGACGCGATTCGTCTTGTTTTTCGTAAACGAATGAATATCTGTCTGATAAAATCCAT
CTGCCAAAGTACTTGGCTTAGCCTTCTTCGCAAAGAGACATGGTACTTCAAAACGATATG
CTGCCATAATAGCTGGCGCAATGCCTGATGCTTCAACAGTTAAGATTTTAGTGATGCCTT
GATCTTTGAACTGTTCATAAAATGTTTCGCCAATGTCATACATTAACACTGCATCAATTT
GATGGTTTAAAAACCCATCTACCTTCAAGATTTTTTCGCCAATGACGACGCCGTCTTCTT
TAACCTTCTACCTACTAGTGAACCTACAACACGAATACCGTCTAATACTAAACGTGGAAT
TGATAAGTCCATTGATTCTGTTGGTAAGCCGACTGCGACTACAGTACCGCCAGCACGTAC
AGATTCAACAGCTGCGTTGAAGGCTTGTTTAGCAACCGCTGTAACAACTGCCGCATGTGC
ACCGCCTACATGTTCTTGAATGTATTTTGCGACATCCACTTCTTTAGGGTTTAAAGTGAC
ATCTGCATCGTATTGTTTCGCAAATTCAAGTTGACTGTCATTGATGTCTACTGCGATAAC
TTTAGCGTTAAATACTTTTTTCGCATATTGTAAAGCTAGGTTTCCAAGTCCACCTAAACC
GAATAAAACAAGCCATTCACCAGCACGTACGCCTGATTCTTTAATTGCTTTGTATGTTGT
GACACCTGCACATGTGATACTGCTGGCAGCTTCTGATGATAAACCATCTGGTTTTTCCAA
CCTTTATATAATTTTTCTCTTTCTTTTTCATCCATTTCAGGCTCGAAAGCTTGATCTAAT
TTCCAACGGTTCGCAATTTCTGCTTTACTATCCCAGAAACCTACCGCAAGTCCTGCAAGA
TAAGCAGCACCTAACGCAGTCGTTTCATTGATTTCAGGACGTTCTACTGTAATATTTTGA
ATATCTGCTTGGAACTGCATTAAGAAGTTGTTCTTAACAGCACCACCATCTACACGGATA
TTCTTCACTTCAATACCTGAGTCTTGAATCATCGCTTCAACCACATCACGTGTTTGGTAA
CAAATAGATTCTAACGTTGCTCTGATAAAGTGTTCTTTCTCAGTCGCACGCGTAATACCA
AATACTGCACCACGCGCATTCGCATCCCAATATGGTGTACCGAGTCCAACAAACGCCGGT
ACCACATAAACACCATCACTTGATTCAATACGGTTCGCATAGTTTTCAGATTCGGGTGCT
GAGTTAATCATACGTAAACCATCTCGCAACCATTGAATAGCTGAACCAGAGACGAAAATA
GAACCTTCTAATGCATAATTCACTTCGCCATCTAAGCCGAATGCGATTGTTGTTAATAAA
CCGTTATCCGATTGAATCGCTTCTTTACCAGTGTTCATTAACATGAATCCACCCGTACCA
TATGTGTTTTTCACTTCACCACGTTCGAAGCATGCTTGTCCGAATAAGGCTGCTTGCTGA
TCCCCTGCGATTCCCGCAATCGGTACTTCTTGTCCAAAGAAATGATAATCTTTTGTTTTC
GCATAAACCTCACTTTTCTTTTGCATGGTTAATCGCTTCAATTGTTTGAGGCATAACACC
GTCGTCTGCTGCAACCACTAAGATTGTGATATCAGTGACTTGCGCACCACGTGCACGCAT
AGTAGTGAAGGCTGCGTGTCCAGGTGTATCTAAGAAAGTGATTTTTTTGCCGTCATTTGT
GATTTGGTAAGCACCGATATGTTGTGTGATACCGCCCGCTTCACCTTGTGTCACATGTGT
ATTTCTGATTGAGTCAAGTAGCGTTGTTTTACCATGGTCAACGTGTCCCATGATTGTTAC
AACTGCTGGACGCTCAATTGCATCCGGATCTTCTTCTTCATCATCGAAGTAGATATCTAA
GTCTTCTTCGTCAATGACTACTTCTTTTTCTACTTCTACACCATAATCATCAGCGATTAA
CTCAATTGTTTCATCGTCTAATGATTGGTTAATGTTTGCCATAATACCAAGTAAGAATAA
TTTCTTAACAATACTTGATGACTCAATATTCATTTTGTCAGCTAATTCGCCAACCGTAAT
ACCTTCTAAATAAGTAATTTTTTCTGGCATTTCTTTTTTCGGTTGTTCTGGTGCTGGTTT
TTGATTATTGTTTTTCTTATGTTTGTTGTTGT
>NAS-41
GATTGCTTGAATGCGTTCTTTCGCAAGTTCTACTTCATCGTTTACCACAACGTAGTCATA
AAGATTCATCATTTCCACTTCTTTACGTGCCTCTTTCACACGACTTTGAATCTTTTCATC
TGTTTCTGTACCGCGGCCGACTAAACGCTCTCTTAAGTGGTCTAAACTTGGCGGTGCTAG
GAAGATAAATAATGCATCAGGGAATTTCTTGCGTACTTGTTTCGCACCTTCAACTTCGAT
TTCTAAGAAGACATCATAACCTTCATCCATAGTGTCTTTCACATACTGTACAGGTGTACC
GTAGTAGTTACCTACATATTCTGCATACTCAATAAATTGATCTTGTGCGATAAGTTGCTC

AAACTCTCGTTTTGTCTTGAAAAAATAATCAACGCCATCTTGTTCACCTTCACGCATTTC
ACGTGTTGTCATTGAAATTGAATATTTGTATGATGTTTTCGGATCATCAAATATTTCTTT
GCGGACAGTACCTTTTCCGACTCCAGATGGACCTGATAAGACAATTAGTAAACCTTTTTC
ATTATCCATGCCTTACGTTTAAACCTTGTAAAACAGGTCCGATTGCTTCATATCCGCCTA
AACGTTGCGCAATTTTGTAACCGATGTTACCTGCTTCTAAGCTTGGGAATACGAATACAT
TTGCGTCACCTTTAATTTGTGCATCTGGTGCTTTTTTAGATGCAACTTCTGGAACGATTG
CTGCGTCAAATTGGAATTCACCATCGATGACAACGTCTTTCAAGCCTTCTGATTCAGCTT
TTTCTTGTGCAGCTTTTGTTGCTTCCGCTACTTTTTCAACATCGTCTGATTTCGCTGAAC
CTTTTGTTGAGAAGCTTAATAACGCTACTTTAGGGTCCATGCCGAAGCTTTTCGCTGTTT
TTGCACTTTCTACTGCAATTTCAGCTAAGTCTGTTGCTTCTAATGTAGGGTTGATTGCAC
AGTCGCCGAAAACATATTGTTCTTCGCCTTTAATCATAAAGAACACACCTGATGTTTTCG
CAACACCTGGTTTAGTTTTGATGATTTGTAATGCTGGACGAACTGTATCGCCTGTTGAGT
GTGCAGCACCGCTGACTAGTCCTTCAGCTTTACCTGCATAAACAAGCATCGTACCGAAGT
AGTTTACATTGTTAAGCATATCTTCTGCTTGCTCTTTAGTCGCTTTACCTTTACGACGTT
CTACAAATGCGTCTACTAATTCTGGTTTCAAGTCGCGACTGAAACTTCTTCGTCTTTCGC
AGTTGGAATATTTTTACCTACGAAGTCAGCGCGTATTGGTAATTCGCGGTGTCCGCGATC
CACTAATGTTGCTAACCCGATTTTTTGTGGTCGAGAATGTAGTAAGATTGCATCAAGTGA
CGCACGTACAGTTCGACCTGTATAAAGCACATCATCAATAATGATAACGACTTTGTCTTT
AAGGTCTACGTCAATGACATAAGACTTCTCAGACATTTTAGGTGTCGGTAACTCTAAATC
ATCTCTAAATTGCGTGATGTCTAATGTTCCTGTAGGAACACTTACGTCTTCAATTTGTTG
AATCTTCGTTTGAATACGTTTCGCTAAAAATTCTCCGCGTGTTTTCACACCAAGCAATAC
AAGATTTTCAGTACCCTTGTTATACTCTAGAATTTCATGTGCGATACGTGTTAAAGTCCG
TTGCATACCTGCATCATCTAATACAATACGCGACTTGGCAAAGAGAAGAAACCTTCAAGC
CAGCTTCTTCTAATCGTTTACGACCTTGTTGGAAACTTTTCTCTACTACAATACCGATGC
CGACTGTTTCTGCACCTGCTTGTTCAACTAAGCGGTTCAAACCAAGTGCCGCATCTCCAT
TCGCTAAAAAGTCATCGATAATCAACACACGGTCATTTTCACCTAAAAATTCTTCAGAGA
TAATGACACGATTCGTCTTGTTTTTCGTAAATGAATGAATATCTGTTTGATAAAATCCAT
CTGCCAAAGTACTTGGCTTAGCCTTCTTCGCAAAGAGACATGGTACTTCAAATCGATATG
CTGCCATAATGGCTGGTGCGATACCTGATGCCTCAACAGTTAAGATTTTAGTGATGCCTT
GATCTTTAAACTGTTCATAAAATGTTTCACCGATGTCGTACATTAATACTGCATCAATTT
GATGGTTTAAAAACCCATCTACCTTCAAGATTTTTTCGCCGATGACGACGCCGTCTTCTT
TAACCTTCTACCTACTAGTGAACCTACTACGCGGATACCGTCTAATACTAAACGTGGAAT
TGATAAGTCCATAGATTCTGTTGGTAAGCCGACTGCGACTACAGTACCGCCAGCACGTAC
AGATTCAACAGCTGCGTTGAAGGCTTGTTTAGCAACCGCTGTAACAACTGCCGCATGTGC
ACCGTCTACATGTTCTTGAATGTATTTTGCGACATCTACTTCTTTAGGGTTTAAAGTAAC
ATCTGCATCGTATTGTTTCGCAAATTCAAGTTGACTGTCATTAATGTCTACTGCGATAAC
TTTAGCGTTAAATACTTTTTTCGCATATTGTAAAGCTAGGTTTCCGAGTCCGCCTAAACC
GAATAAGACAAGCCATTCACCAGCGCGTACGCCTGATTCTTTAATTGCTTTGTATGTTGT
GACACCTGCGCATGTGATACTGCTGGCAGCTTCTGATGATAAACCATCTGGTTTTTCCAC
CTTTATATAATTTTTCTCTTTCTTTTTCATCCATTTCTGGTTCGAACGCTTGATCTAATT
TCCAACGATTTGTGATTTCTTCTTTACTATCCCAGAAACCTACCGCAAGTCCTGCGAGAT
AAGCAGCACCTAACGCAGTTGTTTCATTAATTTCAGGTCGTTCTACTGTAATATTTTGAA
TATCTGCTTGGAATTGCATTAAGAAGTTATTCTTAACAGCACCGCCGTCTACACGAATGT
TTTTCACTTCAATACCGGAATCTTGAATCATTGCTTCAACCACGTCACGTGTTTGGTAGC
AAATAGATTCTAAAGTAGCTCTGATAAAGTGTTCTTTCTCAGTCGCACGTGTAATACCGA
ATACTGCACCGCGCGCATTCGCATCCCAATACGGTGTACCTAGTCCGACGAATGCAGGCA
CAACATAAACACCATCACTTGATTCAATACGGTTCGCGTAGTTTTCAGATTCAGGTGCTG
AGTTAATCATACGCAAACCATCACGTAACCATTGAATCGCTGAACCTGAGACAAAGATAG
AACCTTCTAATGCATAATGTACTTCTCCATCTAAACCGAAAGCAATCGTTGTTAATAAAC
CGTTATCAGATTGAATCGCTTCTTTACCAGTGTTCATTAACATGAAACCACCTGTACCAT
ACGTGTTTTTCACTTCCCCGCGTTCAAAGCACGCTTGTCCGAATAATGCTGCTTGCTGGT
CTCCGGCGATACCTGCAATCGGTACTTCTTGTCCAAAGAAATGATAATCTTTTGTTTTCG
CATAAATCTCACTTTTCTTTCGCATGGTTAATTGCTTCAATTGTTTGAGGCATAACACCA
TCATCTGCCGCAACTACTAAGATAGTGATATCAGTAACTTGCGCACCGCGCGCACGCATT
GTAGTGAAGGCTGCGTGTCCAGGTGTATCTAAGAAAGTGATTTTTTTGCCGTCGTTTGTG
ATTTGGTAAGCACCGATATGTTGTGTGATACCGCCTGCTTCACCTTGTGTCACATGTGTA
TTTCTGATTGAGTCAAGTAGTGTTGTTTTACCATGGTCAACGTGTCCCATGATTGTAACA

ACTGCTGGGCGCTCAATTGCATCTGGATCTTCTTCTTCATCGTCGAAGTAGATATCTAAG
TCTTCTTCATCAATGACAACTTCTTTTTCTACTTCTACGCCGTAATCATCAGCAATTAAC
TCAATTGTTTCATCGTCTAATGATTGGTTGATGTTAGCCATAATACCAAGTAAGAATAAT
TTCTTAACGATACTTGATGACTCAATATTCATTTTGTCAGCTAATTCGCCAACCGTAATA
CCTTCTAAGTAAGTAATTTTTTCTGGTATTTCTTTTTTCGGTTGTTCTGGTGCTGGTTTT
TGATTATTGTTTTTCTTATGTTTGTTGTTGT
>NAS-5
GATTGCTTGAATACGTTCTTTAGCAAGTTCTACTTCATCGTTTACCACAACGTAGTCATA
AAGATTCATCATTTCCACTTCTTTACGTGCTTCTTTCACACGACTTTGAATCTTTTCGTC
TGATTCTGTACCGCGGCCGACTAAACGCTCTCTTAAGTGGTCTAAACTTGGCGGTGCTAG
GAAGATAAATAATGCATCAGGGAATTTCTTGCGTACTTGTTTCGCACCTTCAACTTCGAT
TTCTAAGAAGACATCATAGCCTTCATCCATAGTGTCTTTCACATACTGTACAGGTGTACC
GTAGTAGTTACCTACATATTCTGCATACTCAATAAATTGATCTTGTGCGATAAGTTGCTC
AAACTCTCGTTTTGTCTTGAAAAAATAATCAACGCCATCTTGTTCACCTTCACGCATTTC
ACGTGTCGTCATTGAAATTGAATATTTGTATGATGTTTTCGGATCATCAAATATTTCTTT
GCGGACAGTACCTTTTCCGACTCCAGATGGACCTGATAAGACAATTAGTAAACCTTTTTC
ATTATCCATGCCTTACGTTTAAACCTTGTAAAACAGGTCCGATTGCTTCATATCCGCCTA
AACGTTGCGCAATTTTGTAACCGATGTTACCTGCTTCTAAGCTTGGGAATACGAATACAT
TTGCGTCACCTTTAATTTGTGCATCTGGTGCTTTTTTAGCCGCCACTTCTGGAACGATTG
CTGCGTCGAATTGGAATTCACCATCGATAACAACATCTTTTAAGCCTTCTGATTCAGCTT
TTTCTTGTGCAGCTTTTGTTGCTTCCGCTACTTTTTCAACATCGTCTGATTTTGCTGAAC
CTTTTGTTGAGAAGCTTAATAATGCTACTTTAGGATCCATGCCGAAGCTTTTCGCTGTTT
TTGCACTTTCTACCGCAATTTCAGCTAAGTCTGTTGCTTCTAATGTAGGGTTGATTGCAC
AATCACCGAAAACATATTGTTCTTCGCCTTTAATCATGAAGAACACACCTGATGTTTTCG
CAACACCTGGTTTAGTTTTGATGATTTGTAGTGCTGGACGAACTGTATCGCCTGTTGAGT
GTGCAGCACCGCTGACTAGTCCTTCAGCTTTACCTGCATAAACAAGCATTGTACCGAAGT
AGTTTACATTGTTAAGCATATCTTCAGCTTGTTCTTTTGTCGCTTTGCCTTTACGACGTT
CTACAAATGCGTCTACTAATTCTGGTTTCAAGTCGCGACTAAAACTTCTTCGTCTTTCGC
AGTTGGAATATTTTTACCTACAAAGTCAGCGCGTATTGGTAATTCGCGGTGTCCGCGATC
CACTAATGTTGCTAACCCGATTTTTTGTGGTCGAGAATGTAGTAAGATTGCATCGAGTGA
CGCACGTACAGTTCGACCCGTATAAAGCACATCATCAATGATGATAACGACTTTGTCTTT
AAGGTCTACATCAATGACATAAGACTTCTCAGACATTTTAGGTGTCGGTAACTCTAAATC
ATCTCTGAATTGCGTGATGTCTAATGTTCCTGTAGGAACACTTACGTCTTCAATTTGTTG
AATCTTCATTTGAATACGTTTCGCTAAAAATTCTCCGCGTGTTTTCACACCAAGCAACAC
AAGATTTTCAGTACCTTTGTTATACTCTAGAATTTCATGTGCGATACGTGTTAAAGTCCG
CTGCATGCCTGCATCATCTAATACAATACGCGACTTGGCAAAGCGAAGAAACTTTTAAGC
CAGCTTCTTCTAATCGTTTACGGCCTTGTTGGAAACTTTTTTCCACGACAATACCGATAC
CGACTGTTTCTGCACCTGCTTGTTCAACTAAACGATTCAAACCAAGTGCCGCATCTCCAT
TCGCTAAAAAGTCATCGATAATCAATACACGGTCATTTTCCCCTAAAAATTCTTCAGAGA
TAATGACGCGATTCGTCTTGTTTTTCGTAAACGAATGAATATCTGTCTGATAAAATCCAT
CTGCCAAAGTACTTGGCTTAGCCTTCTTCGCAAAGAGACATGGTACTTCAAAACGATATG
CTGCCATAATAGCTGGCGCAATGCCTGATGCTTCAACAGTTAAGATTTTAGTGATGCCTT
GATCTTTGAACTGTTCATAAAATGTTTCGCCAATGTCATACATTAACACTGCATCAATTT
GATGGTTTAAAAACCCATCTACCTTCAAGATTTTTTCGCCAATGACGACGCCGTCTTCTT
TAACCTTCTACCTACTAGTGAACCTACAACACGAATACCGTCTAATACTAAACGTGGAAT
TGATAAGTCCATTGATTCTGTTGGTAAGCCGACTGCGACTACAGTACCGCCAGCACGTAC
AGATTCAACAGCTGCGTTGAAGGCTTGTTTAGCAACCGCTGTAACAACTGCCGCATGTGC
ACCGCCTACATGTTCTTGAATGTATTTTGCGACATCCACTTCTTTAGGGTTTAAAGTGAC
ATCTGCATCGTATTGTTTCGCAAATTCAAGTTGACTGTCATTGATGTCTACTGCGATAAC
TTTAGCGTTAAATACTTTTTTCGCATATTGTAAAGCTAGGTTTCCAAGTCCACCTAAACC
GAATAAAACAAGCCATTCACCAGCACGTACGCCTGATTCTTTAATTGCTTTGTATGTTGT
GACACCTGCACATGTGATACTGCTGGCAGCTTCTGATGATAAACCATCTGGTTTTTCCAA
CCTTTATATAATTTTTCTCTTTCTTTTTCATCCATTTCAGGCTCGAAAGCTTGATCTAAT
TTCCAACGGTTCGCAATTTCTGCTTTACTATCCCAGAAACCTACCGCAAGTCCTGCAAGA
TAAGCAGCACCTAACGCAGTCGTTTCATTGATTTCAGGACGTTCTACTGTAATATTTTGA
ATATCTGCTTGGAACTGCATTAAGAAGTTGTTCTTAACAGCACCACCATCTACACGGATA
TTCTTCACTTCAATACCTGAGTCTTGAATCATCGCTTCAACCACATCACGTGTTTGGTAA

CAAATAGATTCTAACGTTGCTCTGATAAAGTGTTCTTTCTCAGTCGCACGCGTAATACCA
AATACTGCACCACGCGCATTCGCATCCCAATATGGTGTACCGAGTCCAACAAACGCCGGT
ACCACATAAACACCATCACTTGATTCAATACGGTTCGCATAGTTTTCAGATTCGGGTGCT
GAGTTAATCATACGTAAACCATCTCGCAACCATTGAATAGCTGAACCAGAGACGAAAATA
GAACCTTCTAATGCATAATTCACTTCGCCATCTAAGCCGAATGCGATTGTTGTTAATAAA
CCGTTATCCGATTGAATCGCTTCTTTACCAGTGTTCATTAACATGAATCCACCCGTACCA
TATGTGTTTTTCACTTCACCACGTTCGAAGCATGCTTGTCCGAATAAGGCTGCTTGCTGA
TCCCCTGCGATTCCCGCAATCGGTACTTCTTGTCCAAAGAAATGATAATCTTTTGTTTTC
GCATAAACCTCACTTTTCTTTTGCATGGTTAATCGCTTCAATTGTTTGAGGCATAACACC
GTCGTCTGCTGCAACCACTAAGATTGTGATATCAGTGACTTGCGCACCACGTGCACGCAT
AGTAGTGAAGGCTGCGTGTCCAGGTGTATCTAAGAAAGTGATTTTTTTGCCGTCATTTGT
GATTTGGTAAGCACCGATATGTTGTGTGATACCGCCCGCTTCACCTTGTGTCACATGTGT
ATTTCTGATTGAGTCAAGTAGCGTTGTTTTACCATGGTCAACGTGTCCCATGATTGTTAC
AACTGCTGGACGCTCAATTGCATCCGGATCTTCTTCTTCATCATCGAAGTAGATATCTAA
GTCTTCTTCGTCAATGACTACTTCTTTTTCTACTTCTACACCATAATCATCAGCGATTAA
CTCAATTGTTTCATCGTCTAATGATTGGTTAATGTTTGCCATAATACCAAGTAAGAATAA
TTTCTTAACAATACTTGATGACTCAATATTCATTTTGTCAGCTAATTCGCCAACCGTAAT
ACCTTCTAAATAAGTAATTTTTTCTGGCATTTCTTTTTTCGGTTGTTCTGGTGCTGGTTT
TTGATTATTGTTTTTCTTATGTTTGTTGTTGT
>NAS-1
GATTGCTTGAATACGTTCTTTAGCAAGTTCTACTTCATCGTTTACCACAACGTAGTCATA
AAGATTCATCATTTCCACTTCTTTACGTGCTTCTTTCACACGACTTTGAATCTTTTCGTC
TGATTCTGTACCGCGGCCGACTAAACGCTCTCTTAAGTGGTCTAAACTTGGCGGTGCTAG
GAAGATAAATAATGCATCAGGGAATTTCTTGCGTACTTGTTTCGCACCTTCAACTTCGAT
TTCTAAGAAGACATCATAGCCTTCATCCATAGTGTCTTTCACATACTGTACAGGTGTACC
GTAGTAGTTACCTACATATTCTGCATACTCAATAAATTGATCTTGTGCGATAAGTTGCTC
AAACTCTCGTTTTGTCTTGAAAAAATAATCAACGCCATCTTGTTCACCTTCACGCATTTC
ACGTGTCGTCATTGAAATTGAATATTTGTATGATGTTTTCGGATCATCAAATATTTCTTT
GCGGACAGTACCTTTTCCGACTCCAGATGGACCTGATAAGACAATTAGTAAACCTTTTTC
ATTATCCATGCCTTACGTTTAAACCTTGTAAAACAGGTCCGATTGCTTCATATCCGCCTA
AACGTTGCGCAATTTTGTAACCGATGTTACCTGCTTCTAAGCTTGGGAATACGAATACAT
TTGCGTCACCTTTAATTTGTGCATCTGGTGCTTTTTTAGCCGCCACTTCTGGAACGATTG
CTGCGTCGAATTGGAATTCACCATCGATAACAACATCTTTTAAGCCTTCTGATTCAGCTT
TTTCTTGTGCAGCTTTTGTTGCTTCCGCTACTTTTTCAACATCGTCTGATTTTGCTGAAC
CTTTTGTTGAGAAGCTTAATAATGCTACTTTAGGATCCATGCCGAAGCTTTTCGCTGTTT
TTGCACTTTCTACCGCAATTTCAGCTAAGTCTGTTGCTTCTAATGTAGGGTTGATTGCAC
AATCACCGAAAACATATTGTTCTTCGCCTTTAATCATGAAGAACACACCTGATGTTTTCG
CAACACCTGGTTTAGTTTTGATGATTTGTAGTGCTGGACGAACTGTATCGCCTGTTGAGT
GTGCAGCACCGCTGACTAGTCCTTCAGCTTTACCTGCATAAACAAGCATTGTACCGAAGT
AGTTTACATTGTTAAGCATATCTTCAGCTTGTTCTTTTGTCGCTTTGCCTTTACGACGTT
CTACAAATGCGTCTACTAATTCTGGTTTCAAGTCGCGACTAAAACTTCTTCGTCTTTCGC
AGTTGGAATATTTTTACCTACAAAGTCAGCGCGTATTGGTAATTCGCGGTGTCCGCGATC
CACTAATGTTGCTAACCCGATTTTTTGTGGTCGAGAATGTAGTAAGATTGCATCGAGTGA
CGCACGTACAGTTCGACCCGTATAAAGCACATCATCAATGATGATAACGACTTTGTCTTT
AAGGTCTACATCAATGACATAAGACTTCTCAGACATTTTAGGTGTCGGTAACTCTAAATC
ATCTCTGAATTGCGTGATGTCTAATGTTCCTGTAGGAACACTTACGTCTTCAATTTGTTG
AATCTTCATTTGAATACGTTTCGCTAAAAATTCTCCGCGTGTTTTCACACCAAGCAACAC
AAGATTTTCAGTACCTTTGTTATACTCTAGAATTTCATGTGCGATACGTGTTAAAGTCCG
CTGCATGCCTGCATCATCTAATACAATACGCGACTTGGCAAAGCGAAGAAACTTTTAAGC
CAGCTTCTTCTAATCGTTTACGGCCTTGTTGGAAACTTTTTTCCACGACAATACCGATAC
CGACTGTTTCTGCACCTGCTTGTTCAACTAAACGATTCAAACCAAGTGCCGCATCTCCAT
TCGCTAAAAAGTCATCGATAATCAATACACGGTCATTTTCCCCTAAAAATTCTTCAGAGA
TAATGACGCGATTCGTCTTGTTTTTCGTAAACGAATGAATATCTGTCTGATAAAATCCAT
CTGCCAAAGTACTTGGCTTAGCCTTCTTCGCAAAGAGACATGGTACTTCAAAACGATATG
CTGCCATAATAGCTGGCGCAATGCCTGATGCTTCAACAGTTAAGATTTTAGTGATGCCTT
GATCTTTGAACTGTTCATAAAATGTTTCGCCAATGTCATACATTAACACTGCATCAATTT
GATGGTTTAAAAACCCATCTACCTTCAAGATTTTTCGCCAATGACGACGCCGTCTTCTT

TAACCTTCTACCTACTAGTGAACCTACAACACGAATACCGTCTAATACTAAACGTGGAAT
TGATAAGTCCATTGATTCTGTTGGTAAGCCGACTGCGACTACAGTACCGCCAGCACGTAC
AGATTCAACAGCTGCGTTGAAGGCTTGTTTAGCAACCGCTGTAACAACTGCCGCATGTGC
ACCGCCTACATGTTCTTGAATGTATTTTGCGACATCCACTTCTTTAGGGTTTAAAGTGAC
ATCTGCATCGTATTGTTTCGCAAATTCAAGTTGACTGTCATTGATGTCTACTGCGATAAC
TTTAGCGTTAAATACTTTTTTCGCATATTGTAAAGCTAGGTTTCCAAGTCCACCTAAACC
GAATAAAACAAGCCATTCACCAGCACGTACGCCTGATTCTTTAATTGCTTTGTATGTTGT
GACACCTGCACATGTGATACTGCTGGCAGCTTCTGATGATAAACCATCTGGTTTTTCCAA
CCTTTATATAATTTTTCTCTTTCTTTTTCATCCATTTCAGGCTCGAAAGCTTGATCTAAT
TTCCAACGGTTCGCAATTTCTGCTTTACTATCCCAGAAACCTACCGCAAGTCCTGCAAGA
TAAGCAGCACCTAACGCAGTCGTTTCATTGATTTCAGGACGTTCTACTGTAATATTTTGA
ATATCTGCTTGGAACTGCATTAAGAAGTTGTTCTTAACAGCACCACCATCTACACGGATA
TTCTTCACTTCAATACCTGAGTCTTGAATCATCGCTTCAACCACATCACGTGTTTGGTAA
CAAATAGATTCTAACGTTGCTCTGATAAAGTGTTCTTTCTCAGTCGCACGCGTAATACCA
AATACTGCACCACGCGCATTCGCATCCCAATATGGTGTACCGAGTCCAACAAACGCCGGT
ACCACATAAACACCATCACTTGATTCAATACGGTTCGCATAGTTTTCAGATTCGGGTGCT
GAGTTAATCATACGTAAACCATCTCGCAACCATTGAATAGCTGAACCAGAGACGAAAATA
GAACCTTCTAATGCATAATTCACTTCGCCATCTAAGCCGAATGCGATTGTTGTTAATAAA
CCGTTATCCGATTGAATCGCTTCTTTACCAGTGTTCATTAACATGAATCCACCCGTACCA
TATGTGTTTTTCACTTCACCACGTTCGAAGCATGCTTGTCCGAATAAGGCTGCTTGCTGA
TCCCCTGCGATTCCCGCAATCGGTACTTCTTGTCCAAAGAAATGATAATCTTTTGTTTTC
GCATAAACCTCACTTTTCTTTTGCATGGTTAATCGCTTCAATTGTTTGAGGCATAACACC
GTCGTCTGCTGCAACCACTAAGATTGTGATATCAGTGACTTGCGCACCACGTGCACGCAT
AGTAGTGAAGGCTGCGTGTCCAGGTGTATCTAAGAAAGTGATTTTTTTGCCGTCATTTGT
GATTTGGTAAGCACCGATATGTTGTGTGATACCGCCCGCTTCACCTTGTGTCACATGTGT
ATTTCTGATTGAGTCAAGTAGCGTTGTTTTACCATGGTCAACGTGTCCCATGATTGTTAC
AACTGCTGGACGCTCAATTGCATCCGGATCTTCTTCTTCATCATCGAAGTAGATATCTAA
GTCTTCTTCGTCAATGACTACTTCTTTTTCTACTTCTACACCATAATCATCAGCGATTAA
CTCAATTGTTTCATCGTCTAATGATTGGTTAATGTTTGCCATAATACCAAGTAAGAATAA
TTTCTTAACAATACTTGATGACTCAATATTCATTTTGTCAGCTAATTCGCCAACCGTAAT
ACCTTCTAAATAAGTAATTTTTTCTGGCATTTCTTTTTTCGGTTGTTCTGGTGCTGGTTT
TTGATTATTGTTTTTCTTATGTTTGTTGTTGT
>NAS-209
GATTGCTTGAATACGTTCTTTAGCAAGTTCTACTTCATCGTTTACCACAACGTAGTCATA
AAGATTCATCATTTCCACTTCTTTACGTGCTTCTTTCACACGACTTTGAATCTTTTCGTC
TGATTCTGTACCGCGGCCGACTAAACGCTCTCTTAAGTGGTCTAAACTTGGCGGTGCTAG
GAAGATAAATAATGCATCAGGGAATTTCTTGCGTACTTGTTTCGCACCTTCAACTTCGAT
TTCTAAGAAGACATCATAGCCTTCATCCATAGTGTCTTTCACATACTGTACAGGTGTACC
GTAGTAGTTACCTACATATTCTGCATACTCAATAAATTGATCTTGTGCGATAAGTTGCTC
AAACTCTCGTTTTGTCTTGAAAAAATAATCAACGCCATCTTGTTCACCTTCACGCATTTC
ACGTGTCGTCATTGAAATTGAATATTTGTATGATGTTTTCGGATCATCAAATATTTCTTT
GCGGACAGTACCTTTTCCGACTCCAGATGGACCTGATAAGACAATTAGTAAACCTTTTTC
ATTATCCATGCCTTACGTTTAAACCTTGTAAAACAGGTCCGATTGCTTCATATCCGCCTA
AACGTTGCGCAATTTTGTAACCGATGTTACCTGCTTCTAAGCTTGGGAATACGAATACAT
TTGCGTCACCTTTAATTTGTGCATCTGGTGCTTTTTTAGCCGCCACTTCTGGAACGATTG
CTGCGTCGAATTGGAATTCACCATCGATAACAACGTCTTTTAAGCCTTCTGATTCAGCTT
TTTCTTGTGCAGCTTTTGTTGCTTCCGCTACTTTTTCAACATCGTCTGATTTTGCTGAAC
CTTTTGTTGAGAAGCTTAATAATGCTACTTTAGGATCCATGCCGAAGCTTTTCGCTGTTT
TTGCACTTTCTACCGCAATTTCAGCTAAGTCTGTTGCTTCTAATGTAGGGTTGATTGCAC
AATCACCGAAAACATATTGTTCTTCGCCTTTAATCATGAAGAACACACCTGATGTTTTCG
CAACACCTGGTTTAGTTTTGATGATTTGTAGTGCTGGACGAACTGTATCGCCTGTTGAAT
GTGCAGCACCGCTGACTAGTCCTTCAGCTTTACCTGCATAAACAAGCATTGTACCGAAGT
AGTTTACATTGTTAAGCATATCTTCAGCTTGTTCTTTTGTCGCTTTGCCTTTACGACGTT
CTACAAATGCGTCTACTAATTCTGGTTTCAAGTCGCGACTAAAACTTCTTCGTCTTTCGC
AGTTGGAATATTTTTACCTACGAAGTCAGCGCGTATTGGTAATTCGCGGTGTCCGCGATC
CACTAATGTTGCTAACCCGATTTTTTGTGGTCGAGAATGTAGTAAGATTGCATCGAGTGA
CGCACGTACAGTTCGACCTGTATAAAGCACATCATCAATGATGATAACGACTTTGTCTTT

AAGGTCTACATCAATGACATAAGACTTCTCAGACATTTTAGGTGTCGGTAACTCTAAGTC
ATCTCTGAATTGCGTGATGTCTAATGTTCCTGTAGGAACACTTACGTCTTCAATTTGTTG
AATCTTCGTTTGAATACGTTTCGCTAAAAATTCTCCGCGTGTTTTCACACCAAGCAACAC
AAGATTTTCAGTACCTTTGTTATACTCTAGAATTTCATGTGCGATACGTGTTAAAGTCCG
CTGCATGCCTGCATCATCTAATACAATACGCGACTTGGCAAAGTGAAGAAACTTTCAAGC
CAGCTTCTTCTAAACGTTTACGGCCTTGTTGGAAACTTTTCTCCACGACAATACCGATAC
CGACTGTTTCTGCACCTGCTTGTTCAACTAAACGATTCAAACCAAGTGCCGCATCTCCAT
TCGCTAAAAAGTCATCGATAATCAATACACGATCATTTTCTCCTAAAAATTCTTCAGAGA
TAATGACACGATTCGTCTTGTTTTTCGTAAACGAATGAATATCTGTCTGATAAAATCCAT
CTGCCAAAGTACTTGGCTTAGCCTTCTTCGCAAAGAGACATGGTACTTCAAAACGATATG
CTGCCATAATAGCTGGCGCAATACCTGATGCTTCAACAGTTAAGATTTTAGTGATGCCTT
GATCTTTAAACTGTTCATAAAATGTTTCGCCAATGTCATACATTAACACTGCATCAATTT
GATGGTTTAGAAACCCATCTACCTTCAAGATTTTTTCGCCAATGACGACGCCGTCTTCTT
TAACCTTCTACCTACTAGTGAACCTACAACACGAATACCGTCTAATACTAAACGTGGAAT
TGATAAGTCCATAGATTCTGTTGGTAAGCCGACTGCGACTACAGTACCGCCAGCACGTAC
AGATTCAACAGCTGCGTTGAAGGCTTGTTTAGCAACTGCTGTAACAACTGCCGCATGTGC
ACCGCCTACATGTTCTTGAATGTATTTTGCGACATCCACTTCTTTAGGGTTTAAAGTGAC
ATCTGCATCGTATTGTTTCGCAAATTCAAGTTGACTGTCATTGATGTCTACTGCGATAAC
TTTAGCGTTAAATACTTTTTTCGCATATTGTAAAGCTAGGTTTCCAAGTCCACCTAAACC
GAATAAAACAAGCCATTCACCAGCACGTACGCCTGATTCTTTAATTGCTTTGTATGTTGT
GACACCTGCACATGTGATACTGCTGGCAGCTTCTGATGATAAACCATCTGGTTTTTCCAA
CCTTTATATAATTTTTCTCTTTCTTTTTCATCCATTTCAGGTTCGAAAGCTTGATCTAAT
TTCCAACGGTTCGCAATTTCTGCTTTACTATCCCAGAAACCTACCGCAAGTCCTGCAAGA
TAAGCAGCACCTAACGCAGTCGTTTCATTGATTTCAGGACGTTCTACTGTAATGTTTTGA
ATATCTGCTTGGAACTGCATTAAGAAGTTGTTCTTAACAGCACCACCATCTACACGGATA
TTCTTCACTTCAATACCTGAGTCTTGAATCATCGCTTCAACCACATCACGTGTTTGGTAA
CAAATAGATTCTAACGTTGCTCTGATAAAGTGTTCTTTCTCAGTCGCACGTGTAATACCA
AATACTGCACCACGCGCATTCGCATCCCAATAAGGTGTACCGAGTCCAACAAATGCAGGC
ACTACATAAACGCCATCACTTGATTCAATACGGTTCGCATAGTTTTCAGATTCAGGTGCT
GAGTTAATCATACGTAAACCATCTCGCAACCATTGAATAGCTGAACCAGAGACGAAAATA
GAACCTTCTAATGCATAATTCACTTCGCCATCTAAGCCGAATGCGATTGTTGTTAATAAA
CCGTTATCCGATTGAATCGCTTCTTTACCAGTGTTCATTAACATGAATCCACCCGTACCA
TATGTGTTTTTCACTTCACCACGTTCGAAGCATGCTTGTCCGAATAAGGCTGCTTGCTGA
TCCCCTGCGATTCCCGCAATCGGTACTTCTTGTCCAAAGAAATGATAATCTTTTGTTTTC
GCATAAATCTCACTTTTCTTTTGCATGGTTAATCGCTTCAATTGTTTGAGGCATAACACC
GTCGTCTGCTGCAACCACTAAGATAGTGATATCAGTGACTTGCGCACCACGTGCACGCAT
AGTAGTGAAGGCTGCGTGTCCAGGTGTATCTAAGAAAGTGATTTTTTTGCCGTCATTTGT
GATTTGGTAAGCACCGATATGTTGTGTGATACCGCCCGCTTCACCTTGTGTCACATGTGT
ATTTCTGATTGAGTCAAGTAGCGTTGTTTTACCATGGTCAACGTGTCCCATGATTGTTAC
AACTGCTGGACGCTCAATTGCATCCGGATCTTCTTCTTCATCATCGAAGTAGATGTCTAA
GTCTTCTTCGTCAATGACTACTTCTTTTTCTACTTCTACACCATAATCATCAGCGATTAA
CTCAATTGTTTCATCGTCTAATGATTGGTTAATGTTTGCCATAATACCAAGTAAGAATAA
TTTCTTAACGATACTTGATGACTCAATATTCATTTTGTCAGCTAATTCGCCAACCGTAAT
ACCTTCTAAATAAGTAATTTTTTCTGGCATTTCTTTTTTCGGTTGTTCTGGTGCTGGTTT
TTGATTATTGTTTTTCTTATGTTTGTTGTTGT
>NAS-214
GATTGCTTGAATACGTTCTTTAGCAAGTTCTACTTCATCGTTTACCACAACGTAGTCATA
AAGATTCATCATTTCCACTTCTTTACGTGCTTCTTTCACACGACTTTGAATCTTTTCGTC
TGATTCTGTACCGCGGCCGACTAAACGCTCTCTTAAGTGGTCTAAACTTGGCGGTGCTAG
GAAGATAAATAATGCATCAGGGAATTTCTTGCGTACTTGTTTCGCACCTTCAACTTCGAT
TTCTAAGAAGACATCATAGCCTTCATCCATAGTGTCTTTCACATACTGTACAGGTGTACC
GTAGTAGTTACCTACATATTCTGCATACTCAATAAATTGATCTTGTGCGATAAGTTGCTC
AAACTCTCGTTTTGTCTTGAAAAAATAATCAACGCCATCTTGTTCACCTTCACGCATTTC
ACGTGTCGTCATTGAAATTGAATATTTGTATGATGTTTTCGGATCATCAAATATTTCTTT
GCGGACAGTACCTTTTCCGACTCCAGATGGACCTGATAAGACAATTAGTAAACCTTTTTC
ATTATCCATGCCTTACGTTTAAACCTTGTAAAACAGGTCCGATTGCTTCATATCCGCCTA
AACGTTGCGCAATTTTGTAACCGATGTTACCTGCTTCTAAGCTTGGGAATACGAATACAT

TTGCGTCACCTTTAATTTGTGCATCTGGTGCTTTTTAGCCGCCACTTCTGGAACGATTG
CTGCGTCGAATTGGAATTCACCATCGATAACAACATCTTTTAAGCCTTCTGATTCAGCTT
TTTCTTGTGCAGCTTTTGTTGCTTCCGCTACTTTTTCAACATCGTCTGATTTTGCTGAAC
CTTTTGTTGAGAAGCTTAATAATGCTACTTTAGGATCCATGCCGAAGCTTTTCGCTGTTT
TTGCACTTTCTACCGCAATTTCAGCTAAGTCTGTTGCTTCTAATGTAGGGTTGATTGCAC
AATCACCGAAAACATATTGTTCTTCGCCTTTAATCATGAAGAACACACCTGATGTTTTCG
CAACACCTGGTTTAGTTTTGATGATTTGTAGTGCTGGACGAACTGTATCGCCTGTTGAGT
GTGCAGCACCGCTGACTAGTCCTTCAGCTTTACCTGCATAAACAAGCATTGTACCGAAGT
AGTTTACATTGTTAAGCATATCTTCAGCTTGTTCTTTTGTCGCTTTACCTTTACGACGTT
CTACAAATGCGTCTACTAATTCTGGTTTCAAGTCGCGACTAAAACTTCTTCGTCTTTCGC
AGTTGGAATATTTTTACCTACGAAGTCAGCGCGTATTGGTAATTCGCGGTGTCCGCGATC
CACTAATGTTGCTAACCCGATTTTTTGTGGTCGAGAATGTAGTAAGATTGCATCGAGTGA
CGCACGTACAGTTCGACCTGTATAAAGCACATCATCAATGATGATAACGACTTTGTCTTT
AAGGTCTACATCAATGACATAAGACTTCTCAGACATTTTAGGTGTCGGTAATTCTAAGTC
ATCTCTGAATTGCGTGATGTCTAATGTTCCTGTAGGAACACTTACGTCTTCAATTTGTTG
AATCTTCGTTTGAATACGTTTCGCTAAAAATTCTCCGCGTGTTTTCACACCAAGCAACAC
AAGATTTTCAGTACCTTTGTTATACTCTAAAATTTCATGTGCGATACGTGTTAAAGTCCG
CTGCATGCCTGCATCATCTAATACAATACGCGACTTGGCAAAGTGAAGAAACTTTTAAGC
CAGCTTCTTCTAATCGTTTACGGCCTTGTTGGAAACTTTTCTCTACTACAATACCGATAC
CGACTGTTTCTGCACCTGCTTGTTCAACTAAACGGTTCAAACCAAGTGCCGCATCTCCAT
TCGCTAAAAAGTCATCGATAATCAATACACGGTCATTTTCCCCTAAAAATTCTTCAGAGA
TAATGACGCGATTCGTCTTGTTTTTCGTAAACGAATGAATATCTGTCTGATAAAATCCAT
CTGCCAAAGTACTTGGCTTAGCCTTCTTCGCAAAAAGACATGGTACTTCAAAACGATATG
CTGCCATAATAGCTGGCGCAATACCTGATGCTTCAACAGTTAAGATTTTAGTGATGCCTT
GATCTTTGAACTGTTCATAAAATGTTTCGCCAATGTCATACATTAACACTGCATCAATTT
GATGGTTTAAAAACCCATCTACCTTCAAGATTTTTTCGCCAATGACGACGCCGTCTTCTT
TAACCTTCTACCTACTAGTGAACCTACAACACGAATACCGTCTAATACCAAACGTGGAAT
TGATAAGTCCATAGATTCTGTTGGCAAGCCGACTGCGACTACAGTACCGCCAGCACGTAC
AGATTCAACAGCTGCGTTGAAGGCTTGTTTAGCAACCGCTGTAACAACTGCCGCATGTGC
ACCGTCTACATGTTCTTGAATGTATTTTGCGACATCCACTTCTTTAGGGTTTAAAGTGAC
ATCTGCATCGTATTGTTTCGCAAATTCAAGTTGACTGTCATTGATGTCTACTGCGATAAC
TTTAGCGTTAAATACTTTTTTCGCATATTGTAAAGCTAAGTTTCCAAGTCCACCTAAACC
GAATAAGACAAGCCATTCACCAGCACGTACGCCTGATTCTTTAATCGCTTTGTATGTTGT
GACACCTGCACATGTGATACTGCTGGCAGCTTCTGATGATAAACCATCTGGTTTTTCCAA
CCTTTATATAATTTTTCTCTTTCTTTTTCATCCATTTCAGGCTCGAAAGCTTGATCTAAT
TTCCAACGGTTCGCAATTTCTGCTTTACTATCCCAGAAACCTACCGCAAGTCCTGCAAGA
TAAGCAGCGCCTAACGCAGTCGTTTCATTGATTTCAGGACGTTCTACTGTAATATTTTGA
ATGTCTGCTTGGAACTGCATTAAGAAGTTGTTCTTAACAGCACCACCATCTACACGGATA
TTCTTCACTTCAATACCTGAGTCTTGAATCATCGCTTCAACCACATCACGTGTTTGGTAA
CAAATAGATTCTAACGTTGCTCTGATAAAGTGTTCTTTCTCAGTCGCACGCGTAATACCA
AATACTGCACCACGCGCATTTGCATCCCAATATGGTGTACCGAGTCCAACAAACGCCGGT
ACCACATAAACACCATCACTTGATTCAATACGGTTCGCATAGTTTTCAGATTCAGGTGCT
GAGTTAATCATACGTAAACCATCTCGCAACCATTGAATAGCTGAACCAGAGACGAAAATA
GAACCTTCTAATGCATAATTCACTTCGCCATCTAAGCCGAATGCGATTGTTGTTAATAAA
CCGTTATCAGATTGAATCGCTTCTTTACCAGTGTTCATTAACATGAATCCACCTGTACCG
TACGTGTTTTTCACTTCACCACGTTCGAAGCATGCTTGTCCGAATAAGGCCGCTTGCTGA
TCCCCTGCGATTCCCGCAATCGGTACTTCTTGTCCAAAGAAATGATAATCTTTTGTTTTC
GCATAAATCTCACTTTTCTTTCGCATGGTTAATCGCTTCAATTGTTTGAGGCATAACACC
GTCGTCTGCTGCAACCACTAAGATTGTGATATCAGTGACTTGCGCACCACGTGCACGCAT
AGTAGTGAAGGCTGCGTGTCCAGGTGTATCTAAGAAAGTGATTTTTTTGCCGTCATTTGT
GATTTGGTAAGCACCGATATGTTGTGTGATACCGCCCGCTTCACCTTGTGTCACATGTGT
ATTTCTGATTGAGTCAAGTAGCGTTGTTTTACCATGGTCAACGTGTCCCATGATTGTTAC
AACTGCTGGACGCTCAATTGCATCCGGATCTTCTTCTTCATCATCGAAGTAGATATCTAA
GTCTTCTTCGTCAATGACTACTTCTTTTTCTACTTCTACACCATAATCATCAGCGATTAA
CTCAATTGTTTCATCGTCTAATGATTGGTTAATGTTTGCCATAATACCAAGTAAGAATAA
TTTCTTAACGATACTTGATGACTCAATATTCATTTTGTCAGCTAATTCGCCAACCGTAAT
ACCTTCTAAATAAGTAATTTTTTCTGGCATTTCTTTTTTCGGTTGTTCTGGTGCTGGTTT

TTGATTATTGTTTTTCTTATGTTTGTTGTTGT
>NAS-2
GATTGCTTGAATACGTTCTTTAGCAAGTTCTACTTCATCGTTTACCACAACGTAGTCATA
AAGATTCATCATTTCCACTTCTTTACGTGCTTCTTTCACACGACTTTGAATCTTTTCGTC
TGATTCTGTACCGCGGCCGACTAAACGCTCTCTTAAGTGGTCTAAACTTGGCGGTGCTAG
GAAGATAAATAATGCATCAGGGAATTTCTTGCGTACTTGTTTCGCACCTTCAACTTCGAT
TTCTAAGAAGACATCATAGCCTTCATCCATAGTGTCTTTCACATACTGTACAGGTGTACC
GTAGTAGTTACCTACATATTCTGCATACTCAATAAATTGATCTTGTGCGATAAGTTGCTC
AAACTCTCGTTTTGTCTTGAAAAAATAATCAACGCCATCTTGTTCACCTTCACGCATTTC
ACGTGTCGTCATTGAAATTGAATATTTGTATGATGTTTTCGGATCATCAAATATTTCTTT
GCGGACAGTACCTTTTCCGACTCCAGATGGACCTGATAAGACAATTAGTAAACCTTTTTC
ATTATCCATGCCTTACGTTTAAACCTTGTAAAACAGGTCCGATTGCTTCATATCCGCCTA
AACGTTGCGCAATTTTGTAACCGATGTTACCTGCTTCTAAGCTTGGGAATACGAATACAT
TTGCGTCACCTTTAATTTGTGCATCTGGTGCTTTTTTAGCCGCCACTTCTGGAACGATTG
CTGCGTCGAATTGGAATTCACCATCGATAACAACATCTTTTAAGCCTTCTGATTCAGCTT
TTTCTTGTGCAGCTTTTGTTGCTTCCGCTACTTTTTCAACATCGTCTGATTTTGCTGAAC
CTTTTGTTGAGAAGCTTAATAATGCTACTTTAGGATCCATGCCGAAGCTTTTCGCTGTTT
TTGCACTTTCTACCGCAATTTCAGCTAAGTCTGTTGCTTCTAATGTAGGGTTGATTGCAC
AATCACCGAAAACATATTGTTCTTCGCCTTTAATCATGAAGAACACACCTGATGTTTTCG
CAACACCTGGTTTAGTTTTGATGATTTGTAGTGCTGGACGAACTGTATCGCCTGTTGAGT
GTGCAGCACCGCTGACTAGTCCTTCAGCTTTACCTGCATAAACAAGCATTGTACCGAAGT
AGTTTACATTGTTAAGCATATCTTCAGCTTGTTCTTTTGTCGCTTTGCCTTTACGACGTT
CTACAAATGCGTCTACTAATTCTGGTTTCAAGTCGCGACTAAAACTTCTTCGTCTTTCGC
AGTTGGAATATTTTTACCTACAAAGTCAGCGCGTATTGGTAATTCGCGGTGTCCGCGATC
CACTAATGTTGCTAACCCGATTTTTTGTGGTCGAGAATGTAGTAAGATTGCATCGAGTGA
CGCACGTACAGTTCGACCCGTATAAAGCACATCATCAATGATGATAACGACTTTGTCTTT
AAGGTCTACATCAATGACATAAGACTTCTCAGACATTTTAGGTGTCGGTAACTCTAAATC
ATCTCTGAATTGCGTGATGTCTAATGTTCCTGTAGGAACACTTACGTCTTCAATTTGTTG
AATCTTCATTTGAATACGTTTCGCTAAAAATTCTCCGCGTGTTTTCACACCAAGCAACAC
AAGATTTTCAGTACCTTTGTTATACTCTAGAATTTCATGTGCGATACGTGTTAAAGTCCG
CTGCATGCCTGCATCATCTAATACAATACGCGACTTGGCAAAGCGAAGAAACTTTTAAGC
CAGCTTCTTCTAATCGTTTACGGCCTTGTTGGAAACTTTTTTCCACGACAATACCGATAC
CGACTGTTTCTGCACCTGCTTGTTCAACTAAACGATTCAAACCAAGTGCCGCATCTCCAT
TCGCTAAAAAGTCATCGATAATCAATACACGGTCATTTTCCCCTAAAAATTCTTCAGAGA
TAATGACGCGATTCGTCTTGTTTTTCGTAAACGAATGAATATCTGTCTGATAAAATCCAT
CTGCCAAAGTACTTGGCTTAGCCTTCTTCGCAAAGAGACATGGTACTTCAAAACGATATG
CTGCCATAATAGCTGGCGCAATGCCTGATGCTTCAACAGTTAAGATTTTAGTGATGCCTT
GATCTTTGAACTGTTCATAAAATGTTTCGCCAATGTCATACATTAACACTGCATCAATTT
GATGGTTTAAAAACCCATCTACCTTCAAGATTTTTTCGCCAATGACGACGCCGTCTTCTT
TAACCTTCTACCTACTAGTGAACCTACAACACGAATACCGTCTAATACTAAACGTGGAAT
TGATAAGTCCATTGATTCTGTTGGTAAGCCGACTGCGACTACAGTACCGCCAGCACGTAC
AGATTCAACAGCTGCGTTGAAGGCTTGTTTAGCAACCGCTGTAACAACTGCCGCATGTGC
ACCGCCTACATGTTCTTGAATGTATTTTGCGACATCCACTTCTTTAGGGTTTAAAGTGAC
ATCTGCATCGTATTGTTTCGCAAATTCAAGTTGACTGTCATTGATGTCTACTGCGATAAC
TTTAGCGTTAAATACTTTTTTCGCATATTGTAAAGCTAGGTTTCCAAGTCCACCTAAACC
GAATAAAACAAGCCATTCACCAGCACGTACGCCTGATTCTTTAATTGCTTTGTATGTTGT
GACACCTGCACATGTGATACTGCTGGCAGCTTCTGATGATAAACCATCTGGTTTTTCCAA
CCTTTATATAATTTTTCTCTTTCTTTTTCATCCATTTCAGGCTCGAAAGCTTGATCTAAT
TTCCAACGGTTCGCAATTTCTGCTTTACTATCCCAGAAACCTACCGCAAGTCCTGCAAGA
TAAGCAGCACCTAACGCAGTCGTTTCATTGATTTCAGGACGTTCTACTGTAATATTTTGA
ATATCTGCTTGGAACTGCATTAAGAAGTTGTTCTTAACAGCACCACCATCTACACGGATA
TTCTTCACTTCAATACCTGAGTCTTGAATCATCGCTTCAACCACATCACGTGTTTGGTAA
CAAATAGATTCTAACGTTGCTCTGATAAAGTGTTCTTTCTCAGTCGCACGCGTAATACCA
AATACTGCACCACGCGCATTCGCATCCCAATATGGTGTACCGAGTCCAACAAACGCCGGT
ACCACATAAACACCATCACTTGATTCAATACGGTTCGCATAGTTTTCAGATTCGGGTGCT
GAGTTAATCATACGTAAACCATCTCGCAACCATTGAATAGCTGAACCAGAGACGAAAATA
GAACCTTCTAATGCATAATTCACTTCGCCATCTAAGCCGAATGCGATTGTTGTTAATAAA

CCGTTATCCGATTGAATCGCTTCTTTACCAGTGTTCATTAACATGAATCCACCCGTACCA
TATGTGTTTTTCACTTCACCACGTTCGAAGCATGCTTGTCCGAATAAGGCTGCTTGCTGA
TCCCCTGCGATTCCCGCAATCGGTACTTCTTGTCCAAAGAAATGATAATCTTTTGTTTTC
GCATAAACCTCACTTTTCTTTTGCATGGTTAATCGCTTCAATTGTTTGAGGCATAACACC
GTCGTCTGCTGCAACCACTAAGATTGTGATATCAGTGACTTGCGCACCACGTGCACGCAT
AGTAGTGAAGGCTGCGTGTCCAGGTGTATCTAAGAAAGTGATTTTTTTGCCGTCATTTGT
GATTTGGTAAGCACCGATATGTTGTGTGATACCGCCCGCTTCACCTTGTGTCACATGTGT
ATTTCTGATTGAGTCAAGTAGCGTTGTTTTACCATGGTCAACGTGTCCCATGATTGTTAC
AACTGCTGGACGCTCAATTGCATCCGGATCTTCTTCTTCATCATCGAAGTAGATATCTAA
GTCTTCTTCGTCAATGACTACTTCTTTTTCTACTTCTACACCATAATCATCAGCGATTAA
CTCAATTGTTTCATCGTCTAATGATTGGTTAATGTTTGCCATAATACCAAGTAAGAATAA
TTTCTTAACAATACTTGATGACTCAATATTCATTTTGTCAGCTAATTCGCCAACCGTAAT
ACCTTCTAAATAAGTAATTTTTTCTGGCATTTCTTTTTTCGGTTGTTCTGGTGCTGGTTT
TTGATTATTGTTTTTCTTATGTTTGTTGTTGT
>NZ_CP015642
GATTGCTTGAATACGTTCTTTAGCAAGTTCTACTTCATCGTTTACCACAACGTAGTCATA
AAGATTCATCATTTCCACTTCTTTACGTGCTTCTTTCACACGACTTTGAATCTTTTCGTC
TGATTCTGTACCGCGGCCGACTAAACGCTCTCTTAAGTGGTCTAAACTTGGCGGTGCTAG
GAAGATAAATAATGCATCAGGGAATTTCTTGCGTACTTGTTTCGCACCTTCAACTTCGAT
TTCTAAGAAGACATCATAGCCTTCATCCATAGTGTCTTTCACATACTGTACAGGTGTACC
GTAGTAGTTACCTACATATTCTGCATACTCAATAAATTGATCTTGTGCGATAAGTTGCTC
AAACTCTCGTTTTGTCTTGAAAAAATAATCAACGCCATCTTGTTCACCTTCACGCATTTC
ACGTGTCGTCATTGAAATTGAATATTTGTATGATGTTTTCGGATCATCAAATATTTCTTT
GCGGACAGTACCTTTTCCGACTCCAGATGGACCTGATAAGACAATTAGTAAACCTTTTTC
ATTATCCATGCCTTACGTTTAAACCTTGTAAAACAGGTCCGATTGCTTCATATCCGCCTA
AACGTTGCGCAATTTTGTAACCGATGTTACCTGCTTCTAAGCTTGGGAATACGAATACAT
TTGCGTCACCTTTAATTTGTGCATCCGGTGCTTTTTTAGCCGCCACTTCTGGAACGATTG
CTGCGTCGAATTGGAATTCACCATCGATAACAACATCTTTTAAGCCTTCTGATTCAGCTT
TTTCTTGTGCAGCTTTTGTTGCTTCCGCTACTTTTTCAACATCGTCTGATTTTGCTGAAC
CTTTTGTTGAGAAGCTTAATAATGCTACTTTAGGATCCATGCCGAAGCTTTTCGCTGTTT
TTGCACTTTCTACCGCAATTTCAGCTAAGTCTGTTGCTTCTAATGTAGGGTTGATTGCAC
AATCACCGAAAACATATTGTTCTTCGCCTTTAATCATGAAGAACACACCTGATGTTTTCG
CAACACCTGGTTTAGTTTTGATGATTTGTAGTGCTGGACGAACTGTATCGCCTGTTGAAT
GTGCAGCACCGCTGACTAGTCCTTCAGCTTTACCTGCATAAACAAGCATTGTACCGAAGT
AGTTTACATTGTTAAGCATATCTTCAGCTTGTTCTTTTGTCGCTTTGCCTTTACGACGTT
CTACAAATGCGTCTACTAATTCTGGTTTCAAGTCGCGACTAAAACTTCTTCGTCTTTCGC
AGTTGGAATATTTTTACCTACGAAGTCAGCGCGTATTGGTAATTCGCGGTGTCCGCGATC
CACTAATGTTGCTAACCCGATTTTTTGTGGTCGAGAATGTAGTAAGATTGCATCGAGTGA
CGCACGTACAGTTCGACCTGTATAAAGCACATCATCAATGATGATAACGACTTTGTCTTT
AAGGTCTACATCAATGACATAAGACTTCTCAGACATTTTAGGTGTCGGTAACTCTAAGTC
ATCTCTGAATTGCGTGATGTCTAATGTTCCTGTAGGAACACTTACGTCTTCAATTTGTTG
AATCTTCGTTTGAATACGTTTCGCTAAAAATTCTCCGCGTGTTTTCACACCAAGCAACAC
AAGATTTTCAGTACCTTTGTTATACTCTAGAATTTCATGTGCGATACGTGTTAAAGTCCG
CTGCATGCCTGCATCATCTAATACAATACGCGACTTGGCAAAGTGAAGAAACTTTTAAGC
CAGCTTCTTCTAATCTTTTACGGCCTTGTTGGAAACTTTTCTCTACTACAATACCGATAC
CTACTGTTTCTGCACCTGCTTGTTCAACTAAACGGTTCAAACCAAGTGCCGCATCTCCAT
TCGCTAAAAAGTCATCGATAATCAATACACGGTCATTTTCCCCTAAAAATTCTTCAGAGA
TAATGACGCGATTCGTCTTGTTTTTCGTAAACGAATGAATATCTGTCTGATAAAATCCAT
CTGCCAAAGTACTTGGCTTAGCCTTCTTCGCAAAGAGACATGGTACTTCAAAACGATATG
CTGCCATAATAGCTGGCGCAATGCCTGATGCTTCAACAGTTAAGATTTTAGTGATGCCTT
GATCTTTGAACTGTTCATAAAATGTTTCGCCAATGTCATACATTAACACTGCATCAATTT
GATGGTTTAAAAACCCATCTACCTTCAAGATTTTTTCGCCAATGACGACGCCGTCTTCTT
TAACCTTCTACCTACTAGTGAACCTACAACACGAATACCGTCTAATACTAAACGTGGAAT
TGATAAGTCCATAGATTCTGTTGGTAAGCCGACTGCGACTACAGTACCGCCAGCACGTAC
AGATTCAACAGCTGCGTTGAAGGCTTGTTTAGCAACCGCTGTAACAACTGCCGCATGTGT
ACCGTCTACATGTTCTTGAATGTATTTTACGACATCCACTTCTTTAGGGTTTAAAGTGAC
ATCTGCATCGTATTGTTTCGCAAATTCAAGTTGACTGTCATTGATATCGACTGCGATAAC

TTTCGCATTAAATACTTTTTTCGCATATTGTAAAGCTAGGTTTCCAAGTCCACCTAAACC
GAATAAGACAAGCCATTCACCAGCACGTACGCCTGATTCTTTAATTGCTTTGTATGTTGT
CACGCCTGCACAAGTGATACTGCTGGCAGCTTCTGATGATAAACCATCTGGTTTTTCCAA
CCTTTATATAATTTTTCTCTTTCTTTTTCATCCATTTCAGGTTCGAAAGCTTGATCTAAT
TTCCAACGGTTCGCAATTTCTGCTTTACTATCCCAGAAACCTACCGCAAGTCCTGCAAGA
TAAGCAGCACCTAACGCAGTCGTTTCATTGATTTCAGGACGTTCTACTGTAATGTTTTGA
ATATCTGCTTGGAACTGCATTAAGAAGTTGTTCTTAACAGCACCACCATCTACACGGATA
TTCTTCACTTCAATACCTGAGTCTTGAATCATCGCTTCAACCACATCACGTGTTTGGTAA
CAAATAGATTCTAACGTTGCTCTGATAAAGTGTTCTTTCTCAGTCGCACGCGTAATACCG
AATACTGCACCACGCGCATTCGCATCCCAATAAGGTGTACCGAGTCCAACAAATGCAGGC
ACCACATAAACGCCATCACTTGATTCAATACGGTTCGCATAGTTTTCAGATTCAGGTGCG
GAGTTAATCATACGTAAACCATCTCGCAACCATTGAATAGCTGAGCCAGAGACGAAAATA
GAACCTTCTAATGCATAATTCACTTCGCCATCTAAGCCGAATGCGATTGTTGTTAATAAA
CCGTTATCAGATTGAATCGCTTCTTTACCAGTGTTCATTAACATGAATCCACCTGTACCG
TACGTGTTTTTCACTTCACCACGTTCGAAGCATGCTTGTCCGAATAAGGCTGCTTGCTGA
TCCCCTGCGATTCCCGCAATCGGTACTTCTTGTCCGAAGAAATGATAATCTTTTGTTTTC
GCATAAATCTCACTTTTCTTTTGCATGGTTAATCGCTTCAATTGTTTGAGGCATAACACC
GTCGTCTGCTGCAACCACTAAGATTGTGATATCAGTGACTTGCGCACCACGTGCACGCAT
AGTAGTGAAGGCTGCGTGTCCAGGTGTATCTAAGAAAGTGATTTTTTTGCCGTCATTTGT
GATTTGGTAAGCACCGATATGTTGTGTGATACCGCCCGCTTCACCTTGTGTCACATGTGT
ATTTCTGATTGAGTCAAGTAGCGTTGTTTTACCATGGTCAACGTGTCCCATGATTGTTAC
AACTGCTGGACGCTCAATTGCATCCGGATCTTCTTCTTCATCATCGAAGTAGATATCTAA
GTCTTCTTCGTCAATGACTACTTCTTTTTCTACTTCTACACCATAATCATCAGCGATTAA
CTCAATTGTTTCATCGTCTAATGATTGGTTAATGTTTGCCATAATACCAAGTAAGAATAA
TTTCTTAACAATACTTGATGACTCAATATTCATTTTGTCAGCTAATTCGCCAACCGTAAT
ACCTTCTAAATAAGTAATTTTTTCTGGCATTTCTTTTTTCGGTTGTTCTGGTGCTGGTTT
TTGATTATTGTTTTTCTTATGTTTGTTGTTGT
>NZ_CP017428
GATTGCTTGAATACGTTCTTTAGCAAGTTCTACTTCATCGTTTACCACAACGTAGTCATA
AAGATTCATCATTTCCACTTCTTTACGTGCTTCTTTCACACGACTTTGAATCTTTTCGTC
TGATTCTGTACCGCGGCCGACTAAACGCTCTCTTAAGTGGTCTAAACTTGGCGGTGCTAG
GAAGATAAATAATGCATCAGGGAATTTCTTGCGTACTTGTTTCGCACCTTCAACTTCGAT
TTCTAAGAAGACATCATAGCCTTCATCCATAGTGTCTTTCACATACTGTACAGGTGTACC
GTAGTAGTTACCTACATATTCTGCATACTCAATAAATTGATCTTGTGCGATAAGTTGCTC
AAACTCTCGTTTTGTCTTGAAAAAATAATCAACGCCATCTTGTTCACCTTCACGCATTTC
ACGTGTCGTCATTGAAATTGAATATTTGTATGATGTTTTCGGATCATCAAATATTTCTTT
GCGGACAGTACCTTTTCCGACTCCAGATGGACCTGATAAGACAATTAGTAAACCTTTTTC
ATTATCCATGCCTTACGTTTAAACCTTGTAAAACAGGTCCGATTGCTTCATATCCGCCTA
AACGTTGCGCAATTTTGTAACCGATGTTACCTGCTTCTAAGCTTGGGAATACGAATACAT
TTGCGTCACCTTTAATTTGTGCATCCGGTGCTTTTTTAGCCGCCACTTCTGGAACGATTG
CTGCGTCGAATTGGAATTCACCATCGATAACAACATCTTTTAAGCCTTCTGATTCAGCTT
TTTCTTGTGCAGCTTTTGTTGCTTCCGCTACTTTTTCAACATCGTCTGATTTTGCTGAAC
CTTTTGTTGAGAAGCTTAATAATGCTACTTTAGGATCCATGCCGAAGCTTTTCGCTGTTT
TTGCACTTTCTACCGCAATTTCAGCTAAGTCTGTTGCTTCTAATGTAGGGTTGATTGCAC
AATCACCGAAAACATATTGTTCTTCGCCTTTAATCATGAAGAACACACCTGATGTTTTCG
CAACACCTGGTTTAGTTTTGATGATTTGTAGTGCTGGACGAACTGTATCGCCTGTTGAAT
GTGCAGCACCGCTGACTAGTCCTTCAGCTTTACCTGCATAAACAAGCATTGTACCGAAGT
AGTTTACATTGTTAAGCATATCTTCAGCTTGTTCTTTTGTCGCTTTGCCTTTACGACGTT
CTACAAATGCGTCTACTAATTCTGGTTTCAAGTCGCGACTAAAACTTCTTCGTCTTTCGC
AGTTGGAATATTTTTACCTACGAAGTCAGCGCGTATTGGTAATTCGCGGTGTCCGCGATC
CACTAATGTTGCTAACCCGATTTTTTGTGGTCGAGAATGTAGTAAGATTGCATCGAGTGA
CGCACGTACAGTTCGACCTGTATAAAGCACATCATCAATGATGATAACGACTTTGTCTTT
AAGGTCTACATCAATGACATAAGACTTCTCAGACATTTTAGGTGTCGGTAACTCTAAGTC
ATCTCTGAATTGCGTGATGTCTAATGTTCCTGTAGGAACACTTACGTCTTCAATTTGTTG
AATCTTCGTTTGAATACGTTTCGCTAAAAATTCTCCGCGTGTTTTCACACCAAGCAACAC
AAGATTTTCAGTACCTTTGTTATACTCTAGAATTTCATGTGCGATACGTGTTAAAGTCCG
CTGCATGCCTGCATCATCTAATACAATACGCGACTTGGCAAAGTGAAGAAACTTTTAAGC

CAGCTTCTTCTAATCTTTTACGGCCTTGTTGGAAACTTTTCTCTACTACAATACCGATAC
CTACTGTTTCTGCACCTGCTTGTTCAACTAAACGGTTCAAACCAAGTGCCGCATCTCCAT
TCGCTAAAAAGTCATCGATAATCAATACACGGTCATTTTCCCCTAAAAATTCTTCAGAGA
TAATGACGCGATTCGTCTTGTTTTTCGTAAACGAATGAATATCTGTCTGATAAAATCCAT
CTGCCAAAGTACTTGGCTTAGCCTTCTTCGCAAAGAGACATGGTACTTCAAAACGATATG
CTGCCATAATAGCTGGCGCAATGCCTGATGCTTCAACAGTTAAGATTTTAGTGATGCCTT
GATCTTTGAACTGTTCATAAAATGTTTCGCCAATGTCATACATTAACACTGCATCAATTT
GATGGTTTAAAAACCCATCTACCTTCAAGATTTTTTCGCCAATGACGACGCCGTCTTCTT
TAACCTTCTACCTACTAGTGAACCTACAACACGAATACCGTCTAATACTAAACGTGGAAT
TGATAAGTCCATAGATTCTGTTGGTAAGCCGACTGCGACTACAGTACCGCCAGCACGTAC
AGATTCAACAGCTGCGTTGAAGGCTTGTTTAGCAACCGCTGTAACAACTGCCGCATGTGT
ACCGTCTACATGTTCTTGAATGTATTTTACGACATCCACTTCTTTAGGGTTTAAAGTGAC
ATCTGCATCGTATTGTTTCGCAAATTCAAGTTGACTGTCATTGATATCGACTGCGATAAC
TTTCGCATTAAATACTTTTTTCGCATATTGTAAAGCTAGGTTTCCAAGTCCACCTAAACC
GAATAAGACAAGCCATTCACCAGCACGTACGCCTGATTCTTTAATTGCTTTGTATGTTGT
CACGCCTGCACAAGTGATACTGCTGGCAGCTTCTGATGATAAACCATCTGGTTTTTCCAA
CCTTTATATAATTTTTCTCTTTCTTTTTCATCCATTTCAGGTTCGAAAGCTTGATCTAAT
TTCCAACGGTTCGCAATTTCTGCTTTACTATCCCAGAAACCTACCGCAAGTCCTGCAAGA
TAAGCAGCACCTAACGCAGTCGTTTCATTGATTTCAGGACGTTCTACTGTAATGTTTTGA
ATATCTGCTTGGAACTGCATTAAGAAGTTGTTCTTAACAGCACCACCATCTACACGGATA
TTCTTCACTTCAATACCTGAGTCTTGAATCATCGCTTCAACCACATCACGTGTTTGGTAA
CAAATAGATTCTAACGTTGCTCTGATAAAGTGTTCTTTCTCAGTCGCACGCGTAATACCG
AATACTGCACCACGCGCATTCGCATCCCAATAAGGTGTACCGAGTCCAACAAATGCAGGC
ACCACATAAACGCCATCACTTGATTCAATACGGTTCGCATAGTTTTCAGATTCAGGTGCG
GAGTTAATCATACGTAAACCATCTCGCAACCATTGAATAGCTGAGCCAGAGACGAAAATA
GAACCTTCTAATGCATAATTCACTTCGCCATCTAAGCCGAATGCGATTGTTGTTAATAAA
CCGTTATCAGATTGAATCGCTTCTTTACCAGTGTTCATTAACATGAATCCACCTGTACCG
TACGTGTTTTTCACTTCACCACGTTCGAAGCATGCTTGTCCGAATAAGGCTGCTTGCTGA
TCCCCTGCGATTCCCGCAATCGGTACTTCTTGTCCGAAGAAATGATAATCTTTTGTTTTC
GCATAAATCTCACTTTTCTTTTGCATGGTTAATCGCTTCAATTGTTTGAGGCATAACACC
GTCGTCTGCTGCAACCACTAAGATTGTGATATCAGTGACTTGCGCACCACGTGCACGCAT
AGTAGTGAAGGCTGCGTGTCCAGGTGTATCTAAGAAAGTGATTTTTTTGCCGTCATTTGT
GATTTGGTAAGCACCGATATGTTGTGTGATACCGCCCGCTTCACCTTGTGTCACATGTGT
ATTTCTGATTGAGTCAAGTAGCGTTGTTTTACCATGGTCAACGTGTCCCATGATTGTTAC
AACTGCTGGACGCTCAATTGCATCCGGATCTTCTTCTTCATCATCGAAGTAGATATCTAA
GTCTTCTTCGTCAATGACTACTTCTTTTTCTACTTCTACACCATAATCATCAGCGATTAA
CTCAATTGTTTCATCGTCTAATGATTGGTTAATGTTTGCCATAATACCAAGTAAGAATAA
TTTCTTAACAATACTTGATGACTCAATATTCATTTTGTCAGCTAATTCGCCAACCGTAAT
ACCTTCTAAATAAGTAATTTTTTCTGGCATTTCTTTTTTCGGTTGTTCTGGTGCTGGTTT
TTGATTATTGTTTTTCTTATGTTTGTTGTTGT
>NZ_LT963435
GATTGCTTGAATACGTTCTTTAGCAAGTTCTACTTCATCGTTTACCACAACGTAGTCATA
AAGATTCATCATTTCCACTTCTTTACGTGCTTCTTTCACACGACTTTGAATCTTTTCGTC
TGATTCTGTACCGCGGCCGACTAAACGCTCTCTTAAGTGGTCTAAACTTGGCGGTGCTAG
GAAGATAAATAATGCATCAGGGAATTTCTTGCGTACTTGTTTCGCACCTTCAACTTCGAT
TTCTAAGAAGACATCATAGCCTTCATCCATAGTGTCTTTCACATACTGTACAGGTGTACC
GTAGTAGTTACCTACATATTCTGCATACTCAATAAATTGATCTTGTGCGATAAGTTGCTC
AAACTCTCGTTTTGTCTTGAAAAAATAATCAACGCCATCTTGTTCACCTTCACGCATTTC
ACGTGTCGTCATTGAAATTGAATATTTGTATGATGTTTTCGGATCATCAAATATTTCTTT
GCGGACAGTACCTTTTCCGACTCCAGATGGACCTGATAAGACAATTAGTAAACCTTTTTC
ATTATCCATGCCTTACGTTTAAACCTTGTAAAACAGGTCCGATTGCTTCATATCCGCCTA
AACGTTGCGCAATTTTGTAACCGATGTTACCTGCTTCTAAGCTTGGGAATACGAATACAT
TTGCGTCACCTTTAATTTGTGCATCTGGTGCTTTTTTAGCCGCCACTTCTGGAACGATTG
CTGCGTCGAATTGGAATTCACCATCGATAACAACATCTTTTAAGCCTTCTGATTCAGCTT
TTTCTTGTGCAGCTTTTGTTGCTTCCGCTACTTTTTCAACATCGTCTGATTTTGCTGAAC
CTTTTGTTGAGAAGCTTAATAATGCTACTTTAGGATCCATGCCGAAGCTTTTCGCTGTTT
TTGCACTTTCTACCGCAATTTCAGCTAAGTCTGTTGCTTCTAATGTAGGGTTGATTGCAC

AATCACCGAAAACATATTGTTCTTCGCCTTTAATCATGAAGAACACACCTGATGTTTTCG
CAACACCTGGTTTAGTTTTGATGATTTGTAGTGCTGGACGAACTGTATCGCCTGTTGAGT
GTGCAGCACCGCTGACTAGTCCTTCAGCTTTACCTGCATAAACAAGCATTGTACCGAAGT
AGTTTACATTGTTAAGCATATCTTCAGCTTGTTCTTTTGTCGCTTTACCTTTACGACGTT
CTACAAATGCGTCTACTAATTCTGGTTTCAAGTCGCGACTAAAACTTCTTCGTCTTTCGC
AGTTGGAATATTTTTACCTACGAAGTCAGCGCGTATTGGTAATTCGCGGTGTCCGCGATC
CACTAATGTTGCTAACCCGATTTTTTGTGGTCGAGAATGTAGTAAGATTGCATCGAGTGA
CGCACGTACAGTTCGACCTGTATAAAGCACATCATCAATGATGATAACGACTTTGTCTTT
AAGGTCTACATCAATGACATAAGACTTCTCAGACATTTTAGGTGTCGGTAACTCTAAGTC
ATCTCTGAATTGCGTGATGTCTAATGTTCCTGTAGGAACACTTACGTCTTCAATTTGTTG
AATCTTCGTTTGAATACGTTTCGCTAAAAATTCTCCGCGTGTTTTCACACCAAGCAACAC
AAGATTTTCAGTACCTTTGTTATACTCTAAAATTTCATGTGCGATACGTGTTAAAGTCCG
CTGCATGCCTGCATCATCTAATACAATACGCGACTTGGCAAAGCGAAGAAACTTTTAAGC
CAGCTTCTTCTAATCGTTTACGGCCTTGTTGGAAACTTTTTTCCACGACAATACCGATAC
CGACTGTTTCTGCACCTGCTTGTTCAACTAAACGATTCAAACCAAGTGCCGCATCTCCAT
TCGCTAAAAAGTCATCGATAATCAATACACGATCATTTTCTCCTAAAAATTCTTCAGAGA
TAATGACACGATTCGTCTTGTTTTTCGTAAACGAATGAATATCTGTCTGATAAAATCCAT
CTGCCAAAGTACTTGGCTTAGCCTTCTTCGCAAAGAGACATGGTACTTCAAAACGATATG
CTGCCATAATAGCTGGCGCAATGCCTGATGCTTCAACAGTTAAGATTTTAGTGATGCCTT
GATCTTTAAACTGTTCATAAAATGTTTCGCCAATGTCATACATTAACACTGCATCAATTT
GATGGTTTAAAAACCCATCTACCTTCAAGATTTTTTCGCCAATGACGACGCCGTCTTCTT
TAACCTTCTACCTACTAGTGAACCTACAACACGAATACCGTCTAATACCAAACGTGGAAT
TGATAAGTCCATAGATTCTGTTGGTAAGCCGACTGCGACTACAGTACCGCCAGCACGTAC
AGATTCAACAGCTGCGTTGAAGGCTTGTTTAGCAACCGCTGTAACAACTGCCGCATGTGC
ACCGTCTACATGTTCTTGAATGTATTTTGCGACATCCACTTCTTTAGGGTTTAAAGTAAC
ATCTGCATCGTATTGTTTCGCAAATTCAAGTTGACTGTCATTGATATCGACTGCGATAAC
TTTCGCATTAAATACTTTTTTGGCATATTGCAAAGCTAAGTTTCCAAGTCCACCTAAACC
GAATAAGACAAGCCATTCACCAGCACGTACGCCTGATTCTTTAATCGCTTTGTATGTTGT
GACACCTGCACATGTGATACTGCTGGCAGCTTCTGATGATAAACCATCTGGTTTTTCCAA
CCTTTATATAATTTTTCTCTTTCTTTTTCATCCATTTCAGGCTCGAAAGCTTGATCTAAT
TTCCAACGGTTCGCAATTTCTGCTTTACTATCCCAGAAACCTACCGCAAGTCCTGCAAGA
TAAGCAGCACCTAACGCAGTCGTTTCATTGATTTCAGGACGTTCTACTGTAATATTTTGA
ATATCTGCTTGGAACTGCATTAAGAAGTTGTTCTTAACAGCACCACCATCTACACGGATA
TTCTTCACTTCAATACCTGAGTCTTGAATCATCGCTTCAACCACATCACGTGTTTGGTAA
CAAATAGATTCTAACGTTGCTCTGATAAAGTGTTCTTTCTCAGTCGCACGTGTAATACCA
AATACTGCACCACGTGCATTCGCATCCCAATATGGTGTACCGAGTCCAACAAACGCCGGT
ACCACATAAACATCATCACTTGATTCAATACGGTTCGCATAGTTTTCAGATTCAGGTGCT
GAGTTAATCATACGTAAACCATCTCGCAACCATTGAATAGCTGAACCAGAGACGAAAATA
GAACCTTCTAATGCATAATTCACTTCGCCATCTAAGCCGAATGCGATTGTTGTTAATAAA
CCGTTATCCGATTGAATCGCTTCTTTACCAGTGTTCATTAACATGAATCCACCCGTACCA
TATGTGTTTTTCACTTCACCACGTTCGAAGCATGCTTGTCCGAATAAGGCTGCTTGCTGA
TCCCCTGCGATTCCCGCAATCGGTACTTCTTGTCCGAAGAAATGATAATCTTTTGTTTTC
GCATAAATCTCACTTTTCTTTAGCATGGTTAATCGCTTCAATTGTTTGAGGCATAACACC
GTCGTCTGCTGCAACCACTAAGATTGTGATATCAGTGACTTGCGCACCACGTGCACGCAT
AGTAGTGAAGGCTGCGTGTCCAGGTGTATCTAAGAAAGTGATTTTTTTGCCGTCATTTGT
GATTTGGTAAGCACCGATATGTTGTGTGATACCGCCCGCTTCACCTTGTGTCACATGTGT
ATTTCTGATTGAGTCAAGTAGCGTTGTTTTACCATGGTCAACGTGTCCCATGATTGTTAC
AACTGCTGGACGCTCAATTGCATCCGGATCTTCTTCTTCATCATCGAAGTAGATATCTAA
GTCTTCTTCGTCAATGACTACTTCTTTTTCTACTTCTACACCATAATCATCAGCGATTAA
CTCAATTGTTTCATCGTCTAATGATTGGTTAATGTTTGCCATAATACCAAGTAAGAATAA
TTTCTTAACAATACTTGATGACTCAATATTCATTTTGTCAGCTAATTCGCCAACCGTAAT
ACCTTCTAAATAAGTAATTTTTTCTGGCATTTCTTTTTTCGGTTGTTCTGGTGCTGGTTT
TTGATTATTGTTTTTCTTATGTTTGTTGTTGT
>NZ_CP014016
GATTGCTTGAATACGTTCTTTAGCAAGTTCTACTTCATCGTTTACCACAACGTAGTCATA
AAGATTCATCATTTCCACTTCTTTACGTGCTTCTTTCACACGACTTTGAATCTTTTCGTC
TGATTCTGTACCGCGGCCGACTAAACGCTCTCTTAAGTGGTCTAAACTTGGCGGTGCTAG

GAAGATAAATAATGCATCAGGGAATTTCTTGCGTACTTGTTTCGCACCTTCAACTTCGAT
TTCTAAGAAGACATCATAGCCTTCATCCATAGTGTCTTTCACATACTGTACAGGTGTACC
GTAGTAGTTACCTACATATTCTGCATACTCAATAAATTGATCTTGTGCGATAAGTTGCTC
AAACTCTCGTTTTGTCTTGAAAAAATAATCAACGCCATCTTGTTCACCTTCACGCATTTC
ACGTGTCGTCATTGAAATTGAATATTTGTATGATGTTTTCGGGTCATCAAATATTTCTTT
GCGGACAGTACCTTTTCCGACTCCAGATGGACCTGATAAGACAATTAGTAAACCTTTTTC
ATTATCCATGCCTTACGTTTAAACCTTGTAAAACAGGTCCGATTGCTTCATATCCGCCTA
AACGTTGCGCAATTTTGTAACCGATGTTACCTGCTTCTAAGCTTGGGAATACGAATACAT
TTGCGTCACCTTTAATTTGTGCATCTGGTGCTTTTTTAGCCGCCACTTCTGGAACGATTG
CTGCGTCGAATTGGAATTCACCATCGATAACAACATCTTTTAAGCCTTCTGATTCAGCTT
TTTCTTGTGCAGCTTTTGTTGCTTCCGCTACTTTTTCAACATCGTCTGATTTTGCTGAAC
CTTTTGTTGAGAAGCTTAATAATGCTACTTTAGGATCCATGCCGAAGCTTTTCGCTGTTT
TTGCACTTTCTACCGCAATTTCAGCTAAGTCTGTTGCTTCTAATGTAGGGTTGATTGCAC
AATCACCGAAAACATATTGTTCTTCGCCTTTAATCATGAAGAACACACCTGATGTTTTCG
CAACACCTGGTTTAGTTTTGATGATTTGTAGTGCTGGACGAACTGTATCGCCTGTTGAGT
GTGCAGCACCGCTGACTAGTCCTTCAGCTTTACCTGCATAAACAAGCATTGTACCGAAGT
AGTTTACATTGTTAAGCATATCTTCAGCTTGTTCTTTTGTCGCTTTACCTTTACGACGTT
CTACAAATGCGTCTACTAATTCTGGTTTCAAGTCGCGACTAAAACTTCTTCGTCTTTCGC
AGTTGGAATATTTTTACCTACGAAGTCAGCGCGTATTGGTAATTCGCGGTGTCCGCGATC
CACTAATGTTGCTAACCCGATTTTTTGTGGTCGAGAATGTAGTAAGATTGCATCGAGTGA
CGCACGTACAGTTCGACCTGTATAAAGCACATCATCAATGATGATAACGACTTTGTCTTT
AAGGTCTACATCAATGACATAAGATTTCTCAGACATTTTAGGTGTCGGTAACTCTAAATC
ATCTCTGAATTGCGTGATGTCTAATGTTCCTGTAGGAACACTTACGTCTTCAATTTGTTG
AATCTTCATTTGAATACGTTTCGCTAAAAATTCTCCGCGTGTTTTCACACCAAGCAACAC
AAGATTTTCAGTACCTTTGTTATACTCTAGAATTTCATGTGCGATACGTGTTAAAGTCCG
CTGCATGCCTGCATCATCTAATACAATACGCGACTTGGCAAAGTGAAGAAACTTTCAAGC
CAGCTTCTTCTAAACGTTTACGGCCTTGTTGGAAACTTTTCTCCACAACAATACCGATAC
CGACTGTTTCTGCACCTGCTTGTTCAACTAAACGATTCAAACCAAGTGCCGCATCTCCAT
TCGCTAAAAAGTCATCGATAATCAATACACGATCATTTTCTCCTAAAAATTCTTCAGAAA
TAATGACACGATTCGTCTTGTTTTTCGTAAACGAATGAATATCTGTCTGATAAAATCCAT
CTGCCAAAGTACTTGGCTTAGCCTTCTTCGCAAAGAGACATGGTACTTCAAAACGATATG
CTGCCATAATAGCTGGCGCAATACCTGATGCTTCAACAGTTAAGATTTAGTGATGCCTT
GATCTTTAAACTGTTCATAAAATGTTTCGCCAATGTCATACATTAACACTGCATCAATTT
GATGGTTTAAAAACCCATCTACCTTCAAGATTTTTTCGCCAATGACGACGCCGTCTTCTT
TAACCTTCTACCTACTAGTGAACCTACAACACGAATACCGTCTAATACCAAACGTGGAAT
TGATAAGTCCATAGATTCTGTTGGTAAGCCGACTGCGACTACAGTACCGCCAGCACGTAC
AGATTCAACAGCTGCGTTGAAGGCTTGTTTAGCAACCGCTGTAACAACTGCCGCATGTGC
ACCGTCTACATGTTCTTGAATGTATTTTGCGACATCCACTTCTTTAGGGTTTAAAGTAAC
ATCTGCATCGTATTGTTTCGCAAATTCAAGTTGACTGTCATTGATATCGACTGCGATAAC
TTTCGCATTAAATACTTTTTTGGCATATTGCAAAGCTAAGTTTCCAAGTCCACCTAAACC
GAATAAGACAAGCCATTCACCAGCACGTACGCCTGATTCTTTAATCGCTTTGTATGTTGT
GACACCTGCACATGTGATACTGCTGGCAGCTTCTGATGATAAACCATCTGGTTTTTCCAA
CCTTTATATAATTTTTCTCTTTCTTTTTCATCCATTTCAGGTTCGAAAGCTTGATCTAAT
TTCCAACGGTTCGCAATTTCTGCTTTACTATCCCAGAAACCTACCGCAAGTCCTGCAAGA
TAAGCAGCACCTAACGCAGTCGTTTCATTGATTTCAGGACGTTCTACTGTAATGTTTTGA
ATATCTGCTTGGAACTGCATTAAGAAGTTGTTCTTAACAGCACCACCATCTACACGGATA
TTCTTCACTTCAATACCTGAGTCTTGAATCATCGCTTCAACCACATCACGTGTTTGGTAA
CAAATAGATTCTAACGTTGCTCTGATAAAGTGTTCTTTCTCAGTCGCACGTGTAATACCA
AATACTGCACCACGCGCATTCGCATCCCAATATGGTGTACCGAGTCCAACAAATGCAGGC
ACCACATAAACGCCATCACTTGATTCAATACGGTTCGCATAGTTTTCAGATTCAGGTGCT
GAGTTAATCATACGTAAACCATCTCGCAACCATTGAATAGCTGAGCCAGAGACGAAAATA
GAACCTTCTAATGCATAATTCACTTCGCCATCTAAGCCGAATGCGATTGTTGTTAATAAA
CCGTTATCAGATTGAATCGCTTCTTTACCAGTGTTCATTAACATGAATCCACCTGTACCG
TACGTGTTTTTCACTTCACCACGTTCGAAGCATGCTTGTCCGAATAAGGCTGCTTGCTGA
TCCCCTGCGATTCCCGCAATCGGTACTTCTTGTCCGAAGAAATGATAATCTTTTGTTTTC
GCATAAATCTCACTTTTCTTTCGCATGGTTAATCGCTTCAATTGTTTGAGGCATAACACC
GTCGTCTGCTGCAACCACTAAGATTGTGATATCAGTGACTTGCGCACCACGTGCACGCAT

AGTAGTGAAGGCTGCGTGTCCAGGTGTATCTAAGAAAGTGATTTTTTTGCCGTCATTTGT
GATTTGGTAAGCACCGATATGTTGTGTGATACCGCCCGCTTCACCTTGTGTCACATGTGT
ATTTCTGATTGAGTCAAGTAGCGTTGTTTTACCATGGTCAACGTGTCCCATGATTGTTAC
AACTGCTGGACGCTCAATTGCATCCGGATCTTCTTCTTCATCATCGAAGTAGATGTCTAA
GTCTTCTTCGTCAATGACTACTTCTTTTTCTACTTCTACACCATAATCATCAGCGATTAA
CTCAATTGTTTCATCGTCTAATGATTGGTTAATGTTTGCCATAATACCAAGTAAGAATAA
TTTCTTAACAATACTTGATGACTCAATATTCATTTTGTCAGCTAATTCGCCAACCGTAAT
ACCTTCTAAATAAGTAATTTTTTCTGGCATTTCTTTTTTCGGTTGTTCTGGTGCTGGTTT
TTGATTATTGTTTTTCTTATGTTTGTTGTTGT
>NZ_LR134264
GATTGCTTGAATACGTTCTTTAGCAAGTTCTACTTCATCGTTTACCACAACGTAGTCATA
AAGATTCATCATTTCCACTTCTTTACGTGCTTCTTTCACACGACTTTGAATCTTTTCGTC
TGATTCTGTACCGCGGCCGACTAAACGCTCTCTTAAGTGGTCTAAACTTGGCGGTGCTAG
GAAGATAAATAATGCATCAGGGAATTTCTTGCGTACTTGTTTCGCACCTTCAACTTCGAT
TTCTAAGAAGACATCATAGCCTTCATCCATAGTGTCTTTCACATACTGTACAGGTGTACC
GTAGTAGTTACCTACATATTCTGCATACTCAATAAATTGATCTTGTGCGATAAGTTGCTC
AAACTCTCGTTTTGTCTTGAAAAAATAATCAACGCCATCTTGTTCACCTTCACGCATTTC
ACGTGTCGTCATTGAAATTGAATATTTGTATGATGTTTTCGGATCATCAAATATTTCTTT
GCGGACAGTACCTTTTCCGACTCCAGATGGACCTGATAAGACAATTAGTAAACCTTTTTC
ATTATCCATGCCTTACGTTTAAACCTTGTAAAACAGGTCCGATTGCTTCATATCCGCCTA
AACGTTGCGCAATTTTGTAACCGATGTTACCTGCTTCTAAGCTTGGGAATACGAATACAT
TTGCGTCACCTTTAATTTGTGCATCTGGTGCTTTTTTAGCCGCCACTTCTGGAACGATTG
CTGCGTCGAATTGGAATTCACCATCGATAACAACATCTTTTAAGCCTTCTGATTCAGCTT
TTTCTTGTGCAGCTTTTGTTGCTTCCGCTACTTTTTCAACATCGTCTGATTTTGCTGAAC
CTTTTGTTGAGAAGCTTAATAATGCTACTTTAGGATCCATGCCGAAGCTTTTCGCTGTTT
TTGCACTTTCTACCGCAATTTCAGCTAAGTCTGTTGCTTCTAATGTAGGGTTGATTGCAC
AATCACCGAAAACATATTGTTCTTCGCCTTTAATCATGAAGAACACACCTGATGTTTTCG
CAACACCTGGTTTAGTTTTGATGATTTGTAGTGCTGGACGAACTGTATCGCCTGTTGAGT
GTGCAGCACCGCTGACTAGTCCTTCAGCTTTACCTGCATAAACAAGCATTGTACCGAAGT
AGTTTACATTGTTAAGCATATCTTCAGCTTGTTCTTTTGTCGCTTTACCTTTACGACGTT
CTACAAATGCGTCTACTAATTCTGGTTTCAAGTCGCGACTAAAACTTCTTCGTCTTTCGC
AGTTGGAATATTTTTACCTACGAAGTCAGCGCGTATTGGTAATTCGCGGTGTCCGCGATC
CACTAATGTTGCTAACCCGATTTTTTGTGGTCGAGAATGTAGTAAGATTGCATCGAGTGA
CGCACGTACAGTTCGACCTGTATAAAGCACATCATCAATGATGATAACGACTTTGTCTTT
AAGGTCTACATCAATGACATAAGACTTCTCAGACATTTTAGGTGTCGGTAACTCTAAGTC
ATCTCTGAATTGCGTGATGTCTAATGTTCCTGTAGGAACACTTACGTCTTCAATTTGTTG
AATCTTCGTTTGAATACGTTTCGCTAAAAATTCTCCGCGTGTTTTCACACCAAGCAACAC
AAGATTTTCAGTACCTTTGTTATACTCTAAAATTTCATGTGCGATACGTGTTAAAGTCCG
CTGCATGCCTGCATCATCTAATACAATACGCGACTTGGCAAAGCGAAGAAACTTTTAAGC
CAGCTTCTTCTAATCGTTTACGGCCTTGTTGGAAACTTTTTTCCACGACAATACCGATAC
CGACTGTTTCTGCACCTGCTTGTTCAACTAAACGATTCAAACCAAGTGCCGCATCTCCAT
TCGCTAAAAAGTCATCGATAATCAATACACGATCATTTTCTCCTAAAAATTCTTCAGAGA
TAATGACACGATTCGTCTTGTTTTTCGTAAACGAATGAATATCTGTCTGATAAAATCCAT
CTGCCAAAGTACTTGGCTTAGCCTTCTTCGCAAAGAGACATGGTACTTCAAAACGATATG
CTGCCATAATAGCTGGCGCAATGCCTGATGCTTCAACAGTTAAGATTTTAGTGATGCCTT
GATCTTTAAACTGTTCATAAAATGTTTCGCCAATGTCATACATTAACACTGCATCAATTT
GATGGTTTAAAAACCCATCTACCTTCAAGATTTTTTCGCCAATGACGACGCCGTCTTCTT
TAACCTTCTACCTACTAGTGAACCTACAACACGAATACCGTCTAATACTAAACGTGGAAT
TGATAAGTCCATAGATTCTGTTGGTAAGCCGACTGCGACTACAGTACCGCCAGCACGTAC
AGATTCAACAGCTGCGTTGAAGGCTTGTTTAGCAACCGCTGTAACAACTGCCGCATGTGC
ACCGTCTACATGTTCTTGAATGTATTTTGCGACATCCACTTCTTTAGGGTTTAAAGTAAC
ATCTGCATCGTATTGTTTCGCAAATTCAAGTTGACTGTCATTGATGTCTACTGCGATAAC
TTTAGCGTTAAATACTTTTTTCGCATATTGCAAAGCTAAGTTTCCAAGTCCACCTAAACC
GAATAAGACAAGCCATTCACCAGCACGTACGCCTGATTCTTTAATCGCTTTGTATGTTGT
GACACCTGCACATGTGATACTGCTGGCAGCTTCTGATGATAAACCATCTGGTTTTTCCAA
CCTTTATATAATTTTTCTCTTTCTTTTTCATCCATTTCAGGCTCGAAAGCTTGATCTAAT
TTCCAACGGTTCGCAATTTCTGCTTTACTATCCCAGAAACCTACCGCAAGTCCTGCAAGG

TAAGCAGCACCTAACGCAGTCGTTTCATTGATTTCAGGACGTTCTACTGTAATATTTTGA
ATATCTGCTTGGAACTGCATTAAGAAGTTGTTCTTAACAGCACCACCATCTACACGGATA
TTCTTCACTTCAATACCTGAGTCTTGAATCATCGCTTCAACCACATCACGTGTTTGGTAA
CAAATAGATTCTAACGTTGCTCTGATAAAGTGTTCTTTCTCAGTCGCACGTGTAATACCA
AATACTGCACCACGTGCATTCGCATCCCAATATGGTGTACCGAGTCCAACAAACGCCGGT
ACCACATAAACATCATCACTTGATTCAATACGGTTCGCATAGTTTTCAGATTCAGGTGCT
GAGTTAATCATACGTAAACCATCTCGCAACCATTGAATAGCTGAACCAGAGACGAAAATA
GAACCTTCTAATGCATAATTCACTTCGCCATCTAAGCCGAATGCGATTGTTGTTAATAAA
CCGTTATCCGATTGAATCGCTTCTTTACCAGTGTTCATTAACATGAATCCACCCGTACCA
TATGTGTTTTTCACTTCACCACGTTCGAAGCATGCTTGTCCGAATAAGGCTGCTTGCTGA
TCCCCTGCGATTCCCGCAATCGGTACTTCTTGTCCGAAGAAATGATAATCTTTTGTTTTC
GCATAAATCTCACTTTTCTTTAGCATGGTTAATCGCTTCAATTGTTTGAGGCATAACACC
GTCGTCTGCTGCAACCACTAAGATTGTGATATCAGTGACTTGCGCACCACGTGCACGCAT
AGTAGTGAAGGCTGCGTGTCCAGGTGTATCTAAGAAAGTGATTTTTTTGCCGTCATTTGT
GATTTGGTAAGCACCGATATGTTGTGTGATACCGCCCGCTTCACCTTGTGTCACATGTGT
ATTTCTGATTGAGTCAAGTAGCGTTGTTTTACCATGGTCAACGTGTCCCATGATTGTTAC
AACTGCTGGACGCTCAATTGCATCCGGATCTTCTTCTTCATCATCGAAGTAGATATCTAA
GTCTTCTTCGTCAATGACTACTTCTTTTTCTACTTCTACACCATAATCATCAGCGATTAA
CTCAATTGTTTCATCGTCTAATGATTGGTTAATGTTTGCCATAATACCAAGTAAGAATAA
TTTCTTAACAATACTTGATGACTCAATATTCATTTTGTCAGCTAATTCGCCAACCGTAAT
ACCTTCTAAATAAGTAATTTTTTCTGGCATTTCTTTTTTCGGTTGTTCTGGTGCTGGTTT
TTGATTATTGTTTTTCTTATGTTTGTTGTTGT
>NZ_CP016157
GATTGCTTGAATACGTTCTTTAGCAAGTTCTACTTCATCGTTTACCACAACGTAGTCATA
AAGATTCATCATTTCCACTTCTTTACGTGCTTCTTTCACACGACTTTGAATCTTTTCGTC
TGATTCTGTACCGCGGCCGACTAAACGCTCTCTTAAGTGGTCTAAACTTGGCGGTGCTAG
GAAGATAAATAATGCATCAGGGAATTTCTTGCGTACTTGTTTCGCACCTTCAACTTCGAT
TTCTAAGAAGACATCATAGCCTTCATCCATAGTGTCTTTCACATACTGTACAGGTGTACC
GTAGTAGTTACCTACATATTCTGCATACTCAATAAATTGATCTTGTGCGATAAGTTGCTC
AAACTCTCGTTTTGTCTTGAAAAAATAATCAACGCCATCTTGTTCACCTTCACGCATTTC
ACGTGTCGTCATTGAAATTGAATATTTGTATGATGTTTTCGGATCATCAAATATTTCTTT
GCGGACAGTACCTTTTCCGACTCCAGATGGACCTGATAAGACAATTAGTAAACCTTTTTC
ATTATCCATGCCTTACGTTTAAACCTTGTAAAACAGGTCCGATTGCTTCATATCCGCCTA
AACGTTGCGCAATTTTGTAACCGATGTTACCTGCTTCTAAGCTTGGGAATACGAATACAT
TTGCGTCACCTTTAATTTGTGCATCCGGTGCTTTTTTAGCCGCCACTTCTGGAACGATTG
CTGCGTCGAATTGGAATTCACCATCGATAACAACATCTTTTAAGCCTTCTGATTCAGCTT
TTTCTTGTGCAGCTTTTGTTGCTTCCGCTACTTTTTCAACATCGTCTGATTTTGCTGAAC
CTTTTGTTGAGAAGCTTAATAATGCTACTTTAGGATCCATGCCGAAGCTTTTCGCTGTTT
TTGCACTTTCTACCGCAATTTCAGCTAAGTCTGTTGCTTCTAATGTAGGGTTGATTGCAC
AATCACCGAAAACATATTGTTCTTCGCCTTTAATCATGAAGAACACACCTGATGTTTTCG
CAACACCTGGTTTAGTTTTGATGATTTGTAGTGCTGGACGAACTGTATCGCCTGTTGAAT
GTGCAGCACCGCTGACTAGTCCTTCAGCTTTACCTGCATAAACAAGCATTGTACCGAAGT
AGTTTACATTGTTAAGCATATCTTCAGCTTGTTCTTTTGTCGCTTTGCCTTTACGACGTT
CTACAAATGCGTCTACTAATTCTGGTTTCAAGTCGCGACTAAAACTTCTTCGTCTTTCGC
AGTTGGAATATTTTTACCTACGAAGTCAGCGCGTATTGGTAATTCGCGGTGTCCGCGATC
CACTAATGTTGCTAACCCGATTTTTTGTGGTCGAGAATGTAGTAAGATTGCATCGAGTGA
CGCACGTACAGTTCGACCTGTATAAAGCACATCATCAATGATGATAACGACTTTGTCTTT
AAGGTCTACATCAATGACATAAGACTTCTCAGACATTTTAGGTGTCGGTAACTCTAAGTC
ATCTCTGAATTGCGTGATGTCTAATGTTCCTGTAGGAACACTTACGTCTTCAATTTGTTG
AATCTTCGTTTGAATACGTTTCGCTAAAAATTCTCCGCGTGTTTTCACACCAAGCAACAC
AAGATTTTCAGTACCTTTGTTATACTCTAGAATTTCATGTGCGATACGTGTTAAAGTCCG
CTGCATGCCTGCATCATCTAATACAATACGCGACTTGGCAAAGTGAAGAAACTTTTAAGC
CAGCTTCTTCTAATCTTTTACGGCCTTGTTGGAAACTTTTCTCTACTACAATACCGATAC
CTACTGTTTCTGCACCTGCTTGTTCAACTAAACGGTTCAAACCAAGTGCCGCATCTCCAT
TCGCTAAAAAGTCATCGATAATCAATACACGGTCATTTTCCCCTAAAAATTCTTCAGAGA
TAATGACGCGATTCGTCTTGTTTTTCGTAAACGAATGAATATCTGTCTGATAAAATCCAT
CTGCCAAAGTACTTGGCTTAGCCTTCTTCGCAAAGAGACATGGTACTTCAAAACGATATG

CTGCCATAATAGCTGGCGCAATGCCTGATGCTTCAACAGTTAAGATTTTAGTGATGCCTT
GATCTTTGAACTGTTCATAAAATGTTTCGCCAATGTCATACATTAACACTGCATCAATTT
GATGGTTTAAAAACCCATCTACCTTCAAGATTTTTTCGCCAATGACGACGCCGTCTTCTT
TAACCTTCTACCTACTAGTGAACCTACAACACGAATACCGTCTAATACTAAACGTGGAAT
TGATAAGTCCATAGATTCTGTTGGTAAGCCGACTGCGACTACAGTACCGCCAGCACGTAC
AGATTCAACAGCTGCGTTGAAGGCTTGTTTAGCAACCGCTGTAACAACTGCCGCATGTGT
ACCGTCTACATGTTCTTGAATGTATTTTACGACATCCACTTCTTTAGGGTTTAAAGTGAC
ATCTGCATCGTATTGTTTCGCAAATTCAAGTTGACTGTCATTGATATCGACTGCGATAAC
TTTCGCATTAAATACTTTTTTCGCATATTGTAAAGCTAGGTTTCCAAGTCCACCTAAACC
GAATAAGACAAGCCATTCACCAGCACGTACGCCTGATTCTTTAATTGCTTTGTATGTTGT
CACGCCTGCACAAGTGATACTGCTGGCAGCTTCTGATGATAAACCATCTGGTTTTTCCAA
CCTTTATATAATTTTTCTCTTTCTTTTTCATCCATTTCAGGTTCGAAAGCTTGATCTAAT
TTCCAACGGTTCGCAATTTCTGCTTTACTATCCCAGAAACCTACCGCAAGTCCTGCAAGA
TAAGCAGCACCTAACGCAGTCGTTTCATTGATTTCAGGACGTTCTACTGTAATGTTTTGA
ATATCTGCTTGGAACTGCATTAAGAAGTTGTTCTTAACAGCACCACCATCTACACGGATA
TTCTTCACTTCAATACCTGAGTCTTGAATCATCGCTTCAACCACATCACGTGTTTGGTAA
CAAATAGATTCTAACGTTGCTCTGATAAAGTGTTCTTTCTCAGTCGCACGCGTAATACCG
AATACTGCACCACGCGCATTCGCATCCCAATAAGGTGTACCGAGTCCAACAAATGCAGGC
ACCACATAAACGCCATCACTTGATTCAATACGGTTCGCATAGTTTTCAGATTCAGGTGCG
GAGTTAATCATACGTAAACCATCTCGCAACCATTGAATAGCTGAGCCAGAGACGAAAATA
GAACCTTCTAATGCATAATTCACTTCGCCATCTAAGCCGAATGCGATTGTTGTTAATAAA
CCGTTATCAGATTGAATCGCTTCTTTACCAGTGTTCATTAACATGAATCCACCTGTACCG
TACGTGTTTTTCACTTCACCACGTTCGAAGCATGCTTGTCCGAATAAGGCTGCTTGCTGA
TCCCCTGCGATTCCCGCAATCGGTACTTCTTGTCCGAAGAAATGATAATCTTTTGTTTTC
GCATAAATCTCACTTTTCTTTTGCATGGTTAATCGCTTCAATTGTTTGAGGCATAACACC
GTCGTCTGCTGCAACCACTAAGATTGTGATATCAGTGACTTGCGCACCACGTGCACGCAT
AGTAGTGAAGGCTGCGTGTCCAGGTGTATCTAAGAAAGTGATTTTTTTGCCGTCATTTGT
GATTTGGTAAGCACCGATATGTTGTGTGATACCGCCCGCTTCACCTTGTGTCACATGTGT
ATTTCTGATTGAGTCAAGTAGCGTTGTTTTACCATGGTCAACGTGTCCCATGATTGTTAC
AACTGCTGGACGCTCAATTGCATCCGGATCTTCTTCTTCATCATCGAAGTAGATATCTAA
GTCTTCTTCGTCAATGACTACTTCTTTTTCTACTTCTACACCATAATCATCAGCGATTAA
CTCAATTGTTTCATCGTCTAATGATTGGTTAATGTTTGCCATAATACCAAGTAAGAATAA
TTTCTTAACAATACTTGATGACTCAATATTCATTTTGTCAGCTAATTCGCCAACCGTAAT
ACCTTCTAAATAAGTAATTTTTTCTGGCATTTCTTTTTTCGGTTGTTCTGGTGCTGGTTT
TTGATTATTGTTTTTCTTATGTTTGTTGTTGT
>NZ_CP017430
GATTGCTTGAATACGTTCTTTAGCAAGTTCTACTTCATCGTTTACCACAACGTAGTCATA
AAGATTCATCATTTCCACTTCTTTACGTGCTTCTTTCACACGACTTTGAATCTTTTCGTC
TGATTCTGTACCGCGGCCGACTAAACGCTCTCTTAAGTGGTCTAAACTTGGCGGTGCTAG
GAAGATAAATAATGCATCAGGGAATTTCTTGCGTACTTGTTTCGCACCTTCAACTTCGAT
TTCTAAGAAGACATCATAGCCTTCATCCATAGTGTCTTTCACATACTGTACAGGTGTACC
GTAGTAGTTACCTACATATTCTGCATACTCAATAAATTGATCTTGTGCGATAAGTTGCTC
AAACTCTCGTTTTGTCTTGAAAAAATAATCAACGCCATCTTGTTCACCTTCACGCATTTC
ACGTGTCGTCATTGAAATTGAATATTTGTATGATGTTTTCGGATCATCAAATATTTCTTT
GCGGACAGTACCTTTTCCGACTCCAGATGGACCTGATAAGACAATTAGTAAACCTTTTTC
ATTATCCATGCCTTACGTTTAAACCTTGTAAAACAGGTCCGATTGCTTCATATCCGCCTA
AACGTTGCGCAATTTTGTAACCGATGTTACCTGCTTCTAAGCTTGGGAATACGAATACAT
TTGCGTCACCTTTAATTTGTGCATCCGGTGCTTTTTTAGCCGCCACTTCTGGAACGATTG
CTGCGTCGAATTGGAATTCACCATCGATAACAACATCTTTTAAGCCTTCTGATTCAGCTT
TTTCTTGTGCAGCTTTTGTTGCTTCCGCTACTTTTTCAACATCGTCTGATTTTGCTGAAC
CTTTTGTTGAGAAGCTTAATAATGCTACTTTAGGATCCATGCCGAAGCTTTTCGCTGTTT
TTGCACTTTCTACCGCAATTTCAGCTAAGTCTGTTGCTTCTAATGTAGGGTTGATTGCAC
AATCACCGAAAACATATTGTTCTTCGCCTTTAATCATGAAGAACACACCTGATGTTTTCG
CAACACCTGGTTTAGTTTTGATGATTTGTAGTGCTGGACGAACTGTATCGCCTGTTGAAT
GTGCAGCACCGCTGACTAGTCCTTCAGCTTTACCTGCATAAACAAGCATTGTACCGAAGT
AGTTTACATTGTTAAGCATATCTTCAGCTTGTTCTTTTGTCGCTTTGCCTTTACGACGTT
CTACAAATGCGTCTACTAATTCTGGTTTCAAGTCGCGACTAAAACTTCTTCGTCTTTCGC

AGTTGGAATATTTTTACCTACGAAGTCAGCGCGTATTGGTAATTCGCGGTGTCCGCGATC
CACTAATGTTGCTAACCCGATTTTTTGTGGTCGAGAATGTAGTAAGATTGCATCGAGTGA
CGCACGTACAGTTCGACCTGTATAAAGCACATCATCAATGATGATAACGACTTTGTCTTT
AAGGTCTACATCAATGACATAAGACTTCTCAGACATTTTAGGTGTCGGTAACTCTAAGTC
ATCTCTGAATTGCGTGATGTCTAATGTTCCTGTAGGAACACTTACGTCTTCAATTTGTTG
AATCTTCGTTTGAATACGTTTCGCTAAAAATTCTCCGCGTGTTTTCACACCAAGCAACAC
AAGATTTTCAGTACCTTTGTTATACTCTAGAATTTCATGTGCGATACGTGTTAAAGTCCG
CTGCATGCCTGCATCATCTAATACAATACGCGACTTGGCAAAGTGAAGAAACTTTTAAGC
CAGCTTCTTCTAATCTTTTACGGCCTTGTTGGAAACTTTTCTCTACTACAATACCGATAC
CTACTGTTTCTGCACCTGCTTGTTCAACTAAACGGTTCAAACCAAGTGCCGCATCTCCAT
TCGCTAAAAAGTCATCGATAATCAATACACGGTCATTTTCCCCTAAAAATTCTTCAGAGA
TAATGACGCGATTCGTCTTGTTTTTCGTAAACGAATGAATATCTGTCTGATAAAATCCAT
CTGCCAAAGTACTTGGCTTAGCCTTCTTCGCAAAGAGACATGGTACTTCAAAACGATATG
CTGCCATAATAGCTGGCGCAATGCCTGATGCTTCAACAGTTAAGATTTTAGTGATGCCTT
GATCTTTGAACTGTTCATAAAATGTTTCGCCAATGTCATACATTAACACTGCATCAATTT
GATGGTTTAAAAACCCATCTACCTTCAAGATTTTTTCGCCAATGACGACGCCGTCTTCTT
TAACCTTCTACCTACTAGTGAACCTACAACACGAATACCGTCTAATACTAAACGTGGAAT
TGATAAGTCCATAGATTCTGTTGGTAAGCCGACTGCGACTACAGTACCGCCAGCACGTAC
AGATTCAACAGCTGCGTTGAAGGCTTGTTTAGCAACCGCTGTAACAACTGCCGCATGTGT
ACCGTCTACATGTTCTTGAATGTATTTTACGACATCCACTTCTTTAGGGTTTAAAGTGAC
ATCTGCATCGTATTGTTTCGCAAATTCAAGTTGACTGTCATTGATATCGACTGCGATAAC
TTTCGCATTAAATACTTTTTTCGCATATTGTAAAGCTAGGTTTCCAAGTCCACCTAAACC
GAATAAGACAAGCCATTCACCAGCACGTACGCCTGATTCTTTAATTGCTTTGTATGTTGT
CACGCCTGCACAAGTGATACTGCTGGCAGCTTCTGATGATAAACCATCTGGTTTTTCCAA
CCTTTATATAATTTTTCTCTTTCTTTTTCATCCATTTCAGGTTCGAAAGCTTGATCTAAT
TTCCAACGGTTCGCAATTTCTGCTTTACTATCCCAGAAACCTACCGCAAGTCCTGCAAGA
TAAGCAGCACCTAACGCAGTCGTTTCATTGATTTCAGGACGTTCTACTGTAATGTTTTGA
ATATCTGCTTGGAACTGCATTAAGAAGTTGTTCTTAACAGCACCACCATCTACACGGATA
TTCTTCACTTCAATACCTGAGTCTTGAATCATCGCTTCAACCACATCACGTGTTTGGTAA
CAAATAGATTCTAACGTTGCTCTGATAAAGTGTTCTTTCTCAGTCGCACGCGTAATACCG
AATACTGCACCACGCGCATTCGCATCCCAATAAGGTGTACCGAGTCCAACAAATGCAGGC
ACCACATAAACGCCATCACTTGATTCAATACGGTTCGCATAGTTTTCAGATTCAGGTGCG
GAGTTAATCATACGTAAACCATCTCGCAACCATTGAATAGCTGAGCCAGAGACGAAAATA
GAACCTTCTAATGCATAATTCACTTCGCCATCTAAGCCGAATGCGATTGTTGTTAATAAA
CCGTTATCAGATTGAATCGCTTCTTTACCAGTGTTCATTAACATGAATCCACCTGTACCG
TACGTGTTTTTCACTTCACCACGTTCGAAGCATGCTTGTCCGAATAAGGCTGCTTGCTGA
TCCCCTGCGATTCCCGCAATCGGTACTTCTTGTCCGAAGAAATGATAATCTTTTGTTTTC
GCATAAATCTCACTTTTCTTTTGCATGGTTAATCGCTTCAATTGTTTGAGGCATAACACC
GTCGTCTGCTGCAACCACTAAGATTGTGATATCAGTGACTTGCGCACCACGTGCACGCAT
AGTAGTGAAGGCTGCGTGTCCAGGTGTATCTAAGAAAGTGATTTTTTTGCCGTCATTTGT
GATTTGGTAAGCACCGATATGTTGTGTGATACCGCCCGCTTCACCTTGTGTCACATGTGT
ATTTCTGATTGAGTCAAGTAGCGTTGTTTTACCATGGTCAACGTGTCCCATGATTGTTAC
AACTGCTGGACGCTCAATTGCATCCGGATCTTCTTCTTCATCATCGAAGTAGATATCTAA
GTCTTCTTCGTCAATGACTACTTCTTTTTCTACTTCTACACCATAATCATCAGCGATTAA
CTCAATTGTTTCATCGTCTAATGATTGGTTAATGTTTGCCATAATACCAAGTAAGAATAA
TTTCTTAACAATACTTGATGACTCAATATTCATTTTGTCAGCTAATTCGCCAACCGTAAT
ACCTTCTAAATAAGTAATTTTTTCTGGCATTTCTTTTTTCGGTTGTTCTGGTGCTGGTTT
TTGATTATTGTTTTTCTTATGTTTGTTGTTGT
>NZ_CP023497
GATTGCTTGAATACGTTCTTTAGCAAGTTCTACTTCATCGTTTACCACAACGTAGTCATA
AAGATTCATCATTTCCACTTCTTTACGTGCTTCTTTCACACGACTTTGAATCTTTTCGTC
TGATTCTGTACCGCGGCCGACTAAACGCTCTCTTAAGTGGTCTAAACTTGGCGGTGCTAG
GAAGATAAATAATGCATCAGGGAATTTCTTGCGTACTTGTTTCGCACCTTCAACTTCGAT
TTCTAAGAAGACATCATAGCCTTCATCCATAGTGTCTTTCACATACTGTACAGGTGTACC
GTAGTAGTTACCTACATATTCTGCATACTCAATAAATTGATCTTGTGCGATAAGTTGCTC
AAACTCTCGTTTTGTCTTGAAAAAATAATCAACGCCATCTTGTTCACCTTCACGCATTTC
ACGTGTCGTCATTGAAATTGAATATTTGTATGATGTTTTCGGGTCATCAAATATTTCTTT

GCGGACAGTACCTTTTCCGACTCCAGATGGACCTGATAAGACAATTAGTAAACCTTTTTC
ATTATCCATGCCTTACGTTTAAACCTTGTAAAACAGGTCCGATTGCTTCATATCCGCCTA
AACGTTGCGCAATTTTGTAACCGATGTTACCTGCTTCTAAGCTTGGGAATACGAATACAT
TTGCGTCACCTTTAATTTGTGCATCTGGTGCTTTTTTAGCCGCCACTTCTGGAACGATTG
CTGCGTCGAATTGGAATTCACCATCGATAACAACATCTTTTAAGCCTTCTGATTCAGCTT
TTTCTTGTGCAGCTTTTGTTGCTTCCGCTACTTTTTCAACATCGTCTGATTTTGCTGAAC
CTTTTGTTGAGAAGCTTAATAATGCTACTTTAGGATCCATGCCGAAGCTTTTCGCTGTTT
TTGCACTTTCTACCGCAATTTCAGCTAAGTCTGTTGCTTCTAATGTAGGGTTGATTGCAC
AATCACCGAAAACATATTGTTCTTCGCCTTTAATCATGAAGAACACACCTGATGTTTTCG
CAACACCTGGTTTAGTTTTGATGATTTGTAGTGCTGGACGAACTGTATCGCCTGTTGAGT
GTGCAGCACCGCTGACTAGTCCTTCAGCTTTACCTGCATAAACAAGCATTGTACCGAAGT
AGTTTACATTGTTAAGCATATCTTCAGCTTGTTCTTTTGTCGCTTTACCTTTACGACGTT
CTACAAATGCGTCTACTAATTCTGGTTTCAAGTCGCGACTAAAACTTCTTCGTCTTTCGC
AGTTGGAATATTTTTACCTACGAAGTCAGCGCGTATTGGTAATTCGCGGTGTCCGCGATC
CACTAATGTTGCTAACCCGATTTTTTGTGGTCGAGAATGTAGTAAGATTGCATCGAGTGA
CGCACGTACAGTTCGACCTGTATAAAGCACATCATCAATGATGATAACGACTTTGTCTTT
AAGGTCTACATCAATGACATAAGATTTCTCAGACATTTTAGGTGTCGGTAACTCTAAATC
ATCTCTGAATTGCGTGATGTCTAATGTTCCTGTAGGAACACTTACGTCTTCAATTTGTTG
AATCTTCATTTGAATACGTTTCGCTAAAAATTCTCCGCGTGTTTTCACACCAAGCAACAC
AAGATTTTCAGTACCTTTGTTATACTCTAGAATTTCATGTGCGATACGTGTTAAAGTCCG
CTGCATGCCTGCATCATCTAATACAATACGCGACTTGGCAAAGTGAAGAAACTTTCAAGC
CAGCTTCTTCTAAACGTTTACGGCCTTGTTGGAAACTTTTCTCCACAACAATACCGATAC
CGACTGTTTCTGCACCTGCTTGTTCAACTAAACGATTCAAACCAAGTGCCGCATCTCCAT
TCGCTAAAAAGTCATCGATAATCAATACACGATCATTTTCTCCTAAAAATTCTTCAGAAA
TAATGACACGATTCGTCTTGTTTTTCGTAAACGAATGAATATCTGTCTGATAAAATCCAT
CTGCCAAAGTACTTGGCTTAGCCTTCTTCGCAAAGAGACATGGTACTTCAAAACGATATG
CTGCCATAATAGCTGGCGCAATACCTGATGCTTCAACAGTTAAGATTTTAGTGATGCCTT
GATCTTTAAACTGTTCATAAAATGTTTCGCCAATGTCATACATTAACACTGCATCAATTT
GATGGTTTAAAAACCCATCTACCTTCAAGATTTTTTCGCCAATGACGACGCCGTCTTCTT
TAACCTTCTACCTACTAGTGAACCTACAACACGAATACCGTCTAATACCAAACGTGGAAT
TGATAAGTCCATAGATTCTGTTGGTAAGCCGACTGCGACTACAGTACCGCCAGCACGTAC
AGATTCAACAGCTGCGTTGAAGGCTTGTTTAGCAACCGCTGTAACAACTGCCGCATGTGC
ACCGTCTACATGTTCTTGAATGTATTTTGCGACATCCACTTCTTTAGGGTTTAAAGTAAC
ATCTGCATCGTATTGTTTCGCAAATTCAAGTTGACTGTCATTGATATCGACTGCGATAAC
TTTCGCATTAAATACTTTTTTGGCATATTGCAAAGCTAAGTTTCCAAGTCCACCTAAACC
GAATAAGACAAGCCATTCACCAGCACGTACGCCTGATTCTTTAATCGCTTTGTATGTTGT
GACACCTGCACATGTGATACTGCTGGCAGCTTCTGATGATAAACCATCTGGTTTTTCCAA
CCTTTATATAATTTTTCTCTTTCTTTTTCATCCATTTCAGGTTCGAAAGCTTGATCTAAT
TTCCAACGGTTCGCAATTTCTGCTTTACTATCCCAGAAACCTACCGCAAGTCCTGCAAGA
TAAGCAGCACCTAACGCAGTCGTTTCATTGATTTCAGGACGTTCTACTGTAATGTTTTGA
ATATCTGCTTGGAACTGCATTAAGAAGTTGTTCTTAACAGCACCACCATCTACACGGATA
TTCTTCACTTCAATACCTGAGTCTTGAATCATCGCTTCAACCACATCACGTGTTTGGTAA
CAAATAGATTCTAACGTTGCTCTGATAAAGTGTTCTTTCTCAGTCGCACGTGTAATACCA
AATACTGCACCACGCGCATTCGCATCCCAATATGGTGTACCGAGTCCAACAAATGCAGGC
ACCACATAAACGCCATCACTTGATTCAATACGGTTCGCATAGTTTTCAGATTCAGGTGCT
GAGTTAATCATACGTAAACCATCTCGCAACCATTGAATAGCTGAGCCAGAGACGAAAATA
GAACCTTCTAATGCATAATTCACTTCGCCATCTAAGCCGAATGCGATTGTTGTTAATAAA
CCGTTATCAGATTGAATCGCTTCTTTACCAGTGTTCATTAACATGAATCCACCTGTACCG
TACGTGTTTTTCACTTCACCACGTTCGAAGCATGCTTGTCCGAATAAGGCTGCTTGCTGA
TCCCCTGCGATTCCCGCAATCGGTACTTCTTGTCCGAAGAAATGATAATCTTTTGTTTTC
GCATAAATCTCACTTTTCTTTCGCATGGTTAATCGCTTCAATTGTTTGAGGCATAACACC
GTCGTCTGCTGCAACCACTAAGATTGTGATATCAGTGACTTGCGCACCACGTGCACGCAT
AGTAGTGAAGGCTGCGTGTCCAGGTGTATCTAAGAAAGTGATTTTTTTGCCGTCATTTGT
GATTTGGTAAGCACCGATATGTTGTGTGATACCGCCCGCTTCACCTTGTGTCACATGTGT
ATTTCTGATTGAGTCAAGTAGCGTTGTTTTACCATGGTCAACGTGTCCCATGATTGTTAC
AACTGCTGGACGCTCAATTGCATCCGGATCTTCTTCTTCATCATCGAAGTAGATGTCTAA
GTCTTCTTCGTCAATGACTACTTCTTTTTCTACTTCTACACCATAATCATCAGCGATTAA

CTCAATTGTTTCATCGTCTAATGATTGGTTAATGTTTGCCATAATACCAAGTAAGAATAA
TTTCTTAACAATACTTGATGACTCAATATTCATTTTGTCAGCTAATTCGCCAACCGTAAT
ACCTTCTAAATAAGTAATTTTTTCTGGCATTTCTTTTTTCGGTTGTTCTGGTGCTGGTTT
TTGATTATTGTTTTTCTTATGTTTGTTGTTGT
>NZ_LS483313
GATTGCTTGAATACGTTCTTTAGCAAGTTCTACTTCATCGTTTACCACAACGTAGTCATA
AAGATTCATCATTTCCACTTCTTTACGTGCTTCTTTCACACGACTTTGAATCTTTTCGTC
TGATTCTGTACCGCGGCCGACTAAACGCTCTCTTAAGTGGTCTAAACTTGGCGGTGCTAG
GAAGATAAATAATGCATCAGGGAATTTCTTGCGTACTTGTTTCGCACCTTCAACTTCGAT
TTCTAAGAAGACATCATAGCCTTCATCCATAGTGTCTTTCACATACTGTACAGGTGTACC
GTAGTAGTTACCTACATATTCTGCATACTCAATAAATTGATCTTGTGCGATAAGTTGCTC
AAACTCTCGTTTTGTCTTGAAAAAATAATCAACGCCATCTTGTTCACCTTCACGCATTTC
ACGTGTCGTCATTGAAATTGAATATTTGTATGATGTTTTCGGATCATCAAATATTTCTTT
GCGGACAGTACCTTTTCCGACTCCAGATGGACCTGATAAGACAATTAGTAAACCTTTTTC
ATTATCCATGCCTTACGTTTAAACCTTGTAAAACAGGTCCGATTGCTTCATATCCGCCTA
AACGTTGCGCAATTTTGTAACCGATGTTACCTGCTTCTAAGCTTGGGAATACGAATACAT
TTGCGTCACCTTTAATTTGTGCATCTGGTGCTTTTTTAGCCGCCACTTCTGGAACGATTG
CTGCGTCGAATTGGAATTCACCATCGATAACAACATCTTTTAAGCCTTCTGATTCAGCTT
TTTCTTGTGCAGCTTTTGTTGCTTCCGCTACTTTTTCAACATCGTCTGATTTTGCTGAAC
CTTTTGTTGAGAAGCTTAATAATGCTACTTTAGGATCCATGCCGAAGCTTTTCGCTGTTT
TTGCACTTTCTACCGCAATTTCAGCTAAGTCTGTTGCTTCTAATGTAGGGTTGATTGCAC
AATCACCGAAAACATATTGTTCTTCGCCTTTAATCATGAAGAACACACCTGATGTTTTCG
CAACACCTGGTTTAGTTTTGATGATTTGTAGTGCTGGACGAACTGTATCGCCTGTTGAGT
GTGCAGCACCGCTGACTAGTCCTTCAGCTTTACCTGCATAAACAAGCATTGTACCGAAGT
AGTTTACATTGTTAAGCATATCTTCAGCTTGTTCTTTTGTCGCTTTACCTTTACGACGTT
CTACAAATGCGTCTACTAATTCTGGTTTCAAGTCGCGACTAAAACTTCTTCGTCTTTCGC
AGTTGGAATATTTTTACCTACGAAGTCAGCGCGTATTGGTAATTCGCGGTGTCCGCGATC
CACTAATGTTGCTAACCCGATTTTTTGTGGTCGAGAATGTAGTAAGATTGCATCGAGTGA
CGCACGTACAGTTCGACCTGTATAAAGCACATCATCAATGATGATAACGACTTTGTCTTT
AAGGTCTACATCAATGACATAAGACTTCTCAGACATTTTAGGTGTCGGTAACTCTAAGTC
ATCTCTGAATTGCGTGATGTCTAATGTTCCTGTAGGAACACTTACGTCTTCAATTTGTTG
AATCTTCGTTTGAATACGTTTCGCTAAAAATTCTCCGCGTGTTTTCACACCAAGCAACAC
AAGATTTTCAGTACCTTTGTTATACTCTAAAATTTCATGTGCGATACGTGTTAAAGTCCG
CTGCATGCCTGCATCATCTAATACAATACGCGACTTGGCAAAGCGAAGAAACTTTTAAGC
CAGCTTCTTCTAATCGTTTACGGCCTTGTTGGAAACTTTTTTCCACGACAATACCGATAC
CGACTGTTTCTGCACCTGCTTGTTCAACTAAACGATTCAAACCAAGTGCCGCATCTCCAT
TCGCTAAAAAGTCATCGATAATCAATACACGATCATTTTCTCCTAAAAAATTCTTCAGAGA
TAATGACACGATTCGTCTTGTTTTTCGTAAACGAATGAATATCTGTCTGATAAAATCCAT
CTGCCAAAGTACTTGGCTTAGCCTTCTTCGCAAAGAGACATGGTACTTCAAAACGATATG
CTGCCATAATAGCTGGCGCAATGCCTGATGCTTCAACAGTTAAGATTTTAGTGATGCCTT
GATCTTTAAACTGTTCATAAAATGTTTCGCCAATGTCATACATTAACACTGCATCAATTT
GATGGTTTAAAAACCCATCTACCTTCAAGATTTTTTCGCCAATGACGACGCCGTCTTCTT
TAACCTTCTACCTACTAGTGAACCTACAACACGAATACCGTCTAATACCAAACGTGGAAT
TGATAAGTCCATAGATTCTGTTGGTAAGCCGACTGCGACTACAGTACCGCCAGCACGTAC
AGATTCAACAGCTGCGTTGAAGGCTTGTTTAGCAACCGCTGTAACAACTGCCGCATGTGC
ACCGTCTACATGTTCTTGAATGTATTTTGCGACATCCACTTCTTTAGGGTTTAAAGTAAC
ATCTGCATCGTATTGTTTCGCAAATTCAAGTTGACTGTCATTGATATCGACTGCGATAAC
TTTCGCATTAAATACTTTTTTGGCATATTGCAAAGCTAAGTTTCCAAGTCCACCTAAACC
GAATAAGACAAGCCATTCACCAGCACGTACGCCTGATTCTTTAATCGCTTTGTATGTTGT
GACACCTGCACATGTGATACTGCTGGCAGCTTCTGATGATAAACCATCTGGTTTTTCCAA
CCTTTATATAATTTTTCTCTTTCTTTTTCATCCATTTCAGGCTCGAAAGCTTGATCTAAT
TTCCAACGGTTCGCAATTTCTGCTTTACTATCCCAGAAACCTACCGCAAGTCCTGCAAGA
TAAGCAGCACCTAACGCAGTCGTTTCATTGATTTCAGGACGTTCTACTGTAATATTTTGA
ATATCTGCTTGGAACTGCATTAAGAAGTTGTTCTTAACAGCACCACCATCTACACGGATA
TTCTTCACTTCAATACCTGAGTCTTGAATCATCGCTTCAACCACATCACGTGTTTGGTAA
CAAATAGATTCTAACGTTGCTCTGATAAAGTGTTCTTTCTCAGTCGCACGTGTAATACCA
AATACTGCACCACGTGCATTCGCATCCCAATATGGTGTACCGAGTCCAACAAACGCCGGT

ACCACATAAACATCATCACTTGATTCAATACGGTTCGCATAGTTTTCAGATTCAGGTGCT
GAGTTAATCATACGTAAACCATCTCGCAACCATTGAATAGCTGAACCAGAGACGAAAATA
GAACCTTCTAATGCATAATTCACTTCGCCATCTAAGCCGAATGCGATTGTTGTTAATAAA
CCGTTATCCGATTGAATCGCTTCTTTACCAGTGTTCATTAACATGAATCCACCCGTACCA
TATGTGTTTTTCACTTCACCACGTTCGAAGCATGCTTGTCCGAATAAGGCTGCTTGCTGA
TCCCCTGCGATTCCCGCAATCGGTACTTCTTGTCCGAAGAAATGATAATCTTTTGTTTTC
GCATAAATCTCACTTTTCTTTAGCATGGTTAATCGCTTCAATTGTTTGAGGCATAACACC
GTCGTCTGCTGCAACCACTAAGATTGTGATATCAGTGACTTGCGCACCACGTGCACGCAT
AGTAGTGAAGGCTGCGTGTCCAGGTGTATCTAAGAAAGTGATTTTTTTGCCGTCATTTGT
GATTTGGTAAGCACCGATATGTTGTGTGATACCGCCCGCTTCACCTTGTGTCACATGTGT
ATTTCTGATTGAGTCAAGTAGCGTTGTTTTACCATGGTCAACGTGTCCCATGATTGTTAC
AACTGCTGGACGCTCAATTGCATCCGGATCTTCTTCTTCATCATCGAAGTAGATATCTAA
GTCTTCTTCGTCAATGACTACTTCTTTTTCTACTTCTACACCATAATCATCAGCGATTAA
CTCAATTGTTTCATCGTCTAATGATTGGTTAATGTTTGCCATAATACCAAGTAAGAATAA
TTTCTTAACAATACTTGATGACTCAATATTCATTTTGTCAGCTAATTCGCCAACCGTAAT
ACCTTCTAAATAAGTAATTTTTTCTGGCATTTCTTTTTTCGGTTGTTCTGGTGCTGGTTT
TTGATTATTGTTTTTCTTATGTTTGTTGTTGT
>NAS-199
GATTGCTTGAATACGTTCTTTAGCAAGTTCTACTTCATCGTTTACCACAACGTAGTCATA
AAGATTCATCATTTCCACTTCTTTACGTGCTTCTTTCACACGACTTTGAATCTTTTCGTC
TGATTCTGTACCGCGGCCGACTAAACGCTCTCTTAAGTGGTCTAAACTTGGCGGTGCTAG
GAAGATAAATAATGCATCAGGGAATTTCTTGCGTACTTGTTTCGCACCTTCAACTTCGAT
TTCTAAGAAGACATCATAGCCTTCATCCATAGTGTCTTTCACATACTGTACAGGTGTACC
GTAGTAGTTACCTACATATTCTGCATACTCAATAAATTGATCTTGTGCGATAAGTTGCTC
AAACTCTCGTTTTGTCTTGAAAAAATAATCAACGCCATCTTGTTCACCTTCACGCATTTC
ACGTGTCGTCATTGAAATTGAATATTTGTATGATGTTTTCGGATCATCAAATATTTCTTT
GCGGACAGTACCTTTTCCGACTCCAGATGGACCTGATAAGACAATTAGTAAACCTTTTTC
ATTATCCATGCCTTACGTTTAAACCTTGTAAAACAGGTCCGATTGCTTCATATCCGCCTA
AACGTTGCGCAATTTTGTAACCGATGTTACCTGCTTCTAAGCTTGGGAATACGAATACAT
TTGCGTCACCTTTAATTTGTGCATCCGGTGCTTTTTTAGCCGCCACTTCTGGAACGATTG
CTGCGTCGAATTGGAATTCACCATCGATAACAACATCTTTTAAGCCTTCTGATTCAGCTT
TTTCTTGTGCAGCTTTTGTTGCTTCCGCTACTTTTTCAACATCGTCTGATTTTGCTGAAC
CTTTTGTTGAGAAGCTTAATAATGCTACTTTAGGATCCATGCCGAAGCTTTTCGCTGTTT
TTGCACTTTCTACCGCAATTTCAGCTAAGTCTGTTGCTTCTAATGTAGGGTTGATTGCAC
AATCACCGAAAACATATTGTTCTTCGCCTTTAATCATGAAGAACACACCTGATGTTTTCG
CAACACCTGGTTTAGTTTTGATGATTTGTAGTGCTGGACGAACTGTATCGCCTGTTGAAT
GTGCAGCACCGCTGACTAGTCCTTCAGCTTTACCTGCATAAACAAGCATTGTACCGAAGT
AGTTTACATTGTTAAGCATATCTTCAGCTTGTTCTTTTGTCGCTTTGCCTTTACGACGTT
CTACAAATGCGTCTACTAATTCTGGTTTCAAGTCGCGACTAAAACTTCTTCGTCTTTCGC
AGTTGGAATATTTTTACCTACGAAGTCAGCGCGTATTGGTAATTCGCGGTGTCCGCGATC
CACTAATGTTGCTAACCCGATTTTTTGTGGTCGAGAATGTAGTAAGATTGCATCGAGTGA
CGCACGTACAGTTCGACCTGTATAAAGCACATCATCAATGATGATAACGACTTTGTCTTT
AAGGTCTACATCAATGACATAAGACTTCTCAGACATTTTAGGTGTCGGTAACTCTAAGTC
ATCTCTGAATTGCGTGATGTCTAATGTTCCTGTAGGAACACTTACGTCTTCAATTTGTTG
AATCTTCGTTTGAATACGTTTCGCTAAAAATTCTCCGCGTGTTTTCACACCAAGCAACAC
AAGATTTTCAGTACCTTTGTTATACTCTAGAATTTCATGTGCGATACGTGTTAAAGTCCG
CTGCATGCCTGCATCATCTAATACAATACGCGACTTGGCAAAGTGAAGAAACTTTCAAGC
CAGCTTCTTCTAATCGTTTACGGCCTTGTTGGAAACTTTTCTCTACTACAATACCGATAC
CGACTGTTTCTGCACCTGCTTGTTCAACTAAATGGTTCAAACCAAGTGCCGCATCTCCAT
TCGCTAAAAAGTCATCGATAATCAATACACGGTCATTTTCCCCTAAAAATTCTTCAGAGA
TAATGACGCGATTCGTCTTGTTTTTCGTAAACGAATGAATATCTGTCTGATAAAATCCAT
CTGCCAAAGTACTTGGCTTAGCCTTCTTCGCAAAGAGACATGGTACTTCAAAACGATATG
CTGCCATAATAGCTGGCGCAATGCCTGATGCTTCAACAGTTAAGATTTTAGTGATGCCTT
GATCTTTAAACTGTTCATAAAATGTTTCGCCAATGTCATACATTAACACTGCATCAATTT
GATGGTTTAAAAACCCATCTACCTTCAAGATTTTTTCGCCAATGACGACGCCGTCTTCTT
TAACCTTCTACCTACTAGTGACCTACAACACGAATACCGTCTAATACTAAACGTGGAATT
GATAAGTCCATTGATTCTGTTGGTAAGCCGACTGCGACTACAGTACCGCCAGCACGTACA

GATTCAACAGCTGCGTTGAAGGCTTGTTTAGCAACCGCTGTAACAACTGCCGCATGTGCA
CCGCCTACATGTTCTTGAATGTATTTTGCGACATCCACTTCTTTAGGGTTTAAAGTAACA
TCTGCATCGTATTGTTTCGCAAATTCAAGTTGACTGTCATTGATGTCTACTGCGATAACT
TTAGCGTTAAATACTTTTTTCGCATATTGTAAAGCTAGGTTTCCAAGTCCACCTAAACCG
AATAAGACAAGCCATTCACCAGCGCGTACGCCTGATTCTTTAATCGCTTTGTATGTTGTG
ACACCTGCACATGTGATACTGCTGGCAGCTTCTGATGATAAACCATCTGGTTTTTCCAAC
CTTTATATAATTTTTCTCTTTCTTTTTCATCCATTTCAGGTTCGAAAGCTTGATCTAATT
TCCAACGGTTCGCAATTTCTGCTTTACTATCCCAGAAACCTACCGCAAGTCCTGCAAGAT
AAGCAGCACCTAACGCAGTCGTTTCATTGATTTCAGGACGTTCTACTGTAATGTTTTGAA
TATCTGCTTGGAACTGCATTAAGAAGTTGTTCTTAACAGCACCACCATCTACACGGATAT
TCTTCACTTCAATACCTGAGTCTTGAATCATCGCTTCAACCACATCACGTGTTTGGTAAC
AAATAGATTCTAACGTTGCTCTGATAAAGTGTTCTTTCTCAGTCGCACGCGTAATACCGA
ATACTGCACCACGCGCATTCGCATCCCAATAAGGTGTACCGAGTCCAACAAATGCAGGCA
CCACATAAACGCCATCACTTGATTCAATACGGTTCGCATAGTTTTCAGATTCAGGTGCGG
AGTTAATCATACGTAAACCATCTCGCAACCATTGAATAGCTGAGCCAGAGACGAAAATAG
AACCTTCTAATGCATAATTCACTTCGCCATCTAAGCCGAATGCGATTGTTGTTAATAAAC
CGTTATCAGATTGAATCGCTTCTTTACCAGTGTTCATTAACATGAATCCACCTGTACCGT
ACGTGTTTTTCACTTCACCACGTTCGAAGCATGCTTGTCCGAATAAGGCTGCTTGCTGAT
CCCCTGCGATTCCCGCAATCGGTACTTCTTGTCCGAAGAAATGATAATCTTTTGTTTTCG
CATAAATCTCACTTTTCTTTTGCATGGTTAATCGCTTCAATTGTTTGAGGCATAACACCG
TCGTCTGCTGCAACCACTAAGATTGTGATATCAGTGACTTGCGCACCACGTGCACGCATA
GTAGTGAAGGCTGCGTGTCCAGGTGTATCTAAGAAAGTGATTTTTTTGCCGTCATTTGTG
ATTTGGTAAGCACCGATATGTTGTGTGATACCGCCCGCTTCACCTTGTGTCACATGTGTA
TTTCTGATTGAGTCAAGTAGCGTTGTTTTACCATGGTCAACGTGTCCCATGATTGTTACA
ACTGCTGGACGCTCAATTGCATCCGGATCTTCTTCTTCATCATCGAAGTAGATATCTAAG
TCTTCTTCGTCAATGACTACTTCTTTTTCTACTTCTACACCATAATCATCAGCGATTAAC
TCAATTGTTTCATCGTCTAATGATTGGTTAATGTTTGCCATAATACCAAGTAAGAATAAT
TTCTTAACAATACTTGATGACTCAATATTCATTTTGTCAGCTAATTCGCCAACCGTAATA
CCTTCTAAATAAGTAATTTTTTCTGGCATTTCTTTTTTCGGTTGTTCTGGTGCTGGTTTT
TGATTATTGTTTTTCTTATGTTTGTTGTTGT
>NAS-12
GATTGCTTGAATACGTTCTTTAGCAAGTTCTACTTCATCGTTTACCACAACGTAGTCATA
AAGATTCATCATTTCCACTTCTTTACGTGCTTCTTTCACACGACTTTGAATCTTTTCGTC
TGATTCTGTACCGCGGCCGACTAAACGCTCTCTTAAGTGGTCTAAACTTGGCGGTGCTAG
GAAGATAAATAATGCATCAGGGAATTTCTTGCGTACTTGTTTCGCACCTTCAACTTCGAT
TTCTAAGAAGACATCATAGCCTTCATCCATAGTGTCTTTCACATACTGTACAGGTGTACC
GTAGTAGTTACCTACATATTCTGCATACTCAATAAATTGATCTTGTGCGATAAGTTGCTC
AAACTCTCGTTTTGTCTTGAAAAAATAATCAACGCCATCTTGTTCACCTTCACGCATTTC
ACGTGTCGTCATTGAAATTGAATATTTGTATGATGTTTTCGGATCATCAAATATTTCTTT
GCGGACAGTACCTTTTCCGACTCCAGATGGACCTGATAAGACAATTAGTAAACCTTTTTC
ATTATCCATGCCTTACGTTTAAACCTTGTAAAACAGGTCCGATTGCTTCATATCCGCCTA
AACGTTGCGCAATTTTGTAACCGATGTTACCTGCTTCTAAGCTTGGGAATACGAATACAT
TTGCGTCACCTTTAATTTGTGCATCTGGTGCTTTTTTAGCCGCCACTTCTGGAACGATTG
CTGCGTCGAATTGGAATTCACCATCGATAACAACATCTTTTAAGCCTTCTGATTCAGCTT
TTTCTTGTGCAGCTTTTGTTGCTTCCGCTACTTTTTCAACATCGTCTGATTTTGCTGAAC
CTTTTGTTGAGAAGCTTAATAATGCTACTTTAGGATCCATGCCGAAGCTTTTCGCTGTTT
TTGCACTTTCTACCGCAATTTCAGCTAAGTCTGTTGCTTCTAATGTAGGGTTGATTGCAC
AATCACCGAAAACATATTGTTCTTCGCCTTTAATCATGAAGAACACACCTGATGTTTTCG
CAACACCTGGTTTAGTTTTGATGATTTGTAGTGCTGGACGAACTGTATCGCCTGTTGAGT
GTGCAGCACCGCTGACTAGTCCTTCAGCTTTACCTGCATAAACAAGCATTGTACCGAAGT
AGTTTACATTGTTAAGCATATCTTCAGCTTGTTCTTTTGTCGCTTTGCCTTTACGACGTT
CTACAAATGCGTCTACTAATTCTGGTTTCAAGTCGCGACTAAAACTTCTTCGTCTTTCGC
AGTTGGAATATTTTTACCTACAAAGTCAGCGCGTATTGGTAATTCGCGGTGTCCGCGATC
CACTAATGTTGCTAACCCGATTTTTTGTGGTCGAGAATGTAGTAAGATTGCATCGAGTGA
CGCACGTACAGTTCGACCCGTATAAAGCACATCATCAATGATGATAACGACTTTGTCTTT
AAGGTCTACATCAATGACATAAGACTTCTCAGACATTTTAGGTGTCGGTAACTCTAAATC
ATCTCTGAATTGCGTGATGTCTAATGTTCCTGTAGGAACACTTACGTCTTCAATTTGTTG

AATCTTCATTTGAATACGTTTCGCTAAAAATTCTCCGCGTGTTTTCACACCAAGCAACAC
AAGATTTTCAGTACCTTTGTTATACTCTAGAATTTCATGTGCGATACGTGTTAAAGTCCG
CTGCATGCCTGCATCATCTAATACAATACGCGACTTGGCAAAGCGAAGAAACTTTTAAGC
CAGCTTCTTCTAATCGTTTACGGCCTTGTTGGAAACTTTTTTCCACGACAATACCGATAC
CGACTGTTTCTGCACCTGCTTGTTCAACTAAACGATTCAAACCAAGTGCCGCATCTCCAT
TCGCTAAAAAGTCATCGATAATCAATACACGGTCATTTTCCCCTAAAAATTCTTCAGAGA
TAATGACGCGATTCGTCTTGTTTTTCGTAAACGAATGAATATCTGTCTGATAAAATCCAT
CTGCCAAAGTACTTGGCTTAGCCTTCTTCGCAAAGAGACATGGTACTTCAAAACGATATG
CTGCCATAATAGCTGGCGCAATGCCTGATGCTTCAACAGTTAAGATTTTAGTGATGCCTT
GATCTTTGAACTGTTCATAAAATGTTTCGCCAATGTCATACATTAACACTGCATCAATTT
GATGGTTTAAAAACCCATCTACCTTCAAGATTTTTTCGCCAATGACGACGCCGTCTTCTT
TAACCTTCTACCTACTAGTGAACCTACAACACGAATACCGTCTAATACTAAACGTGGAAT
TGATAAGTCCATTGATTCTGTTGGTAAGCCGACTGCGACTACAGTACCGCCAGCACGTAC
AGATTCAACAGCTGCGTTGAAGGCTTGTTTAGCAACCGCTGTAACAACTGCCGCATGTGC
ACCGCCTACATGTTCTTGAATGTATTTTGCGACATCCACTTCTTTAGGGTTTAAAGTGAC
ATCTGCATCGTATTGTTTCGCAAATTCAAGTTGACTGTCATTGATGTCTACTGCGATAAC
TTTAGCGTTAAATACTTTTTTCGCATATTGTAAAGCTAGGTTTCCAAGTCCACCTAAACC
GAATAAAACAAGCCATTCACCAGCACGTACGCCTGATTCTTTAATTGCTTTGTATGTTGT
GACACCTGCACATGTGATACTGCTGGCAGCTTCTGATGATAAACCATCTGGTTTTTCCAA
CCTTTATATAATTTTTCTCTTTCTTTTTCATCCATTTCAGGCTCGAAAGCTTGATCTAAT
TTCCAACGGTTCGCAATTTCTGCTTTACTATCCCAGAAACCTACCGCAAGTCCTGCAAGA
TAAGCAGCACCTAACGCAGTCGTTTCATTGATTTCAGGACGTTCTACTGTAATATTTTGA
ATATCTGCTTGGAACTGCATTAAGAAGTTGTTCTTAACAGCACCACCATCTACACGGATA
TTCTTCACTTCAATACCTGAGTCTTGAATCATCGCTTCAACCACATCACGTGTTTGGTAA
CAAATAGATTCTAACGTTGCTCTGATAAAGTGTTCTTTCTCAGTCGCACGCGTAATACCA
AATACTGCACCACGCGCATTCGCATCCCAATATGGTGTACCGAGTCCAACAAACGCCGGT
ACCACATAAACACCATCACTTGATTCAATACGGTTCGCATAGTTTTCAGATTCGGGTGCT
GAGTTAATCATACGTAAACCATCTCGCAACCATTGAATAGCTGAACCAGAGACGAAAATA
GAACCTTCTAATGCATAATTCACTTCGCCATCTAAGCCGAATGCGATTGTTGTTAATAAA
CCGTTATCCGATTGAATCGCTTCTTTACCAGTGTTCATTAACATGAATCCACCCGTACCA
TATGTGTTTTTCACTTCACCACGTTCGAAGCATGCTTGTCCGAATAAGGCTGCTTGCTGA
TCCCCTGCGATTCCCGCAATCGGTACTTCTTGTCCAAAGAAATGATAATCTTTTGTTTTC
GCATAAACCTCACTTTTCTTTTGCATGGTTAATCGCTTCAATTGTTTGAGGCATAACACC
GTCGTCTGCTGCAACCACTAAGATTGTGATATCAGTGACTTGCGCACCACGTGCACGCAT
AGTAGTGAAGGCTGCGTGTCCAGGTGTATCTAAGAAAGTGATTTTTTTGCCGTCATTTGT
GATTTGGTAAGCACCGATATGTTGTGTGATACCGCCCGCTTCACCTTGTGTCACATGTGT
ATTTCTGATTGAGTCAAGTAGCGTTGTTTTACCATGGTCAACGTGTCCCATGATTGTTAC
AACTGCTGGACGCTCAATTGCATCCGGATCTTCTTCTTCATCATCGAAGTAGATATCTAA
GTCTTCTTCGTCAATGACTACTTCTTTTTCTACTTCTACACCATAATCATCAGCGATTAA
CTCAATTGTTTCATCGTCTAATGATTGGTTAATGTTTGCCATAATACCAAGTAAGAATAA
TTTCTTAACAATACTTGATGACTCAATATTCATTTGTCAGCTAATTCGCCAACCGTAAT
ACCTTCTAAATAAGTAATTTTTTCTGGCATTTCTTTTTTCGGTTGTTCTGGTGCTGGTTT
TTGATTATTGTTTTTCTTATGTTTGTTGTTGT
>NAS-17
GATTGCTTGAATACGTTCTTTAGCAAGTTCTACTTCATCGTTTACCACAACGTAGTCATA
AAGATTCATCATTTCCACTTCTTTACGTGCTTCTTTCACACGACTTTGAATCTTTTCGTC
TGATTCTGTACCGCGGCCGACTAAACGCTCTCTTAAGTGGTCTAAACTTGGCGGTGCTAG
GAAGATAAATAATGCATCAGGGAATTTCTTGCGTACTTGTTTCGCACCTTCAACTTCGAT
TTCTAAGAAGACATCATAGCCTTCATCCATAGTGTCTTTCACATACTGTACAGGTGTACC
GTAGTAGTTACCTACATATTCTGCATACTCAATAAATTGATCTTGTGCGATAAGTTGCTC
AAACTCTCGTTTTGTCTTGAAAAAATAATCAACGCCATCTTGTTCACCTTCACGCATTTC
ACGTGTCGTCATTGAAATTGAATATTTGTATGATGTTTTCGGATCATCAAATATTTCTTT
GCGGACAGTACCTTTTCCGACTCCAGATGGACCTGATAAGACAATTAGTAAACCTTTTTC
ATTATCCATGCCTTACGTTTAAACCTTGTAAAACAGGTCCGATTGCTTCATATCCGCCTA
AACGTTGCGCAATTTTGTAACCGATGTTACCTGCTTCTAAGCTTGGGAATACGAATACAT
TTGCGTCACCTTTAATTTGTGCATCTGGTGCTTTTTTAGCCGCCACTTCTGGAACGATTG
CTGCGTCGAATTGGAATTCACCATCGATAACAACATCTTTTAAGCCTTCTGATTCAGCTT

TTTCTTGTGCAGCTTTTGTTGCTTCCGCTACTTTTTCAACATCGTCTGATTTTGCTGAAC
CTTTTGTTGAGAAGCTTAATAATGCTACTTTAGGATCCATGCCGAAGCTTTTCGCTGTTT
TTGCACTTTCTACCGCAATTTCAGCTAAGTCTGTTGCTTCTAATGTAGGGTTGATTGCAC
AATCACCGAAAACATATTGTTCTTCGCCTTTAATCATGAAGAACACACCTGATGTTTTCG
CAACACCTGGTTTAGTTTTGATGATTTGTAGTGCTGGACGAACTGTATCGCCTGTTGAGT
GTGCAGCACCGCTGACTAGTCCTTCAGCTTTACCTGCATAAACAAGCATTGTACCGAAGT
AGTTTACATTGTTAAGCATATCTTCAGCTTGTTCTTTTGTCGCTTTGCCTTTACGACGTT
CTACAAATGCGTCTACTAATTCTGGTTTCAAGTCGCGACTAAAACTTCTTCGTCTTTCGC
AGTTGGAATATTTTTACCTACAAAGTCAGCGCGTATTGGTAATTCGCGGTGTCCGCGATC
CACTAATGTTGCTAACCCGATTTTTTGTGGTCGAGAATGTAGTAAGATTGCATCGAGTGA
CGCACGTACAGTTCGACCCGTATAAAGCACATCATCAATGATGATAACGACTTTGTCTTT
AAGGTCTACATCAATGACATAAGACTTCTCAGACATTTTAGGTGTCGGTAACTCTAAATC
ATCTCTGAATTGCGTGATGTCTAATGTTCCTGTAGGAACACTTACGTCTTCAATTTGTTG
AATCTTCATTTGAATACGTTTCGCTAAAAATTCTCCGCGTGTTTTCACACCAAGCAACAC
AAGATTTTCAGTACCTTTGTTATACTCTAGAATTTCATGTGCGATACGTGTTAAAGTCCG
CTGCATGCCTGCATCATCTAATACAATACGCGACTTGGCAAAGCGAAGAAACTTTTAAGC
CAGCTTCTTCTAATCGTTTACGGCCTTGTTGGAAACTTTTTTCCACGACAATACCGATAC
CGACTGTTTCTGCACCTGCTTGTTCAACTAAACGATTCAAACCAAGTGCCGCATCTCCAT
TCGCTAAAAAGTCATCGATAATCAATACACGGTCATTTTCCCCTAAAAATTCTTCAGAGA
TAATGACGCGATTCGTCTTGTTTTTCGTAAACGAATGAATATCTGTCTGATAAAATCCAT
CTGCCAAAGTACTTGGCTTAGCCTTCTTCGCAAAGAGACATGGTACTTCAAAACGATATG
CTGCCATAATAGCTGGCGCAATGCCTGATGCTTCAACAGTTAAGATTTTAGTGATGCCTT
GATCTTTGAACTGTTCATAAAATGTTTCGCCAATGTCATACATTAACACTGCATCAATTT
GATGGTTTAAAAACCCATCTACCTTCAAGATTTTTTCGCCAATGACGACGCCGTCTTCTT
TAACCTTCTACCTACTAGTGAACCTACAACACGAATACCGTCTAATACTAAACGTGGAAT
TGATAAGTCCATTGATTCTGTTGGTAAGCCGACTGCGACTACAGTACCGCCAGCACGTAC
AGATTCAACAGCTGCGTTGAAGGCTTGTTTAGCAACCGCTGTAACAACTGCCGCATGTGC
ACCGCCTACATGTTCTTGAATGTATTTTGCGACATCCACTTCTTTAGGGTTTAAAGTGAC
ATCTGCATCGTATTGTTTCGCAAATTCAAGTTGACTGTCATTGATGTCTACTGCGATAAC
TTTAGCGTTAAATACTTTTTTCGCATATTGTAAAGCTAGGTTTCCAAGTCCACCTAAACC
GAATAAAACAAGCCATTCACCAGCACGTACGCCTGATTCTTTAATTGCTTTGTATGTTGT
GACACCTGCACATGTGATACTGCTGGCAGCTTCTGATGATAAACCATCTGGTTTTTCCAA
CCTTTATATAATTTTTCTCTTTCTTTTTCATCCATTTCAGGCTCGAAAGCTTGATCTAAT
TTCCAACGGTTCGCAATTTCTGCTTTACTATCCCAGAAACCTACCGCAAGTCCTGCAAGA
TAAGCAGCACCTAACGCAGTCGTTTCATTGATTTCAGGACGTTCTACTGTAATATTTTGA
ATATCTGCTTGGAACTGCATTAAGAAGTTGTTCTTAACAGCACCACCATCTACACGGATA
TTCTTCACTTCAATACCTGAGTCTTGAATCATCGCTTCAACCACATCACGTGTTTGGTAA
CAAATAGATTCTAACGTTGCTCTGATAAAGTGTTCTTTCTCAGTCGCACGCGTAATACCA
AATACTGCACCACGCGCATTCGCATCCCAATATGGTGTACCGAGTCCAACAAACGCCGGT
ACCACATAAACACCATCACTTGATTCAATACGGTTCGCATAGTTTTCAGATTCGGGTGCT
GAGTTAATCATACGTAAACCATCTCGCAACCATTGAATAGCTGAACCAGAGACGAAAATA
GAACCTTCTAATGCATAATTCACTTCGCCATCTAAGCCGAATGCGATTGTTGTTAATAAA
CCGTTATCCGATTGAATCGCTTCTTTACCAGTGTTCATTAACATGAATCCACCCGTACCA
TATGTGTTTTTCACTTCACCACGTTCGAAGCATGCTTGTCCGAATAAGGCTGCTTGCTGA
TCCCCTGCGATTCCCGCAATCGGTACTTCTTGTCCAAAGAAATGATAATCTTTTGTTTTC
GCATAAACCTCACTTTTCTTTTGCATGGTTAATCGCTTCAATTGTTTGAGGCATAACACC
GTCGTCTGCTGCAACCACTAAGATTGTGATATCAGTGACTTGCGCACCACGTGCACGCAT
AGTAGTGAAGGCTGCGTGTCCAGGTGTATCTAAGAAAGTGATTTTTTTGCCGTCATTTGT
GATTTGGTAAGCACCGATATGTTGTGTGATACCGCCCGCTTCACCTTGTGTCACATGTGT
ATTTCTGATTGAGTCAAGTAGCGTTGTTTTACCATGGTCAACGTGTCCCATGATTGTTAC
AACTGCTGGACGCTCAATTGCATCCGGATCTTCTTCTTCATCATCGAAGTAGATATCTAA
GTCTTCTTCGTCAATGACTACTTCTTTTTCTACTTCTACACCATAATCATCAGCGATTAA
CTCAATTGTTTCATCGTCTAATGATTGGTTAATGTTTGCCATAATACCAAGTAAGAATAA
TTTCTTAACAATACTTGATGACTCAATATTCATTTTGTCAGCTAATTCGCCAACCGTAAT
ACCTTCTAAATAAGTAATTTTTTCTGGCATTTCTTTTTTCGGTTGTTCTGGTGCTGGTTT
TTGATTATTGTTTTTCTTATGTTTGTTGTTGT
>NAS-3

GATTGCTTGAATACGTTCTTTAGCAAGTTCTACTTCATCGTTTACCACAACGTAGTCATA
AAGATTCATCATTTCCACTTCTTTACGTGCTTCTTTCACACGACTTTGAATCTTTTCGTC
TGATTCTGTACCGCGGCCGACTAAACGCTCTCTTAAGTGGTCTAAACTTGGCGGTGCTAG
GAAGATAAATAATGCATCAGGGAATTTCTTGCGTACTTGTTTCGCACCTTCAACTTCGAT
TTCTAAGAAGACATCATAGCCTTCATCCATAGTGTCTTTCACATACTGTACAGGTGTACC
GTAGTAGTTACCTACATATTCTGCATACTCAATAAATTGATCTTGTGCGATAAGTTGCTC
AAACTCTCGTTTTGTCTTGAAAAAATAATCAACGCCATCTTGTTCACCTTCACGCATTTC
ACGTGTCGTCATTGAAATTGAATATTTGTATGATGTTTTCGGATCATCAAATATTTCTTT
GCGGACAGTACCTTTTCCGACTCCAGATGGACCTGATAAGACAATTAGTAAACCTTTTTC
ATTATCCATGCCTTACGTTTAAACCTTGTAAAACAGGTCCGATTGCTTCATATCCGCCTA
AACGTTGCGCAATTTTGTAACCGATGTTACCTGCTTCTAAGCTTGGGAATACGAATACAT
TTGCGTCACCTTTAATTTGTGCATCTGGTGCTTTTTTAGCCGCCACTTCTGGAACGATTG
CTGCGTCGAATTGGAATTCACCATCGATAACAACATCTTTTAAGCCTTCTGATTCAGCTT
TTTCTTGTGCAGCTTTTGTTGCTTCCGCTACTTTTTCAACATCGTCTGATTTTGCTGAAC
CTTTTGTTGAGAAGCTTAATAATGCTACTTTAGGATCCATGCCGAAGCTTTTCGCTGTTT
TTGCACTTTCTACCGCAATTTCAGCTAAGTCTGTTGCTTCTAATGTAGGGTTGATTGCAC
AATCACCGAAAACATATTGTTCTTCGCCTTTAATCATGAAGAACACACCTGATGTTTTCG
CAACACCTGGTTTAGTTTTGATGATTTGTAGTGCTGGACGAACTGTATCGCCTGTTGAGT
GTGCAGCACCGCTGACTAGTCCTTCAGCTTTACCTGCATAAACAAGCATTGTACCGAAGT
AGTTTACATTGTTAAGCATATCTTCAGCTTGTTCTTTTGTCGCTTTGCCTTTACGACGTT
CTACAAATGCGTCTACTAATTCTGGTTTCAAGTCGCGACTAAAACTTCTTCGTCTTTCGC
AGTTGGAATATTTTTACCTACAAAGTCAGCGCGTATTGGTAATTCGCGGTGTCCGCGATC
CACTAATGTTGCTAACCCGATTTTTTGTGGTCGAGAATGTAGTAAGATTGCATCGAGTGA
CGCACGTACAGTTCGACCCGTATAAAGCACATCATCAATGATGATAACGACTTTGTCTTT
AAGGTCTACATCAATGACATAAGACTTCTCAGACATTTTAGGTGTCGGTAACTCTAAATC
ATCTCTGAATTGCGTGATGTCTAATGTTCCTGTAGGAACACTTACGTCTTCAATTTGTTG
AATCTTCATTTGAATACGTTTCGCTAAAAATTCTCCGCGTGTTTTCACACCAAGCAACAC
AAGATTTTCAGTACCTTTGTTATACTCTAGAATTTCATGTGCGATACGTGTTAAAGTCCG
CTGCATGCCTGCATCATCTAATACAATACGCGACTTGGCAAAGCGAAGAAACTTTTAAGC
CAGCTTCTTCTAATCGTTTACGGCCTTGTTGGAAACTTTTTTCCACGACAATACCGATAC
CGACTGTTTCTGCACCTGCTTGTTCAACTAAACGATTCAAACCAAGTGCCGCATCTCCAT
TCGCTAAAAAGTCATCGATAATCAATACACGGTCATTTTCCCCTAAAAATTCTTCAGAGA
TAATGACGCGATTCGTCTTGTTTTTCGTAAACGAATGAATATCTGTCTGATAAAATCCAT
CTGCCAAAGTACTTGGCTTAGCCTTCTTCGCAAAGAGACATGGTACTTCAAAACGATATG
CTGCCATAATAGCTGGCGCAATGCCTGATGCTTCAACAGTTAAGATTTTAGTGATGCCTT
GATCTTTGAACTGTTCATAAAATGTTTCGCCAATGTCATACATTAACACTGCATCAATTT
GATGGTTTAAAAACCCATCTACCTTCAAGATTTTTTCGCCAATGACGACGCCGTCTTCTT
TAACCTTCTACCTACTAGTGAACCTACAACACGAATACCGTCTAATACTAAACGTGGAAT
TGATAAGTCCATTGATTCTGTTGGTAAGCCGACTGCGACTACAGTACCGCCAGCACGTAC
AGATTCAACAGCTGCGTTGAAGGCTTGTTTAGCAACCGCTGTAACAACTGCCGCATGTGC
ACCGCCTACATGTTCTTGAATGTATTTTGCGACATCCACTTCTTTAGGGTTTAAAGTGAC
ATCTGCATCGTATTGTTTCGCAAATTCAAGTTGACTGTCATTGATGTCTACTGCGATAAC
TTTAGCGTTAAATACTTTTTTCGCATATTGTAAAGCTAGGTTTCCAAGTCCACCTAAACC
GAATAAAACAAGCCATTCACCAGCACGTACGCCTGATTCTTTAATTGCTTTGTATGTTGT
GACACCTGCACATGTGATACTGCTGGCAGCTTCTGATGATAAACCATCTGGTTTTTCCAA
CCTTTATATAATTTTTCTCTTTCTTTTTCATCCATTTCAGGCTCGAAAGCTTGATCTAAT
TTCCAACGGTTCGCAATTTCTGCTTTACTATCCCAGAAACCTACCGCAAGTCCTGCAAGA
TAAGCAGCACCTAACGCAGTCGTTTCATTGATTTCAGGACGTTCTACTGTAATATTTTGA
ATATCTGCTTGGAACTGCATTAAGAAGTTGTTCTTAACAGCACCACCATCTACACGGATA
TTCTTCACTTCAATACCTGAGTCTTGAATCATCGCTTCAACCACATCACGTGTTTGGTAA
CAAATAGATTCTAACGTTGCTCTGATAAAGTGTTCTTTCTCAGTCGCACGCGTAATACCA
AATACTGCACCACGCGCATTCGCATCCCAATATGGTGTACCGAGTCCAACAAACGCCGGT
ACCACATAAACACCATCACTTGATTCAATACGGTTCGCATAGTTTTCAGATTCGGGTGCT
GAGTTAATCATACGTAAACCATCTCGCAACCATTGAATAGCTGAACCAGAGACGAAAATA
GAACCTTCTAATGCATAATTCACTTCGCCATCTAAGCCGAATGCGATTGTTGTTAATAAA
CCGTTATCCGATTGAATCGCTTCTTTACCAGTGTTCATTAACATGAATCCACCCGTACCA
TATGTGTTTTTCACTTCACCACGTTCGAAGCATGCTTGTCCGAATAAGGCTGCTTGCTGA

TCCCCTGCGATTCCCGCAATCGGTACTTCTTGTCCAAAGAAATGATAATCTTTTGTTTTC
GCATAAACCTCACTTTTCTTTTGCATGGTTAATCGCTTCAATTGTTTGAGGCATAACACC
GTCGTCTGCTGCAACCACTAAGATTGTGATATCAGTGACTTGCGCACCACGTGCACGCAT
AGTAGTGAAGGCTGCGTGTCCAGGTGTATCTAAGAAAGTGATTTTTTTGCCGTCATTTGT
GATTTGGTAAGCACCGATATGTTGTGTGATACCGCCCGCTTCACCTTGTGTCACATGTGT
ATTTCTGATTGAGTCAAGTAGCGTTGTTTTACCATGGTCAACGTGTCCCATGATTGTTAC
AACTGCTGGACGCTCAATTGCATCCGGATCTTCTTCTTCATCATCGAAGTAGATATCTAA
GTCTTCTTCGTCAATGACTACTTCTTTTTCTACTTCTACACCATAATCATCAGCGATTAA
CTCAATTGTTTCATCGTCTAATGATTGGTTAATGTTTGCCATAATACCAAGTAAGAATAA
TTTCTTAACAATACTTGATGACTCAATATTCATTTTGTCAGCTAATTCGCCAACCGTAAT
ACCTTCTAAATAAGTAATTTTTTCTGGCATTTCTTTTTTCGGTTGTTCTGGTGCTGGTTT
TTGATTATTGTTTTTCTTATGTTTGTTGTTGT
>NAS-13
GATTGCTTGAATACGTTCTTTAGCAAGTTCTACTTCATCGTTTACCACAACGTAGTCATA
AAGATTCATCATTTCCACTTCTTTACGTGCTTCTTTCACACGACTTTGAATCTTTTCGTC
TGATTCTGTACCGCGGCCGACTAAACGCTCTCTTAAGTGGTCTAAACTTGGCGGTGCTAG
GAAGATAAATAATGCATCAGGGAATTTCTTGCGTACTTGTTTCGCACCTTCAACTTCGAT
TTCTAAGAAGACATCATAGCCTTCATCCATAGTGTCTTTCACATACTGTACAGGTGTACC
GTAGTAGTTACCTACATATTCTGCATACTCAATAAATTGATCTTGTGCGATAAGTTGCTC
AAACTCTCGTTTTGTCTTGAAAAAATAATCAACGCCATCTTGTTCACCTTCACGCATTTC
ACGTGTCGTCATTGAAATTGAATATTTGTATGATGTTTTCGGATCATCAAATATTTCTTT
GCGGACAGTACCTTTTCCGACTCCAGATGGACCTGATAAGACAATTAGTAAACCTTTTTC
ATTATCCATGCCTTACGTTTAAACCTTGTAAAACAGGTCCGATTGCTTCATATCCGCCTA
AACGTTGCGCAATTTTGTAACCGATGTTACCTGCTTCTAAGCTTGGGAATACGAATACAT
TTGCGTCACCTTTAATTTGTGCATCTGGTGCTTTTTTAGCCGCCACTTCTGGAACGATTG
CTGCGTCGAATTGGAATTCACCATCGATAACAACATCTTTTAAGCCTTCTGATTCAGCTT
TTTCTTGTGCAGCTTTTGTTGCTTCCGCTACTTTTTCAACATCGTCTGATTTTGCTGAAC
CTTTTGTTGAGAAGCTTAATAATGCTACTTTAGGATCCATGCCGAAGCTTTTCGCTGTTT
TTGCACTTTCTACCGCAATTTCAGCTAAGTCTGTTGCTTCTAATGTAGGGTTGATTGCAC
AATCACCGAAAACATATTGTTCTTCGCCTTTAATCATGAAGAACACACCTGATGTTTTCG
CAACACCTGGTTTAGTTTTGATGATTTGTAGTGCTGGACGAACTGTATCGCCTGTTGAGT
GTGCAGCACCGCTGACTAGTCCTTCAGCTTTACCTGCATAAACAAGCATTGTACCGAAGT
AGTTTACATTGTTAAGCATATCTTCAGCTTGTTCTTTTGTCGCTTTGCCTTTACGACGTT
CTACAAATGCGTCTACTAATTCTGGTTTCAAGTCGCGACTAAAACTTCTTCGTCTTTCGC
AGTTGGAATATTTTTACCTACAAAGTCAGCGCGTATTGGTAATTCGCGGTGTCCGCGATC
CACTAATGTTGCTAACCCGATTTTTTGTGGTCGAGAATGTAGTAAGATTGCATCGAGTGA
CGCACGTACAGTTCGACCCGTATAAAGCACATCATCAATGATGATAACGACTTTGTCTTT
AAGGTCTACATCAATGACATAAGACTTCTCAGACATTTTAGGTGTCGGTAACTCTAAATC
ATCTCTGAATTGCGTGATGTCTAATGTTCCTGTAGGAACACTTACGTCTTCAATTTGTTG
AATCTTCATTTGAATACGTTTCGCTAAAAATTCTCCGCGTGTTTTCACACCAAGCAACAC
AAGATTTTCAGTACCTTTGTTATACTCTAGAATTTCATGTGCGATACGTGTTAAAGTCCG
CTGCATGCCTGCATCATCTAATACAATACGCGACTTGGCAAAGCGAAGAAACTTTTAAGC
CAGCTTCTTCTAATCGTTTACGGCCTTGTTGGAAACTTTTTTCCACGACAATACCGATAC
CGACTGTTTCTGCACCTGCTTGTTCAACTAAACGATTCAAACCAAGTGCCGCATCTCCAT
TCGCTAAAAAGTCATCGATAATCAATACACGGTCATTTTCCCCTAAAAATTCTTCAGAGA
TAATGACGCGATTCGTCTTGTTTTTCGTAAACGAATGAATATCTGTCTGATAAAATCCAT
CTGCCAAAGTACTTGGCTTAGCCTTCTTCGCAAAGAGACATGGTACTTCAAAACGATATG
CTGCCATAATAGCTGGCGCAATGCCTGATGCTTCAACAGTTAAGATTTTAGTGATGCCTT
GATCTTTGAACTGTTCATAAAATGTTTCGCCAATGTCATACATTAACACTGCATCAATTT
GATGGTTTAAAAACCCATCTACCTTCAAGATTTTTTCGCCAATGACGACGCCGTCTTCTT
TAACCTTCTACCTACTAGTGAACCTACAACACGAATACCGTCTAATACTAAACGTGGAAT
TGATAAGTCCATTGATTCTGTTGGTAAGCCGACTGCGACTACAGTACCGCCAGCACGTAC
AGATTCAACAGCTGCGTTGAAGGCTTGTTTAGCAACCGCTGTAACAACTGCCGCATGTGC
ACCGCCTACATGTTCTTGAATGTATTTTGCGACATCCACTTCTTTAGGGTTTAAAGTGAC
ATCTGCATCGTATTGTTTCGCAAATTCAAGTTGACTGTCATTGATGTCTACTGCGATAAC
TTTAGCGTTAAATACTTTTTTCGCATATTGTAAAGCTAGGTTTCCAAGTCCACCTAAACC
GAATAAAACAAGCCATTCACCAGCACGTACGCCTGATTCTTTAATTGCTTTGTATGTTGT

GACACCTGCACATGTGATACTGCTGGCAGCTTCTGATGATAAACCATCTGGTTTTTCCAA
CCTTTATATAATTTTTCTCTTTCTTTTTCATCCATTTCAGGCTCGAAAGCTTGATCTAAT
TTCCAACGGTTCGCAATTTCTGCTTTACTATCCCAGAAACCTACCGCAAGTCCTGCAAGA
TAAGCAGCACCTAACGCAGTCGTTTCATTGATTTCAGGACGTTCTACTGTAATATTTTGA
ATATCTGCTTGGAACTGCATTAAGAAGTTGTTCTTAACAGCACCACCATCTACACGGATA
TTCTTCACTTCAATACCTGAGTCTTGAATCATCGCTTCAACCACATCACGTGTTTGGTAA
CAAATAGATTCTAACGTTGCTCTGATAAAGTGTTCTTTCTCAGTCGCACGCGTAATACCA
AATACTGCACCACGCGCATTCGCATCCCAATATGGTGTACCGAGTCCAACAAACGCCGGT
ACCACATAAACACCATCACTTGATTCAATACGGTTCGCATAGTTTTCAGATTCGGGTGCT
GAGTTAATCATACGTAAACCATCTCGCAACCATTGAATAGCTGAACCAGAGACGAAAATA
GAACCTTCTAATGCATAATTCACTTCGCCATCTAAGCCGAATGCGATTGTTGTTAATAAA
CCGTTATCCGATTGAATCGCTTCTTTACCAGTGTTCATTAACATGAATCCACCCGTACCA
TATGTGTTTTTCACTTCACCACGTTCGAAGCATGCTTGTCCGAATAAGGCTGCTTGCTGA
TCCCCTGCGATTCCCGCAATCGGTACTTCTTGTCCAAAGAAATGATAATCTTTTGTTTTC
GCATAAACCTCACTTTTCTTTTGCATGGTTAATCGCTTCAATTGTTTGAGGCATAACACC
GTCGTCTGCTGCAACCACTAAGATTGTGATATCAGTGACTTGCGCACCACGTGCACGCAT
AGTAGTGAAGGCTGCGTGTCCAGGTGTATCTAAGAAAGTGATTTTTTTGCCGTCATTTGT
GATTTGGTAAGCACCGATATGTTGTGTGATACCGCCCGCTTCACCTTGTGTCACATGTGT
ATTTCTGATTGAGTCAAGTAGCGTTGTTTTACCATGGTCAACGTGTCCCATGATTGTTAC
AACTGCTGGACGCTCAATTGCATCCGGATCTTCTTCTTCATCATCGAAGTAGATATCTAA
GTCTTCTTCGTCAATGACTACTTCTTTTTCTACTTCTACACCATAATCATCAGCGATTAA
CTCAATTGTTTCATCGTCTAATGATTGGTTAATGTTTGCCATAATACCAAGTAAGAATAA
TTTCTTAACAATACTTGATGACTCAATATTCATTTTGTCAGCTAATTCGCCAACCGTAAT
ACCTTCTAAATAAGTAATTTTTTCTGGCATTTCTTTTTTCGGTTGTTCTGGTGCTGGTTT
TTGATTATTGTTTTTCTTATGTTTGTTGTTGT
>NAS-15
GATTGCTTGAATACGTTCTTTAGCAAGTTCTACTTCATCGTTTACCACAACGTAGTCATA
AAGATTCATCATTTCCACTTCTTTACGTGCTTCTTTCACACGACTTTGAATCTTTTCGTC
TGATTCTGTACCGCGGCCGACTAAACGCTCTCTTAAGTGGTCTAAACTTGGCGGTGCTAG
GAAGATAAATAATGCATCAGGGAATTTCTTGCGTACTTGTTTCGCACCTTCAACTTCGAT
TTCTAAGAAGACATCATAGCCTTCATCCATAGTGTCTTTCACATACTGTACAGGTGTACC
GTAGTAGTTACCTACATATTCTGCATACTCAATAAATTGATCTTGTGCGATAAGTTGCTC
AAACTCTCGTTTTGTCTTGAAAAAATAATCAACGCCATCTTGTTCACCTTCACGCATTTC
ACGTGTCGTCATTGAAATTGAATATTTGTATGATGTTTTCGGATCATCAAATATTTCTTT
GCGGACAGTACCTTTTCCGACTCCAGATGGACCTGATAAGACAATTAGTAAACCTTTTTC
ATTATCCATGCCTTACGTTTAAACCTTGTAAAACAGGTCCGATTGCTTCATATCCGCCTA
AACGTTGCGCAATTTTGTAACCGATGTTACCTGCTTCTAAGCTTGGGAATACGAATACAT
TTGCGTCACCTTTAATTTGTGCATCTGGTGCTTTTTTAGCCGCCACTTCTGGAACGATTG
CTGCGTCGAATTGGAATTCACCATCGATAACAACATCTTTTAAGCCTTCTGATTCAGCTT
TTTCTTGTGCAGCTTTTGTTGCTTCCGCTACTTTTTCAACATCGTCTGATTTTGCTGAAC
CTTTTGTTGAGAAGCTTAATAATGCTACTTTAGGATCCATGCCGAAGCTTTTCGCTGTTT
TTGCACTTTCTACCGCAATTTCAGCTAAGTCTGTTGCTTCTAATGTAGGGTTGATTGCAC
AATCACCGAAAACATATTGTTCTTCGCCTTTAATCATGAAGAACACACCTGATGTTTTCG
CAACACCTGGTTTAGTTTTGATGATTTGTAGTGCTGGACGAACTGTATCGCCTGTTGAGT
GTGCAGCACCGCTGACTAGTCCTTCAGCTTTACCTGCATAAACAAGCATTGTACCGAAGT
AGTTTACATTGTTAAGCATATCTTCAGCTTGTTCTTTTGTCGCTTTGCCTTTACGACGTT
CTACAAATGCGTCTACTAATTCTGGTTTCAAGTCGCGACTAAACTTCTTCGTCTTTCGCA
GTTGGAATATTTTTACCTACAAAGTCAGCGCGTATTGGTAATTCGCGGTGTCCGCGATCC
ACTAATGTTGCTAACCCGATTTTTTGTGGTCGAGAATGTAGTAAGATTGCATCGAGTGAC
GCACGTACAGTTCGACCCGTATAAAGCACATCATCAATGATGATAACGACTTTGTCTTTA
AGGTCTACATCAATGACATAAGACTTCTCAGACATTTTAGGTGTCGGTAACTCTAAATCA
TCTCTGAATTGCGTGATGTCTAATGTTCCTGTAGGAACACTTACGTCTTCAATTTGTTGA
ATCTTCATTTGAATACGTTTCGCTAAAAATTCTCCGCGTGTTTTCACACCAAGCAACACA
AGATTTTCAGTACCTTTGTTATACTCTAGAATTTCATGTGCGATACGTGTTAAAGTCCGC
TGCATGCCTGCATCATCTAATACAATACGCGACTTGGCAAAGCGAAGAAACTTTTAAGCC
AGCTTCTTCTAATCGTTTACGGCCTTGTTGGAAACTTTTTTCCACGACAATACCGATACC
GACTGTTTCTGCACCTGCTTGTTCAACTAAACGATTCAAACCAAGTGCCGCATCTCCATT

CGCTAAAAAGTCATCGATAATCAATACACGGTCATTTTCCCCTAAAAATTCTTCAGAGAT
AATGACGCGATTCGTCTTGTTTTTCGTAAACGAATGAATATCTGTCTGATAAAATCCATC
TGCCAAAGTACTTGGCTTAGCCTTCTTCGCAAAGAGACATGGTACTTCAAAACGATATGC
TGCCATAATAGCTGGCGCAATGCCTGATGCTTCAACAGTTAAGATTTTAGTGATGCCTTG
ATCTTTGAACTGTTCATAAAATGTTTCGCCAATGTCATACATTAACACTGCATCAATTTG
ATGGTTTAAAAACCCATCTACCTTCAAGATTTTTTCGCCAATGACGACGCCGTCTTCTTT
AACCTTCTACCTACTAGTGAACCTACAACACGAATACCGTCTAATACTAAACGTGGAATT
GATAAGTCCATTGATTCTGTTGGTAAGCCGACTGCGACTACAGTACCGCCAGCACGTACA
GATTCAACAGCTGCGTTGAAGGCTTGTTTAGCAACCGCTGTAACAACTGCCGCATGTGCA
CCGCCTACATGTTCTTGAATGTATTTTGCGACATCCACTTCTTTAGGGTTTAAAGTGACA
TCTGCATCGTATTGTTTCGCAAATTCAAGTTGACTGTCATTGATGTCTACTGCGATAACT
TTAGCGTTAAATACTTTTTTCGCATATTGTAAAGCTAGGTTTCCAAGTCCACCTAAACCG
AATAAAACAAGCCATTCACCAGCACGTACGCCTGATTCTTTAATTGCTTTGTATGTTGTG
ACACCTGCACATGTGATACTGCTGGCAGCTTCTGATGATAAACCATCTGGTTTTTCCAAC
CTTTATATAATTTTTCTCTTTCTTTTTCATCCATTTCAGGCTCGAAAGCTTGATCTAATT
TCCAACGGTTCGCAATTTCTGCTTTACTATCCCAGAAACCTACCGCAAGTCCTGCAAGAT
AAGCAGCACCTAACGCAGTCGTTTCATTGATTTCAGGACGTTCTACTGTAATATTTTGAA
TATCTGCTTGGAACTGCATTAAGAAGTTGTTCTTAACAGCACCACCATCTACACGGATAT
TCTTCACTTCAATACCTGAGTCTTGAATCATCGCTTCAACCACATCACGTGTTTGGTAAC
AAATAGATTCTAACGTTGCTCTGATAAAGTGTTCTTTCTCAGTCGCACGCGTAATACCAA
ATACTGCACCACGCGCATTCGCATCCCAATATGGTGTACCGAGTCCAACAAACGCCGGTA
CCACATAAACACCATCACTTGATTCAATACGGTTCGCATAGTTTTCAGATTCGGGTGCTG
AGTTAATCATACGTAAACCATCTCGCAACCATTGAATAGCTGAACCAGAGACGAAAATAG
AACCTTCTAATGCATAATTCACTTCGCCATCTAAGCCGAATGCGATTGTTGTTAATAAAC
CGTTATCCGATTGAATCGCTTCTTTACCAGTGTTCATTAACATGAATCCACCCGTACCAT
ATGTGTTTTTCACTTCACCACGTTCGAAGCATGCTTGTCCGAATAAGGCTGCTTGCTGAT
CCCCTGCGATTCCCGCAATCGGTACTTCTTGTCCAAAGAAATGATAATCTTTTGTTTTCG
CATAAACCTCACTTTTCTTTTGCATGGTTAATCGCTTCAATTGTTTGAGGCATAACACCG
TCGTCTGCTGCAACCACTAAGATTGTGATATCAGTGACTTGCGCACCACGTGCACGCATA
GTAGTGAAGGCTGCGTGTCCAGGTGTATCTAAGAAAGTGATTTTTTTGCCGTCATTTGTG
ATTTGGTAAGCACCGATATGTTGTGTGATACCGCCCGCTTCACCTTGTGTCACATGTGTA
TTTCTGATTGAGTCAAGTAGCGTTGTTTTACCATGGTCAACGTGTCCCATGATTGTTACA
ACTGCTGGACGCTCAATTGCATCCGGATCTTCTTCTTCATCATCGAAGTAGATATCTAAG
TCTTCTTCGTCAATGACTACTTCTTTTTCTACTTCTACACCATAATCATCAGCGATTAAC
TCAATTGTTTCATCGTCTAATGATTGGTTAATGTTTGCCATAATACCAAGTAAGAATAAT
TTCTTAACAATACTTGATGACTCAATATTCATTTTGTCAGCTAATTCGCCAACCGTAATA
CCTTCTAAATAAGTAATTTTTTCTGGCATTTCTTTTTTCGGTTGTTCTGGTGCTGGTTTT
TGATTATTGTTTTTCTTATGTTTGTTGTTGT
>NAS-7
GATTGCTTGAATACGTTCTTTAGCAAGTTCTACTTCATCGTTTACCACAACGTAGTCATA
AAGATTCATCATTTCCACTTCTTTACGTGCTTCTTTCACACGACTTTGAATCTTTTCGTC
TGATTCTGTACCGCGGCCGACTAAACGCTCTCTTAAGTGGTCTAAACTTGGCGGTGCTAG
GAAGATAAATAATGCATCAGGGAATTTCTTGCGTACTTGTTTCGCACCTTCAACTTCGAT
TTCTAAGAAGACATCATAGCCTTCATCCATAGTGTCTTTCACATACTGTACAGGTGTACC
GTAGTAGTTACCTACATATTCTGCATACTCAATAAATTGATCTTGTGCGATAAGTTGCTC
AAACTCTCGTTTTGTCTTGAAAAAATAATCAACGCCATCTTGTTCACCTTCACGCATTTC
ACGTGTCGTCATTGAAATTGAATATTTGTATGATGTTTTCGGATCATCAAATATTTCTTT
GCGGACAGTACCTTTTCCGACTCCAGATGGACCTGATAAGACAATTAGTAAACCTTTTTC
ATTATCCATGCCTTACGTTTAAACCTTGTAAAACAGGTCCGATTGCTTCATATCCGCCTA
AACGTTGCGCAATTTTGTAACCGATGTTACCTGCTTCTAAGCTTGGGAATACGAATACAT
TTGCGTCACCTTTAATTTGTGCATCTGGTGCTTTTTTAGCCGCCACTTCTGGAACGATTG
CTGCGTCGAATTGGAATTCACCATCGATAACAACATCTTTTAAGCCTTCTGATTCAGCTT
TTTCTTGTGCAGCTTTTGTTGCTTCCGCTACTTTTTCAACATCGTCTGATTTTGCTGAAC
CTTTTGTTGAGAAGCTTAATAATGCTACTTTAGGATCCATGCCGAAGCTTTTCGCTGTTT
TTGCACTTTCTACCGCAATTTCAGCTAAGTCTGTTGCTTCTAATGTAGGGTTGATTGCAC
AATCACCGAAAACATATTGTTCTTCGCCTTTAATCATGAAGAACACACCTGATGTTTTCG
CAACACCTGGTTTAGTTTTGATGATTTGTAGTGCTGGACGAACTGTATCGCCTGTTGAGT

GTGCAGCACCGCTGACTAGTCCTTCAGCTTTACCTGCATAAACAAGCATTGTACCGAAGT
AGTTTACATTGTTAAGCATATCTTCAGCTTGTTCTTTTGTCGCTTTGCCTTTACGACGTT
CTACAAATGCGTCTACTAATTCTGGTTTCAAGTCGCGACTAAAACTTCTTCGTCTTTCGC
AGTTGGAATATTTTTACCTACAAAGTCAGCGCGTATTGGTAATTCGCGGTGTCCGCGATC
CACTAATGTTGCTAACCCGATTTTTTGTGGTCGAGAATGTAGTAAGATTGCATCGAGTGA
CGCACGTACAGTTCGACCCGTATAAAGCACATCATCAATGATGATAACGACTTTGTCTTT
AAGGTCTACATCAATGACATAAGACTTCTCAGACATTTTAGGTGTCGGTAACTCTAAATC
ATCTCTGAATTGCGTGATGTCTAATGTTCCTGTAGGAACACTTACGTCTTCAATTTGTTG
AATCTTCATTTGAATACGTTTCGCTAAAAATTCTCCGCGTGTTTTCACACCAAGCAACAC
AAGATTTTCAGTACCTTTGTTATACTCTAGAATTTCATGTGCGATACGTGTTAAAGTCCG
CTGCATGCCTGCATCATCTAATACAATACGCGACTTGGCAAAGCGAAGAAACTTTTAAGC
CAGCTTCTTCTAATCGTTTACGGCCTTGTTGGAAACTTTTTTCCACGACAATACCGATAC
CGACTGTTTCTGCACCTGCTTGTTCAACTAAACGATTCAAACCAAGTGCCGCATCTCCAT
TCGCTAAAAAGTCATCGATAATCAATACACGGTCATTTTCCCCTAAAAAATTCTTCAGAGA
TAATGACGCGATTCGTCTTGTTTTTCGTAAACGAATGAATATCTGTCTGATAAAATCCAT
CTGCCAAAGTACTTGGCTTAGCCTTCTTCGCAAAGAGACATGGTACTTCAAAACGATATG
CTGCCATAATAGCTGGCGCAATGCCTGATGCTTCAACAGTTAAGATTTTAGTGATGCCTT
GATCTTTGAACTGTTCATAAAATGTTTCGCCAATGTCATACATTAACACTGCATCAATTT
GATGGTTTAAAAACCCATCTACCTTCAAGATTTTTTCGCCAATGACGACGCCGTCTTCTT
TAACCTTCTACCTACTAGTGAACCTACAACACGAATACCGTCTAATACTAAACGTGGAAT
TGATAAGTCCATTGATTCTGTTGGTAAGCCGACTGCGACTACAGTACCGCCAGCACGTAC
AGATTCAACAGCTGCGTTGAAGGCTTGTTTAGCAACCGCTGTAACAACTGCCGCATGTGC
ACCGCCTACATGTTCTTGAATGTATTTTGCGACATCCACTTCTTTAGGGTTTAAAGTGAC
ATCTGCATCGTATTGTTTCGCAAATTCAAGTTGACTGTCATTGATGTCTACTGCGATAAC
TTTAGCGTTAAATACTTTTTTCGCATATTGTAAAGCTAGGTTTCCAAGTCCACCTAAACC
GAATAAAACAAGCCATTCACCAGCACGTACGCCTGATTCTTTAATTGCTTTGTATGTTGT
GACACCTGCACATGTGATACTGCTGGCAGCTTCTGATGATAAACCATCTGGTTTTTCCAA
CCTTTATATAATTTTTCTCTTTCTTTTTCATCCATTTCAGGCTCGAAAGCTTGATCTAAT
TTCCAACGGTTCGCAATTTCTGCTTTACTATCCCAGAAACCTACCGCAAGTCCTGCAAGA
TAAGCAGCACCTAACGCAGTCGTTTCATTGATTTCAGGACGTTCTACTGTAATATTTTGA
ATATCTGCTTGGAACTGCATTAAGAAGTTGTTCTTAACAGCACCACCATCTACACGGATA
TTCTTCACTTCAATACCTGAGTCTTGAATCATCGCTTCAACCACATCACGTGTTTGGTAA
CAAATAGATTCTAACGTTGCTCTGATAAAGTGTTCTTTCTCAGTCGCACGCGTAATACCA
AATACTGCACCACGCGCATTCGCATCCCAATATGGTGTACCGAGTCCAACAAACGCCGGT
ACCACATAAACACCATCACTTGATTCAATACGGTTCGCATAGTTTTCAGATTCGGGTGCT
GAGTTAATCATACGTAAACCATCTCGCAACCATTGAATAGCTGAACCAGAGACGAAAATA
GAACCTTCTAATGCATAATTCACTTCGCCATCTAAGCCGAATGCGATTGTTGTTAATAAA
CCGTTATCCGATTGAATCGCTTCTTTACCAGTGTTCATTAACATGAATCCACCCGTACCA
TATGTGTTTTTCACTTCACCACGTTCGAAGCATGCTTGTCCGAATAAGGCTGCTTGCTGA
TCCCCTGCGATTCCCGCAATCGGTACTTCTTGTCCAAAGAAATGATAATCTTTTGTTTTC
GCATAAACCTCACTTTTCTTTTGCATGGTTAATCGCTTCAATTGTTTGAGGCATAACACC
GTCGTCTGCTGCAACCACTAAGATTGTGATATCAGTGACTTGCGCACCACGTGCACGCAT
AGTAGTGAAGGCTGCGTGTCCAGGTGTATCTAAGAAAGTGATTTTTTTGCCGTCATTTGT
GATTTGGTAAGCACCGATATGTTGTGTGATACCGCCCGCTTCACCTTGTGTCACATGTGT
ATTTCTGATTGAGTCAAGTAGCGTTGTTTTACCATGGTCAACGTGTCCCATGATTGTTAC
AACTGCTGGACGCTCAATTGCATCCGGATCTTCTTCTTCATCATCGAAGTAGATATCTAA
GTCTTCTTCGTCAATGACTACTTCTTTTTCTACTTCTACACCATAATCATCAGCGATTAA
CTCAATTGTTTCATCGTCTAATGATTGGTTAATGTTTGCCATAATACCAAGTAAGAATAA
TTTCTTAACAATACTTGATGACTCAATATTCATTTTGTCAGCTAATTCGCCAACCGTAAT
ACCTTCTAAATAAGTAATTTTTTCTGGCATTTCTTTTTTCGGTTGTTCTGGTGCTGGTTT
TTGATTATTGTTTTTCTTATGTTTGTTGTTGT

Image S1: The alignment image of concatenated (combined) MLST sequencing data.

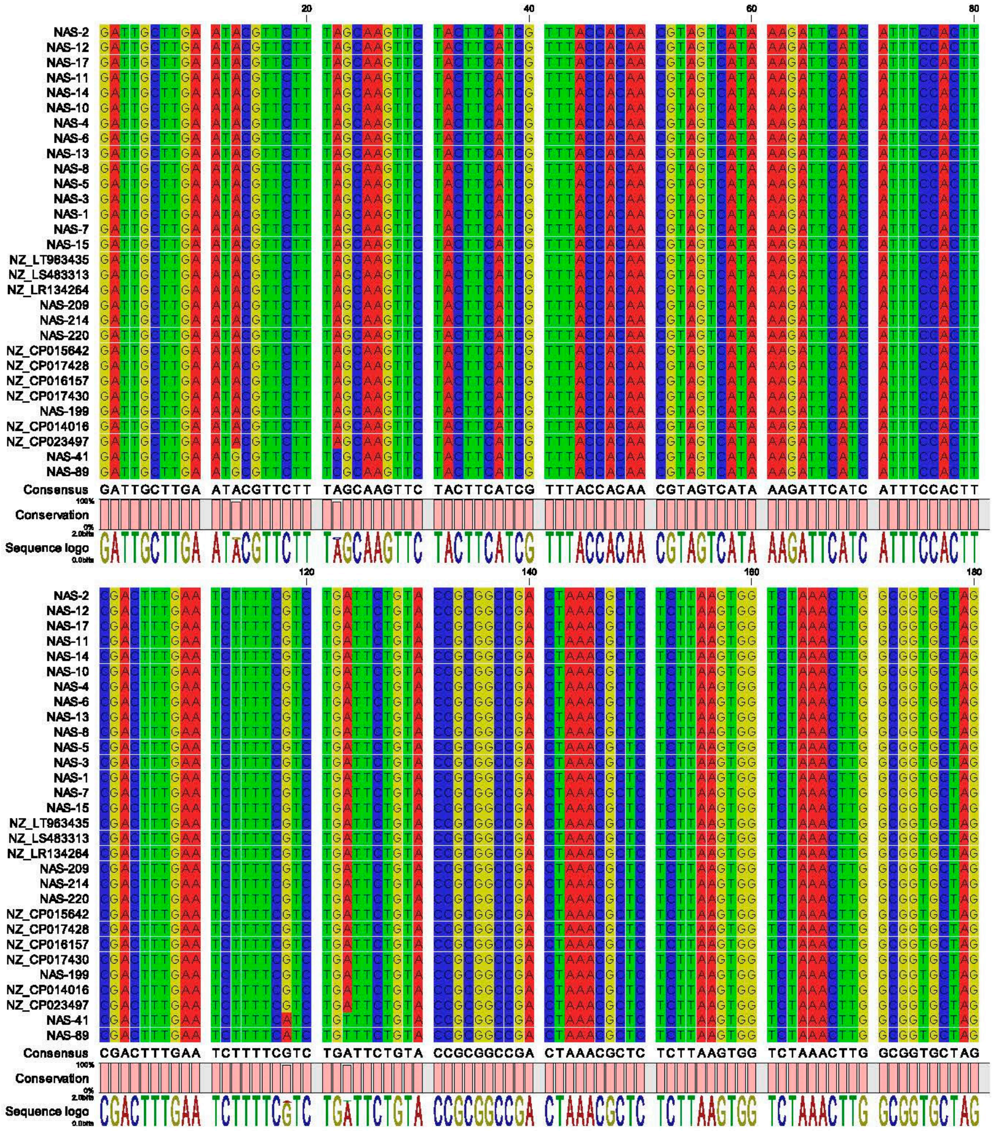

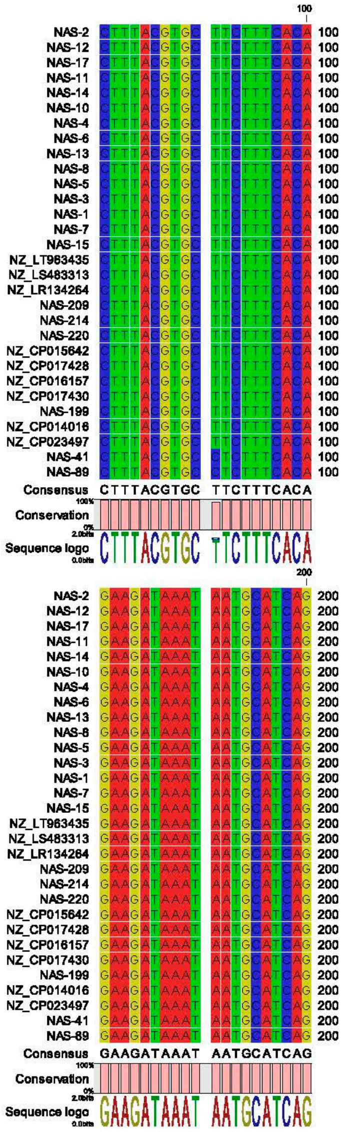

100
NAS-2
NAS-12
NAS-17
NAS-11
NAS-14
NAS-10
NAS-4
NAS-6
NAS-13
NAS-8
NAS-5
NAS-3
NAS-1
NAS-7
NAS-15
NZ_LT963435
NZ_LS483313
NZ_LR134264
NAS-209
NAS-214
NAS-220
NZ_CP015642
NZ_CP017428
NZ_CP016157
NZ_CP017430
NAS-199
NZ_CP014016
NZ_CP023497
NAS-41
NAS-89
Consensus
CTTTACGTGC TTCTTTCACA
Conservation
Sequence logo
200
Consensus
GAAGATAAAT AATGCATCAG
Conservation
Sequence logo

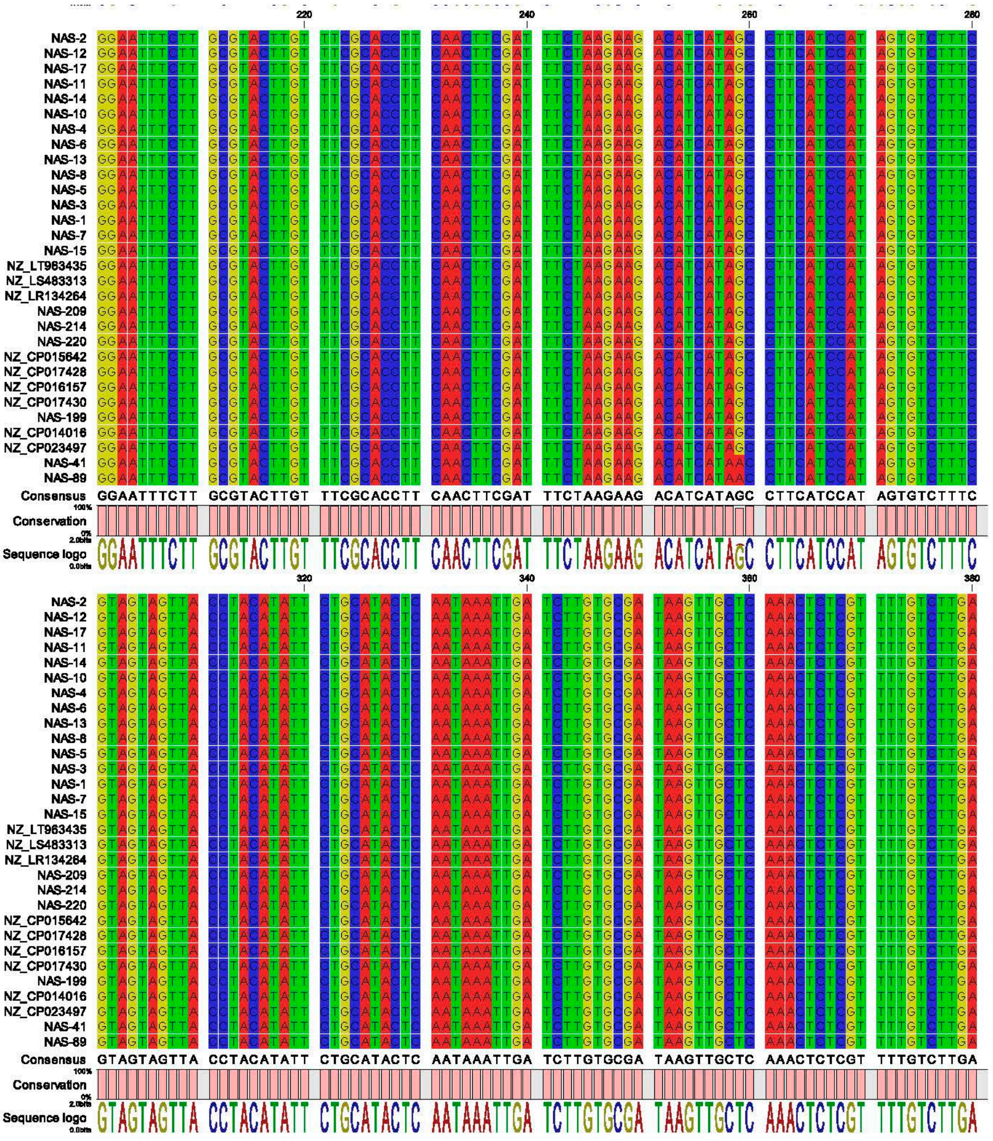

220
240
260
280
NAS-2
NAS-12
NAS-17
NAS-11
NAS-14
NAS-10
NAS-4
NAS-6
NAS-13
NAS-8
NAS-5
NAS-3
NAS-1
NAS-7
NAS-15
NZ_LT963435
NZ_LS483313
NZ_LR134264
NAS-209
NAS-214
NAS-220
NZ_CP015642
NZ_CP017428
NZ_CP016157
NZ_CP017430
NAS-199
NZ_CP014016
NZ_CP023497
NAS-41
NAS-89
Consensus GGAATTTCTT GCGTACTTGT TTCGCACCTT CAACTTCGAT TTCTAAGAAG ACATCATAGC CTTCATCCAT AGTGTCTTTC
Conservation
Sequence logo
320
340
360
380
NAS-2
NAS-12
NAS-17
NAS-11
NAS-14
NAS-10
NAS-4
NAS-6
NAS-13
NAS-8
NAS-5
NAS-3
NAS-1
NAS-7
NAS-15
NZ_LT963435
NZ_LS483313
NZ_LR134264
NAS-209
NAS-214
NAS-220
NZ_CP015642
NZ_CP017428
NZ_CP016157
NZ_CP017430
NAS-199
NZ_CP014016
NZ_CP023497
NAS-41
NAS-89
Consensus GTAGTAGTTA CCTACATATT CTGCATACTC AATAAATTGA TCTTGTGCGA TAAGTTGCTC AAACTCTCGT TTTGTCTTGA
Conservation
Sequence logo

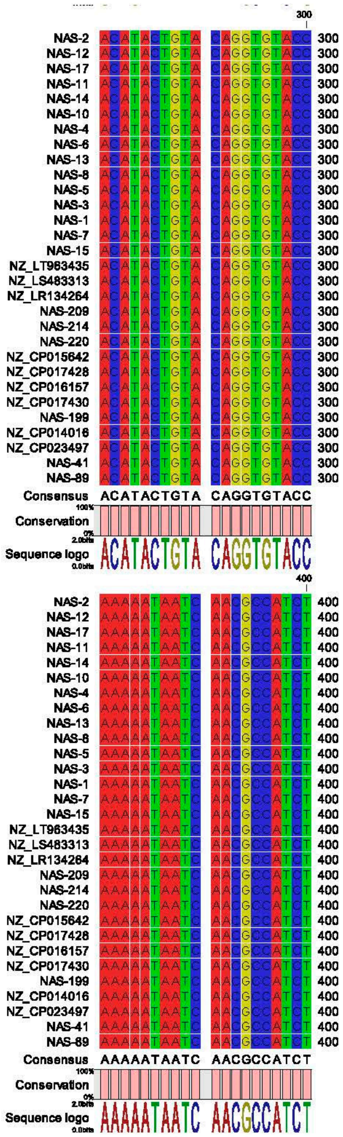
300
NAS-2
NAS-12
NAS-17
NAS-11
NAS-14
NAS-10
NAS-4
NAS-6
NAS-13
NAS-8
NAS-5
NAS-3
NAS-1
NAS-7
NAS-15
NZ_LT963435
NZ_LS483313
NZ_LR134264
NAS-209
NAS-214
NAS-220
NZ_CP015642
NZ_CP017428
NZ_CP016157
NZ_CP017430
NAS-199
NZ_CP014016
NZ_CP023497
NAS-41
NAS-89
Consensus ACATACTGTA CAGGTGTACC
Conservation
Sequence logo
400
Consensus AAAAATAATC AACGCCATCT
Conservation
Sequence logo

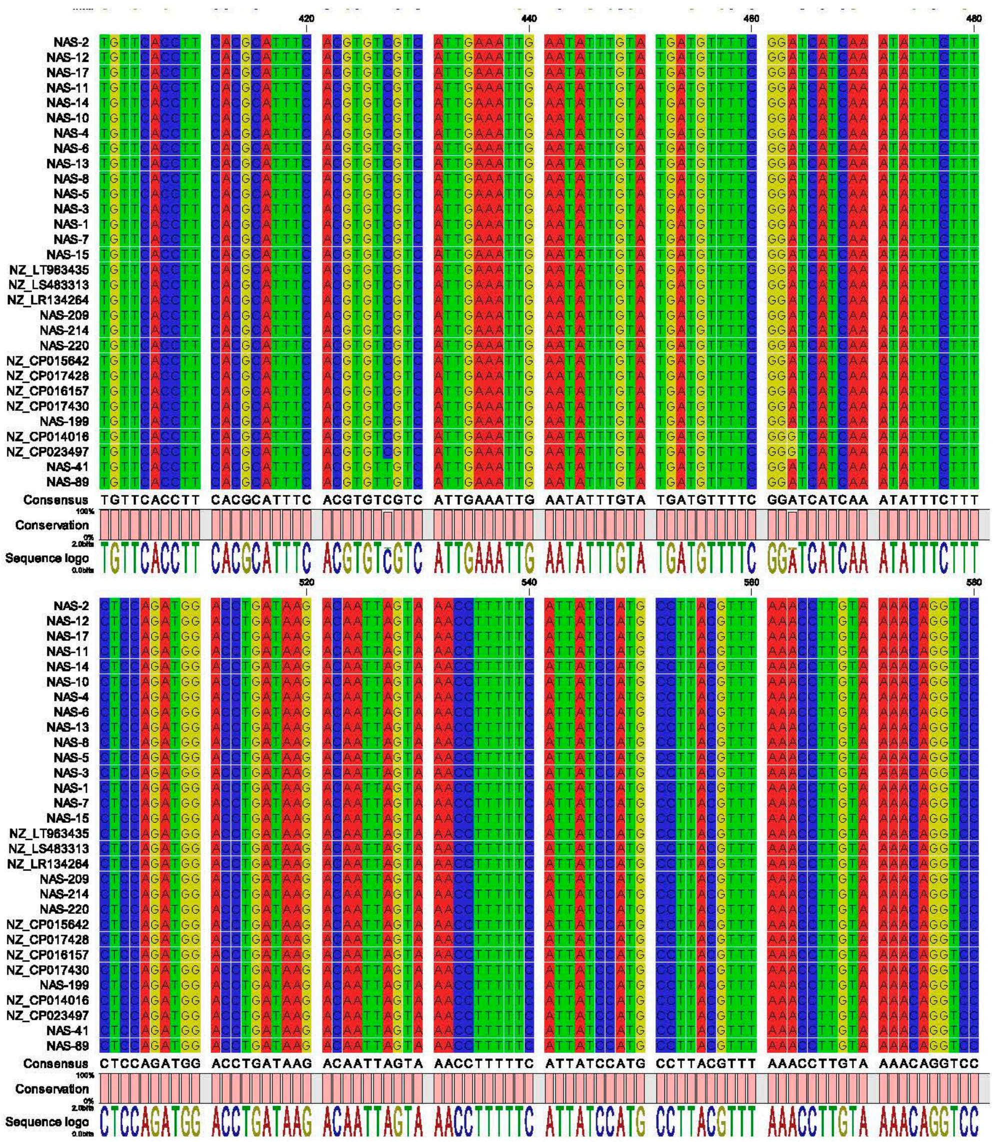
420
440
460
480
NAS-2
NAS-12
NAS-17
NAS-11
NAS-14
NAS-10
NAS-4
NAS-6
NAS-13
NAS-8
NAS-5
NAS-3
NAS-1
NAS-7
NAS-15
NZ_LT963435
NZ_LS483313
NZ_LR134264
NAS-209
NAS-214
NAS-220
NZ_CP015642
NZ_CP017428
NZ_CP016157
NZ_CP017430
NAS-199
NZ_CP014016
NZ_CP023497
NAS-41
NAS-89
Consensus TGTTCACCTT CACGCATTTC ACGTGTCGTC ATTGAAATTG AATATTTGTA TGATGTTTTC GGATCATCAA ATATTTCTTT
Conservation
Sequence logo
520
540
560
580
Consensus CTCCAGATGG ACCTGATAAG ACAATTAGTA AACCTTTTTC ATTATCCATG CCTTACGTTT AAACCTTGTA AAACAGGTCC
Conservation
Sequence logo

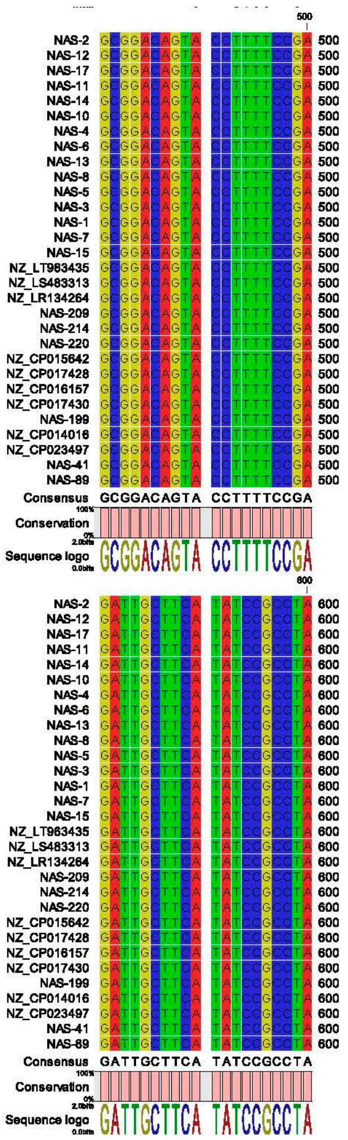
500
NAS-2
NAS-12
NAS-17
NAS-11
NAS-14
NAS-10
NAS-4
NAS-6
NAS-13
NAS-8
NAS-5
NAS-3
NAS-1
NAS-7
NAS-15
NZ_LT963435
NZ_LS483313
NZ_LR134264
NAS-209
NAS-214
NAS-220
NZ_CP015642
NZ_CP017428
NZ_CP016157
NZ_CP017430
NAS-199
NZ_CP014016
NZ_CP023497
NAS-41
NAS-89
Consensus GCGGACAGTA CCTTTTCCGA
Conservation
Sequence logo
600
Consensus GATTGCTTCA TATCCGCCTA
Conservation
Sequence logo

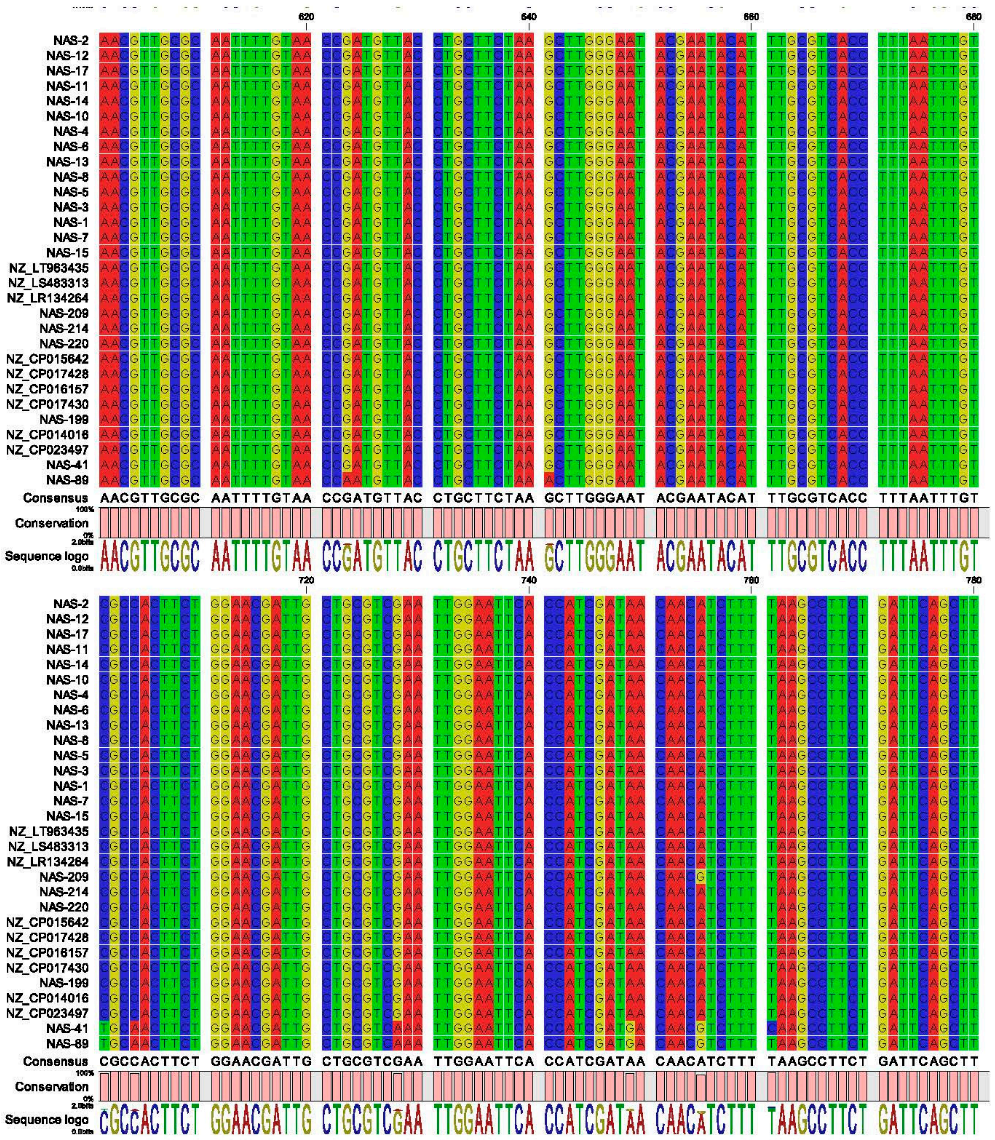
620
640
660
680
NAS-2
NAS-12
NAS-17
NAS-11
NAS-14
NAS-10
NAS-4
NAS-6
NAS-13
NAS-8
NAS-5
NAS-3
NAS-1
NAS-7
NAS-15
NZ_LT963435
NZ_LS483313
NZ_LR134264
NAS-209
NAS-214
NAS-220
NZ_CP015642
NZ_CP017428
NZ_CP016157
NZ_CP017430
NAS-199
NZ_CP014016
NZ_CP023497
NAS-41
NAS-89
Consensus AACGTTGCGC AATTTTGTAA CCGATGTTAC CTGCTTCTAA GCTTGGGAAT ACGAATACAT TTGCGTCACC TTTAATTTGT
Conservation
Sequence logo
720
740
760
780
Consensus CGCCACTTCT GGAACGATTG CTGCGTCGAA TTGGAATTCA CCATCGATAA CAACATCTTT TAAGCCTTCT GATTCAGCTT
Conservation
Sequence logo

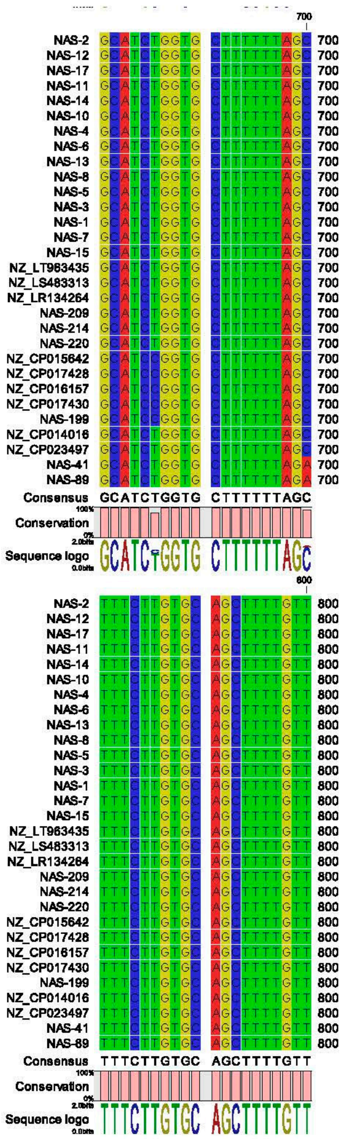
700
NAS-2 700
NAS-12 700
NAS-17 700
NAS-11 700
NAS-14 700
NAS-10 700
NAS-4 700
NAS-6 700
NAS-13 700
NAS-8 700
NAS-5 700
NAS-3 700
NAS-1 700
NAS-7 700
NAS-15 700
NZ_LT963435 700
NZ_LS483313 700
NZ_LR134264 700
NAS-209 700
NAS-214 700
NAS-220 700
NZ_CP015642 700
NZ_CP017428 700
NZ_CP016157 700
NZ_CP017430 700
NAS-199 700
NZ_CP014016 700
NZ_CP023497 700
NAS-41 700
NAS-89 700
Consensus GCATCTGGTG CTTTTTTAGC
Conservation
Sequence logo
800
NAS-2 800
NAS-12 800
NAS-17 800
NAS-11 800
NAS-14 800
NAS-10 800
NAS-4 800
NAS-6 800
NAS-13 800
NAS-8 800
NAS-5 800
NAS-3 800
NAS-1 800
NAS-7 800
NAS-15 800
NZ_LT963435 800
NZ_LS483313 800
NZ_LR134264 800
NAS-209 800
NAS-214 800
NAS-220 800
NZ_CP015642 800
NZ_CP017428 800
NZ_CP016157 800
NZ_CP017430 800
NAS-199 800
NZ_CP014016 800
NZ_CP023497 800
NAS-41 800
NAS-89 800
Consensus TTTCTTGTGC AGCTTTTGTT
Conservation
Sequence logo

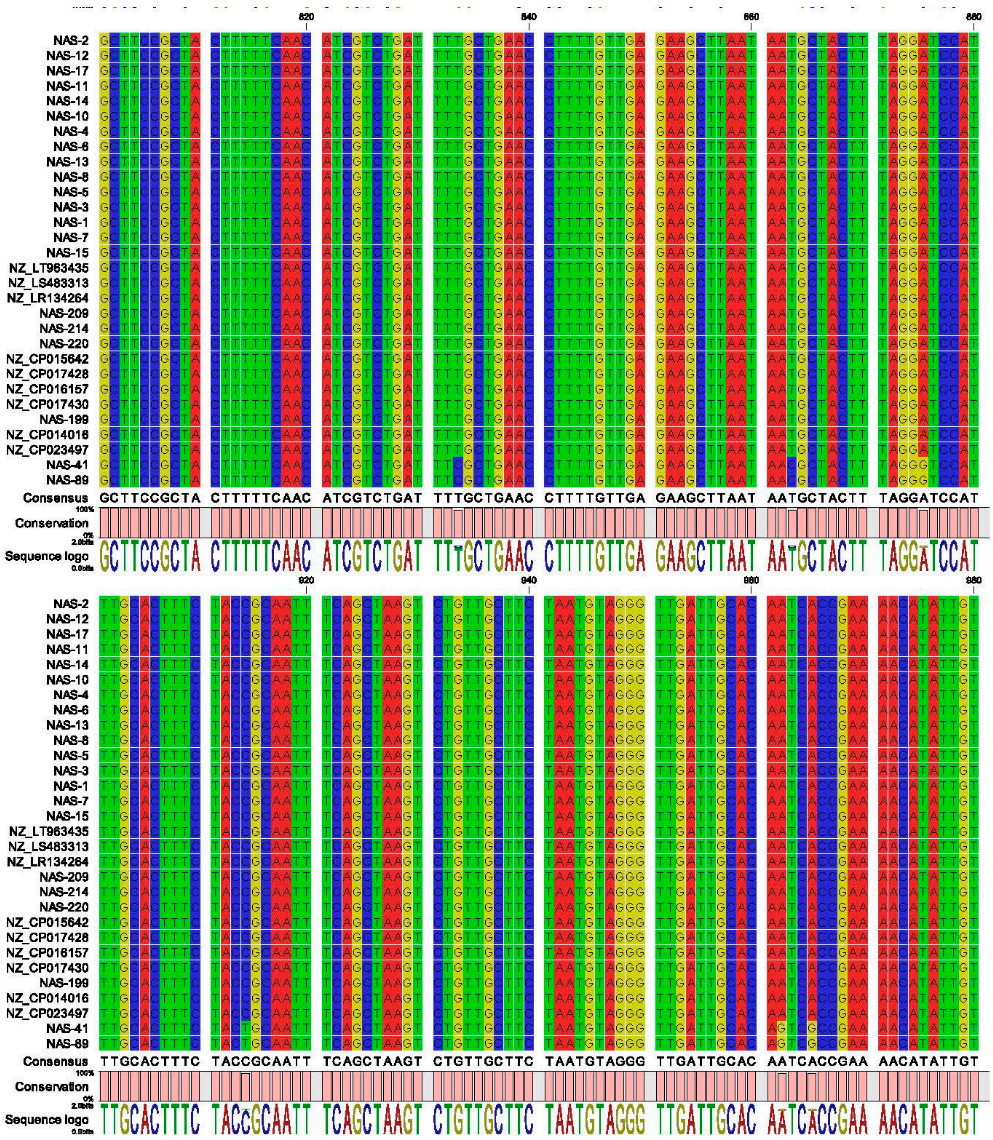
820
840
860
880
NAS-2
NAS-12
NAS-17
NAS-11
NAS-14
NAS-10
NAS-4
NAS-6
NAS-13
NAS-8
NAS-5
NAS-3
NAS-1
NAS-7
NAS-15
NZ_LT963435
NZ_LS483313
NZ_LR134264
NAS-209
NAS-214
NAS-220
NZ_CP015642
NZ_CP017428
NZ_CP016157
NZ_CP017430
NAS-199
NZ_CP014016
NZ_CP023497
NAS-41
NAS-89
Consensus GCTTCCGCTA CTTTTTCAAC ATCGTCTGAT TTTGCTGAAC CTTTTGTTGA GAAGCTTAAT AATGCTACTT TAGGATCCAT
Conservation
Sequence logo
920
940
960
980
NAS-2
NAS-12
NAS-17
NAS-11
NAS-14
NAS-10
NAS-4
NAS-6
NAS-13
NAS-8
NAS-5
NAS-3
NAS-1
NAS-7
NAS-15
NZ_LT963435
NZ_LS483313
NZ_LR134264
NAS-209
NAS-214
NAS-220
NZ_CP015642
NZ_CP017428
NZ_CP016157
NZ_CP017430
NAS-199
NZ_CP014016
NZ_CP023497
NAS-41
NAS-89
Consensus TTGCACTTTC TACCGCAATT TCAGCTAAGT CTGTTGCTTC TAATGTAGGG TTGATTGCAC AATCACCGAA AACATATTGT
Conservation
Sequence logo

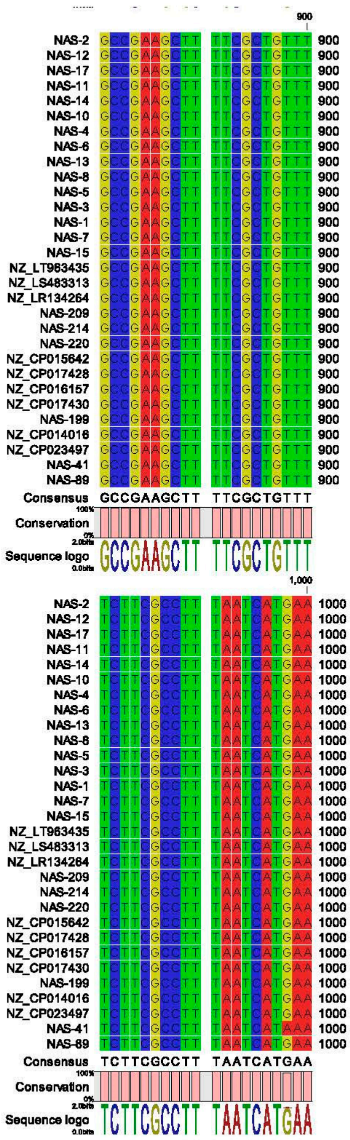
900
NAS-2
NAS-12
NAS-17
NAS-11
NAS-14
NAS-10
NAS-4
NAS-6
NAS-13
NAS-8
NAS-5
NAS-3
NAS-1
NAS-7
NAS-15
NZ_LT963435
NZ_LS483313
NZ_LR134264
NAS-209
NAS-214
NAS-220
NZ_CP015642
NZ_CP017428
NZ_CP016157
NZ_CP017430
NAS-199
NZ_CP014016
NZ_CP023497
NAS-41
NAS-89
Consensus GCCGAAGCTT TTCGCTGTTT
Conservation
Sequence logo
1,000
Consensus TCTTCGCCTT TAATCATGAA
Conservation
Sequence logo

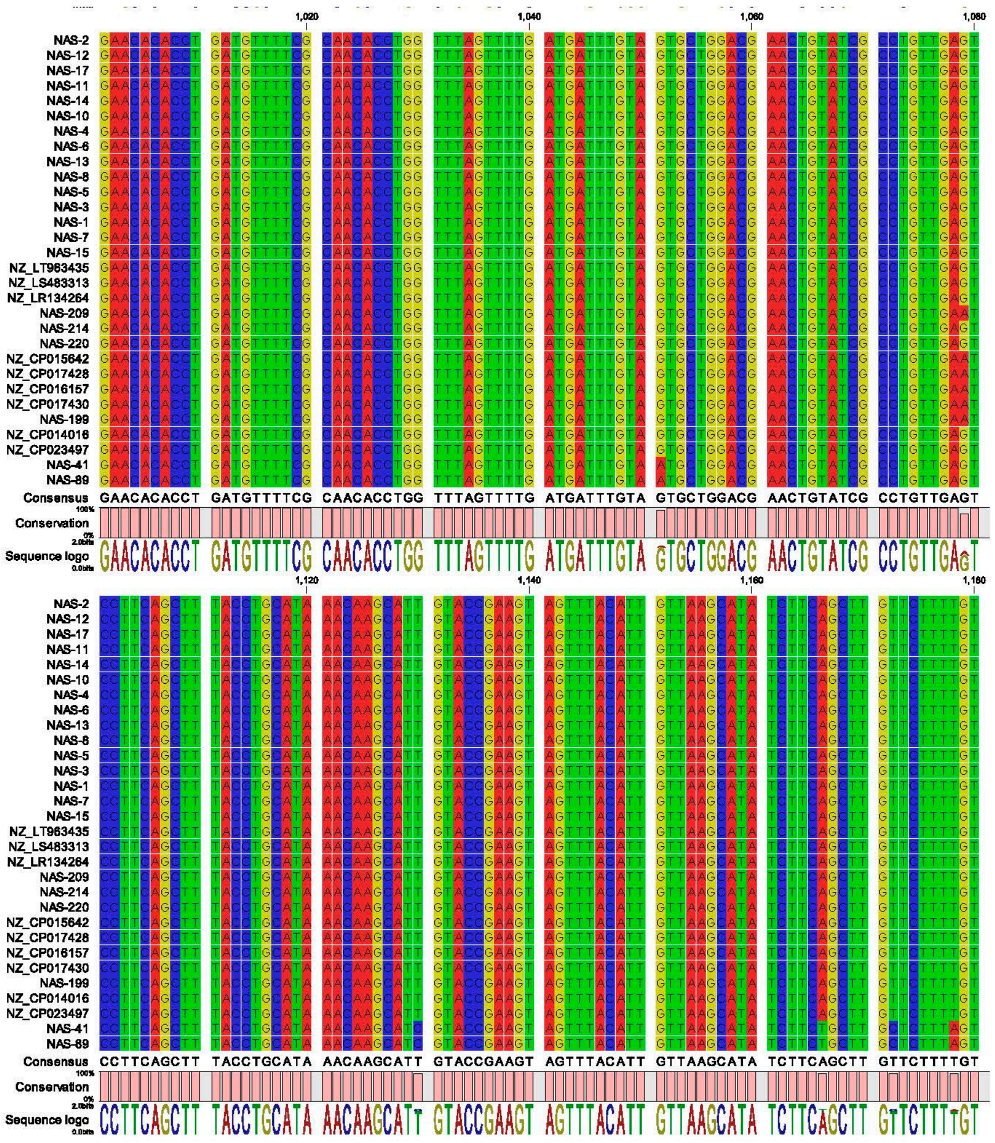
1,020
1,040
1,060
1,080
NAS-2
NAS-12
NAS-17
NAS-11
NAS-14
NAS-10
NAS-4
NAS-6
NAS-13
NAS-8
NAS-5
NAS-3
NAS-1
NAS-7
NAS-15
NZ_LT963435
NZ_LS483313
NZ_LR134264
NAS-209
NAS-214
NAS-220
NZ_CP015642
NZ_CP017428
NZ_CP016157
NZ_CP017430
NAS-199
NZ_CP014016
NZ_CP023497
NAS-41
NAS-89
Consensus GAACACACCT GATGTTTTCG CAACACCTGG TTTAGTTTTG ATGATTTGTA GTGCTGGACG AACTGTATCG CCTGTTGAGT
Conservation
Sequence logo
1,120
1,140
1,160
1,180
Consensus CCTTCAGCTT TACCTGCATA AACAAGCATT GTACCGAAGT AGTTTACATT GTTAAGCATA TCTTCAGCTT GTTCTTTTGT
Conservation
Sequence logo

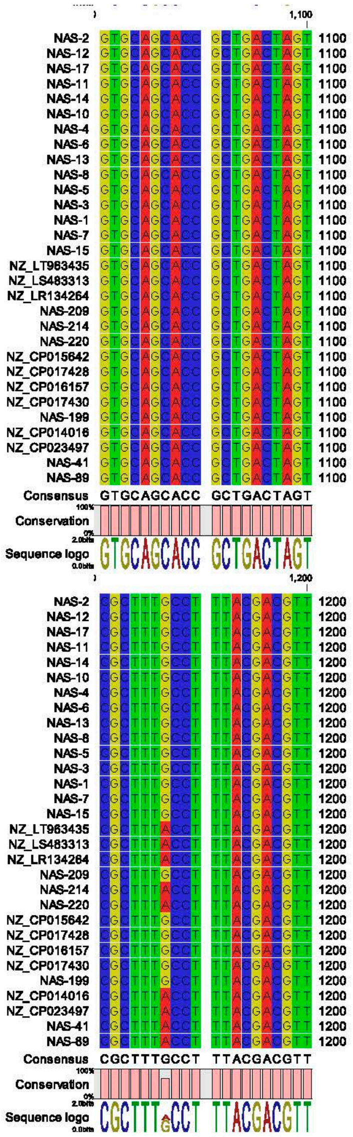
NAS-2
NAS-12
NAS-17
NAS-11
NAS-14
NAS-10
NAS-4
NAS-6
NAS-13
NAS-8
NAS-5
NAS-3
NAS-1
NAS-7
NAS-15
NZ_LT963435
NZ_LS483313
NZ_LR134264
NAS-209
NAS-214
NAS-220
NZ_CP015642
NZ_CP017428
NZ_CP016157
NZ_CP017430
NAS-199
NZ_CP014016
NZ_CP023497
NAS-41
NAS-89
Consensus GTGCAGCACC GCTGACTAGT
Conservation
Sequence logo
Consensus CGCTTTGCCT TTACGACGTT
1,100
1,200
1100
1200

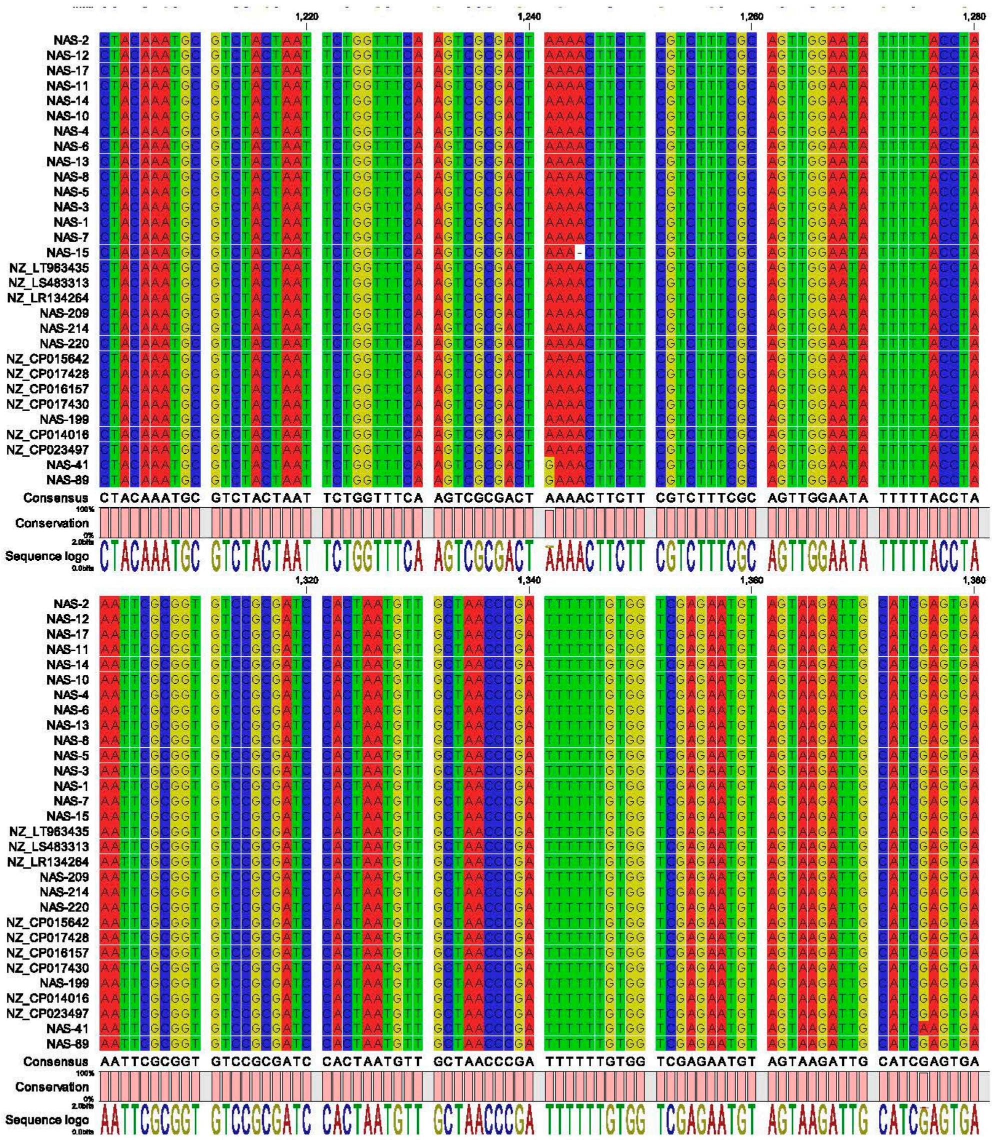
1,220
1,240
1,260
1,280
NAS-2
NAS-12
NAS-17
NAS-11
NAS-14
NAS-10
NAS-4
NAS-6
NAS-13
NAS-8
NAS-5
NAS-3
NAS-1
NAS-7
NAS-15
NZ_LT963435
NZ_LS483313
NZ_LR134264
NAS-209
NAS-214
NAS-220
NZ_CP015642
NZ_CP017428
NZ_CP016157
NZ_CP017430
NAS-199
NZ_CP014016
NZ_CP023497
NAS-41
NAS-89
Consensus CTACAAATGC GTCTACTAAT TCTGGTTTCA AGTCGCGACT AAAACTTCTT CGTCTTTCGC AGTTGGAATA TTTTTACCTA
Conservation
Sequence logo
1,320
1,340
1,360
1,380
NAS-2
NAS-12
NAS-17
NAS-11
NAS-14
NAS-10
NAS-4
NAS-6
NAS-13
NAS-8
NAS-5
NAS-3
NAS-1
NAS-7
NAS-15
NZ_LT963435
NZ_LS483313
NZ_LR134264
NAS-209
NAS-214
NAS-220
NZ_CP015642
NZ_CP017428
NZ_CP016157
NZ_CP017430
NAS-199
NZ_CP014016
NZ_CP023497
NAS-41
NAS-89
Consensus AATTCGCGGT GTCCGCGATC CACTAATGTT GCTAACCCGA TTTTTTGTGG TCGAGAATGT AGTAAGATTG CATCGAGTGA
Conservation
Sequence logo

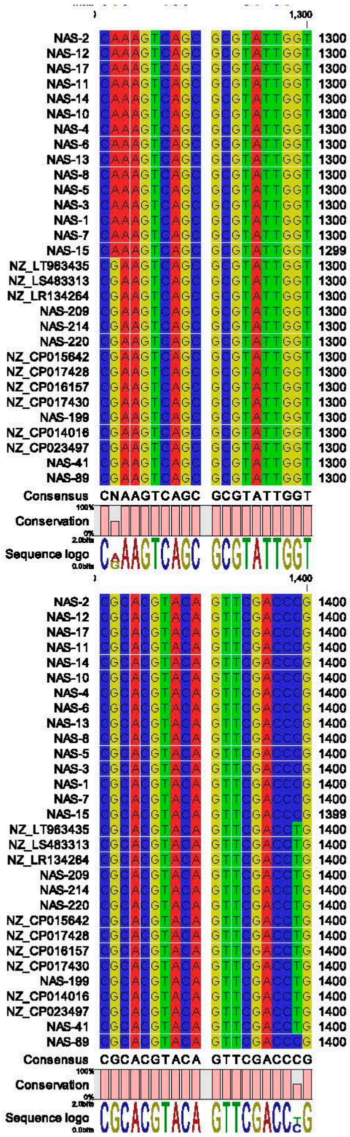
1,300
NAS-2 1300
NAS-12 1300
NAS-17 1300
NAS-11 1300
NAS-14 1300
NAS-10 1300
NAS-4 1300
NAS-6 1300
NAS-13 1300
NAS-8 1300
NAS-5 1300
NAS-3 1300
NAS-1 1300
NAS-7 1300
NAS-15 1299
NZ_LT963435 1300
NZ_LS483313 1300
NZ_LR134264 1300
NAS-209 1300
NAS-214 1300
NAS-220 1300
NZ_CP015642 1300
NZ_CP017428 1300
NZ_CP016157 1300
NZ_CP017430 1300
NAS-199 1300
NZ_CP014016 1300
NZ_CP023497 1300
NAS-41 1300
NAS-89 1300
Consensus CNAAGTCAGC GCGTATTGGT
Conservation
Sequence logo
1,400
NAS-2 1400
NAS-12 1400
NAS-17 1400
NAS-11 1400
NAS-14 1400
NAS-10 1400
NAS-4 1400
NAS-6 1400
NAS-13 1400
NAS-8 1400
NAS-5 1400
NAS-3 1400
NAS-1 1400
NAS-7 1400
NAS-15 1399
NZ_LT963435 1400
NZ_LS483313 1400
NZ_LR134264 1400
NAS-209 1400
NAS-214 1400
NAS-220 1400
NZ_CP015642 1400
NZ_CP017428 1400
NZ_CP016157 1400
NZ_CP017430 1400
NAS-199 1400
NZ_CP014016 1400
NZ_CP023497 1400
NAS-41 1400
NAS-89 1400
Consensus CGCACGTACA GTTCGACCCG
Conservation
Sequence logo

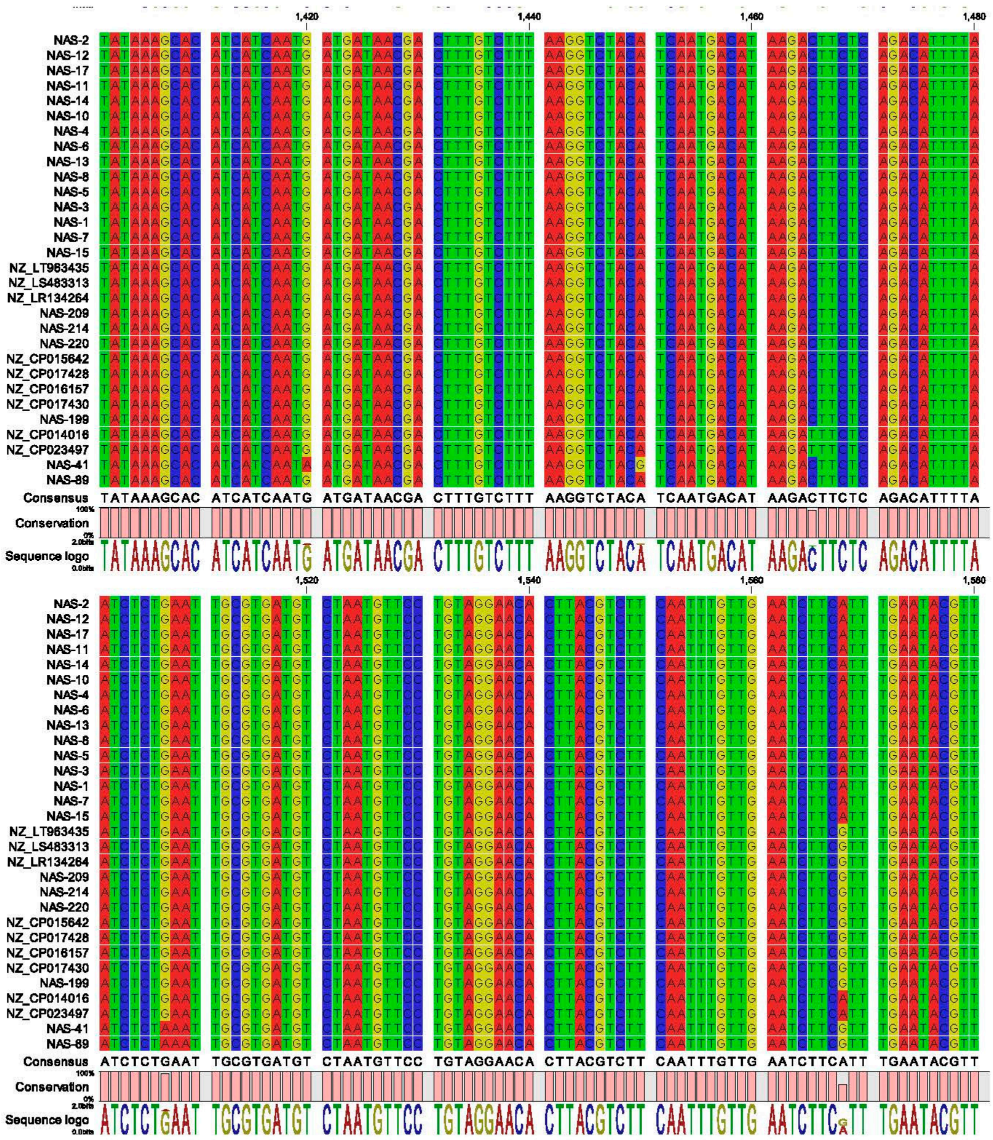
1,420
1,440
1,460
1,480
Consensus
TATAAAGCAC ATCATCAATG ATGATAACGA CTTTGTCTTT AAGGTCTACA TCAATGACAT AAGACTTCTC AGACATTTTA
Conservation
Sequence logo
1,520
1,540
1,560
1,580
ATCTCTGAAT TGCGTGATGT CTAATGTTCC TGTAGGAACA CTTACGTCTT CAATTTGTTG AATCTTCATT TGAATACGTT

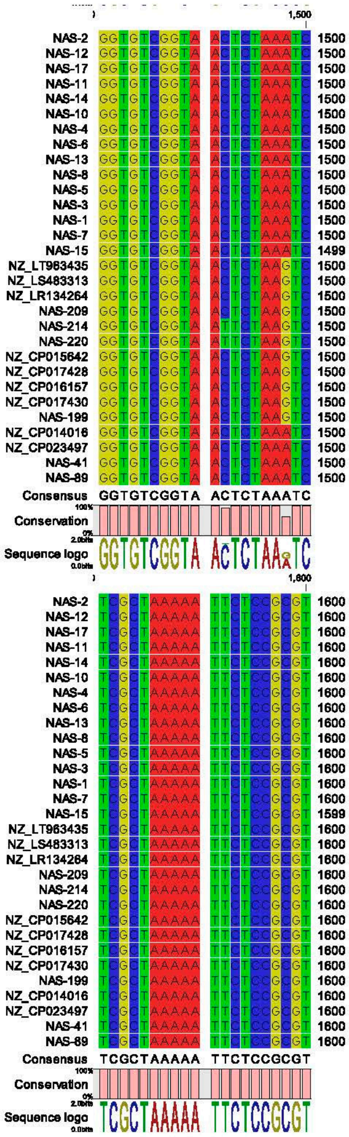
1,500
NAS-2 1500
NAS-12 1500
NAS-17 1500
NAS-11 1500
NAS-14 1500
NAS-10 1500
NAS-4 1500
NAS-6 1500
NAS-13 1500
NAS-8 1500
NAS-5 1500
NAS-3 1500
NAS-1 1500
NAS-7 1500
NAS-15 1499
NZ_LT963435 1500
NZ_LS483313 1500
NZ_LR134264 1500
NAS-209 1500
NAS-214 1500
NAS-220 1500
NZ_CP015642 1500
NZ_CP017428 1500
NZ_CP016157 1500
NZ_CP017430 1500
NAS-199 1500
NZ_CP014016 1500
NZ_CP023497 1500
NAS-41 1500
NAS-89 1500
Consensus GGTGTCGGTA ACTCTAAATC
Conservation
Sequence logo
1,600
NAS-2 1600
NAS-12 1600
NAS-17 1600
NAS-11 1600
NAS-14 1600
NAS-10 1600
NAS-4 1600
NAS-6 1600
NAS-13 1600
NAS-8 1600
NAS-5 1600
NAS-3 1600
NAS-1 1600
NAS-7 1600
NAS-15 1599
NZ_LT963435 1600
NZ_LS483313 1600
NZ_LR134264 1600
NAS-209 1600
NAS-214 1600
NAS-220 1600
NZ_CP015642 1600
NZ_CP017428 1600
NZ_CP016157 1600
NZ_CP017430 1600
NAS-199 1600
NZ_CP014016 1600
NZ_CP023497 1600
NAS-41 1600
NAS-89 1600
Consensus TCGCTAAAAA TTCTCCGCGT
Conservation
Sequence logo

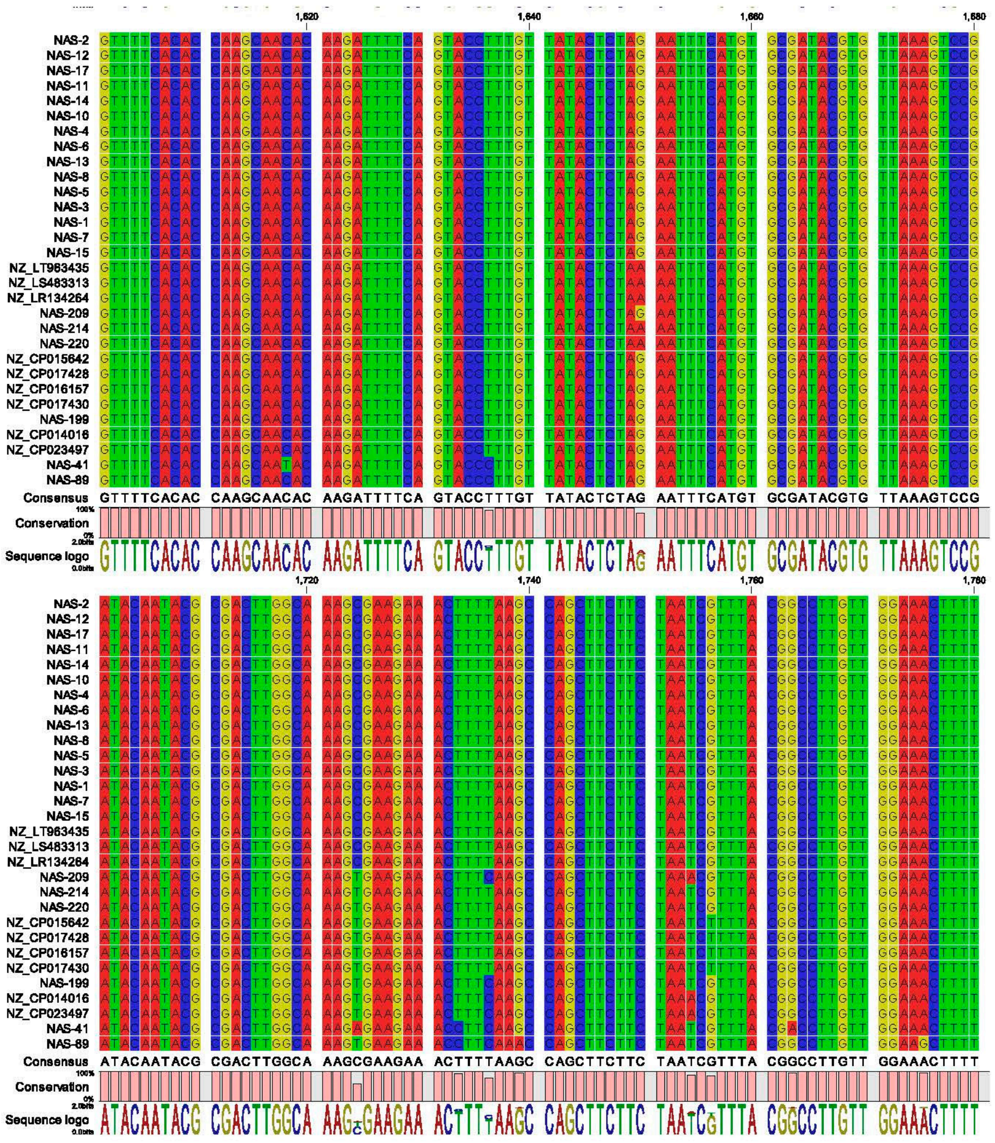
1,620
1,640
1,660
1,680
NAS-2
NAS-12
NAS-17
NAS-11
NAS-14
NAS-10
NAS-4
NAS-6
NAS-13
NAS-8
NAS-5
NAS-3
NAS-1
NAS-7
NAS-15
NZ_LT963435
NZ_LS483313
NZ_LR134264
NAS-209
NAS-214
NAS-220
NZ_CP015642
NZ_CP017428
NZ_CP016157
NZ_CP017430
NAS-199
NZ_CP014016
NZ_CP023497
NAS-41
NAS-89
Consensus GTTTTCACAC CAAGCAACAC AAGATTTTCA GTACCTTTGT TATACTCTAG AATTTCATGT GCGATACGTG TTAAAGTCCG
Conservation
Sequence logo
1,720
1,740
1,760
1,780
NAS-2
NAS-12
NAS-17
NAS-11
NAS-14
NAS-10
NAS-4
NAS-6
NAS-13
NAS-8
NAS-5
NAS-3
NAS-1
NAS-7
NAS-15
NZ_LT963435
NZ_LS483313
NZ_LR134264
NAS-209
NAS-214
NAS-220
NZ_CP015642
NZ_CP017428
NZ_CP016157
NZ_CP017430
NAS-199
NZ_CP014016
NZ_CP023497
NAS-41
NAS-89
Consensus ATACAATACG CGACTTGGCA AAGCGAAGAA ACTTTTAAGC CAGCTTCTTC TAATCGTTTA CGGCCTTGTT GGAAACTTTT
Conservation
Sequence logo

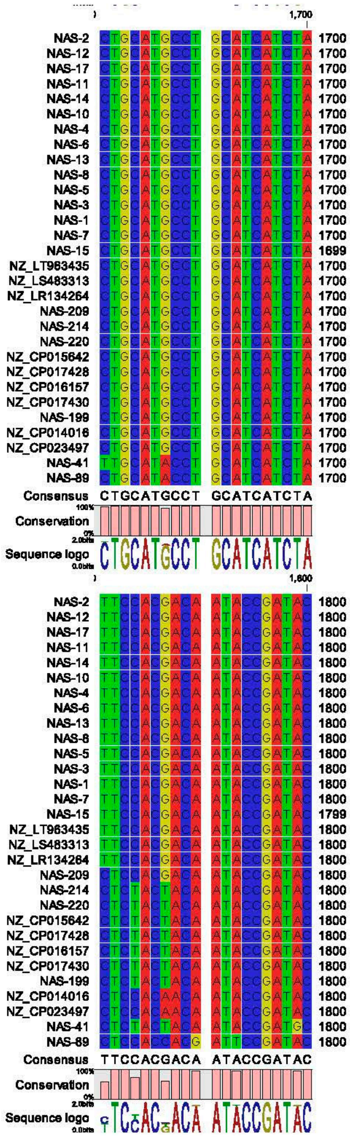

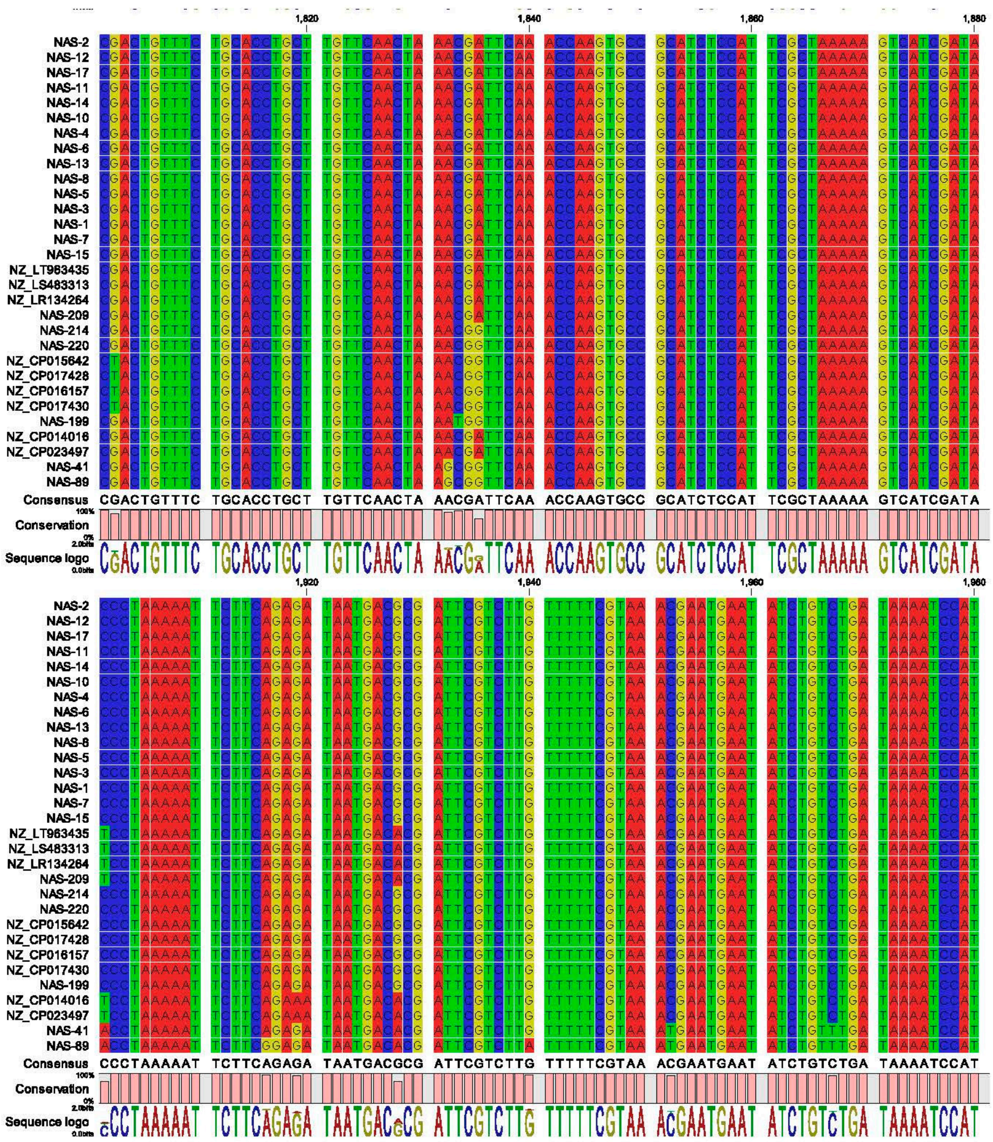
1,820
1,840
1,860
1,880
NAS-2
NAS-12
NAS-17
NAS-11
NAS-14
NAS-10
NAS-4
NAS-6
NAS-13
NAS-8
NAS-5
NAS-3
NAS-1
NAS-7
NAS-15
NZ_LT963435
NZ_LS483313
NZ_LR134264
NAS-209
NAS-214
NAS-220
NZ_CP015642
NZ_CP017428
NZ_CP016157
NZ_CP017430
NAS-199
NZ_CP014016
NZ_CP023497
NAS-41
NAS-89
Consensus CGACTGTTTC TGCACCTGCT TGTTCAACTA AACGATTCAA ACCAAGTGCC GCATCTCCAT TCGCTAAAAA GTCATCGATA
Conservation
Sequence logo
1,920
1,940
1,960
1,980
Consensus CCCTAAAAAT TCTTCAGAGA TAATGACGCG ATTCGTCTTG TTTTTCGTAA ACGAATGAAT ATCTGTCTGA TAAAATCCAT
Conservation
Sequence logo

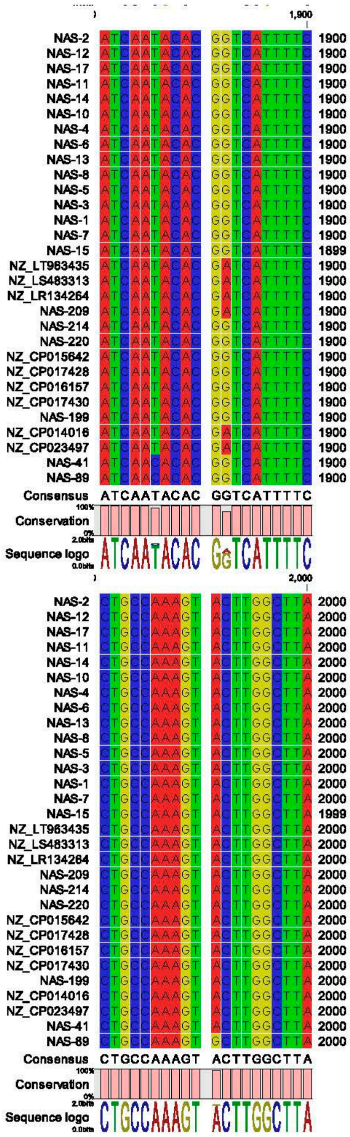

1,900
NAS-2 1900
NAS-12 1900
NAS-17 1900
NAS-11 1900
NAS-14 1900
NAS-10 1900
NAS-4 1900
NAS-6 1900
NAS-13 1900
NAS-8 1900
NAS-5 1900
NAS-3 1900
NAS-1 1900
NAS-7 1900
NAS-15 1899
NZ_LT963435 1900
NZ_LS483313 1900
NZ_LR134264 1900
NAS-209 1900
NAS-214 1900
NAS-220 1900
NZ_CP015642 1900
NZ_CP017428 1900
NZ_CP016157 1900
NZ_CP017430 1900
NAS-199 1900
NZ_CP014016 1900
NZ_CP023497 1900
NAS-41 1900
NAS-89 1900
Consensus ATCAATACAC GGTCATTTTC
Conservation
Sequence logo
2,000
NAS-2 2000
NAS-12 2000
NAS-17 2000
NAS-11 2000
NAS-14 2000
NAS-10 2000
NAS-4 2000
NAS-6 2000
NAS-13 2000
NAS-8 2000
NAS-5 2000
NAS-3 2000
NAS-1 2000
NAS-7 2000
NAS-15 1999
NZ_LT963435 2000
NZ_LS483313 2000
NZ_LR134264 2000
NAS-209 2000
NAS-214 2000
NAS-220 2000
NZ_CP015642 2000
NZ_CP017428 2000
NZ_CP016157 2000
NZ_CP017430 2000
NAS-199 2000
NZ_CP014016 2000
NZ_CP023497 2000
NAS-41 2000
NAS-89 2000
Consensus CTGCCAAAGT ACTTGGCTTA
Conservation
Sequence logo

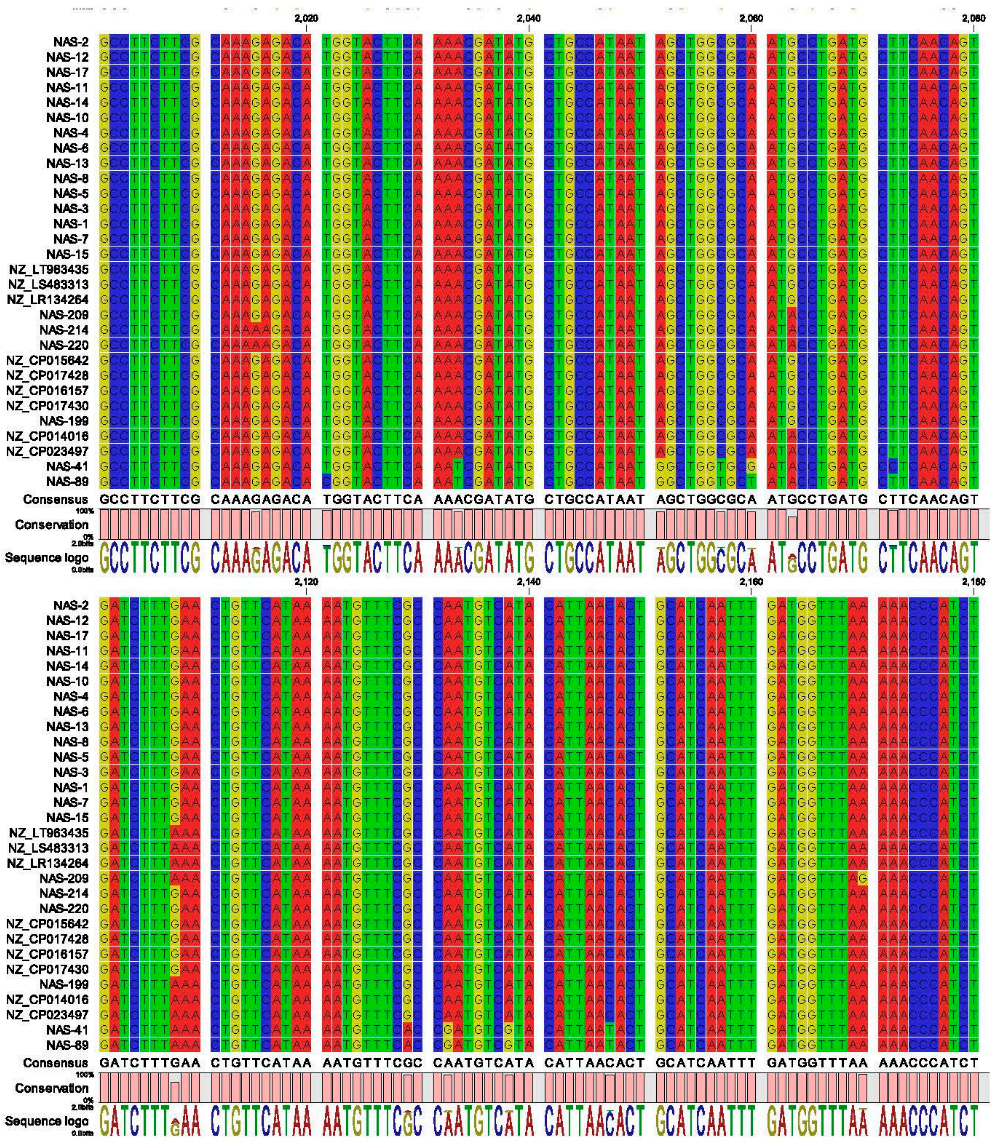
2,020
2,040
2,060
2,080
NAS-2
NAS-12
NAS-17
NAS-11
NAS-14
NAS-10
NAS-4
NAS-6
NAS-13
NAS-8
NAS-5
NAS-3
NAS-1
NAS-7
NAS-15
NZ_LT963435
NZ_LS483313
NZ_LR134264
NAS-209
NAS-214
NAS-220
NZ_CP015642
NZ_CP017428
NZ_CP016157
NZ_CP017430
NAS-199
NZ_CP014016
NZ_CP023497
NAS-41
NAS-89
Consensus GCCTTCTTCG CAAAGAGACA TGGTACTTCA AAACGATATG CTGCCATAAT AGCTGGCGCA ATGCCTGATG CTTCAACAGT
Conservation
Sequence logo
2,120
2,140
2,160
2,180
NAS-2
NAS-12
NAS-17
NAS-11
NAS-14
NAS-10
NAS-4
NAS-6
NAS-13
NAS-8
NAS-5
NAS-3
NAS-1
NAS-7
NAS-15
NZ_LT963435
NZ_LS483313
NZ_LR134264
NAS-209
NAS-214
NAS-220
NZ_CP015642
NZ_CP017428
NZ_CP016157
NZ_CP017430
NAS-199
NZ_CP014016
NZ_CP023497
NAS-41
NAS-89
Consensus GATCTTTGAA CTGTTCATAA AATGTTTCGC CAATGTCATA CATTAACACT GCATCAATTT GATGGTTTAA AAACCCATCT
Conservation
Sequence logo

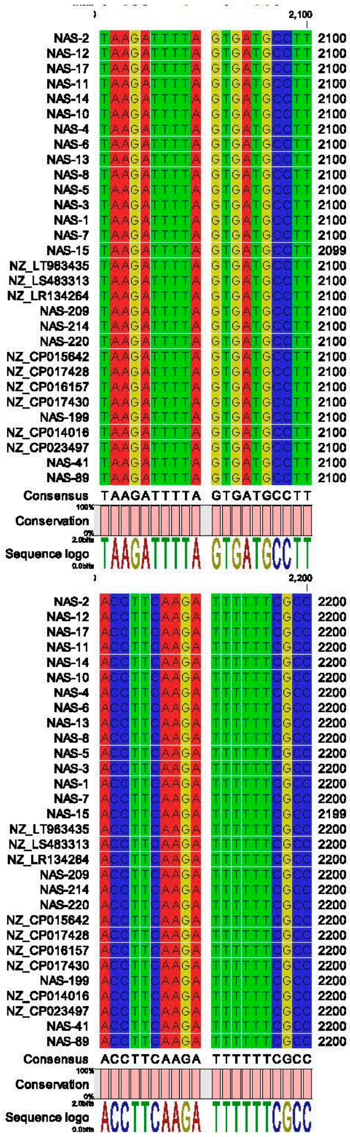
2,100
NAS-2 2100
NAS-12 2100
NAS-17 2100
NAS-11 2100
NAS-14 2100
NAS-10 2100
NAS-4 2100
NAS-6 2100
NAS-13 2100
NAS-8 2100
NAS-5 2100
NAS-3 2100
NAS-1 2100
NAS-7 2100
NAS-15 2099
NZ_LT963435 2100
NZ_LS483313 2100
NZ_LR134264 2100
NAS-209 2100
NAS-214 2100
NAS-220 2100
NZ_CP015642 2100
NZ_CP017428 2100
NZ_CP016157 2100
NZ_CP017430 2100
NAS-199 2100
NZ_CP014016 2100
NZ_CP023497 2100
NAS-41 2100
NAS-89 2100
Consensus TAAGATTTTA GTGATGCCTT
Conservation
Sequence logo TAAGATTTTA GTGATGCCTT
2,200
NAS-2 2200
NAS-12 2200
NAS-17 2200
NAS-11 2200
NAS-14 2200
NAS-10 2200
NAS-4 2200
NAS-6 2200
NAS-13 2200
NAS-8 2200
NAS-5 2200
NAS-3 2200
NAS-1 2200
NAS-7 2200
NAS-15 2199
NZ_LT963435 2200
NZ_LS483313 2200
NZ_LR134264 2200
NAS-209 2200
NAS-214 2200
NAS-220 2200
NZ_CP015642 2200
NZ_CP017428 2200
NZ_CP016157 2200
NZ_CP017430 2200
NAS-199 2200
NZ_CP014016 2200
NZ_CP023497 2200
NAS-41 2200
NAS-89 2200
Consensus ACCTTCAAGA TTTTTTCGCC
Conservation
Sequence logo ACCTTCAAGA TTTTTTCGCC

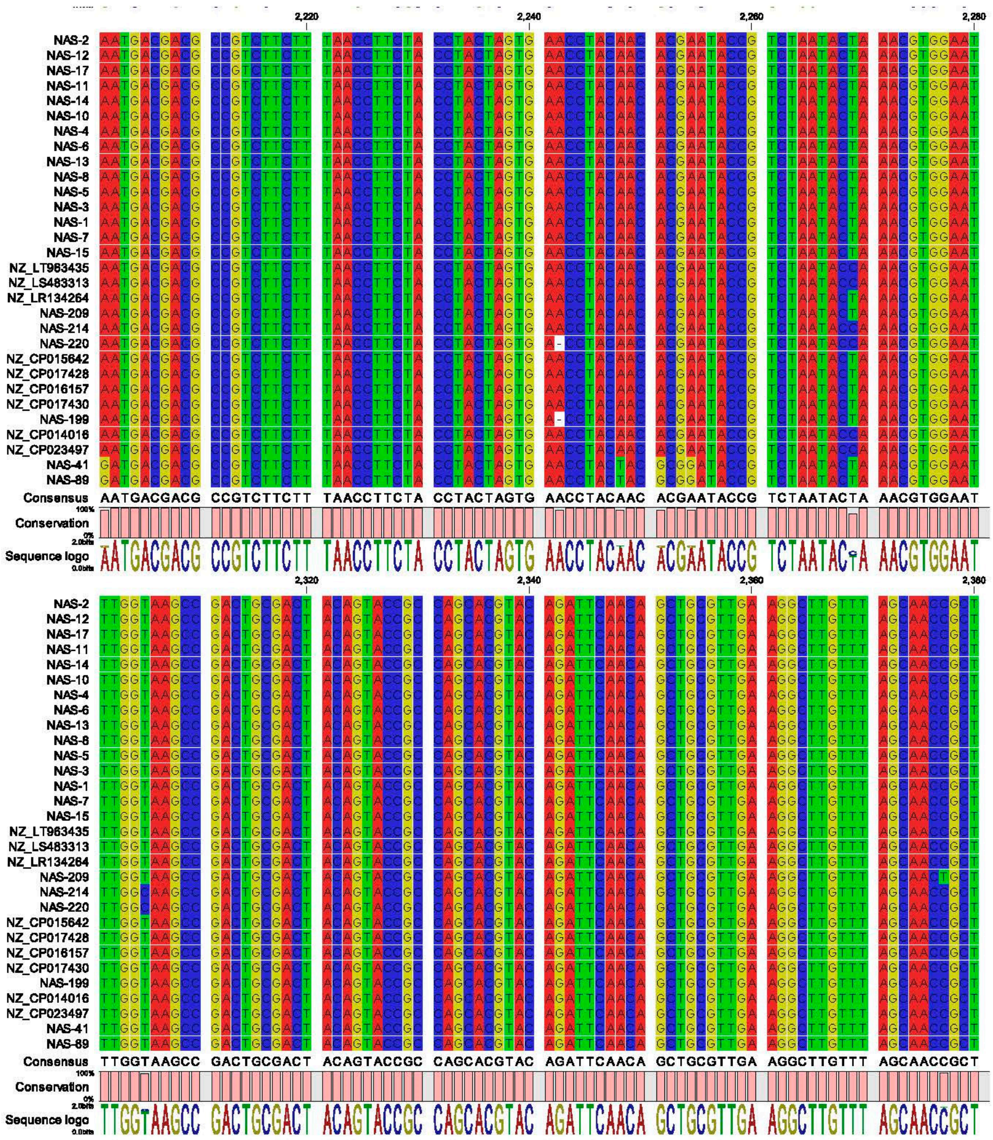
2,220
2,240
2,260
2,280
NAS-2
NAS-12
NAS-17
NAS-11
NAS-14
NAS-10
NAS-4
NAS-6
NAS-13
NAS-8
NAS-5
NAS-3
NAS-1
NAS-7
NAS-15
NZ_LT963435
NZ_LS483313
NZ_LR134264
NAS-209
NAS-214
NAS-220
NZ_CP015642
NZ_CP017428
NZ_CP016157
NZ_CP017430
NAS-199
NZ_CP014016
NZ_CP023497
NAS-41
NAS-89
Consensus
AATGACGACG CCGTCTTCTT TAACCTTCTA CCTACTAGTG AACCTACAAC ACGAATACCG TCTAATACTA AACGTGGAAT
Conservation
Sequence logo
2,320
2,340
2,360
2,380
Consensus
TTGGTAAGCC GACTGCGACT ACAGTACCGC CAGCACGTAC AGATTCAACA GCTGCGTTGA AGGCTTGTTT AGCAACCGCT
Conservation
Sequence logo

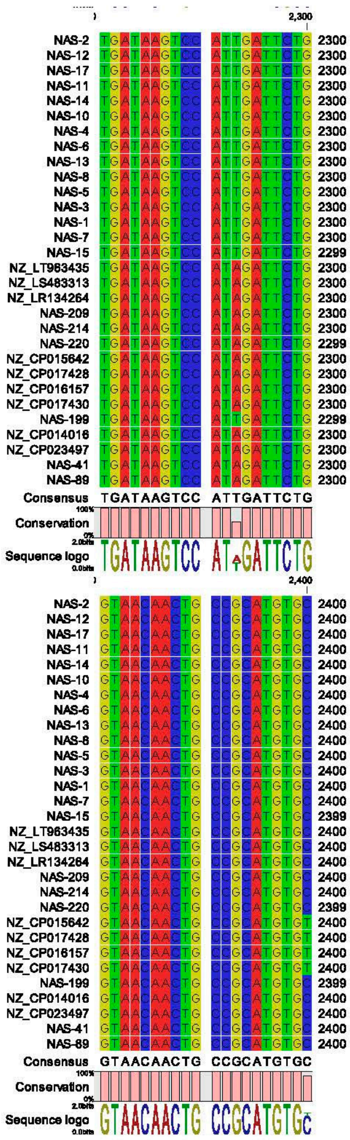

2,300
NAS-2 2300
NAS-12 2300
NAS-17 2300
NAS-11 2300
NAS-14 2300
NAS-10 2300
NAS-4 2300
NAS-6 2300
NAS-13 2300
NAS-8 2300
NAS-5 2300
NAS-3 2300
NAS-1 2300
NAS-7 2300
NAS-15 2299
NZ_LT963435 2300
NZ_LS483313 2300
NZ_LR134264 2300
NAS-209 2300
NAS-214 2300
NAS-220 2299
NZ_CP015642 2300
NZ_CP017428 2300
NZ_CP016157 2300
NZ_CP017430 2300
NAS-199 2299
NZ_CP014016 2300
NZ_CP023497 2300
NAS-41 2300
NAS-89 2300
Consensus TGATAAGTCC ATTGATTCTG
Conservation
Sequence logo
2,400
NAS-2 2400
NAS-12 2400
NAS-17 2400
NAS-11 2400
NAS-14 2400
NAS-10 2400
NAS-4 2400
NAS-6 2400
NAS-13 2400
NAS-8 2400
NAS-5 2400
NAS-3 2400
NAS-1 2400
NAS-7 2400
NAS-15 2399
NZ_LT963435 2400
NZ_LS483313 2400
NZ_LR134264 2400
NAS-209 2400
NAS-214 2400
NAS-220 2399
NZ_CP015642 2400
NZ_CP017428 2400
NZ_CP016157 2400
NZ_CP017430 2400
NAS-199 2399
NZ_CP014016 2400
NZ_CP023497 2400
NAS-41 2400
NAS-89 2400
Consensus GTAACAACTG CCGCATGTGC
Conservation
Sequence logo

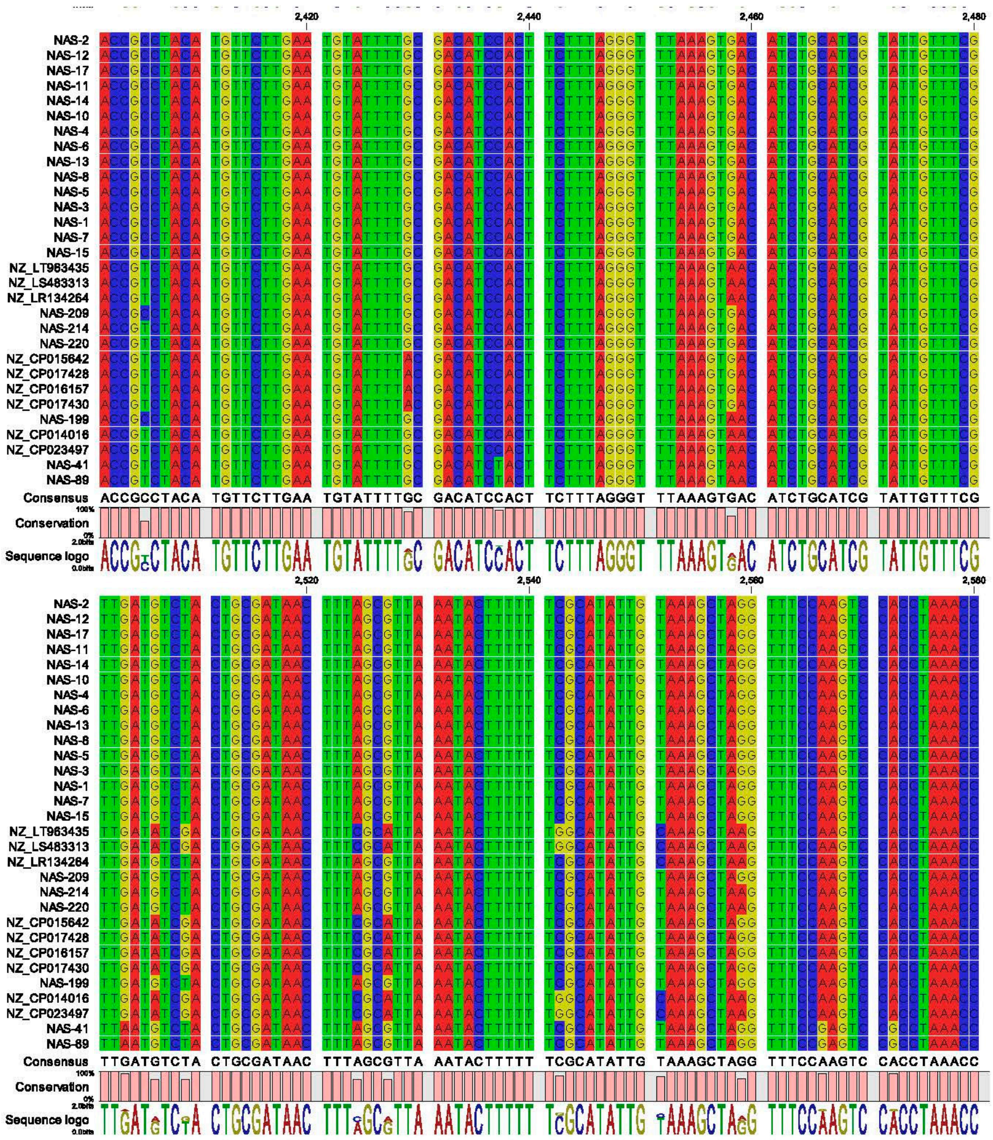

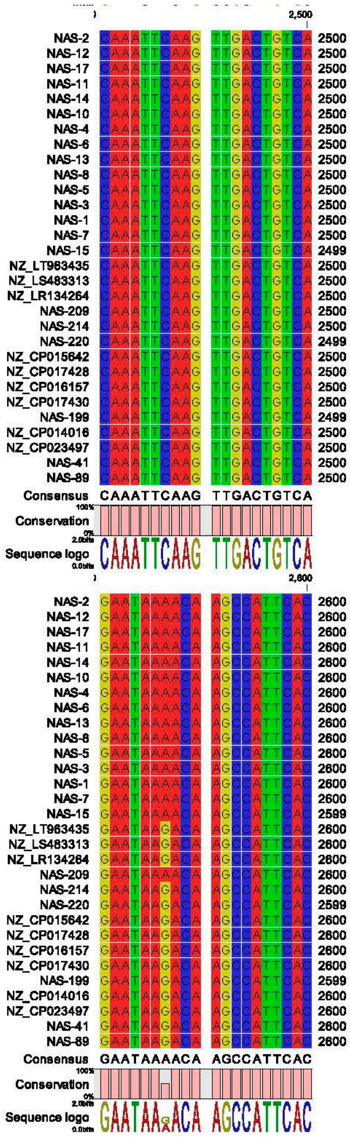

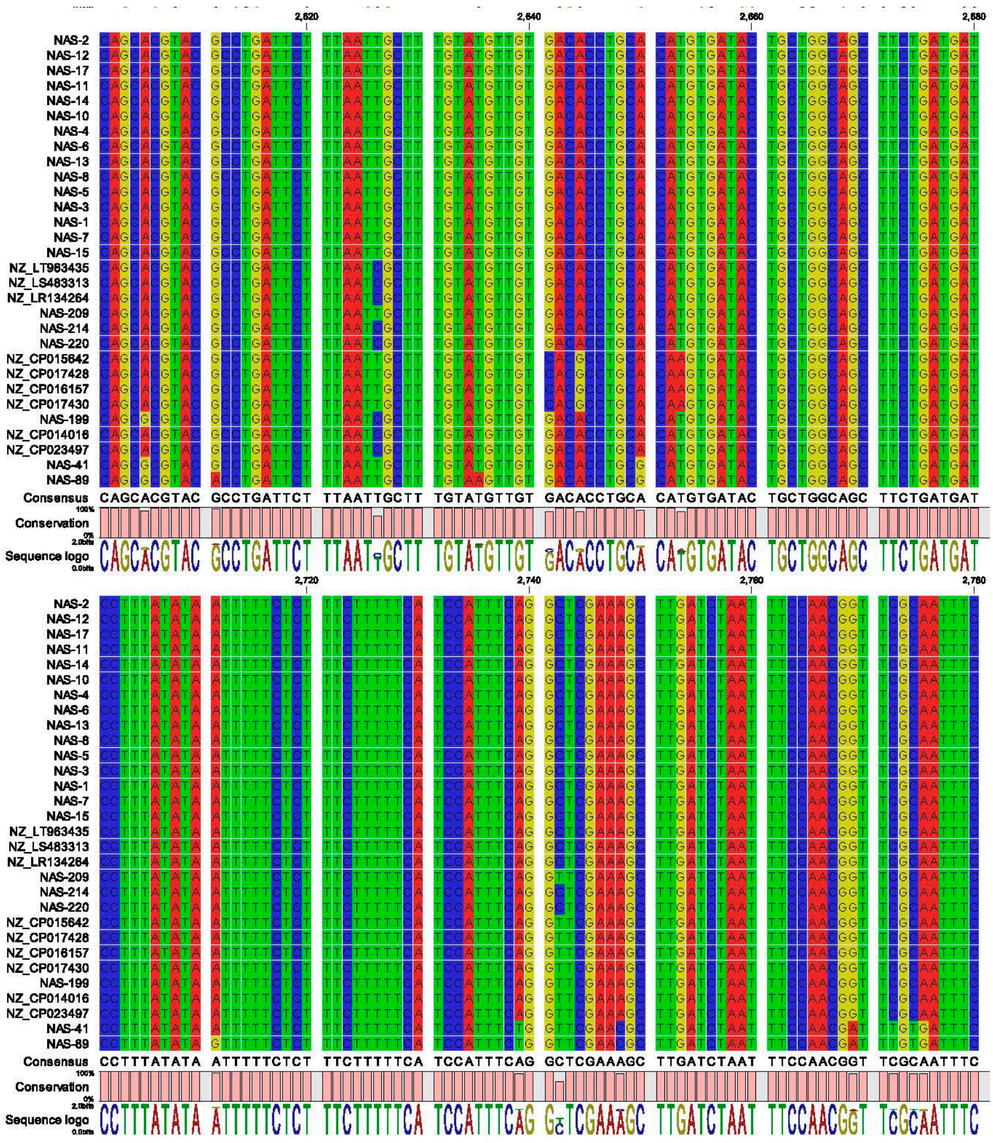
2,620
2,640
2,660
2,680
NAS-2
NAS-12
NAS-17
NAS-11
NAS-14
NAS-10
NAS-4
NAS-6
NAS-13
NAS-8
NAS-5
NAS-3
NAS-1
NAS-7
NAS-15
NZ_LT963435
NZ_LS483313
NZ_LR134264
NAS-209
NAS-214
NAS-220
NZ_CP015642
NZ_CP017428
NZ_CP016157
NZ_CP017430
NAS-199
NZ_CP014016
NZ_CP023497
NAS-41
NAS-89
Consensus CAGCACGTAC GCCTGATTCT TTAATTGCTT TGTATGTTGT GACACCTGCA CATGTGATAC TGCTGGCAGC TTCTGATGAT
Conservation
Sequence logo
2,720
2,740
2,760
2,780
NAS-2
NAS-12
NAS-17
NAS-11
NAS-14
NAS-10
NAS-4
NAS-6
NAS-13
NAS-8
NAS-5
NAS-3
NAS-1
NAS-7
NAS-15
NZ_LT963435
NZ_LS483313
NZ_LR134264
NAS-209
NAS-214
NAS-220
NZ_CP015642
NZ_CP017428
NZ_CP016157
NZ_CP017430
NAS-199
NZ_CP014016
NZ_CP023497
NAS-41
NAS-89
Consensus CCTTTATATA ATTTTTCTCT TTCTTTTTCA TCCATTTCAG GCTCGAAAGC TTGATCTAAT TTCCAACGGT TCGCAATTTC
Conservation
Sequence logo

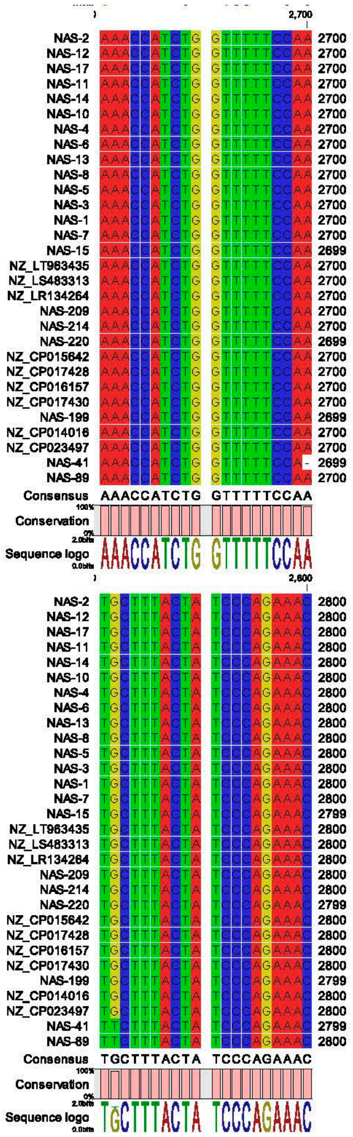
2,700
NAS-2
NAS-12
NAS-17
NAS-11
NAS-14
NAS-10
NAS-4
NAS-6
NAS-13
NAS-8
NAS-5
NAS-3
NAS-1
NAS-7
NAS-15
NZ_LT963435
NZ_LS483313
NZ_LR134264
NAS-209
NAS-214
NAS-220
NZ_CP015642
NZ_CP017428
NZ_CP016157
NZ_CP017430
NAS-199
NZ_CP014016
NZ_CP023497
NAS-41
NAS-89
Consensus AAACCATCTG GTTTTTCCAA
Conservation
Sequence logo
2,800
Consensus TGCTTTACTA TCCCAGAAAC
Conservation
Sequence logo

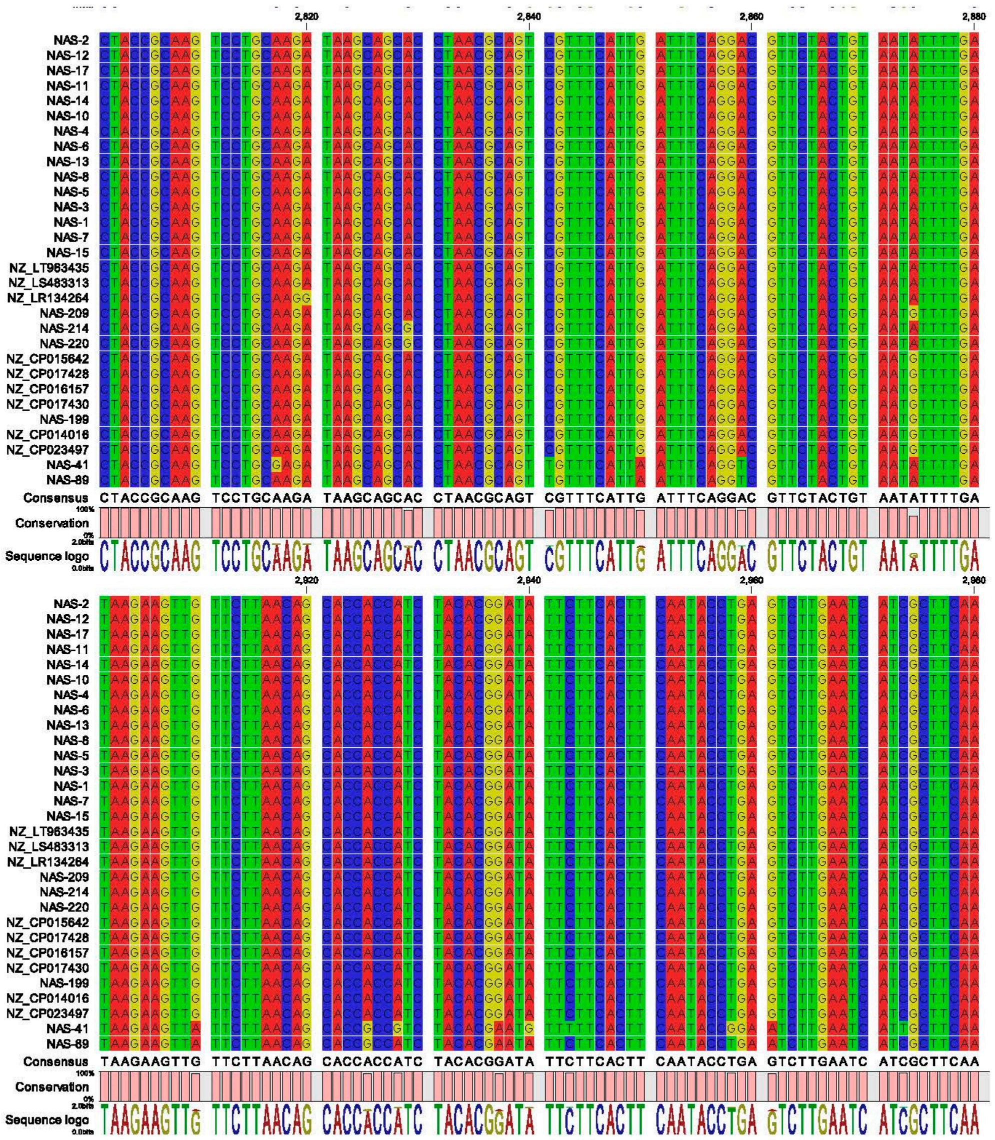
2,820
2,840
2,860
2,880
NAS-2
NAS-12
NAS-17
NAS-11
NAS-14
NAS-10
NAS-4
NAS-6
NAS-13
NAS-8
NAS-5
NAS-3
NAS-1
NAS-7
NAS-15
NZ_LT963435
NZ_LS483313
NZ_LR134264
NAS-209
NAS-214
NAS-220
NZ_CP015642
NZ_CP017428
NZ_CP016157
NZ_CP017430
NAS-199
NZ_CP014016
NZ_CP023497
NAS-41
NAS-89
Consensus CTACCGCAAG TCCTGCAAGA TAAGCAGCAC CTAACGCAGT CGTTTCATTG ATTTCAGGAC GTTCTACTGT AATATTTTGA
Conservation
Sequence logo
2,920
2,940
2,960
2,980
Consensus TAAGAAGTTG TTCTTAACAG CACCACCATC TACACGGATA TTCTTCACTT CAATACCTGA GTCTTGAATC ATCGCTTCAA
Conservation
Sequence logo

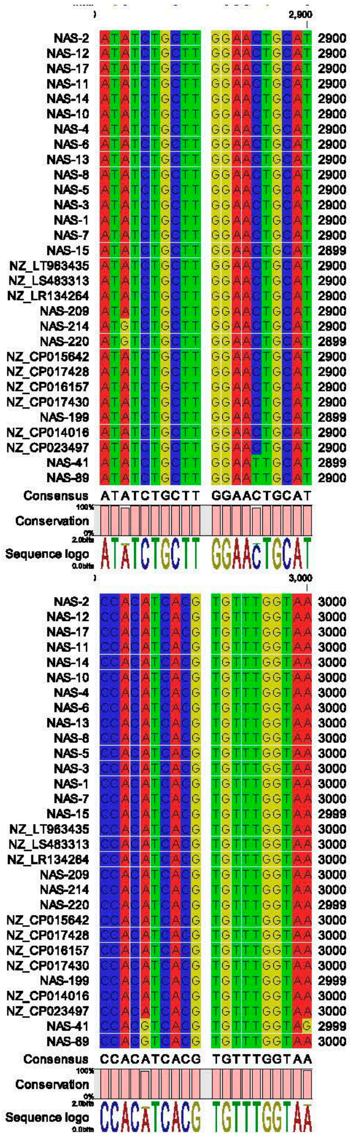

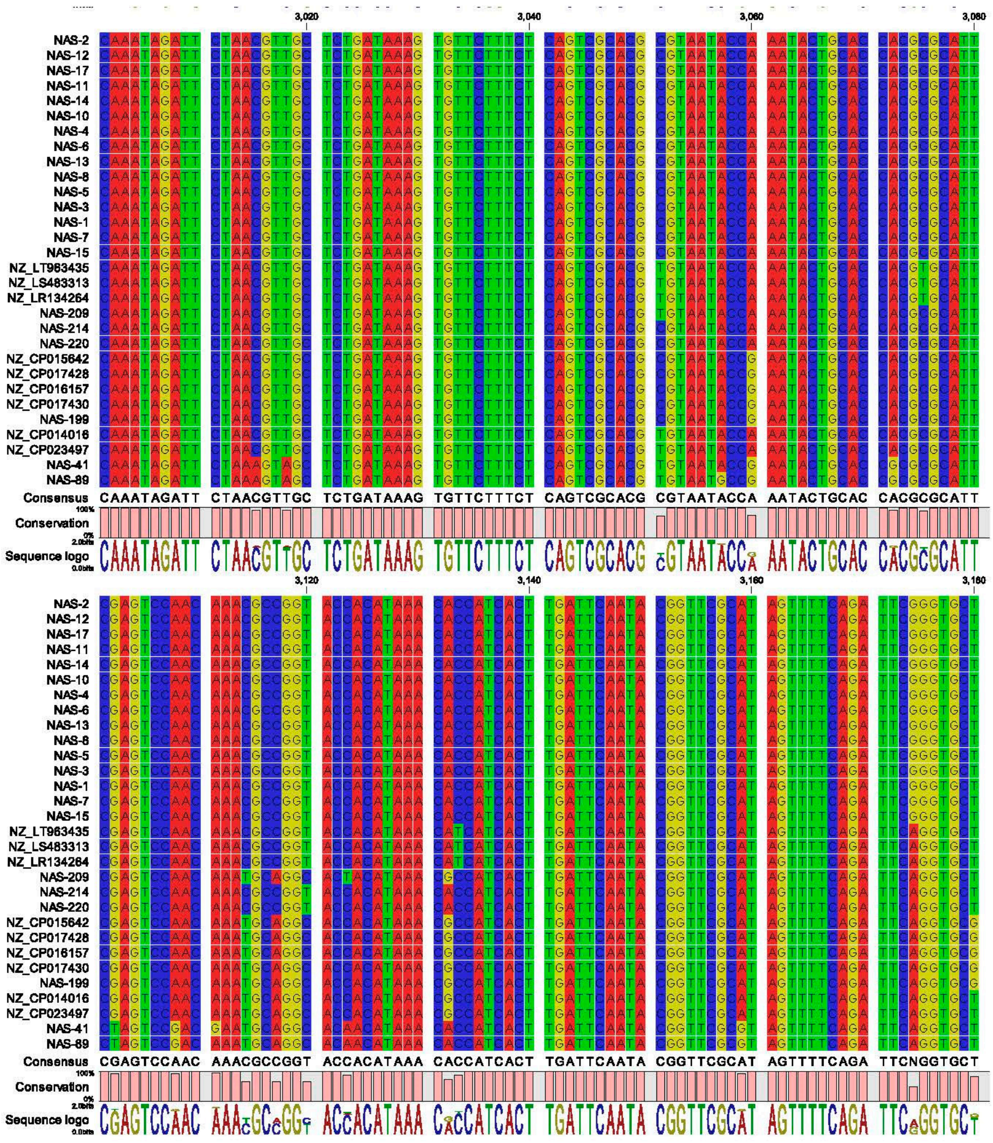
3,020
3,040
3,060
3,080
NAS-2
NAS-12
NAS-17
NAS-11
NAS-14
NAS-10
NAS-4
NAS-6
NAS-13
NAS-8
NAS-5
NAS-3
NAS-1
NAS-7
NAS-15
NZ_LT963435
NZ_LS483313
NZ_LR134264
NAS-209
NAS-214
NAS-220
NZ_CP015642
NZ_CP017428
NZ_CP016157
NZ_CP017430
NAS-199
NZ_CP014016
NZ_CP023497
NAS-41
NAS-89
Consensus CAAATAGATT CTAACGTTGC TCTGATAAAG TGTTCTTTCT CAGTCGCACG CGTAATACCA AATACTGCAC CACGCGCATT
Conservation
Sequence logo
3,120
3,140
3,160
3,180
Consensus CGAGTCCAAC AAACGCCGGT ACCACATAAA CACCATCACT TGATTCAATA CGGTTCGCAT AGTTTTCAGA TTCNGGTGCT
Conservation
Sequence logo

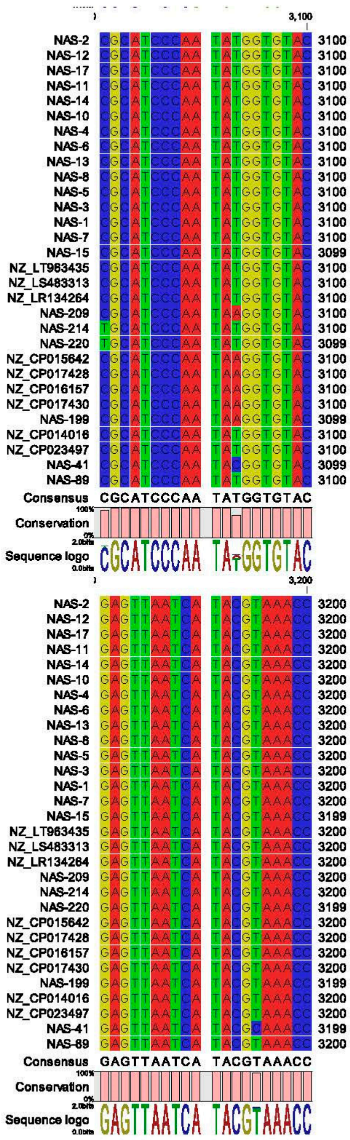

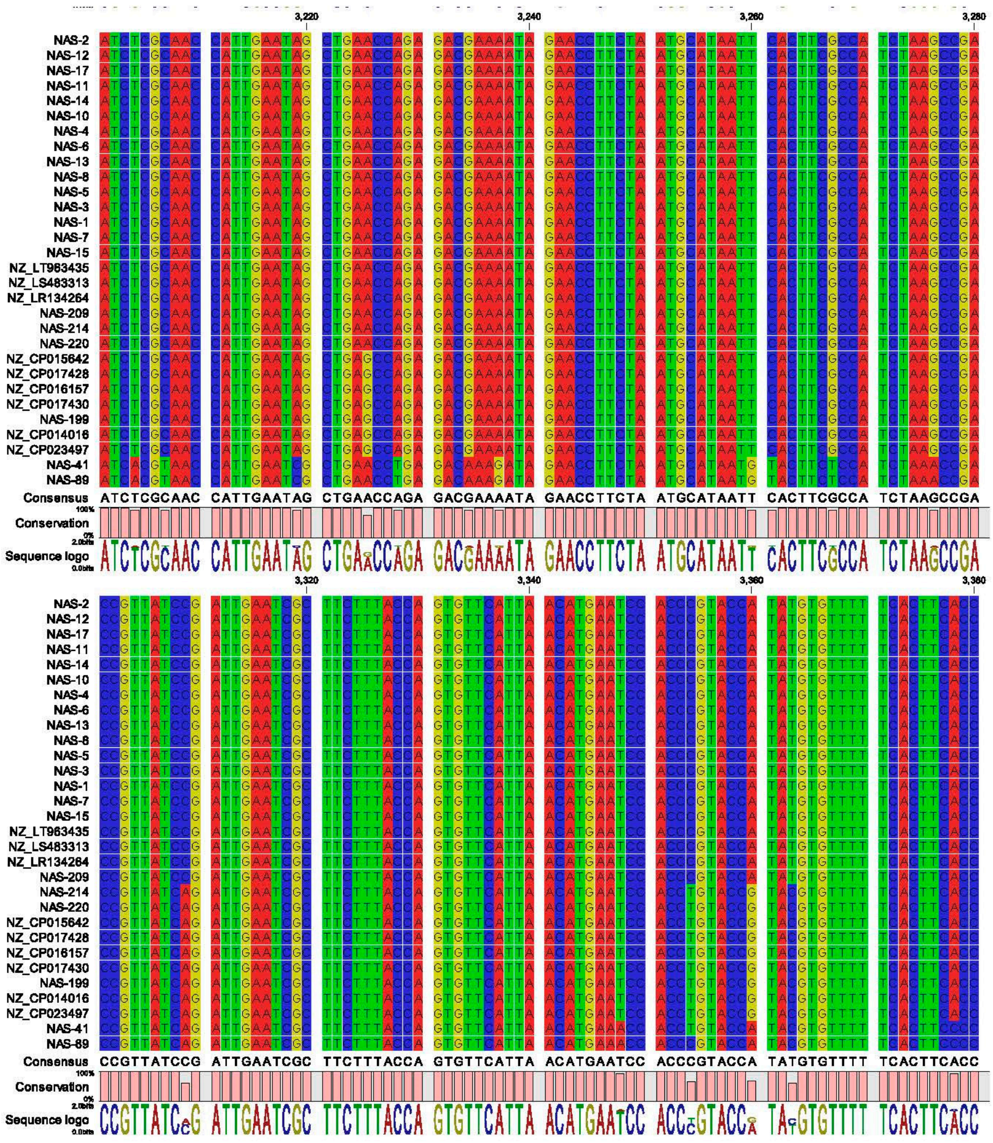
3,220
3,240
3,260
3,280
NAS-2
NAS-12
NAS-17
NAS-11
NAS-14
NAS-10
NAS-4
NAS-6
NAS-13
NAS-8
NAS-5
NAS-3
NAS-1
NAS-7
NAS-15
NZ_LT963435
NZ_LS483313
NZ_LR134264
NAS-209
NAS-214
NAS-220
NZ_CP015642
NZ_CP017428
NZ_CP016157
NZ_CP017430
NAS-199
NZ_CP014016
NZ_CP023497
NAS-41
NAS-89
Consensus ATCTCGCAAC CATTGAATAG CTGAACCAGA GACGAAAATA GAACCTTCTA ATGCATAATT CACTTCGCCA TCTAAGCCGA
Conservation
Sequence logo
3,320
3,340
3,360
3,380
Consensus CCGTTATCCG ATTGAATCGC TTCTTTACCA GTGTTCATTA ACATGAATCC ACCCGTACCA TATGTGTTTT TCACTTCACC
Conservation
Sequence logo

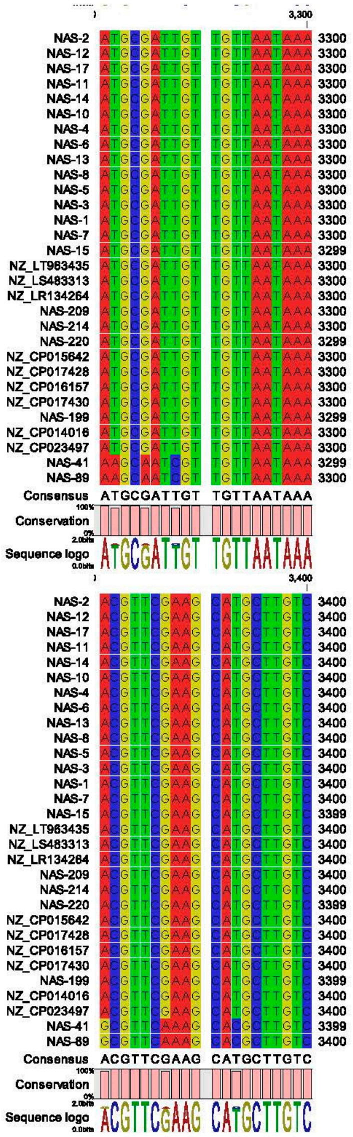
3,300
NAS-2
NAS-12
NAS-17
NAS-11
NAS-14
NAS-10
NAS-4
NAS-6
NAS-13
NAS-8
NAS-5
NAS-3
NAS-1
NAS-7
NAS-15
NZ_LT963435
NZ_LS483313
NZ_LR134264
NAS-209
NAS-214
NAS-220
NZ_CP015642
NZ_CP017428
NZ_CP016157
NZ_CP017430
NAS-199
NZ_CP014016
NZ_CP023497
NAS-41
NAS-89
Consensus
ATGCGATTGT TGTTAATAAA
Conservation
Sequence logo
3,400
Consensus
ACGTTCGAAG CATGCTTGTC
Conservation
Sequence logo

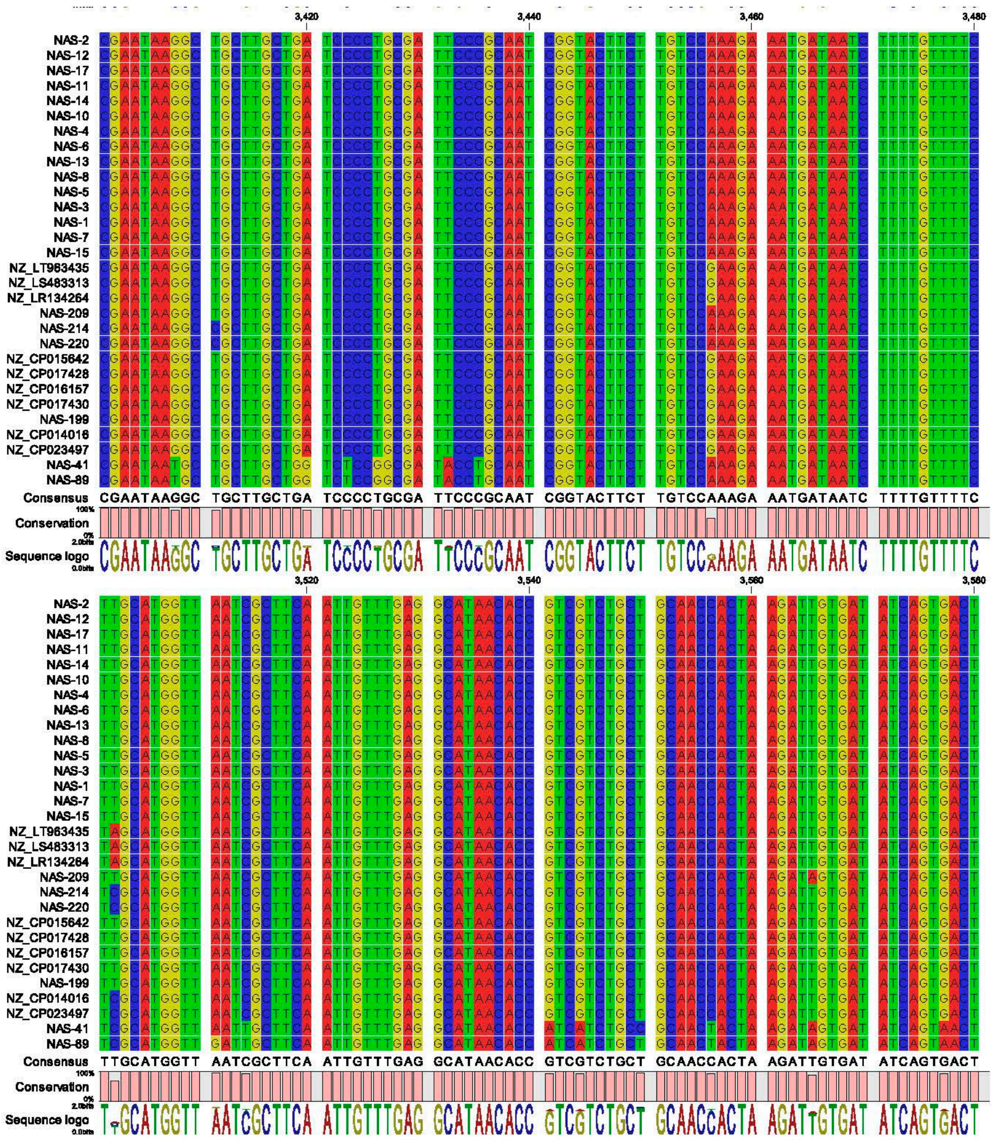

NAS-2
NAS-12
NAS-17
NAS-11
NAS-14
NAS-10
NAS-4
NAS-6
NAS-13
NAS-8
NAS-5
NAS-3
NAS-1
NAS-7
NAS-15
NZ_LT963435
NZ_LS483313
NZ_LR134264
NAS-209
NAS-214
NAS-220
NZ_CP015642
NZ_CP017428
NZ_CP016157
NZ_CP017430
NAS-199
NZ_CP014016
NZ_CP023497
NAS-41
NAS-89
Consensus
Conservation
Sequence logo
CGAATAAGGC TGCTTGCTGA TCCCCTGCGA TTCCCGCAAT CGGTACTTCT TGTCCAAAGA AATGATAATC TTTTGTTTTC
TTGCATGGTT AATCGCTTCA ATTGTTTGAG GCATAACACC GTCGTCTGCT GCAACCACTA AGATTGTGAT ATCAGTGACT
3,420
3,440
3,460
3,480
3,520
3,540
3,560
3,580

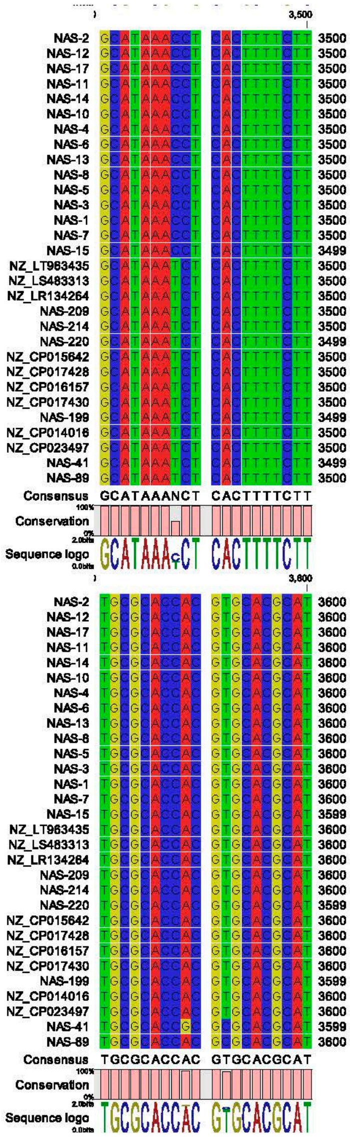
3,500
NAS-2 3500
NAS-12 3500
NAS-17 3500
NAS-11 3500
NAS-14 3500
NAS-10 3500
NAS-4 3500
NAS-6 3500
NAS-13 3500
NAS-8 3500
NAS-5 3500
NAS-3 3500
NAS-1 3500
NAS-7 3500
NAS-15 3499
NZ_LT963435 3500
NZ_LS483313 3500
NZ_LR134264 3500
NAS-209 3500
NAS-214 3500
NAS-220 3499
NZ_CP015642 3500
NZ_CP017428 3500
NZ_CP016157 3500
NZ_CP017430 3500
NAS-199 3499
NZ_CP014016 3500
NZ_CP023497 3500
NAS-41 3499
NAS-89 3500
Consensus GCATAAANCT CACTTTTCTT
Conservation
Sequence logo
3,600
NAS-2 3600
NAS-12 3600
NAS-17 3600
NAS-11 3600
NAS-14 3600
NAS-10 3600
NAS-4 3600
NAS-6 3600
NAS-13 3600
NAS-8 3600
NAS-5 3600
NAS-3 3600
NAS-1 3600
NAS-7 3600
NAS-15 3599
NZ_LT963435 3600
NZ_LS483313 3600
NZ_LR134264 3600
NAS-209 3600
NAS-214 3600
NAS-220 3599
NZ_CP015642 3600
NZ_CP017428 3600
NZ_CP016157 3600
NZ_CP017430 3600
NAS-199 3599
NZ_CP014016 3600
NZ_CP023497 3600
NAS-41 3599
NAS-89 3600
Consensus TGCGCACCAC GTGCACGCAT
Conservation
Sequence logo

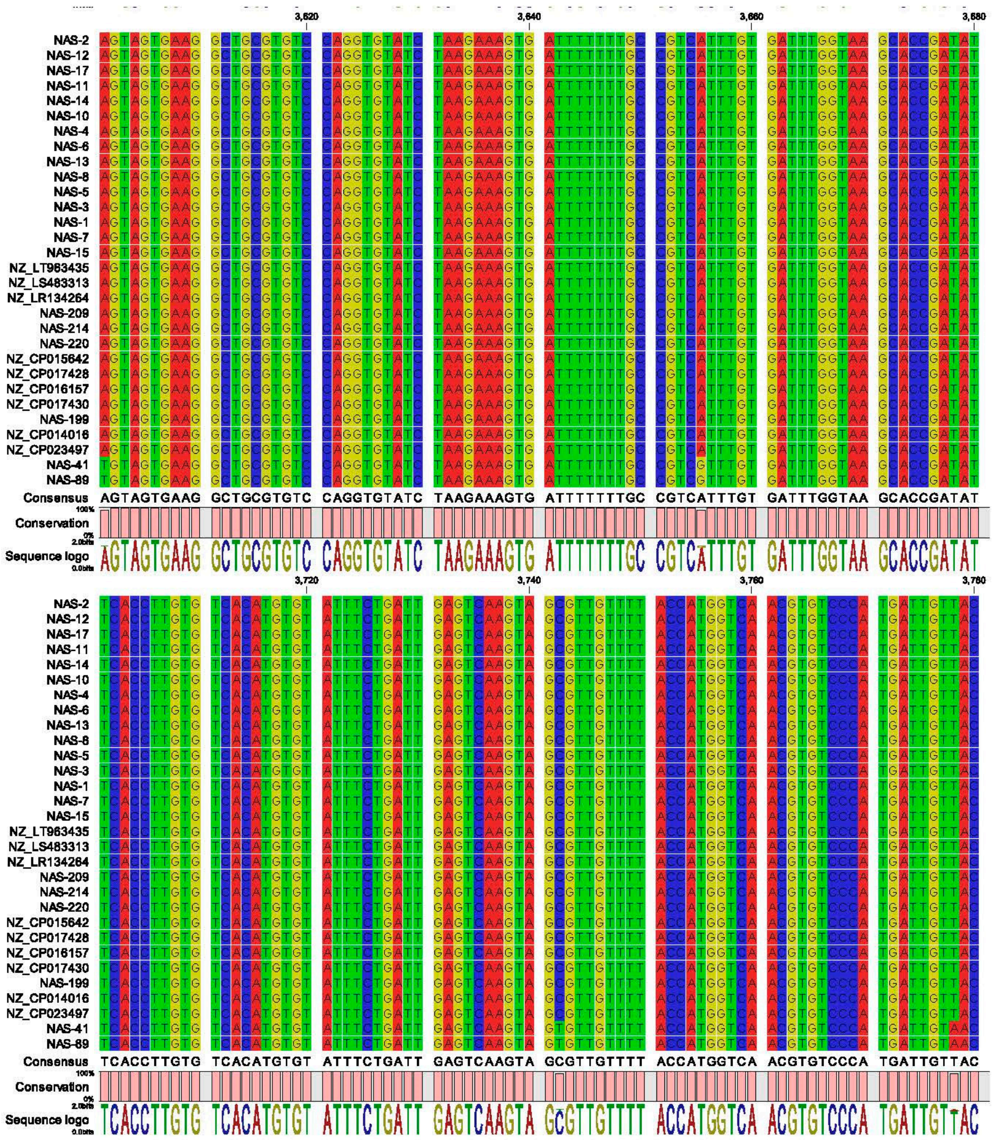

3,620
3,640
3,660
3,680
NAS-2
NAS-12
NAS-17
NAS-11
NAS-14
NAS-10
NAS-4
NAS-6
NAS-13
NAS-8
NAS-5
NAS-3
NAS-1
NAS-7
NAS-15
NZ_LT963435
NZ_LS483313
NZ_LR134264
NAS-209
NAS-214
NAS-220
NZ_CP015642
NZ_CP017428
NZ_CP016157
NZ_CP017430
NAS-199
NZ_CP014016
NZ_CP023497
NAS-41
NAS-89
Consensus AGTAGTGAAG GCTGCGTGTC CAGGTGTATC TAAGAAAGTG ATTTTTTTGC CGTCATTTGT GATTTGGTAA GCACCGATAT
Conservation
Sequence logo
3,720
3,740
3,760
3,780
Consensus TCACCTTGTG TCACATGTGT ATTTCTGATT GAGTCAAGTA GCGTTGTTTT ACCATGGTCA ACGTGTCCCA TGATTGTTAC
Conservation
Sequence logo

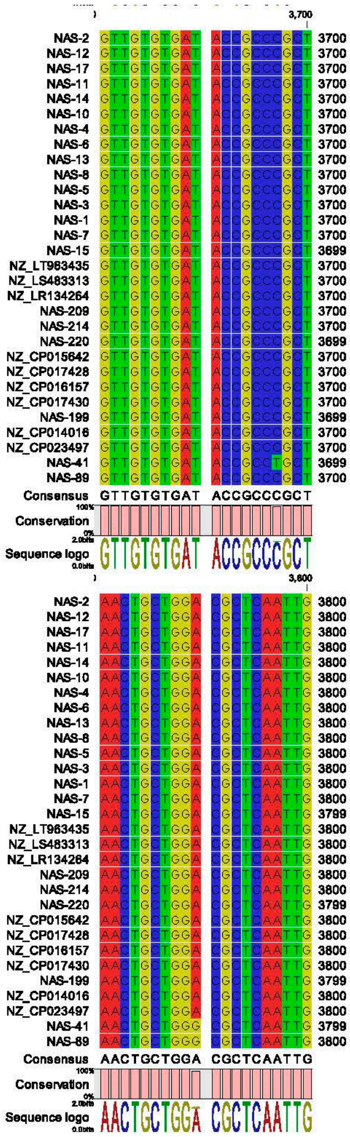
3,700
NAS-2 3700
NAS-12 3700
NAS-17 3700
NAS-11 3700
NAS-14 3700
NAS-10 3700
NAS-4 3700
NAS-6 3700
NAS-13 3700
NAS-8 3700
NAS-5 3700
NAS-3 3700
NAS-1 3700
NAS-7 3700
NAS-15 3699
NZ_LT963435 3700
NZ_LS483313 3700
NZ_LR134264 3700
NAS-209 3700
NAS-214 3700
NAS-220 3699
NZ_CP015642 3700
NZ_CP017428 3700
NZ_CP016157 3700
NZ_CP017430 3700
NAS-199 3699
NZ_CP014016 3700
NZ_CP023497 3700
NAS-41 3699
NAS-89 3700
Consensus GTTGTGTGAT ACCGCCCGCT
Conservation
Sequence logo
3,800
NAS-2 3800
NAS-12 3800
NAS-17 3800
NAS-11 3800
NAS-14 3800
NAS-10 3800
NAS-4 3800
NAS-6 3800
NAS-13 3800
NAS-8 3800
NAS-5 3800
NAS-3 3800
NAS-1 3800
NAS-7 3800
NAS-15 3799
NZ_LT963435 3800
NZ_LS483313 3800
NZ_LR134264 3800
NAS-209 3800
NAS-214 3800
NAS-220 3799
NZ_CP015642 3800
NZ_CP017428 3800
NZ_CP016157 3800
NZ_CP017430 3800
NAS-199 3799
NZ_CP014016 3800
NZ_CP023497 3800
NAS-41 3799
NAS-89 3800
Consensus AACTGCTGGA CGCTCAATTG
Conservation
Sequence logo

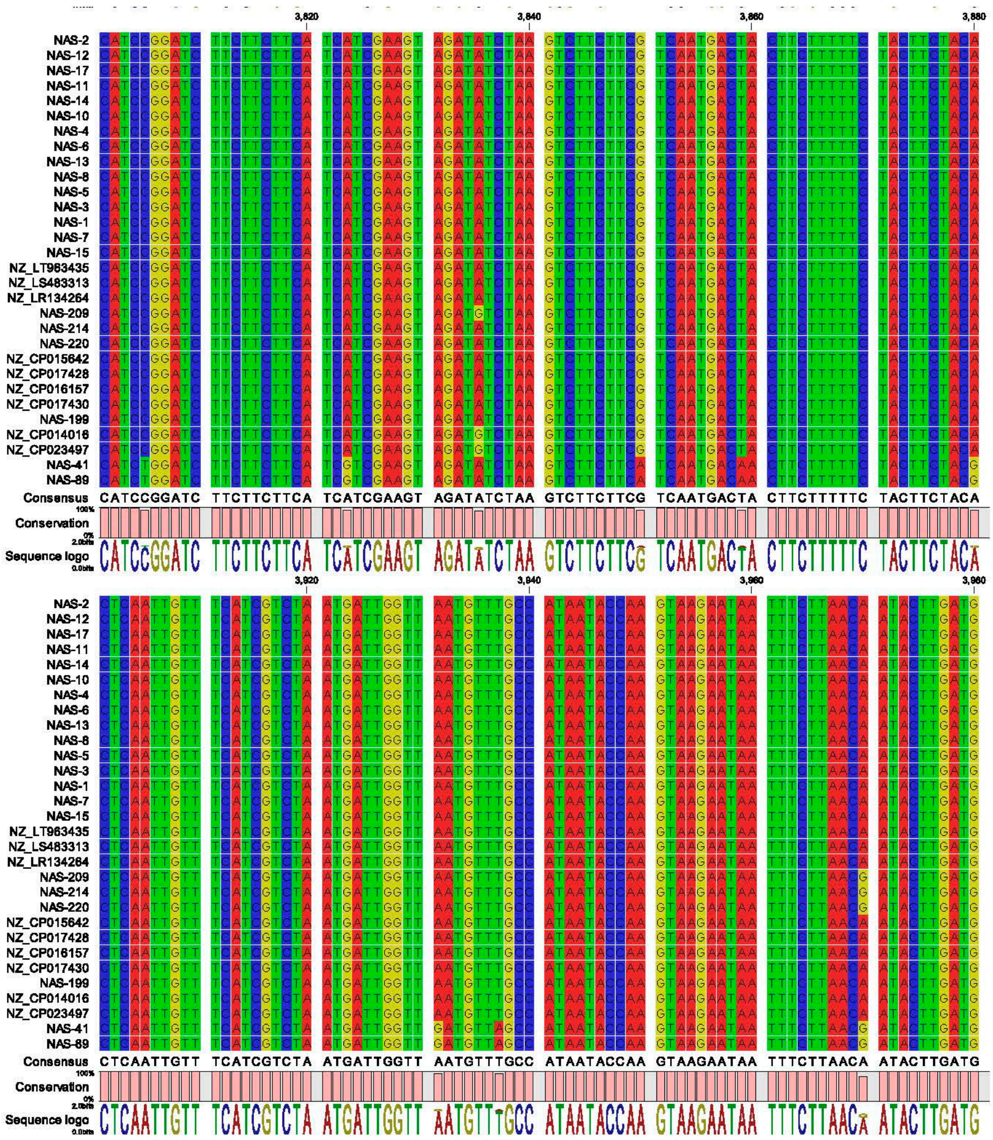
3,820
3,840
3,860
3,880
NAS-2
NAS-12
NAS-17
NAS-11
NAS-14
NAS-10
NAS-4
NAS-6
NAS-13
NAS-8
NAS-5
NAS-3
NAS-1
NAS-7
NAS-15
NZ_LT963435
NZ_LS483313
NZ_LR134264
NAS-209
NAS-214
NAS-220
NZ_CP015642
NZ_CP017428
NZ_CP016157
NZ_CP017430
NAS-199
NZ_CP014016
NZ_CP023497
NAS-41
NAS-89
Consensus CATCCGGATC TTCTTCTTCA TCATCGAAGT AGATATCTAA GTCTTCTTCG TCAATGACTA CTTCTTTTTC TACTTCTACA
Conservation
Sequence logo
3,920
3,940
3,960
3,980
Consensus CTCAATTGTT TCATCGTCTA ATGATTGGTT AATGTTTGCC ATAATACCAA GTAAGAATAA TTTCTTAACA ATACTTGATG
Conservation
Sequence logo

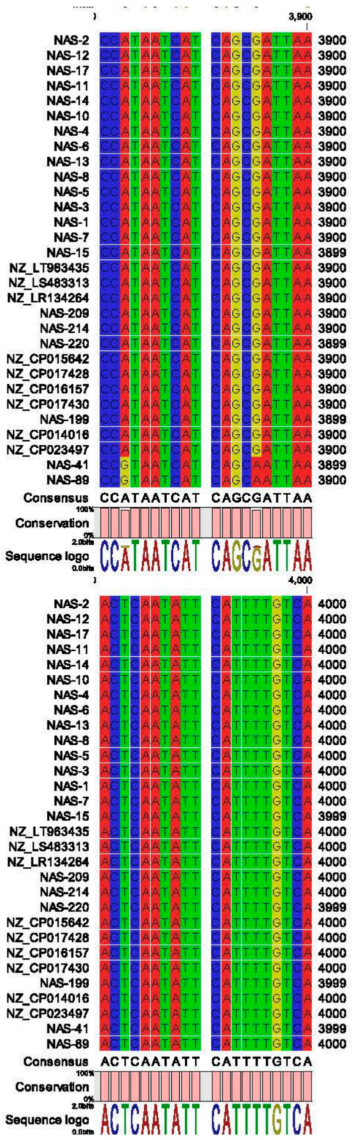
3,900
NAS-2 3900
NAS-12 3900
NAS-17 3900
NAS-11 3900
NAS-14 3900
NAS-10 3900
NAS-4 3900
NAS-6 3900
NAS-13 3900
NAS-8 3900
NAS-5 3900
NAS-3 3900
NAS-1 3900
NAS-7 3900
NAS-15 3899
NZ_LT963435 3900
NZ_LS483313 3900
NZ_LR134264 3900
NAS-209 3900
NAS-214 3900
NAS-220 3899
NZ_CP015642 3900
NZ_CP017428 3900
NZ_CP016157 3900
NZ_CP017430 3900
NAS-199 3899
NZ_CP014016 3900
NZ_CP023497 3900
NAS-41 3899
NAS-89 3900
Consensus CCATAATCAT CAGCGATTAA
Conservation
Sequence logo
4,000
NAS-2 4000
NAS-12 4000
NAS-17 4000
NAS-11 4000
NAS-14 4000
NAS-10 4000
NAS-4 4000
NAS-6 4000
NAS-13 4000
NAS-8 4000
NAS-5 4000
NAS-3 4000
NAS-1 4000
NAS-7 4000
NAS-15 3999
NZ_LT963435 4000
NZ_LS483313 4000
NZ_LR134264 4000
NAS-209 4000
NAS-214 4000
NAS-220 3999
NZ_CP015642 4000
NZ_CP017428 4000
NZ_CP016157 4000
NZ_CP017430 4000
NAS-199 3999
NZ_CP014016 4000
NZ_CP023497 4000
NAS-41 3999
NAS-89 4000
Consensus ACTCAATATT CATTTTGTCA
Conservation
Sequence logo

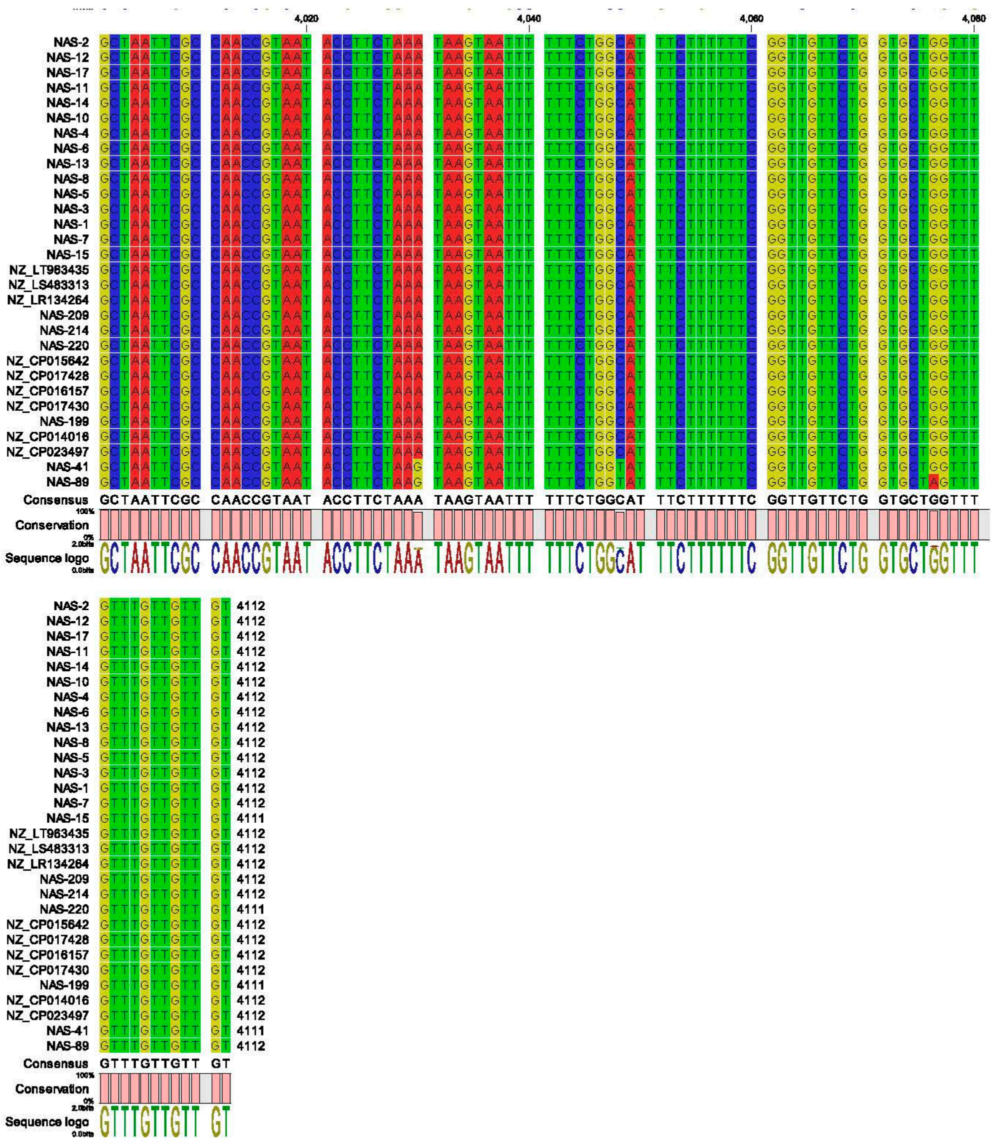
4,020
4,040
4,060
4,080
NAS-2
NAS-12
NAS-17
NAS-11
NAS-14
NAS-10
NAS-4
NAS-6
NAS-13
NAS-8
NAS-5
NAS-3
NAS-1
NAS-7
NAS-15
NZ_LT963435
NZ_LS483313
NZ_LR134264
NAS-209
NAS-214
NAS-220
NZ_CP015642
NZ_CP017428
NZ_CP016157
NZ_CP017430
NAS-199
NZ_CP014016
NZ_CP023497
NAS-41
NAS-89
Consensus GCTAATTCGC CAACCGTAAT ACCTTCTAAA TAAGTAATTT TTTCTGGCAT TTCTTTTTTC GGTTGTTCTG GTGCTGGTTT
Conservation
Sequence logo
Consensus GTTTGTTGTT GT
4112
4112
4112
4112
4112
4112
4112
4112
4112
4112
4112
4112
4112
4112
4111
4112
4112
4112
4112
4112
4111
4112
4112
4112
4112
4111
4112
4112
4111
4112

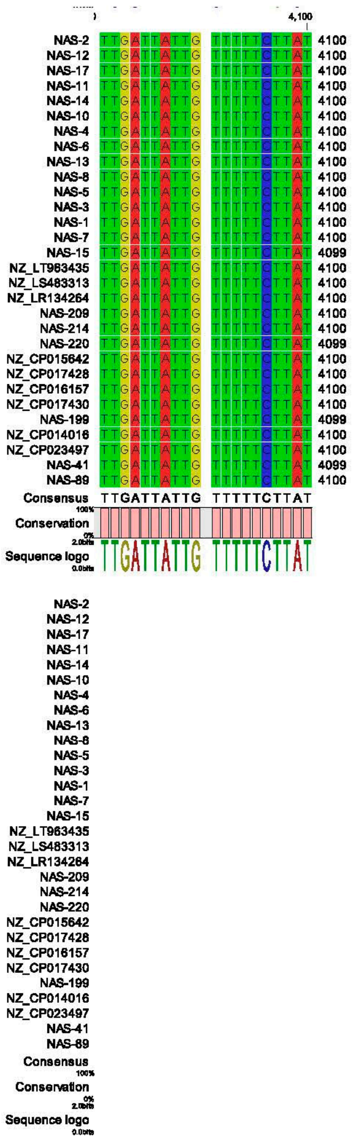

4,100
NAS-2 TTGATTATTG TTTTTCTTAT 4100
NAS-12 TTGATTATTG TTTTTCTTAT 4100
NAS-17 TTGATTATTG TTTTTCTTAT 4100
NAS-11 TTGATTATTG TTTTTCTTAT 4100
NAS-14 TTGATTATTG TTTTTCTTAT 4100
NAS-10 TTGATTATTG TTTTTCTTAT 4100
NAS-4 TTGATTATTG TTTTTCTTAT 4100
NAS-6 TTGATTATTG TTTTTCTTAT 4100
NAS-13 TTGATTATTG TTTTTCTTAT 4100
NAS-8 TTGATTATTG TTTTTCTTAT 4100
NAS-5 TTGATTATTG TTTTTCTTAT 4100
NAS-3 TTGATTATTG TTTTTCTTAT 4100
NAS-1 TTGATTATTG TTTTTCTTAT 4100
NAS-7 TTGATTATTG TTTTTCTTAT 4100
NAS-15 TTGATTATTG TTTTTCTTAT 4099
NZ_LT963435 TTGATTATTG TTTTTCTTAT 4100
NZ_LS483313 TTGATTATTG TTTTTCTTAT 4100
NZ_LR134264 TTGATTATTG TTTTTCTTAT 4100
NAS-209 TTGATTATTG TTTTTCTTAT 4100
NAS-214 TTGATTATTG TTTTTCTTAT 4100
NAS-220 TTGATTATTG TTTTTCTTAT 4099
NZ_CP015642 TTGATTATTG TTTTTCTTAT 4100
NZ_CP017428 TTGATTATTG TTTTTCTTAT 4100
NZ_CP016157 TTGATTATTG TTTTTCTTAT 4100
NZ_CP017430 TTGATTATTG TTTTTCTTAT 4100
NAS-199 TTGATTATTG TTTTTCTTAT 4099
NZ_CP014016 TTGATTATTG TTTTTCTTAT 4100
NZ_CP023497 TTGATTATTG TTTTTCTTAT 4100
NAS-41 TTGATTATTG TTTTTCTTAT 4099
NAS-89 TTGATTATTG TTTTTCTTAT 4100
Consensus TTGATTATTG TTTTTCTTAT
100%
Conservation
0%
2.0bits
Sequence logo TTGATTATTG TTTTTCTTAT
0.0bits
NAS-2
NAS-12
NAS-17
NAS-11
NAS-14
NAS-10
NAS-4
NAS-6
NAS-13
NAS-8
NAS-5
NAS-3
NAS-1
NAS-7
NAS-15
NZ_LT963435
NZ_LS483313
NZ_LR134264
NAS-209
NAS-214
NAS-220
NZ_CP015642
NZ_CP017428
NZ_CP016157
NZ_CP017430
NAS-199
NZ_CP014016
NZ_CP023497
NAS-41
NAS-89
Consensus
100%
Conservation
0%
2.0bits
Sequence logo
0.0bits
